# Differential evolutionary patterns and expression levels between sex-specific and somatic tissue-specific genes in peanut

**DOI:** 10.1038/s41598-017-09905-8

**Published:** 2017-08-21

**Authors:** Hui Song, Qingping Zhang, Pei Tian, Zhibiao Nan

**Affiliations:** State Key Laboratory of Grassland Agro-ecosystems, College of Pastoral Agriculture Science and Technology, Lanzhou University, Lanzhou, 730000 China

## Abstract

The patterns of evolution and expression of tissue-specific genes are poorly understood beyond sex-specific genes. Accordingly, we identified 3,191 tissue-specific genes and 38,745 common genes using 22 RNA-seq datasets from cultivated peanut. The expression levels of tissue-specific genes were significantly lower than those of common genes. Further, the expression levels of sex-specific genes were significantly higher than those of somatic tissue-specific genes. Among sex-specific genes, the expression levels of gynoecium-specific genes were significantly higher than those of androecium-specific genes. Function-specific genes were lacking among tissue-specific genes, and tissue-specific gene annotations overlapped among different tissues. Duplicate gene pairs were classified as homogeneous pairs expressed within the same tissue or heterogeneous pairs expressed in different tissues. Heterogeneous gene pairs evolved more rapidly than homogeneous gene pairs. In addition, somatic tissue-specific genes evolved faster than sex-specific genes. Molecular signatures of selection indicated that somatic tissue-specific genes have mainly experienced relaxed selection, while sex-specific genes have been under stronger selective constraint. Somatic tissue-specific genes had higher codon usage bias than sex-specific genes. These contrasting patterns between somatic tissue-specific and sex-specific genes provide new insights into the basic biology and evolution of peanut, an important crop.

## Introduction

Since the publication of the *Arabidopsis thaliana* genome, many genomic sequences and RNA-seq datasets of other plants have been released. Subsequently, researchers have used these datasets to gain insight into plant growth and development as well as other biological processes. In recent studies, sex-biased genes that are more highly expressed in sexual tissue, including female-biased and male-biased genes, have attracted the interest of researchers because sex-biased expression is fundamentally linked to gene regulation, epigenetics, developmental biology, and sexual antagonism^1^. Sex-biased studies have been reported in humans and animals such as mammals, birds, fish, and fruit flies^1, 2^. Similar studies have been lacking in plants. However, studies examining the evolutionary patterns and expression levels of sex-biased genes have been reported for *A*. *thaliana* ^3^, *Capsella grandiflora* ^4^, *Collinsia heterophylla* ^5^, *Ectocarpus* spp.^6^, and *Silene latifolia* ^7^. Some consistent conclusions have arisen from these analyses of sex-biased genes in plants. First, sex-biased genes tend to evolve more rapidly than unbiased genes^4, 6^. Second, sex-biased genes have mainly experienced adaptive evolution^3, 6, 8^. Third, female-biased gene expression is higher than male-biased gene expression^3^. In addition, female-biased genes tend to be highly expressed in specific tissues, while male-biased genes tend to be expressed at low levels in the same tissues^6, 7^. These findings support the hypothesis that differential expression of female-biased and male-biased genes may resolve sexual antagonism under natural selection and sexual selection^1, 2, 7^. Fourth, a greater bias in codon usage has been observed among female tissues relative to male tissues in *Zea mays* and *Triticum aestivum* ^9^.

However, little is known about the evolutionary patterns and expression levels of tissue-specific genes in plants. There has been a particular lack in comparisons of these two aspects between sex-specific and somatic tissue-specific genes. These comparisons could not be made without RNA-seq datasets of both somatic tissue-specific and sex-specific tissue, especially datasets that focus on male-specific and female-specific tissues. To date, RNA-seq datasets based on cultivated peanut have examined 22 different tissues, including both sexual and somatic tissues^10^. These datasets are a powerful resource for research into the evolutionary patterns and gene expression levels exhibited in sex-specific and somatic tissue-specific genes. We found that different evolutionary patterns and gene expression levels are observed between sex-specific and somatic tissue-specific genes based on these RNA-seq datasets. Overall sex-specific genes were expressed at higher levels than somatic tissue-specific genes. Furthermore, somatic tissue-specific genes experienced relaxed selection, while sex-specific genes have been subject to stronger selective constraint.

## Results

### Tissue-specific genes in cultivated peanut

**Figure 1:**
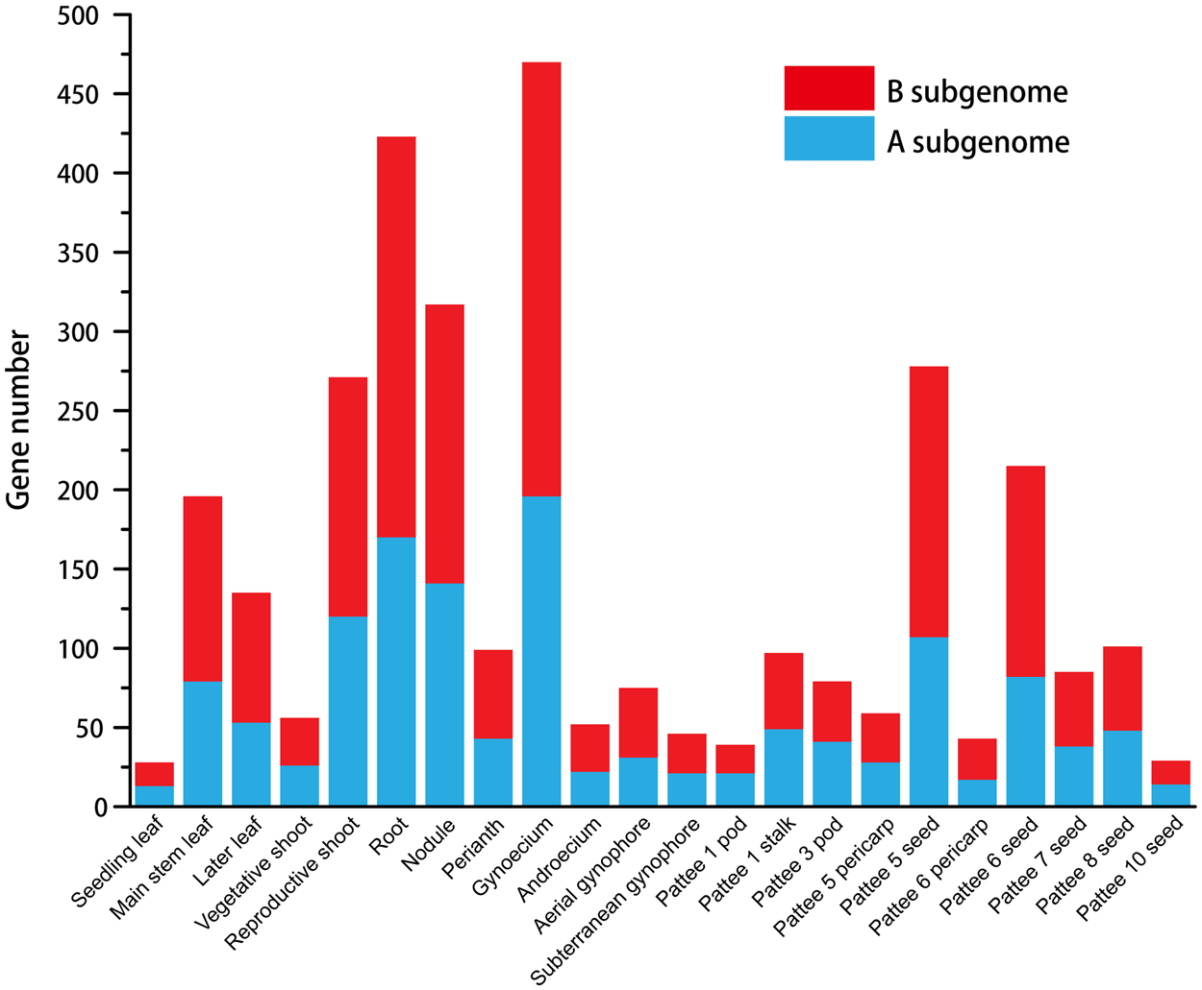
The number of tissue-specific genes in cultivated peanut.

A total of 3,191 tissue-specific genes were identified from 22 RNA-seq datasets for cultivated peanut (Table S1). The largest number of tissue-specific genes were expressed in gynoecium tissue, while the fewest tissue-specific genes were expressed in seedling leaf tissue (Fig. 1). The descending order of tissues ranked by number of tissue-specific genes expressed in them was gynoecium, root, nodule, Pattee 5 seed, reproductive shoot, Pattee 6 seed, main stem leaf, later leaf, Pattee 8 seed, perianth, stalk, Pattee 7 seed, Pattee 3 pod, aerial gynophores, Pattee 5 pericarp, vegetative shoot, androecium, subterranean gynophore, Pattee 6 pericarp, Pattee 1 pod, Pattee 10 seed, and seedling leaf tissue (Fig. 1). RNA-seq data for the leaf, shoot, gynophore, pod, pericarp, and seed can be classified into three, two, two, three, two, and five developmental stages, respectively (Fig. 1 and Table S1). If different developmental stages were considered as individual tissues, we could obtain nine leaf-specific, seventeen shoot-specific, two gynophore-specific, four pod-specific, three pericarp-specific, and twenty-five seed-specific genes, respectively (Table S2). In this study, we used different developmental stages of tissues as the level of analysis because genes can vary in spatial and temporal expression. Sex-specific genes can be expressed at a particular developmental stage without being expressed in later stages^1^. (These genes are available as supplemental material in Table S2 and may be helpful for research on spatial and temporal gene expression patterns in cultivated peanut).

In contrast, we found 38,745 genes expressed simultaneously among 22 tissues, considered common genes hereafter. The cultivated peanut has about 78,574 coding sequences (CDSs) based on the number of genes of its two ancestors, *Arachis duranensis* (36,734 genes) and *Arachis ipaënsis* (41,840 genes)^11^. Therefore, tissue-specific genes account for 4.06% of the total number of genes (3,191 out of 78,574), and commonly expressed genes account for 49.31% of the total number of genes (38,745 out of 78,574) in cultivated peanut. Further, 1,357 tissue-specific genes and 18,627 common genes were derived from *A*. *duranensis*, accounting for 1.73% (1,357 out of 78,574) and 23.71% (18,627 out of 78,574) of cultivated peanut genes. Similarly, 1,834 tissue-specific genes and 20,117 common genes were derived from *A*. *ipaënsis*, accounting for 2.32% (1,834 out of 78,574) and 25.60% (20,117 out of 78,574) of cultivated peanut genes. The tissue-specific and common genes from *A*. *ipaënsis* outnumbered those from *A*. *duranensis*. This is consistent with more gene duplication events having occurred in *A*. *ipaënsis* than in *A*. *duranensis* ^11^. Tissue-specific genes were further classified into sex-specific and somatic tissue-specific genes. In this study, sex-specific genes were expressed specifically in gynoecium and androecium tissues, while somatic tissue-specific genes were expressed specifically in one of the other 20 tissues. Sex-specific and somatic tissue-specific genes accounted for 0.66% (522 out of 78,574) and 3.40% (2,669 out of 78,574) of cultivated peanut genes. The sex-specific and somatic-specific genes from *A*. *duranensis* accounted for 0.28% (218 out of 78,574) and 1.45% (1,139 out of 78,574) of cultivated peanut genes, respectively. The sex-specific and somatic-specific genes from *A*. *ipaënsis* accounted for 0.39% (304 out of 78,574) and 1.95% (1530 out of 78,574) of cultivated peanut genes, respectively.

**Figure 2:**
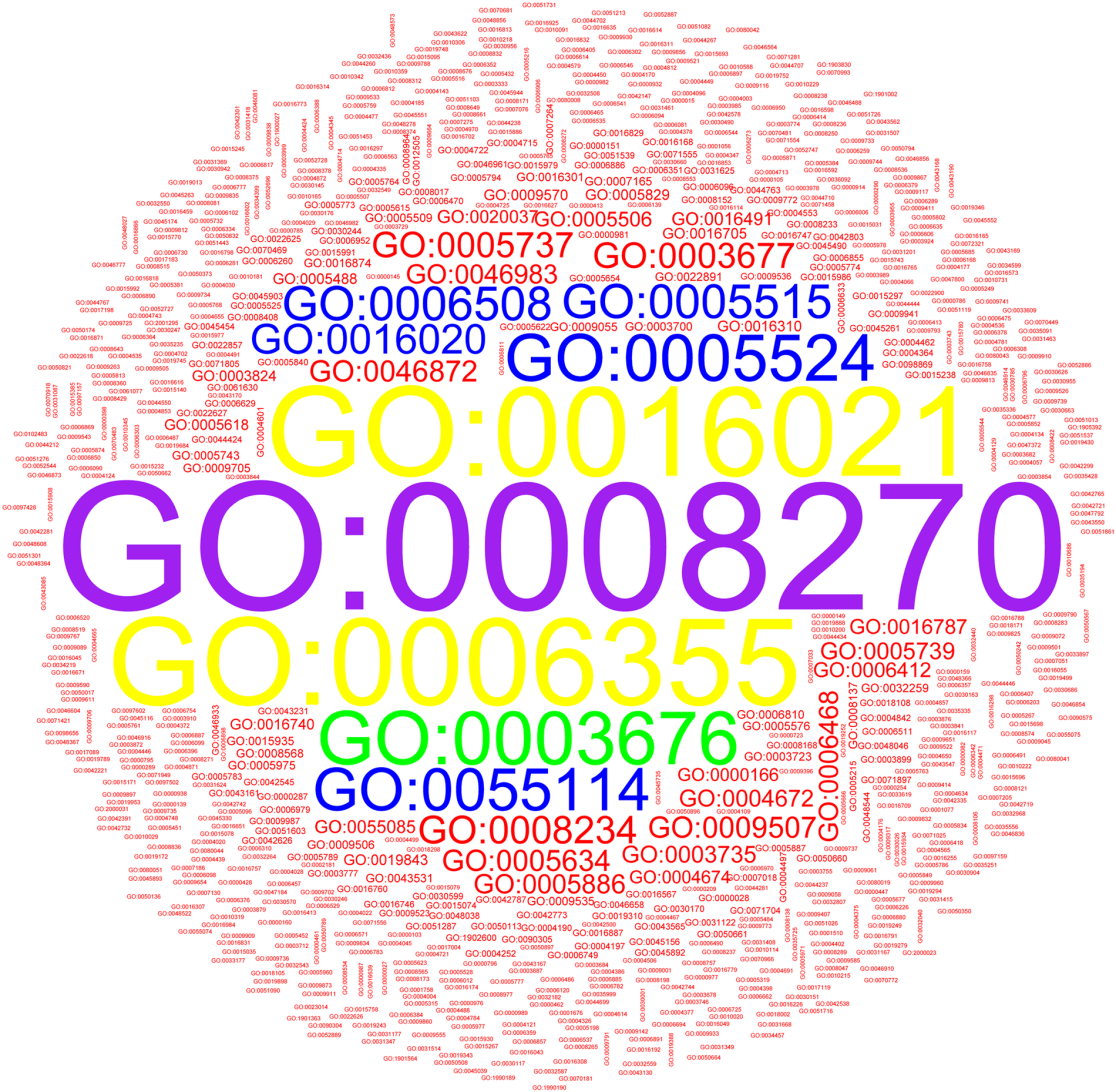
Identification of GO items in tissue-specific genes. The detailed GO annotation listed in Table S3.

Gene expression levels of tissue-specific genes were significantly lower than those of common genes (Mann–Whitney U test, *P* < 0.01). The gene expression levels differed significantly among the 22 tissues (Kruskal–Wallis test, Chi-square = 486.63, *P* < 0.05). It should also be noted that the sex-specific gene expression levels were significantly higher than those of somatic tissue-specific genes (Mann–Whitney U test, *P* < 0.01). Gynoecium-specific gene expression levels were higher than those of androecium-specific genes (Mann–Whitney U test, *P* < 0.01). Tissue-specific genes also overlapped among annotations for different tissues (Fig. S1). These analyses revealed a lack of function-specific genes among tissue-specific genes. Further, gene ontology (GO) analyses revealed that although one tissue may exhibit gene expression across different developmental stages for genes involved in different biological processes, the same biological processes may be shared among different tissues (Fig. S1). We found the most common GO categories to be 0008270 (zinc ion binding), 0006355 (regulation of transcription), 0016021 (transmembrane), 0003676 (nucleic acid binding), 0005524 (ATP binding), 0055114 (oxidation-reduction process), 0005515 (protein binding), and 006508 (proteolysis; Fig. 2). The detailed GO annotation listed in Table S3.

### Evolutionary divergence between tissue-specific duplicated genes

A total of 274 full-length duplicated gene pairs were detected from the cultivated peanut RNA-seq data. *K*_a_, *K*_s_, and *K*_a_/*K*_s_ values were calculated between 232 duplicated gene pairs, and 42 duplicated gene pairs were removed because their *K*_s_ values were less than 0.01 or larger than 0.30. The average values of *K*_a_, *K*_s_, and *K*_a_/*K*_s_ were 0.08, 0.21, and 0.56, respectively. Purifying selection predominated the molecular evolution of 207 duplicate gene pairs with *K*_a_/*K*_s_ values less than 1. In contrast, positive selection played a crucial force in 25 duplicate gene pairs with *K*_a_/*K*_s_ values larger than 1. It should be noted that these duplicate gene pairs possibly underwent adaptive evolution as suggested by their higher average *K*_a_/*K*_s_ values. Similarly, adaptive evolution was detected among sex-biased genes in *Ectocarpus* spp. because their corresponding average *K*_a_/*K*_s_ value exceeded 0.5^6, 8^.

**Figure 3:**
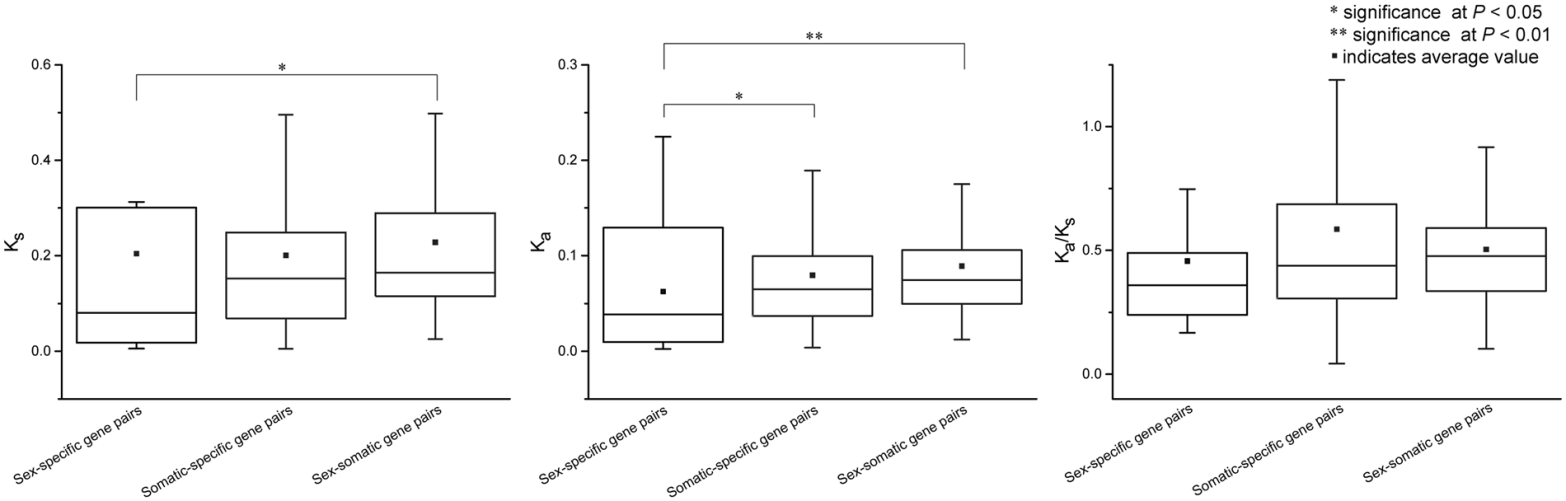
Comparison of *K*_*s*_, *K*_*a*_, and *K*_*a*_/*K*_*s*_ of duplicated gene pairs in sex-specific, somatic-specific, and somatic-sex genes.

Among duplicate gene pairs that underwent purifying selection, 167 and 40 were heterogeneous gene pairs and homogeneous gene pairs, respectively. Among duplicate gene pairs predominantly shaped by positive selection, 16 and 9 were heterogeneous gene pairs and homogeneous gene pairs, respectively. The average *K*_a_ and *K*_s_ values for homogeneous gene pairs were lower than those for heterogeneous gene pairs (Mann–Whitney U test, *P* < 0.05), indicating the heterogeneous gene pairs evolved more rapidly than homogeneous gene pairs. The average *K*_a_/*K*_s_ values of homogeneous gene pairs were larger than those of heterogeneous gene pairs, but this difference was not statistically significant (Mann-Whitney U test, *P* > 0.05). Further, 176 and 19 duplicate gene pairs consisted of somatic tissue-specific genes and sex-specific genes, respectively, while 37 duplicate gene pairs consisted of one somatic tissue-specific gene and one sex-specific gene (somatic-sex-specific gene pair). The *K*_a_ and *K*_s_ values of somatic-sex-specific duplicate genes exceeded those of both somatic tissue-specific genes and sex-specific genes (Fig. 3). This again indicates that heterogeneous gene pairs appear to evolve more rapidly than homogeneous gene pairs. In addition, the average *K*_s_ value was similar between sex-specific and somatic tissue-specific genes, but the average *K*_a_ value of somatic tissue-specific genes exceeded that of sex-specific genes (Fig. 3). The synonymous substitution rate was similar between sex-specific and somatic tissue-specific genes, while the nonsynonymous evolutionary rate of somatic tissue-specific genes was more rapid than that of sex-specific genes. However, the average *K*_a_/*K*_s_ value of somatic tissue-specific genes and somatic-sex-specific genes exceeded that of sex-specific genes, but this difference was not statistically significant (Fig. 3; Mann–Whitney U test, *P* > 0.05). Nevertheless, the average *K*_a_/*K*_s_ values of somatic tissue-specific genes, somatic-sex-specific genes, and sex-specific genes were 0.59, 0.50, and 0.46, respectively. Overall, somatic tissue-specific genes and somatic-sex-specific genes mainly underwent relaxed selection, while sex-specific genes experienced stronger selective constraint.

### Codon usage bias in tissue-specific genes

After filtering criteria were applied, a total of 2,756 sequences were used to analyze codon usage bias. Although frequency of optimal codons (Fop) was not significantly different among different tissue types (Kruskal–Wallis test, Chi-square = 22.68, *P* > 0.05), the Fop value of somatic tissue-specific genes was significantly higher than that of sex-specific genes (Mann–Whitney U test, *P* < 0.05). Moreover, Fop values of gynoecium-specific genes were slightly but not significantly higher than those of androecium-specific genes (Mann–Whitney U test, *P* > 0.05). These results indicated that codon usage bias in somatic tissue-specific genes was higher than that in sex-specific genes. In addition, amino acid sequence length was significantly different across the various tissues (Kruskal–Wallis test, Chi-square = 36.62, *P* < 0.05). The amino acid sequences of sex-specific genes were longer than those of somatic tissue-specific genes (Mann–Whitney U test, *P* < 0.05), while the amino acid sequences of gynoecium-specific genes were non-significantly longer than those of androecium-specific genes (Mann–Whitney U test, *P* > 0.05).

## Discussion

Cultivated peanut is one of the most important oil and protein crops. To elucidate the developmental processes and physiological changes involved in stress responses, many cultivated peanut RNA-seq datasets have been produced. Comparisons of genes differentially expressed between aerial gynophores and subterranean gynophores using RNA-seq data have revealed the involvement of genes that respond to hormones, the ubiquitin proteasome system, oxidative stress, and light signalling^12–14^. The cultivated peanut has been shown to have 842 potential defense-related genes involved in resistance to *Aspergillus flavus* based on RNA-seq data^15^. However, research has not been focused on patterns in the evolution and expression of tissue-specific genes in peanut, especially comparisons between sex-specific and somatic tissue-specific genes. The genomes of the cultivated peanut progenitor species, *A*. *duranensis* and *A*. *ipaënsis*, have recently been completed^11^. Additionally, 22 RNA-seq datasets distributed across different tissues of cultivated peanut have been assembled based on the *A*. *duranensis* and *A*. *ipaënsis* genomes^10^. We used these resources to identify tissue-specific genes and then analyzed the evolutionary pattern and gene expression level of these genes. There were five key findings. (1) The expression levels of sex-specific genes were significantly higher than those of somatic tissue-specific genes. Among sex-specific genes, the expression levels of gynoecium-specific genes were significantly higher than those of androecium-specific genes. (2) The functions of tissue-specific genes overlapped among the different tissues. (3) Heterogeneous gene pairs evolved more rapidly than homogeneous gene pairs. (4) The evolutionary rate of somatic tissue-specific genes exceeded that of sex-specific genes. Molecular signatures of selective pressure indicated that somatic tissue-specific genes mainly experienced relaxed selection, while sex-specific genes were subject to stronger selective constraint. (5) Somatic tissue-specific genes had higher codon usage bias than sex-specific genes.

There are at less two possible explanations for the difference in gene expression level among tissue-specific genes. First, these results are supported by the gene expression and rate of sequence evolution anticorrelation model (E-R anticorrelation model)^16^. Under this model, the most highly expressed genes are also subject to the strongest selective constraint^17^. In this study, sex-specific genes experienced stronger selective constraint than somatic tissue-specific genes, and the expression levels of sex-specific genes were higher than those of somatic tissue-specific genes. However, we cannot use E-R anticorrelation to explain the different gene expression levels exhibited between gynoecium-specific genes and androecium-specific genes because we were unable to estimate the selective pressures on androecium-specific genes (there was only one duplicated gene pair). Nevertheless, previous studies have demonstrated that more selective constraint has acted on male-specific genes relative to female-specific genes^3^. If the same pattern is found in cultivated peanut, the E-R anticorrelation model may explain why the significant difference in expression level between gynoecium-specific genes and androecium-specific genes. Second, different tissue-specific genes had different ages. Gossmann, *et al*.^3^ found that female genes were younger than male genes and that the gene expression levels of female genes were higher than those of male genes in *A*. *thaliana*. A significant shift in gene expression occurs during gametogenesis in *A thaliana*, *Oryza sativa*, and *Glycine max*, in which younger genes than those typically expressed in sporophytes^18^. We therefore suggest that the sex-specific genes may be younger than somatic tissue-specific genes.

The biological function of various tissue-specific genes lacked function-specificity. This contrasts with a previous study focused on tissue-biased genes. Shi, *et al*.^19^ found that root-biased, leaf-biased, and pistil-biased genes mainly participated in stress responses, photosynthesis, and meiotic cell cycles in barley. Though we cannot explain the non-specificity of the functions of tissue-specific genes, we suggest that the origin of tissue-specific genes precedes those of other genes in evolutionary time. Thus, tissue-specific genes have had longer to diverge in function. Accordingly, newer genes were likely progeny of tissue-specific genes. However, there is a lack of research on the relationship between tissue-specific and constitutively expressed genes in cultivated peanut.

Male-biased genes have evolved more rapidly than female-biased genes in many species^1, 2^. However, little is known about the difference between the evolutionary rates of somatic tissue-specific and sex-specific genes. A single published study about barley has demonstrated that pistil-biased orthologous pairs evolve more quickly than stem-, leaf-, and root-biased genes^19^. However, these results were inconsistent with our finding that somatic tissue-specific genes evolved more rapidly than sex-specific genes. This discrepancy may be explained by our use of tissue-specific genes, while this previous study used tissue-biased genes. The evolutionary rates and gene expression levels of tissue-specific and tissue-biased genes may of course differ. We have demonstrated that the expression levels of tissue-specific genes were lower than those of common genes in this study. Recently, Cheng and Kirkpatrick^20^ demonstrated that sex-biased expression is consistent with ongoing sex-specific selection in humans and files. Similarly, the evolutionary patterns and expression levels of these genes may have recently changed. In addition, amino acid sequence length has likely affected the evolutionary rate. Previous studies have shown that amino acid sequence length was negatively correlated with evolutionary rate^21, 22^. In the present study, amino acid sequences of sex-specific genes were longer than those of somatic tissue-specific genes.

Overall, somatic tissue-specific genes exhibited higher codon usage bias than sex-specific genes. We found that natural selection acted on codon usage bias of orthologous pairs between *A*. *duranensis* and *A*. *ipaënsis* (unpublished data). These previous results indicate that somatic tissue-specific genes were more highly expressed than sex-specific genes. However, sex-specific genes were expressed at a higher level than somatic-specific genes in the present study. This discrepancy in conclusions between the two studies may be explained by differences in the abundances of tRNAs in different tissues^23^. Thus, selection on codon bias may differ based on how tRNA abundance affects the efficiency and accuracy of translation processes in various tissues and cell types^24^.

## Materials and Methods

### Sequence data and gene ontology

The data used in the present study include *A*. *duranensis* and *A*. *ipaënsis* genomic CDSs and RNA-seq data from various tissues in cultivated peanut, which are available from PeanutBase (http://peanutbase.org/download)^10, 11^. The 22 RNA-seq datasets were generated from leaf, shoot, peg, fruit, pericarp, seed, stamen, and pistil tissues. All details of the sequencing, de novo transcriptome assembly, and generation of expression values were previously described by Clevenger, *et al*.^10^. Briefly, first, cultivated peanuts (‘Tifrunner’) were grown in a greenhouse (maintained at 24–30 °C). All tissues were harvested at 14:00 except for flower samples, which were collected at 8:30. Three biological replicates of each tissue were sampled from three different plants. The total RNA of each pooled tissue sample was extracted. TruSeq RNA Sample Preparation v2 kits were used for library construction and paired-end 2 × 100 bp sequencing was conducted using an Illumina HiSeq. 2500 instrument with a total of 209 cycles of TruSeq Rapid SBS Kit v1 (Illumina, San Diego, CA, USA) chemistry. Second, an *in silico* amphidiploid genome was created by simply disregarding scaffolds and concatenating the *A*. *duranensis* genome assembly with the *A*. *ipaënsis* genome assembly and labeling each simply the “A” and “B” genomes, respectively. Once the reads were mapped, the SAM file was run through the genome-guided pipeline. Third, total reads were mapped to the transcript assembly from 58 libraries (consisting of samples from 22 distinct tissue types and developmental stages including vegetative and seed stages) using Bowtie, allowing 2 mismatches within a particular 25 bp seed. Fragments per kilobase per million reads mapped (FPKM) were estimated using RSEM^25^ for each library. When reads map to multiple transcripts, RSEM fractionates the read count among the transcripts so read counts are not integers. Transcripts that had less than 1 FPKM for all 58 libraries were filtered out using the Trinity package, because they were deemed to lack sufficient minimum read coverage. The FPKM values for each gene were distinguished for both the A (*A*. *duranensis*) and B (*A*. *ipaënsis*) genomes from cultivated peanut. In this study, the log_2_-transformed FPKM values were considered as normalised gene expression levels in these various tissues. The tissue-specific gene was considered as a gene with a FPKM value in a given tissue but not found in other tissues. Common genes were considered to be genes expressed in all 22 tissues.

GO analysis was conducted using Blast2Go^26^, and the presented figures describing this GO analysis were conducted in WEGO^27^ and using R scripts.

### Measurement of synonymous and nonsynonymous substitution rates

We used CDSs as queries to themselves in a comparison using local BLAST. The following evaluation criteria were used as thresholds to identify gene duplicates^10^: (1) length of aligned sequences > 80% of the longer sequence’s length, (2) identity > 80%, and (3) E-value ≤ 10^−10^. To avoid matches to partial sequences, all query sequences contained full-length coding frames. If a duplicate gene pair was expressed in a single tissue, it was classified as a homogeneous gene pair. In turn, duplicate gene pairs expressed only in different tissues were labeled heterogeneous gene pairs.

MAFFT^28^ was then used to obtain alignments of duplicate gene pairs. PAL2NAL^29^ was used for conversion of protein sequences into their corresponding nucleotide sequences. PAML 4.0^30^ was used to calculate the *K*_a_/*K*_s_, ratio of nonsynonymous to synonymous per site substitution rates. The duplicate gene pairs for which *K*_s_ values were less than 0.01 and greater than 0.30 were excluded because low sequence divergence can result in unreliable estimates and high *K*_s_ values indicate potential sequence saturation, respectively^30^. Generally, *K*_a_/*K*_s_ equal to 1, greater than 1, and less than 1 indicated neutral, positive, and purifying selection, respectively.

### Codon usage bias and amino acid length

Codon usage bias, measured as the frequency of optimal codons (Fop) and amino acid length were estimated by Codon W (version 1.4, http://codonw.sourceforge.net). Fop values can range from 0 to 1. Fop values approaching 1 indicate a given gene has extreme codon usage bias^31^. To avoid partial sequences, the following evaluation criteria were adopted: (1) CDSs must start in ATG and end in TAA, TAG, or TGA and (2) CDSs must lack premature termination or ambiguous codons.

### Statistical analysis

The Mann–Whitney U and Kruskal–Wallis tests were conducted in JMP 9.0 (SAS Institute, Inc., Cary, NC, USA). *P*-values less than 0.05 were considered significant.

## Electronic supplementary material

Supplementary materials

## References

[1] Grath S, Parsch J (2016). Sex-biased gene expression. Annu Rev Genet.

[2] Ellegren H, Parsch J (2007). The evolution of sex-biased genes and sex-biased gene expression. Nat Rev Genet.

[3] Gossmann TI, Schmid MW, Grossniklaus U, Schmid KJ (2013). Selection-driven evolution of sex-biased genes is consistent with sexual selection in *Arabidopsis thaliana*. Mol Bio Evol.

[4] Arunkumar R, Josephs EB, Williamson RJ, Wright SI (2013). Pollen-specific, but not sperm-specific, genes show stronger purifying selection and higher rates of positive selection than sporophytic genes in *Capsella grandiflora*. Mol Bio Evol.

[5] Hersh E (2015). Sexual antagonism in the pistil varies among populations of a hermaphroditic mixed-mating plant. J Evolution Biol.

[6] Lipinska A (2015). Sexual dimorphism and the evolution of sex-biased gene expression in the brown alga. Ectocarpus. Mol Bio Evol.

[7] Zemp N (2016). Evolution of sex-biased gene expression in a dioecious plant. Nature Plants.

[8] Lipinska AP, Damme EJMV, De Clerck O (2016). Molecular evolution of candidate male reproductive genes in the brown algal model Ectocarpus. BMC Evol Biol.

[9] Whittle CA, Malik MR, Krochko JE (2007). Gender-specific selection on codon usage in plant genomes. BMC Genomics.

[10] Clevenger J, Chu Y, Scheffler B, Ozias-Akins P (2016). A developmental transcriptome map for allotetraploid *Arachis hypogaea*. Front Plant Sci.

[11] Bertioli DJ (2016). The genome sequences of *Arachis duranensis* and *Arachis ipaensis*, the diploid ancestors of cultivated peanut. Nat Genet.

[12] Zhang Y (2016). Comparative transcriptome analysis of basal and zygote-located tip regions of peanut ovaries provides insight into the mechanism of light regulation in peanut embryo and pod development. BMC Genomics.

[13] Zhu W (2013). Comparative proteomics analysis of developing peanut aerial and subterranean pods identifies pod swelling related proteins. J Proteomics.

[14] Zhu W (2014). Comparative transcriptome analysis of aerial and subterranean pods development provides insights into seed abortion in peanut. Plant Mol Biol.

[15] Wang H (2016). Comparative transcript profiling of resistant and susceptible peanut post-harvest seeds in response to aflatoxin production by *Aspergillus flavus*. BMC Plant Biol.

[16] Drummond DA, Bloom JD, Adami C, Wilke CO, Arnold FH (2005). Why highly expressed proteins evolve slowly. Proc Natl Acad Sci USA.

[17] Geiler-Samerotte KA (2011). Misfolded proteins impose a dosage-dependent fitness cost and trigger a cytosolic unfolded protein response in yeast. Proc Natl Acad Sci USA.

[18] Gossmann TI, Saleh D, Schmid MW, Spence MA, Schmid KJ (2016). Transcriptomes of plant gametophytes have a higher proportion of rapidly evolving and young genes than sporophytes. Mol Bio Evol.

[19] Shi T (2015). Accelerated rates of protein evolution in barley grain and pistil biased genes mignt be legacy of domestication. Plant Mol Biol.

[20] Cheng C, Kirkpatrick M (2016). Sex-specific selection and sex-biased gene expression in humans and flies. PLoS Genet.

[21] Mugal CF, Wolf JBW, Kaj I (2014). Why time matters: codon evolution and the temporal dynamics of dN/dS. Mol Bio Evol.

[22] Yang L, Gaut BS (2011). Factors that contribute to variation in evolutionary rate among *Arabidopsis* genes. Mol Bio Evol.

[23] Camiolo S, Farina L, Porceddu A (2012). The relation of codon bias to tissue-specific gene expression in *Arabidopsis thaliana*. Genetics.

[24] Novoa EM, de Pouplana LR (2012). Speeding with control: codon usage, tRNAs, and ribosomes. Trends Genet.

[25] Li B, Dewey CN (2011). RSEM: accurate transcript quantification from RNA-Seq data with or without a reference genome. BMC Bioinformatics.

[26] Conesa A (2005). Blast2GO: a universal tool for annotation, visualization and analysis in functional genomics research. Bioinformatics.

[27] Ye J (2006). WEGO: a web tool for plotting GO annotations. Nucleic Acids Res.

[28] Katoh K, Standley DM (2013). MAFFT multiple sequence alignment software version 7: improvements in performance and usability. Mol Bio Evol.

[29] Suyama M, Torrents D, Bork P (2006). PAL2NAL: robust conversion of protein sequence alignments into the corresponding codon alignments. Nucleic Acids Res.

[30] Yang Z (2007). PAML 4: phylogenetic analysis by maximum likelihood. Mol Bio Evol.

[31] Sharp PM, Li WH (1987). The codon adaption index-a measure of directional synonymous codon usage bias, and its potential applications. Nucleic Acids Res.

