# Supplementary material for: Differential evolutionary patterns and expression levels between sex-specific and somatic tissue-specific genes in peanut
Source: Sci Rep. 2017 Aug 21;7:9016. doi: 10.1038/s41598-017-09905-8 (PMC5566475; doi:10.1038/s41598-017-09905-8)
Supplement: Supplementary file 1 — Supplementary materials [file 41598_2017_9905_MOESM1_ESM.doc]

Differential evolutionary patterns and expression levels between sex-specific and somatic tissue-specific genes in peanut

Hui Song*****, Qingping Zhang, Pei Tian, Zhibiao Nan*****

State Key Laboratory of Grassland Agro-ecosystems, College of Pastoral Agriculture Science and Technology, Lanzhou University, Lanzhou, 730000, China

*****Correspondence: biosonghui@outlook.com; zhibiao@lzu.edu.cn


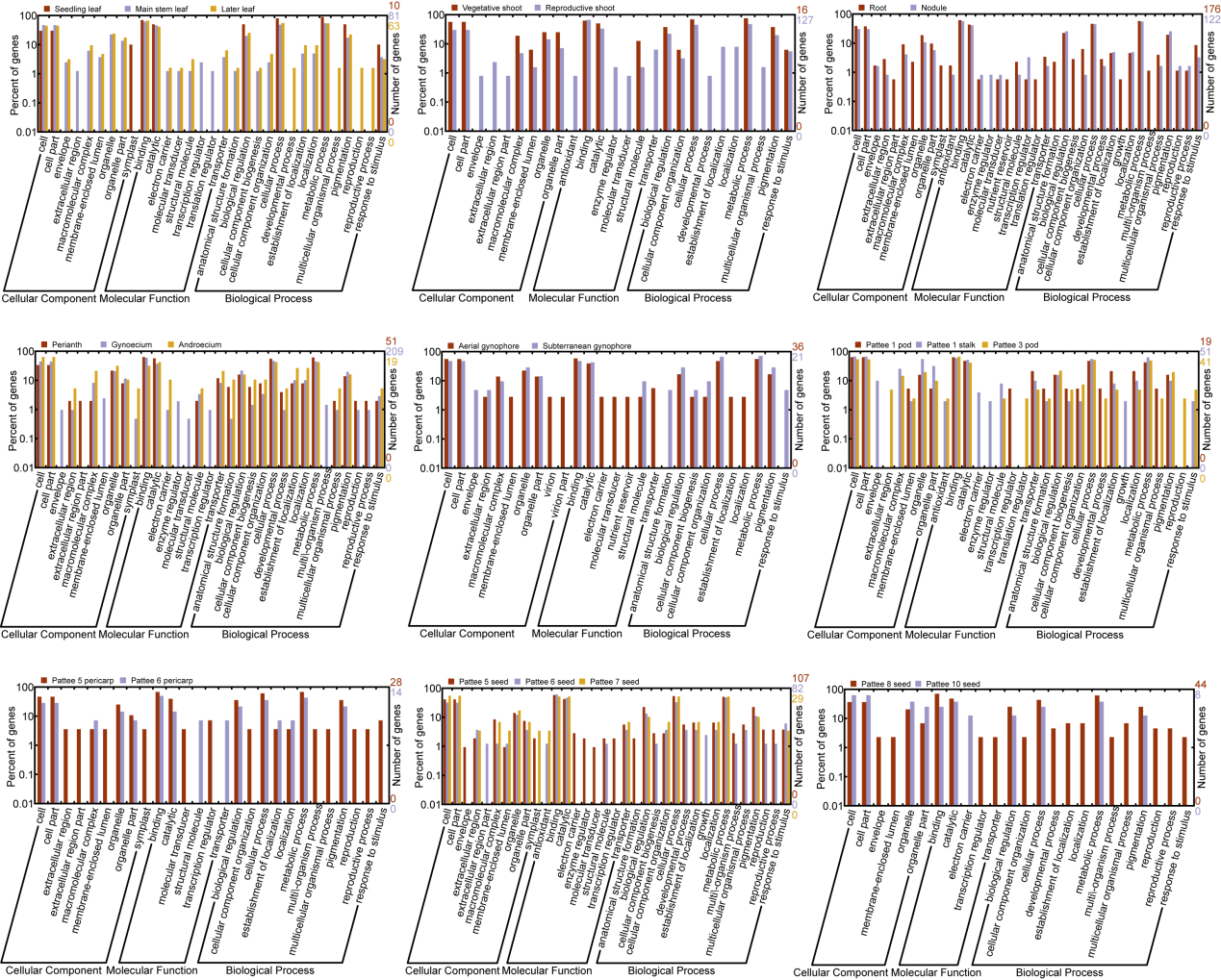


Figure S1 GO items in different tissue-specific genes

Table S1 Expression level and annotation of 22 different tissue-specific genes in cultivated peanut

| Gene name | Expression level^a^ | Annotation |
| --- | --- | --- |
| Seedling leaf | |  |
| Aradu.14KKV | 0.03 | receptor-like protein kinase 2 |
| Aradu.2DG56 | 0.07 | FAR1 DNA-binding domain protein |
| Aradu.2I4K6 | 0.03 | replication factor-A carboxy-terminal domain protein; IPR012340 (Nucleic acid-binding, OB-fold) |
| Aradu.FK7VW | 0.02 | MBOAT (membrane bound O-acyl transferase) family protein; IPR004299 (Membrane bound O-acyl transferase, MBOAT) |
| Aradu.GE72G | 0.02 | Transposon protein, putative, unclassified n=2 Tax=Oryza sativa subsp. japonica RepID=Q2QZM0_ORYSJ |
| Aradu.GS1LY | 0.04 | replication factor-A carboxy-terminal domain protein; IPR012340 (Nucleic acid-binding, OB-fold) |
| Aradu.IDC5G | 0.02 | E3 ubiquitin-protein ligase n=5 Tax=Solanum RepID=M1BDZ1_SOLTU |
| Aradu.KW28B | 0.04 | protein FAR1-RELATED SEQUENCE 6-like isoform X2 [Glycine max]; IPR004330 (FAR1 DNA binding domain) |
| Aradu.KZ4GJ | 0.02 | Unknown protein |
| Aradu.L167J | 0.17 | WAPL (Wings apart-like protein regulation of heterochromatin) protein |
| Aradu.LT6MY | 0.02 | replication protein A 1A; IPR012340 (Nucleic acid-binding, OB-fold) |
| Aradu.QB41L | 0.1 | Unknown protein |
| Aradu.W2C2I | 0.07 | Unknown protein |
| Araip.1G02P | 0.01 | E3 ubiquitin-protein ligase n=5 Tax=Solanum RepID=M1BDZ1_SOLTU |
| Araip.1L9VG | 0.03 | Unknown protein; IPR003653 (Peptidase C48, SUMO/Sentrin/Ubl1); GO:0006508 (proteolysis), GO:0008234 (cysteine-type peptidase activity) |
| Araip.24RU2 | 0.16 | Unknown protein |
| Araip.3UD9L | 0.11 | Unknown protein; IPR010666 (Zinc finger, GRF-type); GO:0008270 (zinc ion binding) |
| Araip.765V5 | 0.04 | E3 ubiquitin-protein ligase n=5 Tax=Solanum RepID=M1BDZ1_SOLTU |
| Araip.EFA16 | 0.02 | protein FAR1-RELATED SEQUENCE 6-like isoform X2 [Glycine max]; IPR004330 (FAR1 DNA binding domain), IPR007527 (Zinc finger, SWIM-type); GO:0008270 (zinc ion binding) |
| Araip.FW38D | 0.07 | FAR-RED impaired response-like protein; IPR004330 (FAR1 DNA binding domain) |
| Araip.H0F0E | 0.09 | Unknown protein |
| Araip.KGG54 | 0.11 | unknown protein |
| Araip.KR83D | 0.12 | homeobox protein knotted-1-like 2-like [Glycine max] |
| Araip.LMI31 | 0.04 | Unknown protein |
| Araip.SC09P | 0.02 | uncharacterized protein LOC102664163 isoform X7 [Glycine max]; IPR004252 (Probable transposase, Ptta/En/Spm, plant) |
| Araip.UP7DF | 0.1 | C3HC4-type RING zinc finger protein |
| Araip.W9UVZ | 0.22 | cellulose synthase like E1; IPR005150 (Cellulose synthase); GO:0016020 (membrane), GO:0016760 (cellulose synthase (UDP-forming) activity), GO:0030244 (cellulose biosynthetic process) |
| Araip.Y894W | 0.09 | receptor kinase 3; IPR011009 (Protein kinase-like domain); GO:0004672 (protein kinase activity), GO:0005524 (ATP binding), GO:0006468 (protein phosphorylation) |
| Main sterm leaf | |  |
| Aradu.1R8GF | 0.02 | DNA-directed RNA polymerases II, IV and V subunit 12 [Glycine max]; IPR006591 (RNA polymerase archaeal subunit P/eukaryotic subunit RPABC4); GO:0003677 (DNA binding), GO:0003899 (DNA-directed RNA polymerase activity) |
| Aradu.1TL24 | 0.04 | hypothetical protein; IPR001878 (Zinc finger, CCHC-type); GO:0003676 (nucleic acid binding), GO:0008270 (zinc ion binding) |
| Aradu.1WK0W | 0.05 | cellulose synthase family protein; IPR005150 (Cellulose synthase), IPR026961 (PGG domain); GO:0016020 (membrane), GO:0016760 (cellulose synthase (UDP-forming) activity), GO:0030244 (cellulose biosynthetic process) |
| Aradu.1ZD47 | 0.02 | Unknown protein |
| Aradu.21JML | 0.01 | Unknown protein |
| Aradu.29AS3 | 0.01 | Dehydrogenase/reductase SDR family member n=3 Tax=Papilionoideae RepID=G7JKG6_MEDTR; IPR002347 (Glucose/ribitol dehydrogenase) |
| Aradu.2TW3V | 0.03 | LRR receptor-like serine/threonine-protein kinase GSO1-like [Glycine max] |
| Aradu.32CZH | 0.02 | flocculation protein FLO11-like isoform X3 [Glycine max]; IPR004252 (Probable transposase, Ptta/En/Spm, plant) |
| Aradu.3LJ8U | 0.01 | E3 ubiquitin-protein ligase n=5 Tax=Solanum RepID=M1BDZ1_SOLTU |
| Aradu.43JEJ | 0.01 | serine/threonine-protein phosphatase 7 long form homolog [Glycine max]; IPR019557 (Aminotransferase-like, plant mobile domain) |
| Aradu.4L9IW | 0.19 | Unknown protein |
| Aradu.59WCK | 0.02 | Unknown protein |
| Aradu.5VY9W | 0.02 | receptor-like protein kinase 1; IPR011009 (Protein kinase-like domain); GO:0004672 (protein kinase activity), GO:0004713 (protein tyrosine kinase activity), GO:0005524 (ATP binding), GO:0006468 (protein phosphorylation) |
| Aradu.61DK7 | 0.03 | RNAase n=1 Tax=Streptococcus thermophilus M17PTZA496 RepID=W4KSI5_STRTR |
| Aradu.6AJ68 | 0.02 | transcription factor SPATULA-like isoform X1 [Glycine max]; IPR011598 (Myc-type, basic helix-loop-helix (bHLH) domain); GO:0046983 (protein dimerization activity) |
| Aradu.6C1F9 | 0.01 | receptor-like protein kinase 2; IPR001611 (Leucine-rich repeat), IPR003591 (Leucine-rich repeat, typical subtype); GO:0005515 (protein binding) |
| Aradu.6I571 | 0.05 | photosystem II CP43 chlorophyll apoprotein; IPR000932 (Photosystem antenna protein-like); GO:0009521 (photosystem), GO:0009767 (photosynthetic electron transport chain), GO:0016020 (membrane), GO:0016168 (chlorophyll binding) |
| Aradu.6S64P | 0.01 | Unknown protein |
| Aradu.72WNN | 0.04 | Cytochrome P450 superfamily protein; IPR001128 (Cytochrome P450); GO:0005506 (iron ion binding), GO:0020037 (heme binding), GO:0055114 (oxidation-reduction process) |
| Aradu.931XS | 0.03 | Unknown protein |
| Aradu.9G4XA | 0.03 | phosphoinositide phosphatase SAC2-like isoform X3 [Glycine max] |
| Aradu.9XT0N | 0.01 | phosphoglucomutase; IPR005841 (Alpha-D-phosphohexomutase superfamily); GO:0005975 (carbohydrate metabolic process) |
| Aradu.9Z3FS | 0.01 | Sec14p-like phosphatidylinositol transfer family protein |
| Aradu.A4X5A | 0.01 | LRR and NB-ARC domain disease resistance protein |
| Aradu.ARU8D | 0.01 | Disease resistance protein (TIR-NBS-LRR class) family; IPR000767 (Disease resistance protein), IPR027417 (P-loop containing nucleoside triphosphate hydrolase); GO:0006952 (defense response), GO:0043531 (ADP binding) |
| Aradu.B2EZC | 0.01 | Ulp1 protease family, carboxy-terminal domain protein |
| Aradu.B4LFF | 0.02 | Unknown protein |
| Aradu.BM5PJ | 0.02 | Unknown protein |
| Aradu.CY1V6 | 0.01 | uncharacterized protein LOC100797259 isoform X4 [Glycine max]; IPR001878 (Zinc finger, CCHC-type), IPR004332 (Transposase, MuDR, plant), IPR007527 (Zinc finger, SWIM-type); GO:0003676 (nucleic acid binding), GO:0008270 (zinc ion binding) |
| Aradu.FH8T1 | 0.01 | zinc finger MYM-type protein 1-like [Glycine max]; IPR008906 (HAT dimerisation domain, C-terminal), IPR012337 (Ribonuclease H-like domain), IPR025398 (Domain of unknown function DUF4371); GO:0003676 (nucleic acid binding), GO:0046983 (protein dimerization activity) |
| Aradu.FTQ92 | 0.02 | signal peptide peptidase |
| Aradu.GJG4Z | 0.01 | serine/threonine-protein phosphatase 7 long form homolog [Glycine max]; IPR019557 (Aminotransferase-like, plant mobile domain) |
| Aradu.GQ9ZJ | 0.04 | novel plant snare 12; IPR000727 (Target SNARE coiled-coil domain), IPR005606 (Sec20); GO:0005515 (protein binding) |
| Aradu.H2IT6 | 0.01 | uncharacterized protein At2g29880-like [Glycine max] |
| Aradu.H33XQ | 0.01 | FAR1-related sequence 11; IPR004330 (FAR1 DNA binding domain) |
| Aradu.H6Y5V | 0.02 | Unknown protein |
| Aradu.H8JMX | 0.01 | short-chain dehydrogenase/reductase; IPR002347 (Glucose/ribitol dehydrogenase) |
| Aradu.HUS6A | 0.02 | aldehyde dehydrogenase family 3 member F1 [Glycine max]; IPR012394 (Aldehyde dehydrogenase NAD(P)-dependent), IPR016161 (Aldehyde/histidinol dehydrogenase); GO:0004030 (aldehyde dehydrogenase [NAD(P)+] activity), GO:0006081 (cellular aldehyde metabolic process), GO:0008152 (metabolic process), GO:0016491 (oxidoreductase activity), GO:0055114 (oxidation-reduction process) |
| Aradu.I4LWX | 0.02 | E3 ubiquitin-protein ligase n=2 Tax=Vitis vinifera RepID=F6HSM1_VITVI |
| Aradu.J93DN | 0.01 | Unknown protein |
| Aradu.JVR5P | 0.02 | Unknown protein |
| Aradu.LV0E1 | 0.01 | Unknown protein |
| Aradu.M9FCM | 0.01 | serine/threonine-protein phosphatase 7 long form homolog [Glycine max]; IPR019557 (Aminotransferase-like, plant mobile domain) |
| Aradu.MT7M6 | 0.02 | Unknown protein |
| Aradu.N2SI1 | 0.01 | Unknown protein |
| Aradu.N327A | 0.02 | Unknown protein |
| Aradu.N7T68 | 0.01 | Helicase-like protein n=1 Tax=Oryza sativa subsp. japonica RepID=Q9AYF0_ORYSJ; IPR025476 (Helitron helicase-like domain) |
| Aradu.NAY5Z | 0.01 | nucleolin 1-like [Glycine max] |
| Aradu.NU4UJ | 0.01 | MYB transcription factor MYB51 [Glycine max]; IPR009057 (Homeodomain-like); GO:0003677 (DNA binding), GO:0003682 (chromatin binding) |
| Aradu.P1X6T | 0.08 | Unknown protein |
| Aradu.P7I9W | 0.01 | hypothetical protein |
| Aradu.PAW9R | 0.01 | Helicase-like protein n=1 Tax=Medicago truncatula RepID=G7JN81_MEDTR; IPR025476 (Helitron helicase-like domain) |
| Aradu.PDT3I | 0.02 | Unknown protein |
| Aradu.Q4JCR | 0.05 | Octicosapeptide/Phox/Bem1p family protein; IPR000270 (Phox/Bem1p); GO:0005515 (protein binding) |
| Aradu.RB0NE | 0.01 | Pectate lyase family protein; IPR011050 (Pectin lyase fold/virulence factor), IPR018082 (AmbAllergen) |
| Aradu.RLD9T | 0.02 | Myb/SANT-like DNA-binding domain protein |
| Aradu.S6CYD | 0.01 | Pentatricopeptide repeat (PPR-like) superfamily protein; IPR002885 (Pentatricopeptide repeat) |
| Aradu.SG3MC | 0.01 | zinc finger MYM-type protein 1-like [Glycine max]; IPR008906 (HAT dimerisation domain, C-terminal), IPR012337 (Ribonuclease H-like domain); GO:0003676 (nucleic acid binding), GO:0046983 (protein dimerization activity) |
| Aradu.SNG7P | 0.05 | beta-galactosidase 15; IPR001944 (Glycoside hydrolase, family 35); GO:0005975 (carbohydrate metabolic process) |
| Aradu.T0V7T | 0.01 | ribulose bisphosphate carboxylase/oxygenase activase |
| Aradu.T8LKA | 0.01 | Ankyrin repeat family protein; IPR020683 (Ankyrin repeat-containing domain), IPR026961 (PGG domain) |
| Aradu.TA9IB | 0.02 | high chlorophyll fluorescence phenotype 173 protein |
| Aradu.TAW1W | 0.01 | Unknown protein; IPR003653 (Peptidase C48, SUMO/Sentrin/Ubl1); GO:0006508 (proteolysis), GO:0008234 (cysteine-type peptidase activity) |
| Aradu.TB70V | 0.02 | protein n=1 Tax=Oryza sativa subsp. japonica RepID=C7J0D1_ORYSJ; IPR001878 (Zinc finger, CCHC-type), IPR007527 (Zinc finger, SWIM-type); GO:0003676 (nucleic acid binding), GO:0008270 (zinc ion binding) |
| Aradu.TP64I | 0.03 | Sentrin-specific protease n=1 Tax=Morus notabilis RepID=W9QIH0_9ROSA |
| Aradu.UG02F | 0.01 | pentatricopeptide repeat-containing protein At3g49730-like isoform X3 [Glycine max] |
| Aradu.UUL58 | 0.06 | Unknown protein |
| Aradu.V09FZ | 0.01 | Unknown protein |
| Aradu.W2UL6 | 0.08 | transportin-1-like isoform X2 [Glycine max] |
| Aradu.XQ3M6 | 0.03 | Unknown protein |
| Aradu.XSE8F | 0.01 | Unknown protein |
| Aradu.YV5Z0 | 0.05 | serine/threonine-protein phosphatase 7 long form homolog [Glycine max]; IPR019557 (Aminotransferase-like, plant mobile domain) |
| Aradu.Z0DDM | 0.02 | Unknown protein |
| Aradu.Z9R7V | 0.04 | Unknown protein |
| Aradu.ZQS8V | 0.02 | Unknown protein |
| Aradu.ZU2WC | 0.01 | Unknown protein |
| Araip.054KN | 0.02 | Unknown protein |
| Araip.05GGX | 0.01 | zinc finger MYM-type protein 1-like [Glycine max]; IPR006580 (Zinc finger, TTF-type), IPR008906 (HAT dimerisation domain, C-terminal), IPR012337 (Ribonuclease H-like domain), IPR025398 (Domain of unknown function DUF4371); GO:0003676 (nucleic acid binding), GO:0046983 (protein dimerization activity) |
| Araip.0A0S5 | 0.02 | Unknown protein |
| Araip.0I9CD | 0.01 | Stress induced protein; IPR000389 (Stress induced protein) |
| Araip.1H2FT | 0.01 | Unknown protein |
| Araip.1R9HQ | 0.02 | plasma membrane H+-ATPase; IPR001757 (Cation-transporting P-type ATPase), IPR023214 (HAD-like domain); GO:0006812 (cation transport), GO:0016021 (integral component of membrane), GO:0019829 (cation-transporting ATPase activity) |
| Araip.1T8ET | 0.06 | Unknown protein |
| Araip.2BB1N | 0.01 | protein FAR1-RELATED SEQUENCE 6-like isoform X2 [Glycine max]; IPR004330 (FAR1 DNA binding domain) |
| Araip.2MB05 | 0.03 | FKBP-like peptidyl-prolyl cis-trans isomerase family protein |
| Araip.2Y2KG | 0.04 | Unknown protein |
| Araip.34IDA | 0.03 | serine/threonine-protein phosphatase 7 long form homolog [Glycine max]; IPR019557 (Aminotransferase-like, plant mobile domain) |
| Araip.3GG4Y | 0.01 | protein FAR1-RELATED SEQUENCE 6-like isoform X2 [Glycine max]; IPR004330 (FAR1 DNA binding domain), IPR006564 (Zinc finger, PMZ-type); GO:0008270 (zinc ion binding) |
| Araip.3GQ4D | 0.01 | Unknown protein |
| Araip.3HR93 | 0.02 | Unknown protein |
| Araip.3Q92Y | 0.01 | NAD-dependent epimerase/dehydratase family protein; IPR016040 (NAD(P)-binding domain) |
| Araip.3Y03H | 0.01 | serine/threonine-protein phosphatase 7 long form homolog [Glycine max]; IPR019557 (Aminotransferase-like, plant mobile domain) |
| Araip.44G06 | 0.02 | uncharacterized protein LOC100779146 [Glycine max] |
| Araip.45QUC | 0.02 | Unknown protein |
| Araip.4JA2X | 0.01 | serine/threonine-protein phosphatase 7 long form homolog [Glycine max]; IPR019557 (Aminotransferase-like, plant mobile domain) |
| Araip.551UD | 0.07 | nudix hydrolase homolog 14 |
| Araip.5BQ4Y | 0.03 | protein n=1 Tax=Oryza sativa subsp. japonica RepID=C7J340_ORYSJ |
| Araip.62H8F | 0.01 | probable acetyl-CoA acetyltransferase, cytosolic 2 isoform X3 [Glycine max]; IPR002155 (Thiolase), IPR016039 (Thiolase-like); GO:0003824 (catalytic activity), GO:0008152 (metabolic process) |
| Araip.67EC7 | 0.01 | serine/threonine-protein phosphatase 7 long form homolog [Glycine max]; IPR019557 (Aminotransferase-like, plant mobile domain) |
| Araip.6VG98 | 0.02 | paired amphipathic helix protein Sin3-like 4-like isoform X4 [Glycine max]; IPR003822 (Paired amphipathic helix), IPR018247 (EF-Hand 1, calcium-binding site); GO:0005634 (nucleus) |
| Araip.7552N | 0.02 | protein n=1 Tax=Oryza sativa subsp. japonica RepID=C7J9W8_ORYSJ; IPR004332 (Transposase, MuDR, plant) |
| Araip.76A0M | 0.03 | ribulose bisphosphate carboxylase large chain |
| Araip.7IW9D | 0.02 | arginine/serine-rich coiled coil protein |
| Araip.7RL7S | 0.01 | Unknown protein |
| Araip.7XJ42 | 0.01 | uncharacterized protein LOC102662047 [Glycine max]; IPR025312 (Domain of unknown function DUF4216) |
| Araip.82PUV | 0.04 | GRF zinc finger protein; IPR010666 (Zinc finger, GRF-type); GO:0008270 (zinc ion binding) |
| Araip.8K2DG | 0.06 | F-box/LRR-like protein |
| Araip.8N7GM | 0.03 | LRR receptor-like kinase |
| Araip.8Y4J6 | 0.01 | Unknown protein |
| Araip.98UU3 | 0.04 | uncharacterized protein LOC102669519 [Glycine max]; IPR001878 (Zinc finger, CCHC-type); GO:0003676 (nucleic acid binding), GO:0008270 (zinc ion binding) |
| Araip.A1XTX | 0.01 | Zinc finger GRF-type protein n=1 Tax=Arachis hypogaea RepID=G0Y6U2_ARAHY; IPR010666 (Zinc finger, GRF-type); GO:0008270 (zinc ion binding) |
| Araip.A3T8C | 0.04 | Uroporphyrinogen decarboxylase; IPR000257 (Uroporphyrinogen decarboxylase (URO-D)); GO:0004853 (uroporphyrinogen decarboxylase activity), GO:0006779 (porphyrin-containing compound biosynthetic process) |
| Araip.AKB1V | 0.03 | Zinc knuckle family protein n=1 Tax=Oryza sativa subsp. japonica RepID=H2KWL4_ORYSJ |
| Araip.C96YB | 0.06 | Unknown protein |
| Araip.CJ5QS | 0.01 | general transcription factor 3C-like protein; IPR016040 (NAD(P)-binding domain) |
| Araip.D0RDY | 0.02 | Unknown protein |
| Araip.D483E | 0.01 | DUF241 domain protein; IPR004320 (Protein of unknown function DUF241, plant) |
| Araip.D4DBV | 0.02 | Unknown protein |
| Araip.D4HI6 | 0.02 | type I inositol 1,4,5-trisphosphate 5-phosphatase CVP2-like isoform X1 [Glycine max]; IPR005135 (Endonuclease/exonuclease/phosphatase) |
| Araip.D65RU | 0.02 | Unknown protein |
| Araip.D9D6E | 0.02 | NADH-ubiquinone oxidoreductase chain 4 n=7 Tax=Medicago RepID=D4HNF2_9FABA |
| Araip.DK6GB | 0.07 | VAC14-like protein; IPR021841 (Vacuolar protein 14 C-terminal Fig4-binding domain), IPR026825 (Vacuole morphology and inheritance protein 14) |
| Araip.E0ZL3 | 0.01 | Leucine-rich repeat protein kinase family protein |
| Araip.ECK9I | 0.03 | integral membrane Yip1 family protein |
| Araip.EV3K6 | 0.03 | Heavy metal transport/detoxification superfamily protein, putative n=1 Tax=Theobroma cacao RepID=UPI00042B223C |
| Araip.FGF62 | 0.06 | Defective in cullin neddylation protein n=2 Tax=Medicago truncatula RepID=G7L1Z9_MEDTR |
| Araip.FK40K | 0.02 | beta-amyrin synthase-like isoform X2 [Glycine max]; IPR008930 (Terpenoid cyclases/protein prenyltransferase alpha-alpha toroid) |
| Araip.FW6V3 | 0.07 | bZIP transcription factor |
| Araip.H5LMW | 0.02 | ATP binding microtubule motor family protein; IPR001752 (Kinesin, motor domain), IPR027417 (P-loop containing nucleoside triphosphate hydrolase), IPR027640 (Kinesin-like protein); GO:0003777 (microtubule motor activity), GO:0005524 (ATP binding), GO:0005871 (kinesin complex), GO:0007018 (microtubule-based movement), GO:0008017 (microtubule binding) |
| Araip.H618Q | 0.05 | Unknown protein |
| Araip.HF7IH | 0.04 | Unknown protein |
| Araip.HL0YC | 0.04 | Unknown protein |
| Araip.HZY70 | 0.17 | NEP1-interacting protein 2-like isoform X1 [Glycine max] |
| Araip.I50S6 | 0.01 | strictosidine synthase 1-like [Glycine max]; IPR009091 (Regulator of chromosome condensation 1/beta-lactamase-inhibitor protein II) |
| Araip.IHI4W | 0.01 | serine/threonine-protein phosphatase 7 long form homolog [Glycine max]; IPR019557 (Aminotransferase-like, plant mobile domain) |
| Araip.J467Z | 0.04 | Unknown protein |
| Araip.K0CYD | 0.03 | 2,4-dihydroxyhept-2-ene-1,7-dioic acid aldolase, putative; IPR015813 (Pyruvate/Phosphoenolpyruvate kinase-like domain); GO:0003824 (catalytic activity), GO:0006725 (cellular aromatic compound metabolic process), GO:0016830 (carbon-carbon lyase activity) |
| Araip.K67FC | 0.01 | Unknown protein |
| Araip.KK305 | 0.01 | uncharacterized protein LOC100778822 isoform X6 [Glycine max]; IPR004252 (Probable transposase, Ptta/En/Spm, plant) |
| Araip.L2LZZ | 0.1 | protein PHYLLO, chloroplastic-like isoform X1 [Glycine max] |
| Araip.M6DPT | 0.03 | FAR-RED impaired response-like protein; IPR004330 (FAR1 DNA binding domain) |
| Araip.MWM4J | 0.02 | Ulp1 protease family, carboxy-terminal domain protein; IPR003653 (Peptidase C48, SUMO/Sentrin/Ubl1); GO:0006508 (proteolysis), GO:0008234 (cysteine-type peptidase activity) |
| Araip.NP2VV | 0.03 | ATPase family AAA domain-containing protein 1-like [Glycine max]; IPR027417 (P-loop containing nucleoside triphosphate hydrolase); GO:0005524 (ATP binding) |
| Araip.NQP0X | 0.01 | Potassium transporter family protein; IPR003855 (K+ potassium transporter); GO:0015079 (potassium ion transmembrane transporter activity), GO:0016020 (membrane), GO:0071805 (potassium ion transmembrane transport) |
| Araip.P23B5 | 0.02 | Unknown protein |
| Araip.PFZ06 | 0.05 | Calcium-binding EF hand family protein |
| Araip.PN7Y2 | 0.01 | protein FAR1-RELATED SEQUENCE 6-like isoform X2 [Glycine max]; IPR006564 (Zinc finger, PMZ-type); GO:0008270 (zinc ion binding) |
| Araip.Q5U4J | 0.04 | Unknown protein |
| Araip.QB4A1 | 0.01 | serine/threonine-protein phosphatase 7 long form homolog [Glycine max]; IPR019557 (Aminotransferase-like, plant mobile domain) |
| Araip.QK3TH | 0.03 | uncharacterized protein LOC100794856 [Glycine max] |
| Araip.QN693 | 0.01 | Unknown protein |
| Araip.QR58A | 0.01 | Unknown protein |
| Araip.QRV7L | 0.01 | serine/threonine-protein phosphatase 7 long form homolog [Glycine max]; IPR019557 (Aminotransferase-like, plant mobile domain) |
| Araip.QW6LV | 0.02 | protein FAR1-RELATED SEQUENCE 12-like isoform X2 [Glycine max]; IPR004330 (FAR1 DNA binding domain) |
| Araip.R4H0G | 0.04 | uncharacterized protein LOC100793882 isoform X3 [Glycine max]; IPR013666 (Pleckstrin-like, plant) |
| Araip.R5P18 | 0.03 | Unknown protein |
| Araip.R7FV0 | 0.02 | Pentatricopeptide repeat (PPR) superfamily protein |
| Araip.RDW8L | 0.01 | 40S ribosomal protein S3a-1; IPR001593 (Ribosomal protein S3Ae), IPR002818 (ThiJ/PfpI); GO:0003735 (structural constituent of ribosome), GO:0005622 (intracellular), GO:0005840 (ribosome), GO:0006412 (translation) |
| Araip.RJH7U | 0.04 | transcription initiation factor TFIID subunit-like protein |
| Araip.S8JEE | 0.05 | beta-galactosidase 3; IPR001944 (Glycoside hydrolase, family 35), IPR017853 (Glycoside hydrolase, superfamily); GO:0005975 (carbohydrate metabolic process) |
| Araip.S95J4 | 0.02 | Unknown protein |
| Araip.SIC5N | 0.01 | zinc finger MYM-type protein 1-like [Glycine max]; IPR006580 (Zinc finger, TTF-type), IPR008906 (HAT dimerisation domain, C-terminal), IPR012337 (Ribonuclease H-like domain), IPR025398 (Domain of unknown function DUF4371); GO:0003676 (nucleic acid binding), GO:0046983 (protein dimerization activity) |
| Araip.SU2VF | 0.01 | protein n=1 Tax=Oryza sativa subsp. japonica RepID=C7J340_ORYSJ; IPR004332 (Transposase, MuDR, plant) |
| Araip.SYR8N | 0.01 | RNA-directed DNA polymerase homolog [Glycine max]; IPR000477 (Reverse transcriptase domain); GO:0003723 (RNA binding), GO:0003964 (RNA-directed DNA polymerase activity), GO:0006278 (RNA-dependent DNA replication) |
| Araip.T1YIW | 0.01 | serine/threonine-protein phosphatase 7 long form homolog [Glycine max]; IPR019557 (Aminotransferase-like, plant mobile domain) |
| Araip.T2Q6Y | 0.05 | Unknown protein |
| Araip.T2UX1 | 0.12 | Unknown protein |
| Araip.T99DR | 0.03 | DNA binding; nucleotide binding; nucleic acid binding; DNA-directed DNA polymerases; DNA-directed DNA polymerases; IPR012337 (Ribonuclease H-like domain); GO:0003676 (nucleic acid binding), GO:0003887 (DNA-directed DNA polymerase activity), GO:0006260 (DNA replication) |
| Araip.TA3U9 | 0.01 | peptide transporter 1; IPR000109 (Proton-dependent oligopeptide transporter family), IPR016196 (Major facilitator superfamily domain, general substrate transporter); GO:0005215 (transporter activity), GO:0006810 (transport), GO:0016020 (membrane) |
| Araip.U4YSI | 0.03 | Unknown protein |
| Araip.U9ULS | 0.01 | Intracellular protease, PfpI family n=2 Tax=Desulfovibrio RepID=B8DJ91_DESVM; IPR002818 (ThiJ/PfpI) |
| Araip.UII8Y | 0.01 | photosystem I P700 chlorophyll A apoprotein; IPR001280 (Photosystem I PsaA/PsaB); GO:0009522 (photosystem I), GO:0009579 (thylakoid), GO:0015979 (photosynthesis), GO:0016021 (integral component of membrane) |
| Araip.UJ8LG | 0.02 | FAR-RED impaired response-like protein; IPR004330 (FAR1 DNA binding domain) |
| Araip.UMC1H | 0.04 | Ycf2 [Glycine max]; IPR008543 (Uncharacterised protein family Ycf2); GO:0005524 (ATP binding), GO:0009507 (chloroplast) |
| Araip.UQL24 | 0.33 | Unknown protein |
| Araip.USY3M | 0.03 | ribulose bisphosphate carboxylase large chain |
| Araip.UT4IL | 0.02 | NADP-dependent alkenal double bond reductase P1; IPR002085 (Alcohol dehydrogenase superfamily, zinc-type), IPR013149 (Alcohol dehydrogenase, C-terminal), IPR016040 (NAD(P)-binding domain); GO:0008270 (zinc ion binding), GO:0016491 (oxidoreductase activity), GO:0055114 (oxidation-reduction process) |
| Araip.UU5SB | 0.03 | Unknown protein |
| Araip.UV9BG | 0.01 | Unknown protein |
| Araip.V5NIN | 0.02 | Unknown protein |
| Araip.VV00M | 0.01 | protein FAR1-RELATED SEQUENCE 6-like isoform X2 [Glycine max]; IPR007527 (Zinc finger, SWIM-type); GO:0008270 (zinc ion binding) |
| Araip.VZS4K | 0.05 | serine/threonine-protein phosphatase 7 long form homolog [Glycine max]; IPR019557 (Aminotransferase-like, plant mobile domain) |
| Araip.W3M81 | 0.01 | serine/threonine-protein phosphatase 7 long form homolog [Glycine max]; IPR019557 (Aminotransferase-like, plant mobile domain) |
| Araip.WD68J | 0.01 | uncharacterized protein LOC102664163 isoform X7 [Glycine max] |
| Araip.WH9XR | 0.04 | transmembrane protein, putative |
| Araip.WTZ3T | 0.02 | Unknown protein |
| Araip.XA3FU | 0.02 | receptor-like protein |
| Araip.XVR2T | 0.03 | glucose-6-phosphate isomerase; IPR001672 (Phosphoglucose isomerase (PGI)); GO:0004347 (glucose-6-phosphate isomerase activity), GO:0006094 (gluconeogenesis), GO:0006096 (glycolysis) |
| Araip.Y9IQB | 0.01 | serine/threonine-protein phosphatase 7 long form homolog isoform X2 [Glycine max]; IPR019557 (Aminotransferase-like, plant mobile domain) |
| Araip.Y9N8B | 0.01 | flocculation protein FLO11-like isoform X3 [Glycine max] |
| Araip.Z2UT4 | 0.01 | glutathione S-transferase tau 5; IPR010987 (Glutathione S-transferase, C-terminal-like), IPR012336 (Thioredoxin-like fold); GO:0005515 (protein binding) |
| Araip.ZJN21 | 0.04 | Pentatricopeptide repeat (PPR) superfamily protein; IPR002885 (Pentatricopeptide repeat) |
| Araip.ZS7TB | 0.03 | ethylene-responsive transcription factor 4 [Glycine max]; IPR008942 (ENTH/VHS), IPR016177 (DNA-binding domain); GO:0003677 (DNA binding), GO:0003700 (sequence-specific DNA binding transcription factor activity) |
| Later leaf |  |  |
| Aradu.09RAX | 0.01 | protein FAR1-RELATED SEQUENCE 6-like isoform X2 [Glycine max]; IPR004330 (FAR1 DNA binding domain), IPR006564 (Zinc finger, PMZ-type); GO:0008270 (zinc ion binding) |
| Aradu.18UGV | 0.02 | Ycf2 [Glycine max] |
| Aradu.1I7E9 | 0.01 | flowering locus protein T; IPR008914 (Phosphatidylethanolamine-binding protein PEBP) |
| Aradu.27Z47 | 0.03 | uncharacterized protein LOC100799047 isoform X5 [Glycine max] |
| Aradu.2T8PE | 0.01 | E3 ubiquitin-protein ligase n=2 Tax=Vitis vinifera RepID=F6HSM1_VITVI |
| Aradu.459CZ | 0.02 | serine/threonine-protein phosphatase 7 long form homolog [Glycine max]; IPR019557 (Aminotransferase-like, plant mobile domain) |
| Aradu.59MTH | 0.01 | auxin transporter-like protein 1-like [Glycine max] |
| Aradu.5KK7K | 0.01 | Unknown protein |
| Aradu.6WP30 | 0.01 | Polyprotein n=1 Tax=Citrus endogenous pararetrovirus RepID=V9QEM3_9RETR |
| Aradu.72X71 | 0.01 | serine/threonine-protein phosphatase 7 long form homolog [Glycine max]; IPR019557 (Aminotransferase-like, plant mobile domain) |
| Aradu.7QZ7K | 0.01 | multidrug MFS transporter n=1 Tax=Pleurocapsa sp. PCC 7319 RepID=UPI000348FB96; IPR010099 (Sugar nucleotide epimerase YfcH,-like putative) |
| Aradu.96CJC | 0.02 | Unknown protein |
| Aradu.9H1HU | 0.03 | F-box family protein; IPR001810 (F-box domain), IPR017451 (F-box associated interaction domain); GO:0005515 (protein binding) |
| Aradu.A43I1 | 0.05 | Unknown protein |
| Aradu.A4QZ4 | 0.06 | Unknown protein |
| Aradu.BF50I | 0.01 | Disease resistance protein (TIR-NBS-LRR class) family; IPR002182 (NB-ARC), IPR027417 (P-loop containing nucleoside triphosphate hydrolase); GO:0043531 (ADP binding) |
| Aradu.C5QE3 | 0.01 | mitochondrial import inner membrane translocase subunit Tim9-like isoform X3 [Glycine max]; IPR004217 (Tim10/DDP family zinc finger) |
| Aradu.D2VSM | 0.03 | JHL23C09.7 protein n=1 Tax=Jatropha curcas RepID=E6NUA1_JATCU |
| Aradu.E1M5Z | 0.01 | serine/threonine-protein phosphatase 7 long form homolog [Glycine max]; IPR019557 (Aminotransferase-like, plant mobile domain) |
| Aradu.ED3AE | 0.02 | Unknown protein |
| Aradu.EG6H7 | 0.01 | uncharacterized protein LOC100800865 isoform X3 [Glycine max]; IPR004332 (Transposase, MuDR, plant) |
| Aradu.EGV6I | 0.01 | receptor like protein 33; IPR001611 (Leucine-rich repeat); GO:0005515 (protein binding) |
| Aradu.FNZ6D | 0.03 | uncharacterized protein LOC100785552 [Glycine max]; IPR025322 (Protein of unknown function DUF4228, plant) |
| Aradu.GB7Z4 | 0.07 | Galactosyltransferase family protein; IPR002659 (Glycosyl transferase, family 31); GO:0006486 (protein glycosylation), GO:0008378 (galactosyltransferase activity), GO:0016020 (membrane) |
| Aradu.H8XMU | 0.03 | AP2-like ethylene-responsive transcription factor BBM2-like [Glycine max] |
| Aradu.HBI25 | 0.01 | rho GTPase-activating protein 2-like [Glycine max]; IPR000095 (CRIB domain), IPR008936 (Rho GTPase activation protein); GO:0005622 (intracellular), GO:0007165 (signal transduction) |
| Aradu.HG8HI | 0.01 | GRF zinc finger protein; IPR010666 (Zinc finger, GRF-type); GO:0008270 (zinc ion binding) |
| Aradu.J9C3K | 0.03 | cysteine-rich receptor-like protein kinase 10-like [Glycine max] |
| Aradu.JM87E | 0.05 | Unknown protein |
| Aradu.JUE7L | 0.02 | Unknown protein |
| Aradu.M2VBP | 0.01 | GRF zinc finger protein; IPR010666 (Zinc finger, GRF-type); GO:0008270 (zinc ion binding) |
| Aradu.NT2EE | 0.02 | Unknown protein |
| Aradu.P7QVI | 0.02 | Malate dehydrogenase (Oxaloacetate-decarboxylating) (NADP(+)) n=1 Tax=Sorangium cellulosum (strain So ce56) RepID=A9G3I5_SORC5; IPR016040 (NAD(P)-binding domain); GO:0004471 (malate dehydrogenase (decarboxylating) (NAD+) activity), GO:0051287 (NAD binding), GO:0055114 (oxidation-reduction process) |
| Aradu.PHQ2K | 0.02 | ubiquitin-conjugating enzyme 32 |
| Aradu.Q0DH2 | 0.03 | Unknown protein |
| Aradu.Q5F54 | 0.01 | Pectate lyase family protein; IPR011050 (Pectin lyase fold/virulence factor), IPR018082 (AmbAllergen) |
| Aradu.Q5HAX | 0.01 | HXXXD-type acyl-transferase family protein; IPR003480 (Transferase), IPR023213 (Chloramphenicol acetyltransferase-like domain) |
| Aradu.QJ0HH | 0.04 | protein n=1 Tax=Oryza sativa subsp. japonica RepID=C7J340_ORYSJ; IPR007527 (Zinc finger, SWIM-type); GO:0008270 (zinc ion binding) |
| Aradu.R0LQU | 0.01 | uncharacterized protein LOC100800865 isoform X3 [Glycine max]; IPR004332 (Transposase, MuDR, plant) |
| Aradu.R562E | 0.04 | Unknown protein |
| Aradu.RDS3V | 0.03 | Unknown protein |
| Aradu.T1RMF | 0.02 | Unknown protein |
| Aradu.U9E9K | 0.01 | FAR1-related sequence 5; IPR004330 (FAR1 DNA binding domain) |
| Aradu.VGM0B | 0.03 | NADH-quinone oxidoreductase protein; IPR001750 (NADH:ubiquinone/plastoquinone oxidoreductase); GO:0008137 (NADH dehydrogenase (ubiquinone) activity), GO:0055114 (oxidation-reduction process) |
| Aradu.VNF1V | 0.01 | SAC3/GANP/Nin1/mts3/eIF-3 p25 family; IPR005062 (SAC3/GANP/Nin1/mts3/eIF-3 p25) |
| Aradu.W393E | 0.01 | Unknown protein |
| Aradu.W9084 | 0.01 | F-box protein interaction domain protein; IPR001810 (F-box domain), IPR011043 (Galactose oxidase/kelch, beta-propeller), IPR017451 (F-box associated interaction domain); GO:0005515 (protein binding) |
| Aradu.WZQ8L | 0.01 | NADH-ubiquinone oxidoreductase chain 4 n=2 Tax=Spermatophyta RepID=N0DNX9_PHAAN |
| Aradu.X5GIV | 0.01 | Unknown protein; IPR003653 (Peptidase C48, SUMO/Sentrin/Ubl1); GO:0006508 (proteolysis), GO:0008234 (cysteine-type peptidase activity) |
| Aradu.X6HFY | 0.01 | Unknown protein; IPR019557 (Aminotransferase-like, plant mobile domain) |
| Aradu.Y4DRW | 0.02 | ABC transporter C family member 3-like isoform X1 [Glycine max]; IPR027417 (P-loop containing nucleoside triphosphate hydrolase); GO:0005524 (ATP binding), GO:0016887 (ATPase activity) |
| Aradu.Z5ZY7 | 0.01 | GRF zinc finger protein; IPR010666 (Zinc finger, GRF-type); GO:0008270 (zinc ion binding) |
| Aradu.ZP2JX | 0.02 | wall-associated receptor kinase-like 8-like [Glycine max] |
| Araip.0FH0C | 0.03 | receptor-like protein kinase 2; IPR001611 (Leucine-rich repeat), IPR003591 (Leucine-rich repeat, typical subtype), IPR013210 (Leucine-rich repeat-containing N-terminal, type 2); GO:0005515 (protein binding) |
| Araip.0I9CT | 0.01 | n=1 Tax=Oryza sativa subsp. japonica RepID=Q7XS05_ORYSJ |
| Araip.12IQX | 0.01 | serine/threonine-protein phosphatase 7 long form homolog [Glycine max]; IPR019557 (Aminotransferase-like, plant mobile domain) |
| Araip.132W1 | 0.05 | Unknown protein |
| Araip.17PIN | 0.01 | Unknown protein |
| Araip.2C3YA | 0.01 | uncharacterized protein LOC100820019 isoform X5 [Glycine max] |
| Araip.2N7Q7 | 0.01 | n=1 Tax=Oryza sativa subsp. japonica RepID=Q7F971_ORYSJ; IPR001878 (Zinc finger, CCHC-type), IPR004332 (Transposase, MuDR, plant), IPR007527 (Zinc finger, SWIM-type); GO:0003676 (nucleic acid binding), GO:0008270 (zinc ion binding) |
| Araip.2SR14 | 0.02 | 30S ribosomal protein S2, chloroplastic n=414 Tax=Mesangiospermae RepID=RR2_VITVI; IPR000568 (ATPase, F0 complex, subunit A), IPR001865 (Ribosomal protein S2), IPR023591 (Ribosomal protein S2, flavodoxin-like domain); GO:0003735 (structural constituent of ribosome), GO:0005622 (intracellular), GO:0005840 (ribosome), GO:0006412 (translation), GO:0015078 (hydrogen ion transmembrane transporter activity), GO:0015935 (small ribosomal subunit), GO:0015986 (ATP synthesis coupled proton transport) |
| Araip.3H2LW | 0.11 | Sugar transporter SWEET n=2 Tax=Citrus RepID=V4U1G9_9ROSI ; GO:0016021 (integral component of membrane) |
| Araip.3N6MS | 0.02 | RNA polymerase II, Rpb4, core protein |
| Araip.43CUM | 0.01 | uncharacterized protein LOC100792279 isoform X1 [Glycine max]; IPR009057 (Homeodomain-like); GO:0003677 (DNA binding), GO:0003682 (chromatin binding) |
| Araip.43ZG0 | 0.01 | protein FAR1-RELATED SEQUENCE 5-like [Glycine max]; IPR001878 (Zinc finger, CCHC-type), IPR004330 (FAR1 DNA binding domain), IPR006564 (Zinc finger, PMZ-type); GO:0003676 (nucleic acid binding), GO:0008270 (zinc ion binding) |
| Araip.45AJT | 0.03 | 30S ribosomal protein S14, chloroplastic n=7 Tax=Mesangiospermae RepID=RR14_LEMMI; IPR001209 (Ribosomal protein S14); GO:0003735 (structural constituent of ribosome), GO:0005622 (intracellular), GO:0005840 (ribosome), GO:0006412 (translation) |
| Araip.48Y96 | 0.01 | serine/threonine-protein phosphatase 7 long form homolog [Glycine max]; IPR019557 (Aminotransferase-like, plant mobile domain) |
| Araip.4ZT13 | 0.01 | serine/threonine-protein phosphatase 7 long form homolog [Glycine max]; IPR019557 (Aminotransferase-like, plant mobile domain) |
| Araip.50S6B | 0.02 | Unknown protein |
| Araip.51F2R | 0.08 | hypothetical protein |
| Araip.5X2RH | 0.01 | protein FAR1-RELATED SEQUENCE 6-like isoform X2 [Glycine max]; IPR007527 (Zinc finger, SWIM-type); GO:0008270 (zinc ion binding) |
| Araip.69UA7 | 0.01 | Protein kinase superfamily protein; IPR011009 (Protein kinase-like domain); GO:0004672 (protein kinase activity), GO:0005524 (ATP binding), GO:0006468 (protein phosphorylation) |
| Araip.727T3 | 0.02 | protein FAR1-RELATED SEQUENCE 6-like isoform X2 [Glycine max]; IPR007527 (Zinc finger, SWIM-type); GO:0008270 (zinc ion binding) |
| Araip.77TND | 0.01 | uncharacterized protein LOC102661763 [Glycine max] |
| Araip.7NN4K | 0.01 | Ulp1 protease family, carboxy-terminal domain protein; IPR003653 (Peptidase C48, SUMO/Sentrin/Ubl1); GO:0006508 (proteolysis), GO:0008234 (cysteine-type peptidase activity) |
| Araip.82TCQ | 0.02 | n=1 Tax=Oryza sativa subsp. japonica RepID=Q5CAI2_ORYSJ |
| Araip.8RL2K | 0.02 | Unknown protein |
| Araip.9130E | 0.01 | serine/threonine-protein phosphatase 7 long form homolog [Glycine max]; IPR019557 (Aminotransferase-like, plant mobile domain) |
| Araip.9D2L2 | 0.02 | protein FAR1-RELATED SEQUENCE 5-like [Glycine max]; IPR001878 (Zinc finger, CCHC-type), IPR004330 (FAR1 DNA binding domain); GO:0003676 (nucleic acid binding), GO:0008270 (zinc ion binding) |
| Araip.A85FN | 0.01 | protein FAR1-RELATED SEQUENCE 12-like isoform X1 [Glycine max]; IPR004330 (FAR1 DNA binding domain) |
| Araip.AWR9N | 0.02 | GRF zinc finger protein; IPR010666 (Zinc finger, GRF-type); GO:0008270 (zinc ion binding) |
| Araip.C66EI | 0.01 | uncharacterized protein LOC102667659 [Glycine max] |
| Araip.D3SGH | 0.01 | Helicase-like protein n=1 Tax=Medicago truncatula RepID=G7J0A2_MEDTR |
| Araip.DH1E6 | 0.01 | Zinc finger GRF-type protein n=1 Tax=Arachis hypogaea RepID=G0Y6U2_ARAHY |
| Araip.DIY5M | 0.01 | n=1 Tax=Oryza sativa subsp. japonica RepID=Q7XRM9_ORYSJ |
| Araip.DK6NC | 0.04 | Unknown protein |
| Araip.DLA7V | 0.01 | serine/threonine-protein phosphatase 7 long form homolog [Glycine max]; IPR019557 (Aminotransferase-like, plant mobile domain) |
| Araip.ED8IZ | 0.02 | Unknown protein |
| Araip.ER1FK | 0.02 | Unknown protein |
| Araip.F6VX2 | 0.02 | PttB n=1 Tax=Medicago truncatula RepID=G7K5I5_MEDTR |
| Araip.FBL3D | 0.02 | cinnamyl alcohol dehydrogenase |
| Araip.FS20H | 0.03 | Unknown protein |
| Araip.FYN1T | 0.01 | uncharacterized protein LOC102664163 isoform X7 [Glycine max]; IPR004252 (Probable transposase, Ptta/En/Spm, plant) |
| Araip.G4N41 | 0.08 | ATP-dependent Clp protease proteolytic protein; IPR023562 (Clp protease proteolytic subunit /Translocation-enhancing protein TepA); GO:0004252 (serine-type endopeptidase activity), GO:0006508 (proteolysis) |
| Araip.H1PDA | 0.01 | protein FAR1-RELATED SEQUENCE 6-like isoform X2 [Glycine max]; IPR004330 (FAR1 DNA binding domain) |
| Araip.H6TMI | 0.01 | Unknown protein |
| Araip.HJ21V | 0.01 | E3 ubiquitin-protein ligase n=5 Tax=Solanum RepID=M1BDZ1_SOLTU |
| Araip.HLT5M | 0.01 | Protein kinase superfamily protein; IPR000023 (Phosphofructokinase domain), IPR011009 (Protein kinase-like domain); GO:0003872 (6-phosphofructokinase activity), GO:0004672 (protein kinase activity), GO:0005524 (ATP binding), GO:0005945 (6-phosphofructokinase complex), GO:0006096 (glycolysis), GO:0006468 (protein phosphorylation) |
| Araip.I1716 | 0.01 | serine/threonine-protein phosphatase 7 long form homolog [Glycine max]; IPR019557 (Aminotransferase-like, plant mobile domain) |
| Araip.I7KG4 | 0.08 | nuclear matrix constituent protein-related |
| Araip.IDZ3P | 0.01 | protein FAR1-RELATED SEQUENCE 6-like isoform X2 [Glycine max]; IPR004330 (FAR1 DNA binding domain) |
| Araip.IL35N | 0.13 | Catalytic/ protein phosphatase type 2C/ protein serine/threonine phosphatase n=2 Tax=Papilionoideae RepID=G7J6T1_MEDTR; IPR001932 (Protein phosphatase 2C (PP2C)-like domain); GO:0003824 (catalytic activity), GO:0004722 (protein serine/threonine phosphatase activity), GO:0006470 (protein dephosphorylation) |
| Araip.IR03X | 0.02 | photosystem II reaction center protein H; IPR001056 (Photosystem II PsbH, phosphoprotein); GO:0009523 (photosystem II), GO:0015979 (photosynthesis), GO:0016020 (membrane), GO:0042301 (phosphate ion binding), GO:0050821 (protein stabilization) |
| Araip.J79NL | 0.01 | Unknown protein |
| Araip.JY4UI | 0.01 | protein FAR1-RELATED SEQUENCE 5-like [Glycine max]; IPR007527 (Zinc finger, SWIM-type); GO:0008270 (zinc ion binding) |
| Araip.KD3P3 | 0.02 | hypothetical protein |
| Araip.L7B77 | 0.01 | serine/threonine-protein phosphatase 7 long form homolog [Glycine max]; IPR019557 (Aminotransferase-like, plant mobile domain) |
| Araip.LEN56 | 0.03 | Defective in cullin neddylation protein n=4 Tax=Citrus RepID=V4UX97_9ROSI |
| Araip.MFT4K | 0.02 | probable ubiquitin-like-specific protease 2B-like isoform X3 [Glycine max] |
| Araip.N8NYX | 0.02 | protein n=1 Tax=Oryza sativa subsp. japonica RepID=C7J9W8_ORYSJ; IPR007527 (Zinc finger, SWIM-type); GO:0008270 (zinc ion binding) |
| Araip.P3TAV | 0.06 | mitogen-activated protein kinase kinase kinase 21; IPR011009 (Protein kinase-like domain) |
| Araip.PC8YA | 0.02 | DUF241 domain protein; IPR004320 (Protein of unknown function DUF241, plant) |
| Araip.Q5R2V | 0.01 | GRF zinc finger protein; IPR010666 (Zinc finger, GRF-type); GO:0008270 (zinc ion binding) |
| Araip.Q8L8H | 0.04 | photosystem II protein; IPR000484 (Photosynthetic reaction centre, L/M); GO:0009772 (photosynthetic electron transport in photosystem II) |
| Araip.RC3QB | 0.01 | serine/threonine-protein phosphatase 7 long form homolog isoform X2 [Glycine max] |
| Araip.S1GID | 0.02 | Unknown protein |
| Araip.S2DR4 | 0.04 | Unknown protein |
| Araip.S8PJI | 0.03 | Unknown protein; IPR001763 (Rhodanese-like domain) |
| Araip.S9T92 | 0.02 | replication factor-A carboxy-terminal domain protein; IPR012340 (Nucleic acid-binding, OB-fold) |
| Araip.T1NAP | 0.01 | Unknown protein; IPR003653 (Peptidase C48, SUMO/Sentrin/Ubl1); GO:0006508 (proteolysis), GO:0008234 (cysteine-type peptidase activity) |
| Araip.TJ2RV | 0.01 | receptor-like kinase; IPR001611 (Leucine-rich repeat), IPR013210 (Leucine-rich repeat-containing N-terminal, type 2); GO:0005515 (protein binding) |
| Araip.TP1BS | 0.01 | Helicase-like protein n=1 Tax=Medicago truncatula RepID=G7JG32_MEDTR; IPR025476 (Helitron helicase-like domain) |
| Araip.TQ31F | 0.01 | uncharacterized protein LOC100809482 isoform X5 [Glycine max]; IPR007700 (Protein of unknown function DUF668) |
| Araip.U0593 | 0.03 | FGGY family of carbohydrate kinase |
| Araip.UIR7V | 0.04 | PPR containing plant-like protein |
| Araip.V7EGI | 0.01 | Unknown protein |
| Araip.VJI5M | 0.01 | zinc finger CCCH domain-containing protein 48-like isoform X2 [Glycine max]; IPR015943 (WD40/YVTN repeat-like-containing domain); GO:0005515 (protein binding) |
| Araip.VTG84 | 0.03 | Unknown protein |
| Araip.VTX88 | 0.03 | Unknown protein |
| Araip.XQU3L | 0.02 | NAD(P)H-quinone oxidoreductase subunit K; IPR006137 (NADH:ubiquinone oxidoreductase-like, 20kDa subunit); GO:0051536 (iron-sulfur cluster binding), GO:0055114 (oxidation-reduction process) |
| Araip.XR6XW | 0.01 | Unknown protein |
| Araip.Z03P0 | 0.08 | Unknown protein |
| Araip.Z2EE7 | 0.01 | protein FAR1-RELATED SEQUENCE 6-like isoform X2 [Glycine max]; IPR007527 (Zinc finger, SWIM-type); GO:0008270 (zinc ion binding) |
| Araip.Z871F | 0.01 | serine/threonine-protein phosphatase 7 long form homolog [Glycine max]; IPR019557 (Aminotransferase-like, plant mobile domain) |
| Araip.ZP7QC | 0.01 | Sugar transporter SWEET n=2 Tax=Solanum RepID=K4B8C8_SOLLC ; GO:0016021 (integral component of membrane) |
| Vegetative shoot | |  |
| Aradu.0S9KB | 0.06 | Glutamyl-tRNA(Gln) amidotransferase subunit A, chloroplastic/mitochondrial n=2 Tax=Papilionoideae RepID=G7IZY8_MEDTR; IPR000120 (Amidase), IPR023631 (Amidase signature domain) |
| Aradu.14VEG | 0.03 | Unknown protein |
| Aradu.23L8Z | 0.2 | F-box/FBD/LRR-repeat protein At3g14710-like [Glycine max] |
| Aradu.271S6 | 0.02 | Galactose oxidase/kelch repeat superfamily protein; IPR001810 (F-box domain), IPR008906 (HAT dimerisation domain, C-terminal), IPR012337 (Ribonuclease H-like domain), IPR015916 (Galactose oxidase, beta-propeller); GO:0003676 (nucleic acid binding), GO:0005515 (protein binding), GO:0046983 (protein dimerization activity) |
| Aradu.39FGL | 0.08 | uncharacterized protein LOC102662489 isoform X1 [Glycine max] |
| Aradu.412CE | 0.05 | Unknown protein |
| Aradu.61MQR | 0.09 | uncharacterized protein LOC100793937 [Glycine max] |
| Aradu.64DUS | 0.11 | Unknown protein |
| Aradu.66H3X | 0.06 | Unknown protein |
| Aradu.70FEI | 0.03 | hypothetical protein |
| Aradu.9EE58 | 0.03 | Unknown protein |
| Aradu.AP4D9 | 0.04 | short-chain dehydrogenase-reductase B; IPR002198 (Short-chain dehydrogenase/reductase SDR); GO:0008152 (metabolic process), GO:0016491 (oxidoreductase activity) |
| Aradu.BV83A | 0.08 | Unknown protein; IPR009027 (Ribosomal protein L9/RNase H1, N-terminal) |
| Aradu.CQ642 | 0.03 | hypothetical protein |
| Aradu.E0XYA | 0.04 | protein FAR1-RELATED SEQUENCE 5-like [Glycine max] |
| Aradu.EN9I9 | 0.15 | Unknown protein |
| Aradu.F8I5L | 0.04 | Alpha-L RNA-binding motif/Ribosomal protein S4 family protein; IPR002942 (RNA-binding S4 domain); GO:0003723 (RNA binding) |
| Aradu.FG7MN | 0.11 | Unknown protein |
| Aradu.HH1DZ | 0.08 | Unknown protein |
| Aradu.KD7QN | 0.05 | Cyclin B1; 4 |
| Aradu.RGH0Y | 0.05 | Unknown protein |
| Aradu.RII91 | 0.04 | Alpha-L RNA-binding motif/Ribosomal protein S4 family protein; IPR002942 (RNA-binding S4 domain); GO:0003723 (RNA binding) |
| Aradu.TGX18 | 0.03 | Helicase-like protein n=1 Tax=Medicago truncatula RepID=G7KPQ9_MEDTR; IPR025476 (Helitron helicase-like domain) |
| Aradu.U8B7M | 0.02 | nucleolin 1-like [Glycine max] |
| Aradu.W1WN9 | 0.1 | Unknown protein |
| Aradu.X1RIB | 0.04 | regulator of telomere elongation helicase-like protein; IPR006555 (ATP-dependent helicase, C-terminal); GO:0003676 (nucleic acid binding), GO:0005524 (ATP binding), GO:0006139 (nucleobase-containing compound metabolic process), GO:0008026 (ATP-dependent helicase activity) |
| Araip.0BZ8N | 0.03 | Catalytic/ protein phosphatase type 2C/ protein serine/threonine phosphatase n=2 Tax=Papilionoideae RepID=G7J6T1_MEDTR; IPR001932 (Protein phosphatase 2C (PP2C)-like domain); GO:0003824 (catalytic activity), GO:0004722 (protein serine/threonine phosphatase activity), GO:0006470 (protein dephosphorylation) |
| Araip.0EK4Q | 0.05 | serine/threonine-protein phosphatase 7 long form homolog [Glycine max]; IPR019557 (Aminotransferase-like, plant mobile domain) |
| Araip.21KDR | 0.04 | serine/threonine-protein phosphatase 7 long form homolog [Glycine max]; IPR019557 (Aminotransferase-like, plant mobile domain) |
| Araip.252QI | 0.03 | Unknown protein |
| Araip.26HXI | 0.02 | uncharacterized protein At2g29880-like [Glycine max]; IPR024752 (Myb/SANT-like domain) |
| Araip.33PU5 | 0.01 | arginyl-tRNA-protein transferase; IPR016181 (Acyl-CoA N-acyltransferase), IPR017137 (Arginine-tRNA-protein transferase 1, eukaryotic); GO:0004057 (arginyltransferase activity), GO:0016598 (protein arginylation) |
| Araip.45PGJ | 0.04 | double-stranded-RNA-binding protein 4; IPR014720 (Double-stranded RNA-binding domain) |
| Araip.56DXT | 0.07 | LORELEI-LIKE-GPI-ANCHORED PROTEIN 1 |
| Araip.5H75Y | 0.03 | Unknown protein |
| Araip.75B5J | 0.09 | aldehyde dehydrogenase family 3 member F1 [Glycine max]; IPR016162 (Aldehyde dehydrogenase, N-terminal); GO:0008152 (metabolic process), GO:0016491 (oxidoreductase activity), GO:0055114 (oxidation-reduction process) |
| Araip.80WRQ | 0.05 | disease resistance protein (TIR-NBS-LRR class), putative |
| Araip.8H2GI | 0.15 | Unknown protein |
| Araip.A372L | 0.24 | Unknown protein |
| Araip.A9KWJ | 0.15 | ribulose bisphosphate carboxylase large chain domain protein; IPR000685 (Ribulose bisphosphate carboxylase, large subunit, C-terminal); GO:0000287 (magnesium ion binding) |
| Araip.AWM0X | 0.01 | serine/threonine-protein phosphatase 7 long form homolog [Glycine max]; IPR019557 (Aminotransferase-like, plant mobile domain) |
| Araip.C2UEQ | 0.02 | n=1 Tax=Oryza sativa subsp. japonica RepID=Q7X8F3_ORYSJ |
| Araip.DNQ5S | 0.1 | Unknown protein |
| Araip.EM1U3 | 0.04 | ABC transporter B family protein |
| Araip.JU3GD | 0.18 | Unknown protein |
| Araip.M1PPS | 0.02 | Unknown protein; IPR004330 (FAR1 DNA binding domain) |
| Araip.M8TH1 | 0.06 | uncharacterized protein LOC102662489 isoform X2 [Glycine max] |
| Araip.QT1N2 | 0.02 | protein FAR1-RELATED SEQUENCE 6-like isoform X2 [Glycine max]; IPR007527 (Zinc finger, SWIM-type); GO:0008270 (zinc ion binding) |
| Araip.RBS2M | 0.02 | protein FAR1-RELATED SEQUENCE 6-like isoform X2 [Glycine max]; IPR004330 (FAR1 DNA binding domain), IPR007527 (Zinc finger, SWIM-type); GO:0008270 (zinc ion binding) |
| Araip.RFZ0T | 0.07 | 6b-interacting protein 2 n=1 Tax=Nicotiana tabacum RepID=B1B5X5_TOBAC |
| Araip.RG6BR | 0.11 | CTV.20 n=1 Tax=Poncirus trifoliata RepID=Q8H6Q8_PONTR |
| Araip.RID67 | 0.06 | phosphoglucan phosphatase LSF2, chloroplastic-like [Glycine max] |
| Araip.S95SW | 0.04 | Unknown protein |
| Araip.T3ML8 | 0.01 | RNA-directed DNA polymerase homolog [Glycine max]; IPR000477 (Reverse transcriptase domain), IPR001878 (Zinc finger, CCHC-type); GO:0003676 (nucleic acid binding), GO:0003723 (RNA binding), GO:0003964 (RNA-directed DNA polymerase activity), GO:0006278 (RNA-dependent DNA replication), GO:0008270 (zinc ion binding) |
| Araip.T908W | 0.06 | Unknown protein |
| Araip.YU8XN | 0.02 | 3-oxo-5-alpha-steroid 4-dehydrogenase family protein; IPR001104 (3-oxo-5-alpha-steroid 4-dehydrogenase, C-terminal); GO:0005737 (cytoplasm), GO:0006629 (lipid metabolic process), GO:0016021 (integral component of membrane) |
| Reproductive shoot | |  |
| Aradu.0746P | 0.02 | hypothetical protein |
| Aradu.07IAJ | 0.01 | Zinc knuckle family protein n=1 Tax=Oryza sativa subsp. japonica RepID=H2KW38_ORYSJ; IPR001878 (Zinc finger, CCHC-type); GO:0003676 (nucleic acid binding), GO:0008270 (zinc ion binding) |
| Aradu.09SBV | 0.03 | uncharacterized protein LOC102669491 isoform X2 [Glycine max] |
| Aradu.0G9H8 | 0.03 | uncharacterized protein LOC102659952 isoform X5 [Glycine max]; IPR007021 (Domain of unknown function DUF659) |
| Aradu.0N746 | 0.03 | uncharacterized protein LOC102668708 isoform X2 [Glycine max]; IPR008974 (TRAF-like); GO:0005515 (protein binding) |
| Aradu.0R2MC | 0.05 | Unknown protein |
| Aradu.0S8DA | 0.04 | Unknown protein |
| Aradu.12C9A | 0.03 | UPF0505 protein-like isoform X2 [Glycine max] |
| Aradu.16N2V | 1.67 | Unknown protein |
| Aradu.1H4AP | 1.79 | transcription factor ABORTED MICROSPORES-like isoform X3 [Glycine max]; IPR004252 (Probable transposase, Ptta/En/Spm, plant) |
| Aradu.1Z51J | 0.09 | Unknown protein |
| Aradu.2H1FS | 0.02 | protein FAR1-RELATED SEQUENCE 5-like [Glycine max]; IPR007527 (Zinc finger, SWIM-type); GO:0008270 (zinc ion binding) |
| Aradu.2IN3E | 0.01 | heat shock protein 70; IPR013126 (Heat shock protein 70 family) |
| Aradu.3579H | 0.04 | glucose-6-phosphate isomerase; IPR001672 (Phosphoglucose isomerase (PGI)), IPR021135 (Phosphoenolpyruvate carboxylase); GO:0004347 (glucose-6-phosphate isomerase activity), GO:0006094 (gluconeogenesis), GO:0006096 (glycolysis), GO:0006099 (tricarboxylic acid cycle), GO:0008964 (phosphoenolpyruvate carboxylase activity), GO:0015977 (carbon fixation) |
| Aradu.37SXQ | 0.09 | MATE efflux family protein |
| Aradu.3DU6T | 0.03 | serine/threonine-protein phosphatase 7 long form homolog [Glycine max]; IPR019557 (Aminotransferase-like, plant mobile domain) |
| Aradu.3F7AN | 0.08 | Unknown protein; IPR010666 (Zinc finger, GRF-type); GO:0008270 (zinc ion binding) |
| Aradu.3T2DT | 0.01 | uncharacterized protein LOC100797259 isoform X4 [Glycine max]; IPR001878 (Zinc finger, CCHC-type), IPR004332 (Transposase, MuDR, plant); GO:0003676 (nucleic acid binding), GO:0008270 (zinc ion binding) |
| Aradu.4632I | 0.03 | uncharacterized protein LOC100793937 [Glycine max]; IPR025312 (Domain of unknown function DUF4216) |
| Aradu.46U1T | 0.02 | NAC domain containing protein 28; IPR003441 (NAC domain); GO:0003677 (DNA binding) |
| Aradu.4B259 | 0.03 | Unknown protein |
| Aradu.4BT6A | 0.06 | Unknown protein |
| Aradu.4X3T1 | 14.66 | leguminosin group486 secreted peptide; IPR010264 (Plant self-incompatibility S1) |
| Aradu.4Y2WU | 0.02 | Unknown protein |
| Aradu.52ZWJ | 0.02 | FAR1-related sequence 10; IPR004330 (FAR1 DNA binding domain) |
| Aradu.53LCI | 0.01 | Ulp1 protease family, carboxy-terminal domain protein |
| Aradu.5GH1I | 0.22 | uncharacterized protein LOC100792919 isoform X4 [Glycine max] |
| Aradu.5Y4IB | 0.05 | pectinesterase 11; IPR011050 (Pectin lyase fold/virulence factor); GO:0005618 (cell wall), GO:0030599 (pectinesterase activity), GO:0042545 (cell wall modification) |
| Aradu.6888Y | 0.88 | Unknown protein |
| Aradu.69ZTL | 0.03 | Zinc finger MYM-type protein n=1 Tax=Medicago truncatula RepID=G7IL89_MEDTR; IPR008906 (HAT dimerisation domain, C-terminal), IPR012337 (Ribonuclease H-like domain); GO:0003676 (nucleic acid binding), GO:0046983 (protein dimerization activity) |
| Aradu.6BD4D | 0.03 | glycolipid transfer protein 1; IPR014830 (Glycolipid transfer protein domain); GO:0005737 (cytoplasm), GO:0017089 (glycolipid transporter activity), GO:0046836 (glycolipid transport), GO:0051861 (glycolipid binding) |
| Aradu.6DK48 | 0.02 | serine/threonine-protein phosphatase 7 long form homolog [Glycine max]; IPR019557 (Aminotransferase-like, plant mobile domain) |
| Aradu.6FC6A | 0.05 | Unknown protein |
| Aradu.6J25Z | 0.02 | Mitovirus RNA-dependent RNA polymerase; IPR008686 (RNA-dependent RNA polymerase, mitoviral) |
| Aradu.78DPI | 0.02 | CTV.20 n=1 Tax=Poncirus trifoliata RepID=Q8H6Q8_PONTR |
| Aradu.7H228 | 0.03 | Unknown protein; IPR010666 (Zinc finger, GRF-type); GO:0008270 (zinc ion binding) |
| Aradu.7L9NI | 0.06 | arginine/serine-rich coiled coil protein |
| Aradu.7PQ7F | 0.02 | replication protein A 70 kDa DNA-binding subunit C-like [Glycine max]; IPR012340 (Nucleic acid-binding, OB-fold) |
| Aradu.89VQ1 | 0.12 | Unknown protein |
| Aradu.97AME | 0.02 | disease resistance protein (TIR-NBS-LRR class), putative; IPR000767 (Disease resistance protein), IPR027417 (P-loop containing nucleoside triphosphate hydrolase); GO:0006952 (defense response), GO:0043531 (ADP binding) |
| Aradu.9853W | 0.02 | serine/threonine-protein phosphatase 7 long form homolog [Glycine max]; IPR019557 (Aminotransferase-like, plant mobile domain) |
| Aradu.A1HB3 | 0.03 | Unknown protein |
| Aradu.A6TP6 | 0.09 | Unknown protein |
| Aradu.AKB8L | 0.01 | serine/threonine-protein phosphatase 7 long form homolog [Glycine max]; IPR019557 (Aminotransferase-like, plant mobile domain) |
| Aradu.AL625 | 0.02 | zinc finger MYM-type protein 1-like [Glycine max]; IPR008906 (HAT dimerisation domain, C-terminal), IPR012337 (Ribonuclease H-like domain); GO:0003676 (nucleic acid binding), GO:0046983 (protein dimerization activity) |
| Aradu.BB920 | 0.01 | tRNA wybutosine-synthesizing protein 1 homolog [Glycine max] |
| Aradu.BI2TM | 0.13 | serine/threonine-protein phosphatase 7 long form homolog [Glycine max]; IPR019557 (Aminotransferase-like, plant mobile domain) |
| Aradu.C75E5 | 0.04 | Unknown protein |
| Aradu.CB26G | 0.01 | protein pelota-like [Glycine max]; IPR004405 (Translation release factor pelota) |
| Aradu.CLZ5M | 0.01 | receptor-like kinase 902; IPR025875 (Leucine rich repeat 4) |
| Aradu.CXZ9C | 0.04 | uncharacterized protein LOC102663175 [Glycine max]; IPR008974 (TRAF-like); GO:0005515 (protein binding) |
| Aradu.D1FGN | 0.03 | arginine/serine-rich coiled coil protein |
| Aradu.DG9SV | 0.08 | Unknown protein |
| Aradu.DUR94 | 0.01 | serine/threonine-protein phosphatase 7 long form homolog [Glycine max]; IPR019557 (Aminotransferase-like, plant mobile domain) |
| Aradu.E6J8T | 0.01 | uncharacterized protein LOC100797259 isoform X4 [Glycine max]; IPR001878 (Zinc finger, CCHC-type), IPR004332 (Transposase, MuDR, plant), IPR007527 (Zinc finger, SWIM-type); GO:0003676 (nucleic acid binding), GO:0008270 (zinc ion binding) |
| Aradu.F36DY | 0.01 | Unknown protein |
| Aradu.FC9JY | 0.08 | DUF223 domain protein; IPR012340 (Nucleic acid-binding, OB-fold) |
| Aradu.FK3G3 | 0.03 | phosphatidylinositol 4-phosphate 5-kinase 4-like [Glycine max]; IPR001487 (Bromodomain), IPR002498 (Phosphatidylinositol-4-phosphate 5-kinase, core), IPR003311 (AUX/IAA protein), IPR023610 (Phosphatidylinositol-4-phosphate 5-kinase), IPR027483 (Phosphatidylinositol-4-phosphate 5-kinase, C-terminal); GO:0005515 (protein binding), GO:0005634 (nucleus), GO:0016307 (phosphatidylinositol phosphate kinase activity), GO:0046488 (phosphatidylinositol metabolic process), GO:0046983 (protein dimerization activity) |
| Aradu.FN562 | 0.35 | cytochrome P450 705A5 [Glycine max] |
| Aradu.FSE8H | 1.27 | Unknown protein |
| Aradu.FZ4W4 | 0.05 | RAN binding protein 1 |
| Aradu.G0B5Z | 0.03 | Unknown protein |
| Aradu.G1GW1 | 0.03 | Unknown protein |
| Aradu.G8B40 | 0.07 | chalcone synthase [Glycine max]; IPR016039 (Thiolase-like); GO:0003824 (catalytic activity), GO:0008152 (metabolic process), GO:0009058 (biosynthetic process) |
| Aradu.I20NV | 0.05 | Transposon protein n=1 Tax=Arachis hypogaea RepID=G0Y6V7_ARAHY |
| Aradu.I2JAX | 0.02 | Ulp1 protease family, carboxy-terminal domain protein; IPR003653 (Peptidase C48, SUMO/Sentrin/Ubl1); GO:0006508 (proteolysis), GO:0008234 (cysteine-type peptidase activity) |
| Aradu.IK08B | 0.03 | receptor-like kinase 1; IPR013210 (Leucine-rich repeat-containing N-terminal, type 2), IPR025875 (Leucine rich repeat 4) |
| Aradu.JE2H0 | 0.04 | two-component response regulator ARR2-like [Glycine max]; IPR009057 (Homeodomain-like), IPR011006 (CheY-like superfamily); GO:0000156 (phosphorelay response regulator activity), GO:0000160 (phosphorelay signal transduction system), GO:0003677 (DNA binding) |
| Aradu.JEW9E | 0.06 | serine/threonine-protein phosphatase 7 long form homolog [Glycine max]; IPR019557 (Aminotransferase-like, plant mobile domain) |
| Aradu.JJ9LB | 0.01 | protein n=1 Tax=Oryza sativa subsp. japonica RepID=C7J9W8_ORYSJ |
| Aradu.JN2RB | 0.04 | uncharacterized protein At2g29880-like [Glycine max]; IPR024752 (Myb/SANT-like domain) |
| Aradu.JW828 | 0.07 | uncharacterized protein LOC102666531 [Glycine max] |
| Aradu.JYU1S | 0.02 | Mitovirus RNA-dependent RNA polymerase; IPR008686 (RNA-dependent RNA polymerase, mitoviral) |
| Aradu.K4NTV | 0.11 | 50S ribosomal protein L2 |
| Aradu.KEH3G | 0.03 | replication factor-A carboxy-terminal domain protein |
| Aradu.L0G5R | 0.2 | Heavy metal transport/detoxification superfamily protein; IPR006121 (Heavy metal-associated domain, HMA); GO:0030001 (metal ion transport), GO:0046872 (metal ion binding) |
| Aradu.L1LJC | 0.04 | ORF45f n=1 Tax=Pinus koraiensis RepID=Q85WT0_PINKO |
| Aradu.L5MSW | 0.07 | Unknown protein |
| Aradu.L82DA | 0.06 | Unknown protein |
| Aradu.LW5Z2 | 0.02 | protein n=1 Tax=Oryza sativa subsp. japonica RepID=C7J340_ORYSJ; IPR004332 (Transposase, MuDR, plant) |
| Aradu.MJC8J | 0.04 | nucleolin 1-like [Glycine max] |
| Aradu.N0BLW | 0.02 | Membrane transporter D1 n=3 Tax=Andropogoneae RepID=B6U4Q3_MAIZE; IPR005828 (General substrate transporter), IPR016196 (Major facilitator superfamily domain, general substrate transporter); GO:0016020 (membrane), GO:0016021 (integral component of membrane), GO:0022857 (transmembrane transporter activity), GO:0022891 (substrate-specific transmembrane transporter activity), GO:0055085 (transmembrane transport) |
| Aradu.N8N4N | 0.03 | SNF1-related kinase regulatory subunit beta-2; IPR014756 (Immunoglobulin E-set) |
| Aradu.NL1HL | 0.02 | FAR1-related sequence 5; IPR004330 (FAR1 DNA binding domain), IPR007527 (Zinc finger, SWIM-type); GO:0008270 (zinc ion binding) |
| Aradu.PN9TC | 0.04 | Cytochrome P450 superfamily protein; IPR001128 (Cytochrome P450); GO:0005506 (iron ion binding), GO:0020037 (heme binding), GO:0055114 (oxidation-reduction process) |
| Aradu.PQ2RV | 0.06 | protein ASPARTIC PROTEASE IN GUARD CELL 1-like [Glycine max]; IPR021109 (Aspartic peptidase domain) |
| Aradu.PRG7Z | 0.04 | Unknown protein |
| Aradu.PU69R | 0.03 | CLP1-similar protein 5; IPR010655 (Pre-mRNA cleavage complex II Clp1), IPR027417 (P-loop containing nucleoside triphosphate hydrolase) |
| Aradu.PUX5Q | 1.51 | GDSL-like Lipase/Acylhydrolase superfamily protein; IPR001087 (Lipase, GDSL); GO:0006629 (lipid metabolic process), GO:0016787 (hydrolase activity) |
| Aradu.Q3EMG | 0.02 | protein FAR1-RELATED SEQUENCE 7-like isoform X2 [Glycine max]; IPR004330 (FAR1 DNA binding domain), IPR007527 (Zinc finger, SWIM-type); GO:0008270 (zinc ion binding) |
| Aradu.QG58S | 0.04 | F-box protein interaction domain protein; IPR001810 (F-box domain); GO:0005515 (protein binding) |
| Aradu.QHS2G | 0.09 | uncharacterized protein LOC102669519 [Glycine max] |
| Aradu.RM9D5 | 0.06 | HEAT SHOCK PROTEIN 89.1; IPR001404 (Heat shock protein Hsp90 family); GO:0005524 (ATP binding), GO:0006457 (protein folding), GO:0006950 (response to stress), GO:0051082 (unfolded protein binding) |
| Aradu.RMF67 | 0.02 | CTV.20 n=1 Tax=Poncirus trifoliata RepID=Q8H6Q8_PONTR |
| Aradu.RN59F | 0.01 | serine/threonine-protein phosphatase 7 long form homolog [Glycine max]; IPR019557 (Aminotransferase-like, plant mobile domain) |
| Aradu.RRM7Q | 0.07 | uncharacterized protein LOC102666531 [Glycine max] |
| Aradu.U01CD | 0.29 | B3 DNA-binding domain protein; IPR015300 (DNA-binding pseudobarrel domain); GO:0003677 (DNA binding) |
| Aradu.U8YJ6 | 0.04 | Unknown protein |
| Aradu.U9AKX | 0.06 | LRR and NB-ARC domain disease resistance protein |
| Aradu.UXX37 | 0.05 | n=1 Tax=Oryza sativa subsp. japonica RepID=Q7XU62_ORYSJ; IPR007527 (Zinc finger, SWIM-type); GO:0008270 (zinc ion binding) |
| Aradu.V4FJI | 0.05 | Ycf1 n=47 Tax=Mentheae RepID=S4T8D9_ORIVU |
| Aradu.W1WQU | 5.09 | LOB domain-containing protein 2; IPR004883 (Lateral organ boundaries, LOB) |
| Aradu.W27BV | 0.07 | serine/threonine-protein phosphatase 7 long form homolog [Glycine max]; IPR019557 (Aminotransferase-like, plant mobile domain) |
| Aradu.W3E39 | 0.09 | Unknown protein |
| Aradu.W5D1Q | 0.06 | 2-methyl-6-phytylbenzoquinone methyltranferase; IPR013216 (Methyltransferase type 11); GO:0008152 (metabolic process), GO:0008168 (methyltransferase activity) |
| Aradu.WN32D | 0.01 | Ulp1 protease family, carboxy-terminal domain protein; IPR003653 (Peptidase C48, SUMO/Sentrin/Ubl1); GO:0006508 (proteolysis), GO:0008234 (cysteine-type peptidase activity) |
| Aradu.WYE4I | 0.02 | serine/threonine-protein phosphatase 7 long form homolog [Glycine max]; IPR019557 (Aminotransferase-like, plant mobile domain) |
| Aradu.X1ANV | 0.02 | global transcription factor group protein; IPR001487 (Bromodomain); GO:0005515 (protein binding) |
| Aradu.X1XML | 0.07 | Unknown protein |
| Aradu.X51AF | 0.86 | 7SK snRNA methylphosphate capping enzyme n=3 Tax=Boreoeutheria RepID=G3I651_CRIGR; IPR010456 (Ribosomal L11 methyltransferase, PrmA), IPR010675 (Bicoid-interacting 3); GO:0005737 (cytoplasm), GO:0006479 (protein methylation), GO:0008168 (methyltransferase activity), GO:0008276 (protein methyltransferase activity) |
| Aradu.XR86W | 0.17 | Unknown protein |
| Aradu.XS047 | 0.02 | uncharacterized protein LOC100797259 isoform X4 [Glycine max]; IPR001878 (Zinc finger, CCHC-type); GO:0003676 (nucleic acid binding), GO:0008270 (zinc ion binding) |
| Aradu.Y5115 | 0.01 | Cytochrome P450 superfamily protein; IPR001128 (Cytochrome P450); GO:0005506 (iron ion binding), GO:0020037 (heme binding), GO:0055114 (oxidation-reduction process) |
| Aradu.Y5GD6 | 0.12 | Unknown protein |
| Aradu.YB3HA | 0.01 | uncharacterized protein LOC102663857 [Glycine max] |
| Aradu.YGK3H | 0.05 | probable receptor-like protein kinase At1g67000-like isoform X3 [Glycine max]; IPR011009 (Protein kinase-like domain) |
| Aradu.YKM8J | 0.04 | Helicase-like protein n=1 Tax=Oryza sativa subsp. japonica RepID=Q6YTQ6_ORYSJ |
| Aradu.Z6UQQ | 0.06 | Auxin-responsive family protein; IPR004877 (Cytochrome b561, eukaryote), IPR005018 (DOMON domain), IPR017214 (Uncharacterised conserved protein UCP037471); GO:0016021 (integral component of membrane) |
| Aradu.ZJQ6Y | 0.06 | PLANT CADMIUM RESISTANCE 2; IPR006461 (Uncharacterised protein family Cys-rich) |
| Aradu.ZW8JA | 0.06 | Unknown protein |
| Araip.093NU | 0.01 | serine/threonine-protein phosphatase 7 long form homolog [Glycine max]; IPR019557 (Aminotransferase-like, plant mobile domain) |
| Araip.0F5DW | 0.04 | Transposon protein n=1 Tax=Arachis hypogaea RepID=G0Y6V7_ARAHY |
| Araip.0HF3D | 0.02 | TNP1 n=1 Tax=Medicago truncatula RepID=G7K958_MEDTR |
| Araip.0I39J | 0.01 | serine/threonine-protein phosphatase 7 long form homolog [Glycine max]; IPR019557 (Aminotransferase-like, plant mobile domain) |
| Araip.0MQ1C | 1.24 | Major facilitator superfamily protein; IPR010658 (Nodulin-like) |
| Araip.108MH | 0.09 | auxin response factor 17; IPR010525 (Auxin response factor), IPR015300 (DNA-binding pseudobarrel domain); GO:0003677 (DNA binding), GO:0005634 (nucleus), GO:0009725 (response to hormone) |
| Araip.139H0 | 0.06 | zinc finger CCCH domain-containing protein 13-like isoform X2 [Glycine max] |
| Araip.17PG7 | 0.03 | Unknown protein |
| Araip.1872M | 0.08 | uncharacterized mitochondrial protein AtMg00810-like [Glycine max] |
| Araip.1J0F0 | 0.04 | 4,5-DOPA dioxygenase extradiol-like protein; IPR004183 (Extradiol ring-cleavage dioxygenase, class III enzyme, subunit B); GO:0006725 (cellular aromatic compound metabolic process), GO:0008198 (ferrous iron binding), GO:0016491 (oxidoreductase activity) |
| Araip.1R5ZB | 0.02 | putative ubiquitin-like-specific protease 1B-like isoform X3 [Glycine max]; IPR003653 (Peptidase C48, SUMO/Sentrin/Ubl1); GO:0006508 (proteolysis), GO:0008234 (cysteine-type peptidase activity) |
| Araip.1S7HT | 0.01 | Unknown protein |
| Araip.1V2RF | 0.04 | Unknown protein |
| Araip.1V67D | 0.04 | Gibberellin-regulated family protein; IPR003854 (Gibberellin regulated protein) |
| Araip.22URS | 0.04 | respiratory burst oxidase homologue D |
| Araip.2A9YP | 0.01 | E3 ubiquitin-protein ligase XBAT33-like isoform X1 [Glycine max]; IPR020683 (Ankyrin repeat-containing domain); GO:0005515 (protein binding) |
| Araip.2MX5Q | 0.01 | Unknown protein |
| Araip.3H14C | 1.08 | Unknown protein |
| Araip.41MB0 | 0.03 | Unknown protein |
| Araip.42DTV | 0.01 | Pentatricopeptide repeat (PPR) superfamily protein |
| Araip.48WYY | 0.07 | MYB transcription factor MYB64 [Glycine max]; IPR009057 (Homeodomain-like); GO:0003677 (DNA binding) |
| Araip.4I7GM | 0.03 | hypothetical protein |
| Araip.4P18L | 0.07 | Unknown protein |
| Araip.4X4MZ | 0.09 | Family of unknown function (DUF566); IPR007573 (Protein of unknown function DUF566) |
| Araip.57EWM | 0.08 | hypothetical protein |
| Araip.582SS | 0.04 | Zinc finger MYM-type protein n=1 Tax=Medicago truncatula RepID=G7IY07_MEDTR; IPR025398 (Domain of unknown function DUF4371) |
| Araip.58FK6 | 0.01 | n=1 Tax=Oryza sativa subsp. japonica RepID=Q7XU62_ORYSJ |
| Araip.5JV8M | 0.09 | Unknown protein |
| Araip.5R38D | 9.51 | LOB domain-containing protein 2; IPR004883 (Lateral organ boundaries, LOB) |
| Araip.6Z9G4 | 0.01 | uncharacterized protein LOC100776940 isoform X4 [Glycine max]; IPR004332 (Transposase, MuDR, plant), IPR007527 (Zinc finger, SWIM-type); GO:0008270 (zinc ion binding) |
| Araip.71HA7 | 0.04 | Unknown protein |
| Araip.72XP5 | 0.06 | GRF zinc finger protein; IPR010666 (Zinc finger, GRF-type); GO:0008270 (zinc ion binding) |
| Araip.73R6C | 0.02 | protein FAR1-RELATED SEQUENCE 6-like isoform X2 [Glycine max]; IPR004330 (FAR1 DNA binding domain), IPR007527 (Zinc finger, SWIM-type); GO:0008270 (zinc ion binding) |
| Araip.75NY1 | 0.01 | UDP-Glycosyltransferase superfamily protein; IPR002213 (UDP-glucuronosyl/UDP-glucosyltransferase), IPR010264 (Plant self-incompatibility S1), IPR010666 (Zinc finger, GRF-type); GO:0008152 (metabolic process), GO:0008270 (zinc ion binding) |
| Araip.7RN3S | 0.09 | Zinc finger MYM-type protein n=1 Tax=Medicago truncatula RepID=G7LFJ4_MEDTR |
| Araip.80WIT | 0.01 | E3 ubiquitin-protein ligase n=5 Tax=Solanum RepID=M1BDZ1_SOLTU; IPR003653 (Peptidase C48, SUMO/Sentrin/Ubl1); GO:0006508 (proteolysis), GO:0008234 (cysteine-type peptidase activity) |
| Araip.837EW | 0.14 | Unknown protein |
| Araip.8M725 | 0.01 | DUF21 domain-containing protein isoform X2 [Glycine max]; IPR007877 (Protein of unknown function DUF707), IPR013087 (Zinc finger C2H2-type/integrase DNA-binding domain); GO:0003676 (nucleic acid binding) |
| Araip.91144 | 0.01 | uncharacterized protein LOC100800865 isoform X3 [Glycine max]; IPR004332 (Transposase, MuDR, plant) |
| Araip.93XX8 | 0.09 | Unknown protein |
| Araip.97LFH | 2.2 | Unknown protein |
| Araip.97U8J | 0.01 | serine/threonine-protein phosphatase 7 long form homolog [Glycine max]; IPR019557 (Aminotransferase-like, plant mobile domain) |
| Araip.9D9GU | 0.01 | RNA-directed DNA polymerase homolog [Glycine max]; IPR000477 (Reverse transcriptase domain), IPR001878 (Zinc finger, CCHC-type); GO:0003676 (nucleic acid binding), GO:0003723 (RNA binding), GO:0003964 (RNA-directed DNA polymerase activity), GO:0006278 (RNA-dependent DNA replication), GO:0008270 (zinc ion binding) |
| Araip.9I52R | 0.01 | nucleolin 1-like [Glycine max] |
| Araip.A3Y00 | 0.06 | replication factor-A carboxy-terminal domain protein |
| Araip.AB04Q | 0.07 | pectinesterase 11; IPR011050 (Pectin lyase fold/virulence factor); GO:0005618 (cell wall), GO:0030599 (pectinesterase activity), GO:0042545 (cell wall modification) |
| Araip.AH9TM | 0.03 | Unknown protein |
| Araip.ALX3U | 0.05 | NAD(P)H:quinone oxidoreductase, type IV protein; IPR016040 (NAD(P)-binding domain); GO:0004471 (malate dehydrogenase (decarboxylating) (NAD+) activity), GO:0051287 (NAD binding), GO:0055114 (oxidation-reduction process) |
| Araip.AN559 | 0.03 | Werner syndrome-like exonuclease; IPR012337 (Ribonuclease H-like domain); GO:0003676 (nucleic acid binding), GO:0006139 (nucleobase-containing compound metabolic process), GO:0008408 (3'-5' exonuclease activity) |
| Araip.AY61M | 0.03 | Myb/SANT-like DNA-binding domain protein; IPR024752 (Myb/SANT-like domain) |
| Araip.B9TWE | 0.02 | Polyprotein n=1 Tax=Citrus endogenous pararetrovirus RepID=V9QEM3_9RETR |
| Araip.BC8ET | 0.02 | F-box protein interaction domain protein; IPR001810 (F-box domain), IPR017451 (F-box associated interaction domain); GO:0005515 (protein binding) |
| Araip.CC4RZ | 0.06 | F-box protein interaction domain protein; IPR001810 (F-box domain), IPR011043 (Galactose oxidase/kelch, beta-propeller), IPR017451 (F-box associated interaction domain); GO:0005515 (protein binding) |
| Araip.CDY0S | 0.05 | Unknown protein |
| Araip.CH327 | 0.01 | RNA-directed DNA polymerase homolog [Glycine max]; IPR000477 (Reverse transcriptase domain), IPR001878 (Zinc finger, CCHC-type); GO:0003676 (nucleic acid binding), GO:0003723 (RNA binding), GO:0003964 (RNA-directed DNA polymerase activity), GO:0006278 (RNA-dependent DNA replication), GO:0008270 (zinc ion binding) |
| Araip.D6RS0 | 0.01 | viral movement protein |
| Araip.D79D1 | 0.76 | DUF3755 family protein |
| Araip.DCT9V | 0.01 | protein FAR1-RELATED SEQUENCE 6-like isoform X2 [Glycine max]; IPR004330 (FAR1 DNA binding domain), IPR007527 (Zinc finger, SWIM-type); GO:0008270 (zinc ion binding) |
| Araip.DUQ43 | 0.05 | Unknown protein; IPR001878 (Zinc finger, CCHC-type); GO:0003676 (nucleic acid binding), GO:0008270 (zinc ion binding) |
| Araip.DV2WW | 0.01 | protein FAR1-RELATED SEQUENCE 5-like [Glycine max]; IPR004330 (FAR1 DNA binding domain) |
| Araip.F0ITC | 0.02 | Unknown protein |
| Araip.FMV0I | 0.08 | mitochondrial pyruvate carrier 1-like isoform X5 [Glycine max]; IPR005336 (Mitochondrial pyruvate carrier); GO:0005743 (mitochondrial inner membrane), GO:0006850 (mitochondrial pyruvate transport) |
| Araip.GIL07 | 0.05 | Calcium-binding EF-hand family protein; IPR011992 (EF-hand domain pair); GO:0005509 (calcium ion binding) |
| Araip.GU914 | 0.09 | Pentatricopeptide repeat (PPR-like) superfamily protein; IPR002885 (Pentatricopeptide repeat) |
| Araip.H5CC0 | 0.23 | Unknown protein |
| Araip.HI284 | 0.01 | protein FAR1-RELATED SEQUENCE 6-like isoform X2 [Glycine max]; IPR004330 (FAR1 DNA binding domain), IPR006564 (Zinc finger, PMZ-type); GO:0008270 (zinc ion binding) |
| Araip.HTM0X | 0.18 | leguminosin group486 secreted peptide; IPR010264 (Plant self-incompatibility S1) |
| Araip.HUQ4W | 0.04 | lipoxygenase 1; IPR000907 (Lipoxygenase); GO:0046872 (metal ion binding), GO:0055114 (oxidation-reduction process) |
| Araip.I16YM | 1.05 | leguminosin group486 secreted peptide; IPR010264 (Plant self-incompatibility S1) |
| Araip.I1DFI | 0.28 | protein FAR1-RELATED SEQUENCE 6-like isoform X2 [Glycine max]; IPR004330 (FAR1 DNA binding domain) |
| Araip.I6KFJ | 0.05 | Unknown protein |
| Araip.IAK9D | 0.05 | Unknown protein |
| Araip.IJ1LM | 1.39 | leguminosin group486 secreted peptide; IPR010264 (Plant self-incompatibility S1) |
| Araip.IQP7X | 0.19 | Unknown protein |
| Araip.J41L9 | 0.08 | protein FAR1-RELATED SEQUENCE 6-like isoform X2 [Glycine max]; IPR006564 (Zinc finger, PMZ-type); GO:0008270 (zinc ion binding) |
| Araip.J9FJT | 0.02 | zinc finger (C3HC4-type RING finger) family protein; IPR001607 (Zinc finger, UBP-type), IPR013083 (Zinc finger, RING/FYVE/PHD-type); GO:0008270 (zinc ion binding) |
| Araip.K2ALY | 0.03 | Unknown protein |
| Araip.K56L2 | 0.18 | Unknown protein |
| Araip.KEM3Z | 0.13 | heat shock protein 70; IPR013126 (Heat shock protein 70 family) |
| Araip.L1C3P | 0.03 | Unknown protein |
| Araip.L2JFZ | 0.02 | bromodomain-containing protein 9-like [Glycine max]; IPR001487 (Bromodomain); GO:0005515 (protein binding) |
| Araip.L4G8E | 0.05 | GRF zinc finger protein; IPR010666 (Zinc finger, GRF-type); GO:0008270 (zinc ion binding) |
| Araip.L52FQ | 0.03 | Unknown protein |
| Araip.L5SZS | 0.01 | zinc finger MYM-type protein 1-like [Glycine max]; IPR012337 (Ribonuclease H-like domain), IPR025398 (Domain of unknown function DUF4371); GO:0003676 (nucleic acid binding) |
| Araip.L7PZX | 0.04 | Unknown protein |
| Araip.L8JRF | 0.02 | Unknown protein |
| Araip.LN16T | 0.06 | paired amphipathic helix protein Sin3-like 4-like isoform X2 [Glycine max]; IPR003822 (Paired amphipathic helix); GO:0005634 (nucleus) |
| Araip.LN9X0 | 0.16 | protein FAR1-RELATED SEQUENCE 6-like isoform X2 [Glycine max]; IPR006564 (Zinc finger, PMZ-type); GO:0008270 (zinc ion binding) |
| Araip.M37X3 | 0.08 | Unknown protein |
| Araip.MX9UF | 0.02 | E3 ubiquitin-protein ligase n=5 Tax=Solanum RepID=M1BDZ1_SOLTU |
| Araip.N04V2 | 0.02 | Unknown protein; IPR003653 (Peptidase C48, SUMO/Sentrin/Ubl1); GO:0006508 (proteolysis), GO:0008234 (cysteine-type peptidase activity) |
| Araip.N24A4 | 0.07 | Unknown protein |
| Araip.NFY3N | 0.01 | molybdenum cofactor sulfurase |
| Araip.NG6WE | 0.05 | F-box protein interaction domain protein; IPR001810 (F-box domain); GO:0005515 (protein binding) |
| Araip.NMK61 | 0.02 | adenine phosphoribosyltransferase 5; IPR000836 (Phosphoribosyltransferase domain); GO:0009116 (nucleoside metabolic process) |
| Araip.NQD22 | 0.06 | nodulin MtN21 /EamA-like transporter family protein |
| Araip.NYC0D | 0.07 | endoplasmic reticulum auxin binding protein 1; IPR000526 (Auxin-binding protein); GO:0004872 (receptor activity), GO:0005788 (endoplasmic reticulum lumen) |
| Araip.NZ56F | 0.08 | Non-lysosomal glucosylceramidase |
| Araip.PFN86 | 0.07 | Unknown protein |
| Araip.PJ8VB | 0.03 | sulfate transporter 1; 3; IPR002645 (STAS domain), IPR011547 (Sulphate transporter); GO:0008272 (sulfate transport), GO:0015116 (sulfate transmembrane transporter activity), GO:0016021 (integral component of membrane) |
| Araip.Q0R4V | 0.04 | Ubiquitin-protein ligase/ zinc ion binding protein n=3 Tax=Zea mays RepID=B6SL08_MAIZE; IPR002867 (Zinc finger, C6HC-type), IPR013083 (Zinc finger, RING/FYVE/PHD-type); GO:0008270 (zinc ion binding), GO:0046872 (metal ion binding) |
| Araip.Q5JY6 | 0.01 | cysteine-rich receptor-like protein kinase 8-like [Glycine max]; IPR002902 (Gnk2-homologous domain), IPR011009 (Protein kinase-like domain), IPR013320 (Concanavalin A-like lectin/glucanase, subgroup); GO:0004672 (protein kinase activity), GO:0005524 (ATP binding), GO:0006468 (protein phosphorylation) |
| Araip.Q95VT | 0.14 | Unknown protein |
| Araip.QC86V | 0.05 | rapid alkalinization factor 1; IPR008801 (Rapid ALkalinization Factor) |
| Araip.QF04Q | 0.04 | QWRF motif-containing protein 8-like isoform X3 [Glycine max]; IPR007573 (Protein of unknown function DUF566) |
| Araip.QG9WY | 0.02 | uncharacterized protein LOC100776940 isoform X4 [Glycine max]; IPR001878 (Zinc finger, CCHC-type), IPR007527 (Zinc finger, SWIM-type); GO:0003676 (nucleic acid binding), GO:0008270 (zinc ion binding) |
| Araip.QGU80 | 0.03 | putative uncharacterized protein DDB_G0282499-like [Glycine max] |
| Araip.QRK37 | 0.02 | Unknown protein |
| Araip.R51YT | 0.07 | transcription factor bHLH90-like isoform X1 [Glycine max] |
| Araip.R7L63 | 0.05 | protein FAR-RED IMPAIRED RESPONSE 1-like isoform X3 [Glycine max]; IPR004330 (FAR1 DNA binding domain) |
| Araip.RH22Z | 0.03 | Dehydrogenase/reductase SDR family member n=3 Tax=Papilionoideae RepID=G7JKG6_MEDTR; IPR002347 (Glucose/ribitol dehydrogenase); GO:0008152 (metabolic process), GO:0016491 (oxidoreductase activity) |
| Araip.RSF1H | 0.17 | RING-H2 finger protein 2B; IPR013083 (Zinc finger, RING/FYVE/PHD-type); GO:0005515 (protein binding), GO:0008270 (zinc ion binding) |
| Araip.S1VGF | 1.7 | hypothetical protein |
| Araip.S344X | 0.04 | C6HC-type zinc finger RING/U-box protein; IPR013083 (Zinc finger, RING/FYVE/PHD-type) |
| Araip.S4HBM | 0.03 | Unknown protein |
| Araip.S6RNY | 0.29 | Unknown protein |
| Araip.S8XT3 | 0.19 | Unknown protein |
| Araip.SS86R | 0.02 | serine/threonine-protein phosphatase 7 long form homolog [Glycine max]; IPR019557 (Aminotransferase-like, plant mobile domain) |
| Araip.T00B1 | 0.03 | ubiquitin-conjugating enzyme 33 |
| Araip.T0U20 | 0.04 | Gibberellin-regulated family protein; IPR003854 (Gibberellin regulated protein) |
| Araip.T8ITS | 0.06 | Defender against death (DAD family) protein; IPR003038 (DAD/Ost2); GO:0004579 (dolichyl-diphosphooligosaccharide-protein glycotransferase activity), GO:0008250 (oligosaccharyltransferase complex), GO:0016021 (integral component of membrane) |
| Araip.TA0RC | 0.01 | Cysteine proteinases superfamily protein; IPR013128 (Peptidase C1A), IPR025660 (Cysteine peptidase, histidine active site), IPR025661 (Cysteine peptidase, asparagine active site); GO:0006508 (proteolysis), GO:0008234 (cysteine-type peptidase activity) |
| Araip.U14Y9 | 0.02 | LINE-type retrotransposon LIb DNA, complete sequence, Insertion at the S10 site n=2 Tax=Ipomoea batatas RepID=Q2HX36_IPOBA; IPR025836 (Zinc knuckle CX2CX4HX4C) |
| Araip.U9WZ6 | 0.01 | serine/threonine-protein phosphatase 7 long form homolog [Glycine max]; IPR019557 (Aminotransferase-like, plant mobile domain) |
| Araip.UH6PP | 0.01 | Zinc knuckle family protein n=1 Tax=Oryza sativa subsp. japonica RepID=H2KW38_ORYSJ; IPR001878 (Zinc finger, CCHC-type); GO:0003676 (nucleic acid binding), GO:0008270 (zinc ion binding) |
| Araip.V14VA | 0.07 | sodium hydrogen exchanger 2; IPR006153 (Cation/H+ exchanger), IPR018422 (Cation/H+ exchanger, CPA1 family); GO:0006812 (cation transport), GO:0015299 (solute:hydrogen antiporter activity), GO:0016021 (integral component of membrane), GO:0055085 (transmembrane transport) |
| Araip.V7ZLK | 0.04 | SNF1-related protein kinase 1.3 |
| Araip.V85MR | 0.05 | Transposon protein n=1 Tax=Arachis hypogaea RepID=G0Y6V7_ARAHY |
| Araip.VA01H | 0.05 | Unknown protein |
| Araip.VIX6R | 0.06 | protein FAR1-RELATED SEQUENCE 6-like isoform X2 [Glycine max]; IPR007527 (Zinc finger, SWIM-type); GO:0008270 (zinc ion binding) |
| Araip.VM3AJ | 0.08 | Unknown protein |
| Araip.VN620 | 0.02 | Pentatricopeptide repeat (PPR-like) superfamily protein; IPR002885 (Pentatricopeptide repeat) |
| Araip.VTL6U | 0.03 | myb transcription factor; IPR009057 (Homeodomain-like); GO:0003677 (DNA binding), GO:0003682 (chromatin binding) |
| Araip.VU0AC | 0.05 | P-loop nucleoside triphosphate hydrolase superfamily protein; IPR026852 (Helicase Sen1-like), IPR027417 (P-loop containing nucleoside triphosphate hydrolase) |
| Araip.VV1RA | 0.01 | serine/threonine-protein phosphatase 7 long form homolog [Glycine max]; IPR019557 (Aminotransferase-like, plant mobile domain) |
| Araip.W0EII | 0.07 | Unknown protein |
| Araip.W72X4 | 0.01 | protein FAR1-RELATED SEQUENCE 6-like isoform X2 [Glycine max]; IPR006564 (Zinc finger, PMZ-type); GO:0008270 (zinc ion binding) |
| Araip.WMX3R | 0.09 | phosphate transporter 1; 1; IPR005828 (General substrate transporter), IPR016196 (Major facilitator superfamily domain, general substrate transporter); GO:0005315 (inorganic phosphate transmembrane transporter activity), GO:0006817 (phosphate ion transport), GO:0016021 (integral component of membrane), GO:0022857 (transmembrane transporter activity), GO:0055085 (transmembrane transport) |
| Araip.X0AD5 | 0.02 | serine/threonine-protein phosphatase 7 long form homolog [Glycine max]; IPR019557 (Aminotransferase-like, plant mobile domain) |
| Araip.X443P | 0.05 | arginine/serine-rich coiled coil protein |
| Araip.X715D | 39 | unknown protein |
| Araip.X8D6B | 0.01 | uncharacterized protein LOC102670101 [Glycine max]; IPR001878 (Zinc finger, CCHC-type), IPR004332 (Transposase, MuDR, plant), IPR007527 (Zinc finger, SWIM-type); GO:0003676 (nucleic acid binding), GO:0008270 (zinc ion binding) |
| Araip.XLK7P | 0.03 | Disease resistance protein (TIR-NBS-LRR class) family |
| Araip.XU9JQ | 0.03 | Translation initiation factor IF-2, putative isoform 2 n=2 Tax=Theobroma cacao RepID=UPI00042AFAF3 |
| Araip.YBI8C | 0.07 | Unknown protein |
| Araip.YL5BM | 0.13 | unknown protein |
| Araip.YVS7I | 0.03 | F-box protein interaction domain protein; IPR001810 (F-box domain), IPR017451 (F-box associated interaction domain); GO:0005515 (protein binding) |
| Araip.Z162Q | 0.03 | 60S ribosomal protein L2, related protein; IPR002171 (Ribosomal protein L2); GO:0003735 (structural constituent of ribosome), GO:0005622 (intracellular), GO:0005840 (ribosome), GO:0006412 (translation) |
| Araip.Z9C4Z | 0.04 | Retrotransposon protein, putative, Ty1-copia subclass n=1 Tax=Oryza sativa subsp. japonica RepID=Q2QWB6_ORYSJ; IPR001878 (Zinc finger, CCHC-type); GO:0003676 (nucleic acid binding), GO:0008270 (zinc ion binding) |
| Araip.Z9MZ7 | 0.01 | zinc finger MYM-type protein 1-like [Glycine max]; IPR006580 (Zinc finger, TTF-type), IPR025398 (Domain of unknown function DUF4371) |
| Araip.ZJ6XY | 0.04 | Unknown protein |
| Root |  |  |
| Aradu.02EIY | 0.01 | katanin p60 ATPase-containing subunit A1-like [Glycine max]; IPR027417 (P-loop containing nucleoside triphosphate hydrolase); GO:0000166 (nucleotide binding), GO:0005524 (ATP binding), GO:0017111 (nucleoside-triphosphatase activity) |
| Aradu.0310T | 0.01 | hypothetical protein |
| Aradu.111NR | 0.01 | Unknown protein |
| Aradu.117LS | 0.04 | Unknown protein |
| Aradu.1Y59R | 0.03 | 125 kDa kinesin-like protein |
| Aradu.20QHG | 0.01 | serine/threonine-protein phosphatase 7 long form homolog [Glycine max]; IPR019557 (Aminotransferase-like, plant mobile domain) |
| Aradu.26BRP | 0.03 | protein kinase family protein; IPR011009 (Protein kinase-like domain) |
| Aradu.26TX1 | 0.02 | Mitovirus RNA-dependent RNA polymerase; IPR008686 (RNA-dependent RNA polymerase, mitoviral) |
| Aradu.29H1P | 0.1 | Cytochrome P450 superfamily protein; IPR001128 (Cytochrome P450); GO:0005506 (iron ion binding), GO:0020037 (heme binding), GO:0055114 (oxidation-reduction process) |
| Aradu.2C8NI | 0.02 | Unknown protein |
| Aradu.2F2AY | 0.02 | Unknown protein |
| Aradu.3BG6B | 0.01 | serine/threonine-protein phosphatase 7 long form homolog [Glycine max]; IPR019557 (Aminotransferase-like, plant mobile domain) |
| Aradu.3E07Q | 0.01 | Unknown protein |
| Aradu.3H35T | 0.01 | uncharacterized protein LOC100797259 isoform X3 [Glycine max]; IPR004332 (Transposase, MuDR, plant), IPR007527 (Zinc finger, SWIM-type); GO:0008270 (zinc ion binding) |
| Aradu.47ZL6 | 0.02 | putative pentatricopeptide repeat-containing protein At5g37570-like [Glycine max]; IPR002885 (Pentatricopeptide repeat) |
| Aradu.4A356 | 0.09 | carotenoid 9,10(9',10')-cleavage dioxygenase-like isoform X1 [Glycine max]; IPR004294 (Carotenoid oxygenase) |
| Aradu.4FW5N | 0.03 | respiratory burst oxidase homologue D; IPR011992 (EF-hand domain pair); GO:0005509 (calcium ion binding) |
| Aradu.4T7TX | 0.08 | histone acetyltransferase HAC1-like isoform X3 [Glycine max]; IPR000197 (Zinc finger, TAZ-type); GO:0003712 (transcription cofactor activity), GO:0004402 (histone acetyltransferase activity), GO:0005634 (nucleus), GO:0008270 (zinc ion binding) |
| Aradu.57CQ1 | 0.02 | zinc finger MYM-type protein 1-like [Glycine max]; IPR025398 (Domain of unknown function DUF4371) |
| Aradu.5A3FQ | 0.01 | protein FAR1-RELATED SEQUENCE 6-like isoform X2 [Glycine max]; IPR004330 (FAR1 DNA binding domain), IPR007527 (Zinc finger, SWIM-type); GO:0008270 (zinc ion binding) |
| Aradu.5BK9D | 0.01 | serine/threonine-protein phosphatase 7 long form homolog [Glycine max]; IPR019557 (Aminotransferase-like, plant mobile domain) |
| Aradu.5H9U8 | 0.05 | ribosomal protein S19; IPR002222 (Ribosomal protein S19/S15), IPR023575 (Ribosomal protein S19, superfamily); GO:0003735 (structural constituent of ribosome), GO:0005840 (ribosome), GO:0006412 (translation) |
| Aradu.5QE2T | 0.02 | Unknown protein; IPR003653 (Peptidase C48, SUMO/Sentrin/Ubl1); GO:0006508 (proteolysis), GO:0008234 (cysteine-type peptidase activity) |
| Aradu.5W7T2 | 0.02 | arogenate dehydrogenase; IPR016040 (NAD(P)-binding domain) |
| Aradu.63IQV | 0.01 | uncharacterized protein LOC100803389 [Glycine max] |
| Aradu.6A0WH | 0.01 | Unknown protein |
| Aradu.78G5E | 0.01 | Pentatricopeptide repeat (PPR) superfamily protein; IPR002885 (Pentatricopeptide repeat) |
| Aradu.7P0LU | 0.01 | Tetratricopeptide repeat (TPR)-like superfamily protein |
| Aradu.7TM7Q | 0.01 | Myb/SANT-like DNA-binding domain protein; IPR024752 (Myb/SANT-like domain) |
| Aradu.859VR | 0.01 | Ulp1 protease family, carboxy-terminal domain protein; IPR003653 (Peptidase C48, SUMO/Sentrin/Ubl1); GO:0006508 (proteolysis), GO:0008234 (cysteine-type peptidase activity) |
| Aradu.8H3TU | 0.01 | protein FAR1-RELATED SEQUENCE 6-like isoform X2 [Glycine max]; IPR007527 (Zinc finger, SWIM-type); GO:0008270 (zinc ion binding) |
| Aradu.945W4 | 0.03 | Unknown protein |
| Aradu.97U1V | 0.01 | Unknown protein |
| Aradu.98P27 | 0.03 | F-box family protein; IPR001810 (F-box domain), IPR017451 (F-box associated interaction domain); GO:0005515 (protein binding) |
| Aradu.9NU5H | 0.03 | 2OG-Fe(II) oxygenase family oxidoreductase |
| Aradu.9T99P | 0.01 | caffeoylshikimate esterase-like isoform X3 [Glycine max]; IPR022742 (Putative lysophospholipase) |
| Aradu.A8MR4 | 0.02 | auxin response factor 19 |
| Aradu.AJ40W | 0.1 | Unknown protein |
| Aradu.B0UYZ | 0.03 | Tir-nbs-lrr resistance protein n=1 Tax=Medicago truncatula RepID=G7LI81_MEDTR |
| Aradu.B63C9 | 0.01 | Unknown protein |
| Aradu.BE9S0 | 0.14 | carotenoid cleavage dioxygenase 1; IPR004294 (Carotenoid oxygenase) |
| Aradu.BL1AX | 0.02 | dehydroascorbate reductase 1; IPR010987 (Glutathione S-transferase, C-terminal-like), IPR012336 (Thioredoxin-like fold) |
| Aradu.BNA6Q | 0.01 | serine/threonine-protein phosphatase 7 long form homolog [Glycine max]; IPR019557 (Aminotransferase-like, plant mobile domain) |
| Aradu.BP33J | 0.01 | Helicase, putative n=1 Tax=Oryza sativa subsp. japonica RepID=Q8H8F3_ORYSJ |
| Aradu.BP7VP | 0.1 | E3 ubiquitin-protein ligase n=5 Tax=Solanum RepID=M1BDZ1_SOLTU |
| Aradu.C1IWL | 0.02 | Unknown protein |
| Aradu.C6S61 | 0.03 | pentatricopeptide (PPR) repeat-containing protein |
| Aradu.CGA52 | 0.01 | protein FAR1-RELATED SEQUENCE 6-like isoform X2 [Glycine max]; IPR004330 (FAR1 DNA binding domain), IPR007527 (Zinc finger, SWIM-type); GO:0008270 (zinc ion binding) |
| Aradu.CXQ8J | 0.02 | Unknown protein |
| Aradu.D235C | 0.02 | Unknown protein |
| Aradu.D5X9X | 0.04 | Unknown protein |
| Aradu.D91UV | 0.06 | hypothetical protein |
| Aradu.D958N | 0.01 | protein FAR1-RELATED SEQUENCE 6-like isoform X2 [Glycine max]; IPR004330 (FAR1 DNA binding domain) |
| Aradu.DTV4K | 0.02 | uncharacterized protein LOC102668940 [Glycine max] |
| Aradu.DV9QN | 0.02 | Unknown protein |
| Aradu.E34IK | 0.01 | transmembrane protein, putative |
| Aradu.E9JFH | 0.02 | uncharacterized protein LOC100794165 isoform X3 [Glycine max] |
| Aradu.ERC4X | 0.04 | Nuclear division RFT1-like protein n=1 Tax=Medicago truncatula RepID=G7L7L6_MEDTR |
| Aradu.ETT8A | 0.01 | Pentatricopeptide repeat (PPR) superfamily protein; IPR002885 (Pentatricopeptide repeat) |
| Aradu.EX900 | 0.02 | uncharacterized protein LOC100776940 isoform X3 [Glycine max] |
| Aradu.F018C | 0.02 | BTB/POZ domain-containing protein; IPR008974 (TRAF-like), IPR011333 (BTB/POZ fold), IPR011705 (BTB/Kelch-associated); GO:0005515 (protein binding) |
| Aradu.F0J8L | 0.02 | transmembrane protein, putative |
| Aradu.F0X60 | 0.01 | uncharacterized protein LOC100797259 isoform X3 [Glycine max]; IPR004332 (Transposase, MuDR, plant), IPR007527 (Zinc finger, SWIM-type); GO:0008270 (zinc ion binding) |
| Aradu.FCF8I | 0.03 | NADH-ubiquinone oxidoreductase chain n=309 Tax=Magnoliophyta RepID=G7IB69_MEDTR; IPR003945 (NADH-plastoquinone oxidoreductase, chain 5); GO:0008137 (NADH dehydrogenase (ubiquinone) activity), GO:0042773 (ATP synthesis coupled electron transport), GO:0055114 (oxidation-reduction process) |
| Aradu.FI0GE | 0.01 | receptor-like kinase |
| Aradu.G0GR3 | 0.01 | uncharacterized protein LOC100820019 isoform X4 [Glycine max] |
| Aradu.G1698 | 0.01 | uncharacterized protein LOC102666122 [Glycine max] |
| Aradu.G2MK0 | 0.02 | CTV.20 n=1 Tax=Poncirus trifoliata RepID=Q8H6Q8_PONTR |
| Aradu.GFS4B | 0.01 | myb transcription factor; IPR009057 (Homeodomain-like); GO:0003677 (DNA binding), GO:0003682 (chromatin binding) |
| Aradu.GJY1F | 0.01 | Unknown protein |
| Aradu.GP0UW | 0.42 | uncharacterized protein LOC100789932 isoform X1 [Glycine max] |
| Aradu.GQ3IU | 0.01 | uncharacterized protein LOC100800865 isoform X3 [Glycine max]; IPR004332 (Transposase, MuDR, plant) |
| Aradu.GS201 | 0.01 | Unknown protein |
| Aradu.GW63C | 0.11 | Unknown protein |
| Aradu.GZ89R | 0.01 | Unknown protein |
| Aradu.H5ZX3 | 0.13 | uncharacterized protein LOC102668394 [Glycine max] |
| Aradu.H7D8L | 0.04 | protein FAR1-RELATED SEQUENCE 6-like isoform X4 [Glycine max]; IPR004330 (FAR1 DNA binding domain) |
| Aradu.H8VWB | 0.01 | uncharacterized protein LOC102666745 [Glycine max] |
| Aradu.HI1V8 | 0.01 | JHL23C09.7 protein n=1 Tax=Jatropha curcas RepID=E6NUA1_JATCU |
| Aradu.HK8V7 | 0.01 | uncharacterized protein LOC102662489 isoform X1 [Glycine max] |
| Aradu.HT9MM | 0.05 | ribosomal protein S19; IPR002222 (Ribosomal protein S19/S15), IPR023575 (Ribosomal protein S19, superfamily); GO:0003735 (structural constituent of ribosome), GO:0005840 (ribosome), GO:0006412 (translation) |
| Aradu.HTZ83 | 0.01 | Unknown protein |
| Aradu.HVD1R | 0.01 | double-stranded-RNA-binding protein 4; IPR014720 (Double-stranded RNA-binding domain) |
| Aradu.HWR8R | 0.01 | protein n=1 Tax=Oryza sativa subsp. japonica RepID=Q0JG27_ORYSJ; IPR003653 (Peptidase C48, SUMO/Sentrin/Ubl1); GO:0006508 (proteolysis), GO:0008234 (cysteine-type peptidase activity) |
| Aradu.I722Z | 0.11 | Zinc finger GRF-type protein n=1 Tax=Arachis hypogaea RepID=G0Y6U2_ARAHY |
| Aradu.I93LB | 0.01 | SET domain protein |
| Aradu.IJL0M | 0.03 | Unknown protein |
| Aradu.J4F74 | 0.01 | uncharacterized protein LOC100797259 isoform X3 [Glycine max]; IPR004332 (Transposase, MuDR, plant) |
| Aradu.J75KM | 0.01 | RNA polymerase II, Rpb4, core protein |
| Aradu.J86YI | 0.01 | serine/threonine-protein phosphatase 7 long form homolog [Glycine max]; IPR019557 (Aminotransferase-like, plant mobile domain) |
| Aradu.JCG7S | 0.02 | E3 ubiquitin-protein ligase n=5 Tax=Solanum RepID=M1BDZ1_SOLTU |
| Aradu.JL67Z | 0.01 | ammonium transporter 1 member 1 [Glycine max]; IPR024041 (Ammonium transporter AmtB-like domain); GO:0008519 (ammonium transmembrane transporter activity), GO:0015696 (ammonium transport), GO:0016020 (membrane) |
| Aradu.JN1SS | 0.01 | Unknown protein |
| Aradu.JN9JX | 0.02 | 30S ribosomal protein S15; IPR000589 (Ribosomal protein S15); GO:0003735 (structural constituent of ribosome), GO:0005622 (intracellular), GO:0005840 (ribosome), GO:0006412 (translation) |
| Aradu.JNP3M | 0.02 | glucosidase II beta subunit-like protein |
| Aradu.JQG3M | 0.01 | basic helix-loop-helix (bHLH) DNA-binding superfamily protein; IPR025610 (Transcription factor MYC/MYB N-terminal) |
| Aradu.JS0TU | 0.11 | Unknown protein |
| Aradu.K82FP | 0.02 | Unknown protein |
| Aradu.KC0H7 | 0.01 | TdcA1-ORF1-ORF2 protein n=1 Tax=Medicago truncatula RepID=G7IGM2_MEDTR |
| Aradu.KJ4ZH | 0.01 | ribosomal protein L18; IPR000039 (Ribosomal protein L18e); GO:0003735 (structural constituent of ribosome), GO:0005622 (intracellular), GO:0005840 (ribosome), GO:0006412 (translation) |
| Aradu.KKS6I | 0.01 | E3 ubiquitin-protein ligase n=5 Tax=Solanum RepID=M1BDZ1_SOLTU |
| Aradu.L3NMB | 0.1 | Zinc finger GRF-type protein n=1 Tax=Arachis hypogaea RepID=G0Y6U2_ARAHY; IPR010666 (Zinc finger, GRF-type); GO:0008270 (zinc ion binding) |
| Aradu.L8UXN | 0.07 | Unknown protein |
| Aradu.LT4XX | 0.03 | NADH-ubiquinone oxidoreductase chain n=309 Tax=Magnoliophyta RepID=G7IB69_MEDTR; IPR003945 (NADH-plastoquinone oxidoreductase, chain 5); GO:0008137 (NADH dehydrogenase (ubiquinone) activity), GO:0042773 (ATP synthesis coupled electron transport), GO:0055114 (oxidation-reduction process) |
| Aradu.M0B59 | 0.08 | JHL23C09.8 protein n=1 Tax=Jatropha curcas RepID=E6NUA2_JATCU |
| Aradu.M2D2D | 0.01 | Unknown protein; IPR003653 (Peptidase C48, SUMO/Sentrin/Ubl1); GO:0006508 (proteolysis), GO:0008234 (cysteine-type peptidase activity) |
| Aradu.MI7GZ | 0.01 | Unknown protein |
| Aradu.ML4Y0 | 0.01 | uncharacterized protein LOC100776940 isoform X3 [Glycine max]; IPR004332 (Transposase, MuDR, plant), IPR007527 (Zinc finger, SWIM-type); GO:0008270 (zinc ion binding) |
| Aradu.MP1IM | 0.05 | Unknown protein |
| Aradu.MP46F | 0.01 | Transposon protein n=1 Tax=Arachis hypogaea RepID=G0Y6V7_ARAHY |
| Aradu.MZ2PS | 0.05 | uncharacterized protein LOC100797101 [Glycine max] |
| Aradu.N34YY | 0.02 | hypothetical protein |
| Aradu.N3VMQ | 0.01 | FAR-RED impaired response-like protein; IPR004330 (FAR1 DNA binding domain) |
| Aradu.N9ISK | 0.01 | phosphoenolpyruvate carboxylase 2 |
| Aradu.NL435 | 0.03 | protein n=1 Tax=Oryza sativa subsp. japonica RepID=C7J9W8_ORYSJ |
| Aradu.NQG0Q | 0.02 | Unknown protein |
| Aradu.P5YBM | 0.02 | Helicase-like protein n=1 Tax=Medicago truncatula RepID=G7J5V9_MEDTR; IPR025476 (Helitron helicase-like domain) |
| Aradu.PH7SK | 0.03 | Unknown protein |
| Aradu.PHA3J | 0.01 | viral movement protein; IPR001878 (Zinc finger, CCHC-type); GO:0003676 (nucleic acid binding), GO:0008270 (zinc ion binding) |
| Aradu.Q011E | 0.01 | uncharacterized protein LOC100776940 isoform X3 [Glycine max]; IPR007527 (Zinc finger, SWIM-type); GO:0008270 (zinc ion binding) |
| Aradu.Q20L5 | 0.04 | DNA polymerase III subunit gamma/tau |
| Aradu.QCH32 | 0.24 | Unknown protein |
| Aradu.QFN6G | 0.04 | Pentatricopeptide repeat (PPR-like) superfamily protein; IPR002885 (Pentatricopeptide repeat) |
| Aradu.QG4E2 | 0.01 | protein FAR1-RELATED SEQUENCE 6-like isoform X2 [Glycine max]; IPR004330 (FAR1 DNA binding domain) |
| Aradu.QS09M | 0.02 | Ulp1 protease family C-terminal catalytic domain containing protein n=1 Tax=Medicago truncatula RepID=G7L5H3_MEDTR; IPR003653 (Peptidase C48, SUMO/Sentrin/Ubl1); GO:0006508 (proteolysis), GO:0008234 (cysteine-type peptidase activity) |
| Aradu.QUC4D | 0.02 | LRR receptor-like serine/threonine-protein kinase GSO2-like [Glycine max] |
| Aradu.R2C4W | 0.01 | Unknown protein; IPR003653 (Peptidase C48, SUMO/Sentrin/Ubl1); GO:0006508 (proteolysis), GO:0008234 (cysteine-type peptidase activity) |
| Aradu.R2QCS | 0.02 | long-chain acyl-CoA synthetase 6; IPR002487 (Transcription factor, K-box); GO:0003700 (sequence-specific DNA binding transcription factor activity), GO:0005634 (nucleus) |
| Aradu.R2WCM | 0.11 | Unknown protein |
| Aradu.RDR0H | 0.05 | Unknown protein |
| Aradu.REC96 | 0.01 | Unknown protein |
| Aradu.S0327 | 0.01 | serpin-like protein; IPR000215 (Serpin family), IPR023796 (Serpin domain); GO:0005615 (extracellular space) |
| Aradu.S0HI3 | 0.06 | n=1 Tax=Oryza sativa subsp. japonica RepID=Q7F971_ORYSJ |
| Aradu.S33NT | 0.03 | Unknown protein |
| Aradu.S4246 | 0.11 | LysM domain protein; IPR018392 (LysM domain); GO:0016998 (cell wall macromolecule catabolic process) |
| Aradu.S4XX2 | 0.01 | Myb/SANT-like DNA-binding domain protein; IPR024752 (Myb/SANT-like domain) |
| Aradu.SJ8S8 | 0.02 | serine/threonine-protein phosphatase 7 long form homolog [Glycine max]; IPR019557 (Aminotransferase-like, plant mobile domain) |
| Aradu.SKI52 | 0.01 | Phosphoinositide phosphatase family protein; IPR002013 (Synaptojanin, N-terminal); GO:0042578 (phosphoric ester hydrolase activity) |
| Aradu.SMT90 | 0.01 | Unknown protein |
| Aradu.SQ35L | 0.01 | zinc finger MYM-type protein 1-like [Glycine max]; IPR006580 (Zinc finger, TTF-type), IPR025398 (Domain of unknown function DUF4371) |
| Aradu.T1YYL | 0.01 | Zinc finger GRF-type protein n=1 Tax=Arachis hypogaea RepID=G0Y6U2_ARAHY |
| Aradu.T9KLM | 0.02 | replication protein A 70 kDa DNA-binding subunit C-like [Glycine max]; IPR012340 (Nucleic acid-binding, OB-fold) |
| Aradu.TB2PX | 0.01 | FAR1 DNA-binding domain protein; IPR004330 (FAR1 DNA binding domain) |
| Aradu.TF1N7 | 0.01 | uncharacterized protein LOC100817480 [Glycine max] |
| Aradu.TWJ70 | 0.01 | E3 ubiquitin-protein ligase n=5 Tax=Solanum RepID=M1BDZ1_SOLTU |
| Aradu.UHA05 | 0.01 | Transposon protein n=1 Tax=Arachis hypogaea RepID=G0Y6V7_ARAHY |
| Aradu.UNJ6K | 0.12 | choline transporter-like protein 2-like [Glycine max]; IPR007603 (Choline transporter-like) |
| Aradu.USC1D | 0.05 | Unknown protein |
| Aradu.V7R1P | 0.02 | pentatricopeptide (PPR) repeat-containing protein |
| Aradu.VN48D | 0.01 | uncharacterized protein LOC102666784 [Glycine max]; IPR007527 (Zinc finger, SWIM-type); GO:0008270 (zinc ion binding) |
| Aradu.VU55N | 0.06 | Unknown protein |
| Aradu.W4A9H | 0.01 | Ulp1 protease family, carboxy-terminal domain protein |
| Aradu.W958P | 0.01 | Transposon protein n=1 Tax=Arachis hypogaea RepID=G0Y6V7_ARAHY |
| Aradu.WE5VH | 0.07 | Unknown protein |
| Aradu.WPS4N | 0.02 | DUF223 domain protein; IPR012340 (Nucleic acid-binding, OB-fold) |
| Aradu.WR2NE | 0.01 | zinc finger MYM-type protein 1-like [Glycine max]; IPR006580 (Zinc finger, TTF-type), IPR012337 (Ribonuclease H-like domain), IPR025398 (Domain of unknown function DUF4371); GO:0003676 (nucleic acid binding) |
| Aradu.X2F3E | 0.01 | choline monooxygenase, chloroplastic-like isoform X2 [Glycine max] |
| Aradu.X47P5 | 0.35 | ferric reduction oxidase 2; IPR013130 (Ferric reductase transmembrane component-like domain) |
| Aradu.X4TEU | 0.02 | transcription elongation factor B; IPR001232 (SKP1 component); GO:0006511 (ubiquitin-dependent protein catabolic process) |
| Aradu.XD8SQ | 0.07 | TPR repeat-containing thioredoxin TTL1-like [Glycine max]; IPR011990 (Tetratricopeptide-like helical); GO:0005515 (protein binding) |
| Aradu.XMW8A | 0.01 | alcohol dehydrogenase 1; IPR002085 (Alcohol dehydrogenase superfamily, zinc-type), IPR011032 (GroES (chaperonin 10)-like); GO:0008270 (zinc ion binding), GO:0016491 (oxidoreductase activity), GO:0055114 (oxidation-reduction process) |
| Aradu.Y16TP | 0.03 | receptor-like kinase, putative |
| Aradu.Y44CQ | 0.01 | MATE efflux family protein |
| Aradu.YB2SP | 0.01 | Unknown protein |
| Aradu.YKK9G | 0.02 | Unknown protein; IPR004320 (Protein of unknown function DUF241, plant) |
| Aradu.YNH8J | 0.01 | serine/threonine-protein phosphatase 7 long form homolog [Glycine max]; IPR019557 (Aminotransferase-like, plant mobile domain) |
| Aradu.YQ5EP | 0.03 | zinc finger MYM-type protein 1-like [Glycine max]; IPR008906 (HAT dimerisation domain, C-terminal), IPR012337 (Ribonuclease H-like domain); GO:0003676 (nucleic acid binding), GO:0046983 (protein dimerization activity) |
| Aradu.Z3QBD | 0.08 | uncharacterized protein LOC100797101 [Glycine max] |
| Aradu.ZE0WK | 0.01 | uncharacterized protein At1g04910-like isoform X1 [Glycine max]; IPR019378 (GDP-fucose protein O-fucosyltransferase) |
| Aradu.ZL3P6 | 0.01 | receptor kinase 2; IPR011009 (Protein kinase-like domain) |
| Araip.03WTF | 0.01 | Unknown protein |
| Araip.0D160 | 0.01 | putative Myb family transcription factor At1g14600-like isoform X2 [Glycine max]; IPR009057 (Homeodomain-like), IPR011989 (Armadillo-like helical); GO:0003677 (DNA binding), GO:0003682 (chromatin binding) |
| Araip.0P65D | 0.01 | uncharacterized protein LOC100797259 isoform X4 [Glycine max]; IPR001878 (Zinc finger, CCHC-type), IPR004332 (Transposase, MuDR, plant), IPR007527 (Zinc finger, SWIM-type); GO:0003676 (nucleic acid binding), GO:0008270 (zinc ion binding) |
| Araip.0S39D | 0.07 | Unknown protein |
| Araip.123EL | 0.01 | Unknown protein |
| Araip.17Z4K | 0.01 | serine/threonine-protein phosphatase 7 long form homolog [Glycine max]; IPR019557 (Aminotransferase-like, plant mobile domain) |
| Araip.19ZK8 | 0.04 | Endosomal targeting BRO1-like domain-containing protein; IPR004328 (BRO1 domain) |
| Araip.1JT8A | 0.01 | protein VAC14 homolog [Glycine max] |
| Araip.1P8MY | 0.01 | uncharacterized protein LOC100797259 isoform X3 [Glycine max]; IPR004332 (Transposase, MuDR, plant) |
| Araip.1Q0S3 | 0.02 | Unknown protein |
| Araip.1QW3D | 0.01 | lectin-receptor kinase; IPR008985 (Concanavalin A-like lectin/glucanases superfamily), IPR013320 (Concanavalin A-like lectin/glucanase, subgroup), IPR016363 (Lectin); GO:0030246 (carbohydrate binding) |
| Araip.1VF25 | 0.03 | Unknown protein |
| Araip.1X9VL | 0.02 | Unknown protein |
| Araip.203E2 | 0.01 | uncharacterized protein LOC100797101 [Glycine max] |
| Araip.25GXQ | 0.05 | Unknown protein |
| Araip.29N7Y | 0.01 | Transposon protein n=1 Tax=Arachis hypogaea RepID=G0Y6V7_ARAHY |
| Araip.2ES8K | 0.12 | Glutathione S-transferase family protein; IPR010987 (Glutathione S-transferase, C-terminal-like), IPR012336 (Thioredoxin-like fold); GO:0005515 (protein binding) |
| Araip.2K8JU | 0.01 | Transposon protein n=1 Tax=Arachis hypogaea RepID=G0Y6U6_ARAHY; IPR001878 (Zinc finger, CCHC-type); GO:0003676 (nucleic acid binding), GO:0008270 (zinc ion binding) |
| Araip.2N8DS | 0.02 | Unknown protein |
| Araip.2NT8C | 0.01 | Unknown protein |
| Araip.2R52D | 0.02 | Unknown protein |
| Araip.321HD | 0.01 | ATP binding microtubule motor family protein; IPR001752 (Kinesin, motor domain), IPR027417 (P-loop containing nucleoside triphosphate hydrolase), IPR027640 (Kinesin-like protein); GO:0003777 (microtubule motor activity), GO:0005524 (ATP binding), GO:0005871 (kinesin complex), GO:0007018 (microtubule-based movement), GO:0008017 (microtubule binding) |
| Araip.37423 | 0.01 | animal RPA1 domain protein; IPR012340 (Nucleic acid-binding, OB-fold) |
| Araip.39JN9 | 0.01 | uncharacterized protein LOC100785875 isoform X1 [Glycine max]; IPR024752 (Myb/SANT-like domain) |
| Araip.3AC4F | 0.01 | Unknown protein |
| Araip.3H844 | 0.01 | Fatty acid hydroxylase superfamily; IPR006694 (Fatty acid hydroxylase); GO:0005506 (iron ion binding), GO:0006633 (fatty acid biosynthetic process), GO:0016491 (oxidoreductase activity), GO:0055114 (oxidation-reduction process) |
| Araip.3K90H | 0.02 | Unknown protein; IPR004252 (Probable transposase, Ptta/En/Spm, plant) |
| Araip.3M7QV | 0.01 | zinc finger MYM-type protein 1-like [Glycine max]; IPR008906 (HAT dimerisation domain, C-terminal); GO:0046983 (protein dimerization activity) |
| Araip.3NV18 | 0.01 | MATE efflux family protein; IPR002528 (Multi antimicrobial extrusion protein); GO:0006855 (drug transmembrane transport), GO:0015238 (drug transmembrane transporter activity), GO:0015297 (antiporter activity), GO:0016020 (membrane), GO:0055085 (transmembrane transport) |
| Araip.3V6I8 | 0.02 | aldehyde dehydrogenase family 2 member B4, mitochondrial-like [Glycine max]; IPR016161 (Aldehyde/histidinol dehydrogenase); GO:0008152 (metabolic process), GO:0016491 (oxidoreductase activity), GO:0055114 (oxidation-reduction process) |
| Araip.3X7QK | 0.01 | lecithin:cholesterol acyltransferase family protein; IPR003386 (Lecithin:cholesterol/phospholipid:diacylglycerol acyltransferase), IPR022644 (Orn/DAP/Arg decarboxylase 2, N-terminal); GO:0003824 (catalytic activity), GO:0006629 (lipid metabolic process), GO:0008374 (O-acyltransferase activity) |
| Araip.46D9X | 0.01 | Ulp1 protease family, carboxy-terminal domain protein; IPR003653 (Peptidase C48, SUMO/Sentrin/Ubl1); GO:0006508 (proteolysis), GO:0008234 (cysteine-type peptidase activity) |
| Araip.47GXC | 0.02 | Fatty acid hydroxylase superfamily; IPR021940 (Uncharacterised domain Wax2, C-terminal) |
| Araip.4H6RM | 0.05 | probable ubiquitin-like-specific protease 2B-like isoform X2 [Glycine max] |
| Araip.4U1AF | 0.02 | UPF0261 SACE-like protein; IPR008322 (Uncharacterised protein family UPF0261) |
| Araip.4V7T8 | 0.01 | Unknown protein |
| Araip.50IMR | 0.05 | E3 ubiquitin-protein ligase n=5 Tax=Solanum RepID=M1BDZ1_SOLTU |
| Araip.50WXC | 0.01 | Zinc knuckle family protein n=1 Tax=Oryza sativa subsp. japonica RepID=H2KWL4_ORYSJ; IPR001878 (Zinc finger, CCHC-type); GO:0003676 (nucleic acid binding), GO:0008270 (zinc ion binding) |
| Araip.56GV7 | 0.01 | CTV.20 n=1 Tax=Poncirus trifoliata RepID=Q8H6Q8_PONTR |
| Araip.57QZ1 | 0.01 | Unknown protein |
| Araip.5J0LW | 0.02 | LRR receptor-like kinase |
| Araip.5NT6K | 0.01 | protein FAR1-RELATED SEQUENCE 6-like isoform X2 [Glycine max]; IPR004330 (FAR1 DNA binding domain), IPR007527 (Zinc finger, SWIM-type); GO:0008270 (zinc ion binding) |
| Araip.5SL6S | 0.02 | receptor like protein 47 |
| Araip.5WL8Q | 0.01 | protein disulfide isomerase (PDI)-like protein; IPR012336 (Thioredoxin-like fold) |
| Araip.5YN1M | 0.12 | protein n=1 Tax=Oryza sativa subsp. japonica RepID=C7J586_ORYSJ; IPR019557 (Aminotransferase-like, plant mobile domain) |
| Araip.60FYC | 0.02 | Retrotransposon gag protein n=1 Tax=Arachis hypogaea RepID=G0Y6U4_ARAHY |
| Araip.67SHA | 0.02 | Unknown protein |
| Araip.68Y5F | 0.02 | clustered mitochondria protein-like isoform X2 [Glycine max] |
| Araip.6H4W6 | 0.03 | Unknown protein |
| Araip.6T61F | 0.03 | Ulp1 protease family, carboxy-terminal domain protein |
| Araip.6Y957 | 0.01 | zinc finger MYM-type protein 1-like [Glycine max]; IPR008906 (HAT dimerisation domain, C-terminal), IPR012337 (Ribonuclease H-like domain); GO:0003676 (nucleic acid binding), GO:0046983 (protein dimerization activity) |
| Araip.72TSZ | 0.23 | blue copper protein-like [Glycine max]; IPR008972 (Cupredoxin); GO:0005507 (copper ion binding), GO:0009055 (electron carrier activity) |
| Araip.76H2I | 0.01 | receptor-like protein kinase 4; IPR011009 (Protein kinase-like domain), IPR013320 (Concanavalin A-like lectin/glucanase, subgroup); GO:0004672 (protein kinase activity), GO:0005524 (ATP binding), GO:0006468 (protein phosphorylation) |
| Araip.7E1UN | 0.02 | zinc finger MYM-type protein 1-like [Glycine max]; IPR008906 (HAT dimerisation domain, C-terminal), IPR012337 (Ribonuclease H-like domain); GO:0003676 (nucleic acid binding), GO:0046983 (protein dimerization activity) |
| Araip.7TJ7Y | 0.01 | Unknown protein |
| Araip.7VA2Q | 0.02 | serine/threonine-protein phosphatase 7 long form homolog [Glycine max]; IPR019557 (Aminotransferase-like, plant mobile domain) |
| Araip.80FFF | 0.02 | Unknown protein |
| Araip.80VZY | 0.01 | serine/threonine-protein phosphatase 7 long form homolog [Glycine max]; IPR019557 (Aminotransferase-like, plant mobile domain) |
| Araip.83X32 | 0.04 | Protein disulfide isomerase (PDI)-like protein 2 n=2 Tax=Cucumis RepID=Q5DMX0_CUCME; IPR012336 (Thioredoxin-like fold) |
| Araip.86CIW | 0.01 | disease resistance protein (CC-NBS-LRR class) family protein |
| Araip.8H51Y | 0.01 | protein FAR-RED IMPAIRED RESPONSE 1-like [Glycine max] |
| Araip.8JT0Y | 0.01 | protein FAR1-RELATED SEQUENCE 6-like isoform X2 [Glycine max]; IPR004330 (FAR1 DNA binding domain), IPR006564 (Zinc finger, PMZ-type); GO:0008270 (zinc ion binding) |
| Araip.8QR0J | 0.02 | Unknown protein |
| Araip.8Y6B9 | 0.01 | Unknown protein |
| Araip.8Z9LN | 0.04 | Unknown protein |
| Araip.95VTL | 0.03 | Unknown protein |
| Araip.98F15 | 0.02 | Unknown protein |
| Araip.9A39Y | 0.01 | GDSL-like Lipase/Acylhydrolase superfamily protein; IPR013831 (SGNH hydrolase-type esterase domain); GO:0016787 (hydrolase activity) |
| Araip.9AH94 | 0.03 | serine/threonine-protein phosphatase 7 long form homolog [Glycine max]; IPR019557 (Aminotransferase-like, plant mobile domain) |
| Araip.9BB1P | 0.05 | polyvinylalcohol dehydrogenase-like protein; IPR011047 (Quinonprotein alcohol dehydrogenase-like superfamily) |
| Araip.9P8WC | 0.01 | Unknown protein |
| Araip.9ZK9N | 0.01 | Unknown protein; IPR003653 (Peptidase C48, SUMO/Sentrin/Ubl1); GO:0006508 (proteolysis), GO:0008234 (cysteine-type peptidase activity) |
| Araip.A0B9A | 0.02 | homogentisate phytyltransferase 1; IPR000537 (UbiA prenyltransferase family); GO:0004659 (prenyltransferase activity), GO:0016021 (integral component of membrane) |
| Araip.A5K65 | 0.02 | Unknown protein |
| Araip.A5LLZ | 0.02 | GRF zinc finger protein; IPR010666 (Zinc finger, GRF-type); GO:0008270 (zinc ion binding) |
| Araip.A7ULM | 0.01 | Zinc finger GRF-type protein n=1 Tax=Arachis hypogaea RepID=G0Y6U2_ARAHY |
| Araip.AIY5J | 0.01 | serine/threonine-protein phosphatase 7 long form homolog [Glycine max]; IPR019557 (Aminotransferase-like, plant mobile domain) |
| Araip.AKG7U | 0.01 | Unknown protein |
| Araip.AL7QR | 0.01 | Unknown protein |
| Araip.AMF5I | 0.02 | Unknown protein |
| Araip.AN4S6 | 0.01 | serine/threonine-protein phosphatase 7 long form homolog [Glycine max]; IPR019557 (Aminotransferase-like, plant mobile domain) |
| Araip.AR0YG | 0.03 | Ulp1 protease family, carboxy-terminal domain protein; IPR003653 (Peptidase C48, SUMO/Sentrin/Ubl1); GO:0006508 (proteolysis), GO:0008234 (cysteine-type peptidase activity) |
| Araip.AVN7S | 0.01 | serine/threonine-protein phosphatase 7 long form homolog [Glycine max]; IPR019557 (Aminotransferase-like, plant mobile domain) |
| Araip.B2UEI | 0.01 | Unknown protein |
| Araip.B8YA7 | 0.03 | Unknown protein |
| Araip.BE53N | 0.01 | GRF zinc finger protein; IPR010666 (Zinc finger, GRF-type); GO:0008270 (zinc ion binding) |
| Araip.BI4QJ | 0.01 | E3 ubiquitin-protein ligase n=5 Tax=Solanum RepID=M1BDZ1_SOLTU |
| Araip.BKD5D | 0.02 | Unknown protein |
| Araip.BNZ9Z | 0.01 | Unknown protein |
| Araip.BS49C | 0.01 | serine/threonine-protein phosphatase 7 long form homolog [Glycine max]; IPR019557 (Aminotransferase-like, plant mobile domain) |
| Araip.C0JQU | 0.02 | ubiquitin fusion degradation 1; IPR004854 (Ubiquitin fusion degradation protein UFD1); GO:0006511 (ubiquitin-dependent protein catabolic process) |
| Araip.C2IXB | 0.01 | Unknown protein |
| Araip.C3AIS | 0.01 | Unknown protein |
| Araip.C643G | 0.01 | E3 ubiquitin-protein ligase n=5 Tax=Solanum RepID=M1BDZ1_SOLTU |
| Araip.C86ZQ | 0.01 | Unknown protein |
| Araip.CEW5G | 0.02 | Unknown protein |
| Araip.CH8F6 | 0.01 | ribonucleoside-diphosphate reductase; IPR000788 (Ribonucleotide reductase large subunit, C-terminal); GO:0006260 (DNA replication), GO:0055114 (oxidation-reduction process) |
| Araip.CIY2B | 0.01 | n=2 Tax=Oryza sativa subsp. japonica RepID=Q7XS42_ORYSJ; IPR003653 (Peptidase C48, SUMO/Sentrin/Ubl1); GO:0006508 (proteolysis), GO:0008234 (cysteine-type peptidase activity) |
| Araip.CSI7Z | 0.04 | Unknown protein |
| Araip.CU2VF | 0.01 | Cold acclimation protein COR413-TM1 n=2 Tax=Medicago truncatula RepID=G7L817_MEDTR |
| Araip.CWZ66 | 0.05 | Unknown protein |
| Araip.CX9E5 | 0.01 | protein disulfide isomerase (PDI)-like protein; IPR012336 (Thioredoxin-like fold) |
| Araip.D71AK | 0.03 | Unknown protein |
| Araip.D77Z3 | 0.01 | uncharacterized protein LOC100800865 isoform X3 [Glycine max]; IPR001878 (Zinc finger, CCHC-type), IPR004332 (Transposase, MuDR, plant), IPR007527 (Zinc finger, SWIM-type); GO:0003676 (nucleic acid binding), GO:0008270 (zinc ion binding) |
| Araip.DBT6L | 0.02 | xylose isomerase family protein; IPR013022 (Xylose isomerase-like, TIM barrel domain) |
| Araip.DF1A3 | 0.01 | thioredoxin X; IPR005746 (Thioredoxin), IPR012336 (Thioredoxin-like fold); GO:0006662 (glycerol ether metabolic process), GO:0015035 (protein disulfide oxidoreductase activity), GO:0045454 (cell redox homeostasis) |
| Araip.E0G45 | 0.01 | Unknown protein |
| Araip.E21DU | 0.05 | Reverse transcriptase n=3 Tax=Dahlia mosaic virus RepID=T1SGN7_9VIRU |
| Araip.E6Q02 | 0.01 | transmembrane protein, putative |
| Araip.EE6SM | 0.03 | receptor like protein 15; IPR001611 (Leucine-rich repeat); GO:0005515 (protein binding) |
| Araip.EIY8C | 0.23 | cysteine-rich receptor-kinase-like protein; IPR002902 (Gnk2-homologous domain) |
| Araip.F0GR2 | 0.01 | uncharacterized protein LOC100805683 [Glycine max]; IPR025312 (Domain of unknown function DUF4216) |
| Araip.F0H2W | 0.01 | probable sphingolipid transporter spinster homolog 2-like isoform X2 [Glycine max] |
| Araip.F1MPF | 0.13 | bromo-adjacent homology (BAH) domain-containing protein |
| Araip.F45US | 0.01 | n=1 Tax=Oryza sativa subsp. japonica RepID=Q7XSJ2_ORYSJ; IPR004332 (Transposase, MuDR, plant) |
| Araip.FL897 | 0.01 | Helicase-like protein n=1 Tax=Medicago truncatula RepID=G7KPQ9_MEDTR; IPR025476 (Helitron helicase-like domain) |
| Araip.FMM6X | 0.01 | Unknown protein |
| Araip.FPJ3C | 0.01 | protein FAR1-RELATED SEQUENCE 6-like isoform X2 [Glycine max]; IPR004330 (FAR1 DNA binding domain), IPR007527 (Zinc finger, SWIM-type); GO:0008270 (zinc ion binding) |
| Araip.FV3M5 | 0.04 | pectinesterase 11; IPR011050 (Pectin lyase fold/virulence factor); GO:0005618 (cell wall), GO:0030599 (pectinesterase activity), GO:0042545 (cell wall modification) |
| Araip.FY1L9 | 0.07 | Unknown protein |
| Araip.G7S11 | 2.02 | unknown protein; FUNCTIONS IN: molecular_function unknown; INVOLVED IN: biological_process unknown; LOCATED IN: endomembrane system |
| Araip.GE45V | 0.06 | SAC3/GANP/Nin1/mts3/eIF-3 p25 family |
| Araip.GK0VH | 0.01 | Unknown protein |
| Araip.GLN5F | 0.01 | brefeldin A-inhibited guanine nucleotide-exchange protein 5-like isoform X1 [Glycine max] |
| Araip.H34GT | 0.02 | CTV.20 n=1 Tax=Poncirus trifoliata RepID=Q8H6Q8_PONTR |
| Araip.H64P3 | 0.02 | ubiquitin-protein ligase, cullin 4; IPR011991 (Winged helix-turn-helix DNA-binding domain); GO:0006511 (ubiquitin-dependent protein catabolic process), GO:0031461 (cullin-RING ubiquitin ligase complex), GO:0031625 (ubiquitin protein ligase binding) |
| Araip.H7Y1U | 0.01 | plasma membrane H+-ATPase; IPR001757 (Cation-transporting P-type ATPase), IPR023214 (HAD-like domain), IPR023298 (P-type ATPase, transmembrane domain); GO:0006812 (cation transport), GO:0016021 (integral component of membrane), GO:0019829 (cation-transporting ATPase activity) |
| Araip.HM1IB | 0.01 | protein FAR1-RELATED SEQUENCE 9-like isoform X5 [Glycine max]; IPR001878 (Zinc finger, CCHC-type); GO:0003676 (nucleic acid binding), GO:0008270 (zinc ion binding) |
| Araip.HM85T | 0.01 | Unknown protein |
| Araip.HUG3D | 0.02 | calmodulin-binding transcription activator; IPR005559 (CG-1 DNA-binding domain); GO:0003677 (DNA binding), GO:0005634 (nucleus) |
| Araip.HYA7N | 0.02 | Unknown protein |
| Araip.I0IIG | 0.01 | zinc finger MYM-type protein 1-like [Glycine max]; IPR008906 (HAT dimerisation domain, C-terminal); GO:0046983 (protein dimerization activity) |
| Araip.I55GG | 0.01 | GRF zinc finger protein; IPR010666 (Zinc finger, GRF-type); GO:0008270 (zinc ion binding) |
| Araip.I75UX | 0.01 | disease resistance protein (TIR-NBS-LRR class), putative; IPR000767 (Disease resistance protein), IPR027417 (P-loop containing nucleoside triphosphate hydrolase); GO:0006952 (defense response), GO:0043531 (ADP binding) |
| Araip.I7LNN | 0.03 | Pentatricopeptide repeat (PPR) superfamily protein |
| Araip.IH2Y7 | 0.01 | DNA ligase IV |
| Araip.IH553 | 0.01 | starch branching enzyme I; IPR013783 (Immunoglobulin-like fold), IPR014756 (Immunoglobulin E-set), IPR015902 (Glycoside hydrolase, family 13); GO:0003824 (catalytic activity), GO:0005975 (carbohydrate metabolic process) |
| Araip.IQ0FG | 0.01 | Unknown protein; IPR010666 (Zinc finger, GRF-type); GO:0008270 (zinc ion binding) |
| Araip.IQP0W | 0.01 | Unknown protein |
| Araip.IR6Z3 | 0.01 | Unknown protein |
| Araip.IY8RW | 0.01 | protein FAR1-RELATED SEQUENCE 6-like isoform X2 [Glycine max]; IPR007527 (Zinc finger, SWIM-type); GO:0008270 (zinc ion binding) |
| Araip.J2Y8Y | 0.03 | LRR receptor-like kinase family protein; IPR001611 (Leucine-rich repeat); GO:0005515 (protein binding) |
| Araip.J44GD | 0.03 | serine/threonine-protein phosphatase 7 long form homolog [Glycine max]; IPR019557 (Aminotransferase-like, plant mobile domain) |
| Araip.J87L2 | 0.02 | Unknown protein |
| Araip.JEU5K | 0.01 | WRKY transcription factor |
| Araip.JH4AN | 0.02 | Unknown protein |
| Araip.JHF73 | 0.01 | Transposon protein n=1 Tax=Arachis hypogaea RepID=G0Y6V7_ARAHY |
| Araip.JU31M | 0.04 | Unknown protein; IPR003653 (Peptidase C48, SUMO/Sentrin/Ubl1); GO:0006508 (proteolysis), GO:0008234 (cysteine-type peptidase activity) |
| Araip.K5EBH | 0.01 | protein FAR1-RELATED SEQUENCE 6-like isoform X2 [Glycine max]; IPR006564 (Zinc finger, PMZ-type); GO:0008270 (zinc ion binding) |
| Araip.K95II | 0.07 | UDP-glucosyltransferase family protein; IPR002213 (UDP-glucuronosyl/UDP-glucosyltransferase); GO:0008152 (metabolic process) |
| Araip.K9TUR | 0.07 | DNA-directed RNA polymerase subunit beta; IPR007642 (RNA polymerase Rpb2, domain 2), IPR015712 (DNA-directed RNA polymerase, subunit 2); GO:0003677 (DNA binding), GO:0003899 (DNA-directed RNA polymerase activity), GO:0032549 (ribonucleoside binding) |
| Araip.KK1HC | 0.01 | uncharacterized protein LOC100796221 [Glycine max]; IPR004252 (Probable transposase, Ptta/En/Spm, plant) |
| Araip.KKA0A | 0.01 | subtilisin-like protease-like [Glycine max]; IPR003137 (Protease-associated domain, PA) |
| Araip.KRT2F | 0.01 | CTV.20, putative n=1 Tax=Solanum tuberosum RepID=Q60CX3_SOLTU |
| Araip.KY12D | 0.01 | octicosapeptide/phox/Bem1p domain protein |
| Araip.L5DYK | 0.02 | uncharacterized protein LOC102666104 [Glycine max] |
| Araip.L5M22 | 0.01 | Unknown protein |
| Araip.LM0RV | 0.02 | unknown protein DS12 from 2D-PAGE of leaf, chloroplastic-like [Glycine max]; IPR020810 (Enolase, C-terminal); GO:0000015 (phosphopyruvate hydratase complex), GO:0000287 (magnesium ion binding), GO:0004634 (phosphopyruvate hydratase activity), GO:0006096 (glycolysis) |
| Araip.LN5XD | 0.01 | cyclin-dependent kinase B1; 2; IPR011009 (Protein kinase-like domain) |
| Araip.LPD4W | 0.01 | receptor-like protein kinase 2; IPR001611 (Leucine-rich repeat); GO:0005515 (protein binding) |
| Araip.LU1J2 | 0.01 | Unknown protein |
| Araip.M0YRU | 0.07 | Unknown protein |
| Araip.M39BZ | 0.01 | Unknown protein |
| Araip.M3UCT | 0.02 | Transposon protein n=1 Tax=Arachis hypogaea RepID=G0Y6V7_ARAHY |
| Araip.M4P5P | 0.01 | Unknown protein |
| Araip.M4TY9 | 0.01 | Protein of unknown function (DUF594); IPR007658 (Protein of unknown function DUF594), IPR025315 (Domain of unknown function DUF4220) |
| Araip.M5E56 | 0.03 | Unknown protein |
| Araip.M5VFQ | 0.01 | protein FAR1-RELATED SEQUENCE 6-like isoform X2 [Glycine max]; IPR007527 (Zinc finger, SWIM-type); GO:0008270 (zinc ion binding) |
| Araip.M94TQ | 0.01 | serine/threonine-protein phosphatase 7 long form homolog [Glycine max] |
| Araip.MAU9L | 0.01 | uncharacterized protein LOC100776940 isoform X3 [Glycine max]; IPR004332 (Transposase, MuDR, plant) |
| Araip.MB1S8 | 0.01 | E3 ubiquitin-protein ligase n=5 Tax=Solanum RepID=M1BDZ1_SOLTU |
| Araip.ME042 | 0.01 | AP2-like ethylene-responsive transcription factor BBM2-like [Glycine max] |
| Araip.MGF6M | 0.03 | LOB domain-containing protein 33; IPR004883 (Lateral organ boundaries, LOB) |
| Araip.MNP2R | 0.01 | zinc finger MYM-type protein 1-like [Glycine max]; IPR006580 (Zinc finger, TTF-type), IPR025398 (Domain of unknown function DUF4371) |
| Araip.MQ6S8 | 0.01 | GRF zinc finger protein; IPR010666 (Zinc finger, GRF-type); GO:0008270 (zinc ion binding) |
| Araip.MU5XW | 0.02 | Unknown protein |
| Araip.N9VWI | 0.01 | serine/threonine-protein phosphatase 7 long form homolog [Glycine max]; IPR019557 (Aminotransferase-like, plant mobile domain) |
| Araip.NHA91 | 0.02 | Unknown protein |
| Araip.NPY9F | 0.02 | uncharacterized protein LOC100797259 isoform X3 [Glycine max]; IPR004332 (Transposase, MuDR, plant), IPR007527 (Zinc finger, SWIM-type); GO:0008270 (zinc ion binding) |
| Araip.NV0Q4 | 0.01 | Zinc knuckle family protein n=1 Tax=Oryza sativa subsp. japonica RepID=H2KW38_ORYSJ |
| Araip.P1JWX | 0.01 | Unknown protein |
| Araip.P5D8L | 0.01 | Unknown protein; IPR001878 (Zinc finger, CCHC-type); GO:0003676 (nucleic acid binding), GO:0008270 (zinc ion binding) |
| Araip.P8G1A | 0.01 | E3 ubiquitin-protein ligase n=5 Tax=Solanum RepID=M1BDZ1_SOLTU |
| Araip.PBZ1G | 0.01 | DNA ligase IV |
| Araip.PK7XM | 0.01 | Unknown protein |
| Araip.PTD4G | 0.01 | Helicase-like protein n=1 Tax=Medicago truncatula RepID=G7JN83_MEDTR; IPR025476 (Helitron helicase-like domain) |
| Araip.PUG95 | 0.02 | UDP-Glycosyltransferase superfamily protein; IPR002213 (UDP-glucuronosyl/UDP-glucosyltransferase); GO:0008152 (metabolic process) |
| Araip.PY3HK | 0.01 | DUF223 domain protein; IPR012340 (Nucleic acid-binding, OB-fold) |
| Araip.Q2RSY | 0.01 | Unknown protein |
| Araip.Q2RZ8 | 0.08 | Zinc finger MYM-type protein n=1 Tax=Medicago truncatula RepID=G7IY07_MEDTR |
| Araip.Q64T1 | 0.02 | pre-mRNA-splicing factor SF2-like protein; IPR012677 (Nucleotide-binding, alpha-beta plait); GO:0000166 (nucleotide binding) |
| Araip.QHJ6J | 0.01 | uncharacterized protein LOC102664163 isoform X5 [Glycine max]; IPR004252 (Probable transposase, Ptta/En/Spm, plant) |
| Araip.QQ5Y5 | 0.03 | serine hydroxymethyltransferase 2 |
| Araip.RTX2B | 0.01 | uncharacterized protein At2g29880-like [Glycine max]; IPR024752 (Myb/SANT-like domain) |
| Araip.RV5A3 | 0.02 | serine/threonine-protein phosphatase 7 long form homolog [Glycine max]; IPR019557 (Aminotransferase-like, plant mobile domain) |
| Araip.S14AG | 0.07 | transcription elongation factor (TFIIS) family protein |
| Araip.S2J45 | 0.01 | protein FAR1-RELATED SEQUENCE 6-like isoform X2 [Glycine max]; IPR004330 (FAR1 DNA binding domain), IPR007527 (Zinc finger, SWIM-type); GO:0008270 (zinc ion binding) |
| Araip.S4KIF | 0.02 | zinc finger MYM-type protein 1-like [Glycine max] |
| Araip.S56LG | 0.08 | DNA polymerase n=1 Tax=Silene vulgaris RepID=U5HST1_SILVU; IPR012337 (Ribonuclease H-like domain); GO:0003676 (nucleic acid binding) |
| Araip.S5HFC | 0.01 | GRF zinc finger protein; IPR010666 (Zinc finger, GRF-type); GO:0008270 (zinc ion binding) |
| Araip.S7CN9 | 0.01 | protein FAR1-RELATED SEQUENCE 5-like [Glycine max]; IPR001878 (Zinc finger, CCHC-type); GO:0003676 (nucleic acid binding), GO:0008270 (zinc ion binding) |
| Araip.SA484 | 0.01 | Helicase-like protein n=1 Tax=Medicago truncatula RepID=G7JN81_MEDTR |
| Araip.SKG82 | 0.01 | serine/threonine-protein phosphatase 7 long form homolog [Glycine max]; IPR019557 (Aminotransferase-like, plant mobile domain) |
| Araip.TC7SJ | 0.01 | IQ-domain 33; IPR000048 (IQ motif, EF-hand binding site); GO:0005515 (protein binding) |
| Araip.TE12R | 0.01 | uncharacterized protein LOC102660738 isoform X3 [Glycine max] |
| Araip.TF6DK | 0.02 | phosphatidylinositol- 4-phosphate 5-kinase 5; IPR003409 (MORN motif) |
| Araip.TG277 | 0.01 | Zinc knuckle family protein n=1 Tax=Solanum demissum RepID=Q6L424_SOLDE |
| Araip.TLL5M | 0.08 | Unknown protein |
| Araip.U0498 | 0.21 | carotenoid 9,10(9',10')-cleavage dioxygenase-like isoform X1 [Glycine max]; IPR004294 (Carotenoid oxygenase) |
| Araip.U20S9 | 0.01 | phosphatidylinositol 3,4,5-trisphosphate 3-phosphatase and dual-specificity protein phosphatase PTEN-like [Glycine max]; IPR023395 (Mitochondrial carrier domain) |
| Araip.U5K69 | 0.01 | Unknown protein; IPR012340 (Nucleic acid-binding, OB-fold) |
| Araip.U9IX6 | 0.01 | protein FAR1-RELATED SEQUENCE 6-like isoform X2 [Glycine max]; IPR007527 (Zinc finger, SWIM-type); GO:0008270 (zinc ion binding) |
| Araip.UNX5Q | 0.01 | germin-like protein 2; IPR001929 (Germin); GO:0030145 (manganese ion binding), GO:0045735 (nutrient reservoir activity) |
| Araip.UQ6QH | 0.03 | dehydroascorbate reductase 1; IPR010987 (Glutathione S-transferase, C-terminal-like) |
| Araip.UU7KH | 0.01 | Unknown protein |
| Araip.V36MZ | 0.01 | Unknown protein; IPR009027 (Ribosomal protein L9/RNase H1, N-terminal) |
| Araip.V6GJM | 0.01 | Unknown protein |
| Araip.V6LFF | 0.04 | peroxidase family protein; IPR010255 (Haem peroxidase); GO:0004601 (peroxidase activity), GO:0006979 (response to oxidative stress), GO:0020037 (heme binding), GO:0055114 (oxidation-reduction process) |
| Araip.V6R80 | 0.01 | nodulin MtN21 /EamA-like transporter family protein; IPR000620 (Drug/metabolite transporter); GO:0016020 (membrane) |
| Araip.V8P4D | 0.01 | serine/threonine-protein phosphatase 7 long form homolog isoform X2 [Glycine max]; IPR003653 (Peptidase C48, SUMO/Sentrin/Ubl1); GO:0006508 (proteolysis), GO:0008234 (cysteine-type peptidase activity) |
| Araip.VB2WC | 0.01 | serine/threonine-protein phosphatase 7 long form homolog [Glycine max]; IPR019557 (Aminotransferase-like, plant mobile domain) |
| Araip.VEQ25 | 0.01 | uncharacterized protein LOC100814154 isoform X1 [Glycine max]; IPR013666 (Pleckstrin-like, plant) |
| Araip.VF6IF | 0.03 | Unknown protein |
| Araip.VW11I | 0.01 | Unknown protein |
| Araip.W0H6M | 0.01 | putative ubiquitin-like-specific protease 1B-like isoform X2 [Glycine max]; IPR003653 (Peptidase C48, SUMO/Sentrin/Ubl1); GO:0006508 (proteolysis), GO:0008234 (cysteine-type peptidase activity) |
| Araip.W331D | 0.01 | receptor-like protein kinase 2; IPR001611 (Leucine-rich repeat); GO:0005515 (protein binding) |
| Araip.W7P1V | 0.01 | uncharacterized protein LOC100805509 isoform X4 [Glycine max]; IPR004252 (Probable transposase, Ptta/En/Spm, plant) |
| Araip.WA1HP | 0.02 | Zinc knuckle family protein n=1 Tax=Oryza sativa subsp. japonica RepID=H2KWL4_ORYSJ; IPR001878 (Zinc finger, CCHC-type); GO:0003676 (nucleic acid binding), GO:0008270 (zinc ion binding) |
| Araip.WD35J | 0.02 | FAR-RED impaired response-like protein, putative |
| Araip.WEQ9Q | 0.01 | uncharacterized protein LOC102659642 [Glycine max]; IPR010666 (Zinc finger, GRF-type); GO:0008270 (zinc ion binding) |
| Araip.WL58S | 0.02 | protein FAR1-RELATED SEQUENCE 6-like isoform X4 [Glycine max]; IPR004330 (FAR1 DNA binding domain) |
| Araip.WTB7G | 0.01 | ubiquitin-protein ligase, cullin 4; IPR001373 (Cullin, N-terminal); GO:0006511 (ubiquitin-dependent protein catabolic process), GO:0031461 (cullin-RING ubiquitin ligase complex), GO:0031625 (ubiquitin protein ligase binding) |
| Araip.WZ133 | 0.01 | myo-inositol oxygenase 4; IPR007828 (Inositol oxygenase); GO:0005506 (iron ion binding), GO:0005737 (cytoplasm), GO:0019310 (inositol catabolic process), GO:0050113 (inositol oxygenase activity), GO:0055114 (oxidation-reduction process) |
| Araip.X0NCM | 0.01 | Unknown protein |
| Araip.X6B1P | 0.01 | protein CASP-like [Glycine max] |
| Araip.X99F6 | 0.07 | uncharacterized GPI-anchored protein At1g61900-like isoform X4 [Glycine max] |
| Araip.X9FFV | 0.01 | uncharacterized protein LOC100785875 isoform X4 [Glycine max]; IPR024752 (Myb/SANT-like domain) |
| Araip.XL11B | 0.01 | Unknown protein; IPR003653 (Peptidase C48, SUMO/Sentrin/Ubl1); GO:0006508 (proteolysis), GO:0008234 (cysteine-type peptidase activity) |
| Araip.Y03J3 | 0.01 | hypothetical protein |
| Araip.Y5IG4 | 0.01 | hypothetical protein |
| Araip.Y65HX | 0.02 | serine/threonine-protein phosphatase 7 long form homolog [Glycine max]; IPR019557 (Aminotransferase-like, plant mobile domain) |
| Araip.YJY2F | 0.03 | serine/threonine-protein phosphatase 7 long form homolog [Glycine max]; IPR019557 (Aminotransferase-like, plant mobile domain) |
| Araip.YKJ2J | 0.02 | Unknown protein |
| Araip.YQ8CF | 0.02 | Unknown protein |
| Araip.YXX5A | 0.03 | Transposon protein n=1 Tax=Arachis hypogaea RepID=G0Y6V7_ARAHY |
| Araip.YYD48 | 0.01 | Protein kinase superfamily protein; IPR011009 (Protein kinase-like domain); GO:0004672 (protein kinase activity), GO:0005524 (ATP binding), GO:0006468 (protein phosphorylation) |
| Araip.Z5R7T | 0.01 | MYB transcription factor MYB51 [Glycine max]; IPR009057 (Homeodomain-like); GO:0003677 (DNA binding), GO:0003682 (chromatin binding) |
| Araip.Z8HBD | 0.01 | glucose-6-phosphate dehydrogenase 6; IPR001282 (Glucose-6-phosphate dehydrogenase); GO:0004345 (glucose-6-phosphate dehydrogenase activity), GO:0006006 (glucose metabolic process), GO:0050661 (NADP binding), GO:0055114 (oxidation-reduction process) |
| Araip.Z8K55 | 0.02 | GRF zinc finger protein; IPR010666 (Zinc finger, GRF-type); GO:0008270 (zinc ion binding) |
| Araip.Z8L6P | 0.02 | transcription factor bHLH7-like isoform X1 [Glycine max] |
| Araip.ZES8J | 0.05 | Unknown protein |
| Araip.ZS74A | 0.01 | serine/threonine-protein phosphatase 7 long form homolog [Glycine max]; IPR019557 (Aminotransferase-like, plant mobile domain) |
| Araip.ZVL0H | 0.01 | Unknown protein |
| Nodule |  |  |
| Aradu.0R0X2 | 0.03 | E3 ubiquitin-protein ligase n=5 Tax=Solanum RepID=M1BDZ1_SOLTU |
| Aradu.157ST | 0.01 | Quinoprotein amine dehydrogenase, beta chain-like; RIC1-like guanyl-nucleotide exchange factor |
| Aradu.16NNX | 0.19 | AMP-binding enzyme |
| Aradu.17DA9 | 0.05 | probable glycosyltransferase At5g03795-like isoform X2 [Glycine max] |
| Aradu.1D069 | 0.02 | Unknown protein |
| Aradu.1F4A0 | 0.06 | Unknown protein |
| Aradu.22KKF | 0.03 | serine/threonine-protein phosphatase 7 long form homolog [Glycine max]; IPR019557 (Aminotransferase-like, plant mobile domain) |
| Aradu.23Q7Y | 0.02 | FAR1-related sequence 10; IPR004330 (FAR1 DNA binding domain) |
| Aradu.27LTR | 0.02 | leguminosin group486 secreted peptide |
| Aradu.27WYI | 0.01 | uncharacterized protein LOC100782920 [Glycine max] |
| Aradu.2F7MX | 0.01 | uncharacterized protein LOC102661730 [Glycine max] |
| Aradu.2P0CH | 0.01 | flocculation protein FLO11-like isoform X5 [Glycine max] |
| Aradu.2PP86 | 0.01 | 2-aminoethanethiol dioxygenase-like [Glycine max]; IPR012864 (Cysteamine dioxygenase); GO:0047800 (cysteamine dioxygenase activity), GO:0055114 (oxidation-reduction process) |
| Aradu.2RD4M | 0.03 | receptor-like kinase; IPR001890 (RNA-binding, CRM domain); GO:0003723 (RNA binding) |
| Aradu.2V0TP | 0.02 | Disease resistance protein (TIR-NBS class); IPR000157 (Toll/interleukin-1 receptor homology (TIR) domain); GO:0005515 (protein binding), GO:0007165 (signal transduction) |
| Aradu.3FV9M | 0.01 | uncharacterized protein LOC100776940 isoform X4 [Glycine max]; IPR001878 (Zinc finger, CCHC-type), IPR004332 (Transposase, MuDR, plant), IPR007527 (Zinc finger, SWIM-type); GO:0003676 (nucleic acid binding), GO:0008270 (zinc ion binding) |
| Aradu.3JM3M | 0.03 | Unknown protein |
| Aradu.3K14G | 0.01 | protein FAR1-RELATED SEQUENCE 5-like [Glycine max]; IPR004330 (FAR1 DNA binding domain), IPR007527 (Zinc finger, SWIM-type); GO:0008270 (zinc ion binding) |
| Aradu.3Y77K | 0.01 | flocculation protein FLO11-like isoform X3 [Glycine max] |
| Aradu.428B5 | 0.02 | Unknown protein |
| Aradu.483IH | 0.01 | Unknown protein |
| Aradu.4F0JI | 0.02 | hypothetical protein |
| Aradu.529A4 | 0.01 | Ulp1 protease family, carboxy-terminal domain protein |
| Aradu.52QQD | 0.01 | cellulose synthase 1 |
| Aradu.53CXJ | 0.01 | Unknown protein |
| Aradu.5G0HF | 0.04 | Unknown protein |
| Aradu.5HP69 | 0.01 | Helicase-like protein n=1 Tax=Medicago truncatula RepID=G7IZZ2_MEDTR; IPR025476 (Helitron helicase-like domain) |
| Aradu.60F3E | 0.02 | Unknown protein |
| Aradu.62I6K | 0.01 | protein FAR1-RELATED SEQUENCE 6-like isoform X2 [Glycine max]; IPR004330 (FAR1 DNA binding domain), IPR007527 (Zinc finger, SWIM-type); GO:0008270 (zinc ion binding) |
| Aradu.6B025 | 0.01 | diphthamide biosynthesis protein 1-like [Glycine max]; IPR002728 (Diphthamide synthesis, DPH1/DPH2); GO:0005737 (cytoplasm), GO:0017183 (peptidyl-diphthamide biosynthetic process from peptidyl-histidine) |
| Aradu.6ES1Q | 0.01 | signal peptide peptidase; IPR007369 (Peptidase A22B, signal peptide peptidase); GO:0004190 (aspartic-type endopeptidase activity), GO:0016021 (integral component of membrane) |
| Aradu.6F8HR | 0.02 | CTV.19 n=1 Tax=Poncirus trifoliata RepID=Q8H6Q9_PONTR |
| Aradu.72IBH | 0.04 | uncharacterized protein LOC100780312 [Glycine max]; IPR007021 (Domain of unknown function DUF659), IPR012337 (Ribonuclease H-like domain); GO:0003676 (nucleic acid binding) |
| Aradu.72SVA | 0.18 | serine/threonine-protein phosphatase 7 long form homolog [Glycine max]; IPR019557 (Aminotransferase-like, plant mobile domain) |
| Aradu.77S03 | 0.02 | Unknown protein |
| Aradu.7J1H4 | 0.02 | protein n=1 Tax=Oryza sativa subsp. japonica RepID=C7J9W8_ORYSJ; IPR007527 (Zinc finger, SWIM-type); GO:0008270 (zinc ion binding) |
| Aradu.7W8ZG | 0.03 | Unknown protein |
| Aradu.7YD8P | 0.07 | Unknown protein |
| Aradu.8ZJ17 | 0.01 | protein n=1 Tax=Oryza sativa subsp. japonica RepID=C7J8U9_ORYSJ |
| Aradu.9A7HH | 0.03 | Unknown protein |
| Aradu.9AK3C | 0.01 | aspartic proteinase A1; IPR001461 (Aspartic peptidase), IPR021109 (Aspartic peptidase domain); GO:0004190 (aspartic-type endopeptidase activity), GO:0006508 (proteolysis) |
| Aradu.A2J7X | 0.01 | Unknown protein |
| Aradu.A2UTQ | 0.01 | Ulp1 protease family, carboxy-terminal domain protein; IPR003653 (Peptidase C48, SUMO/Sentrin/Ubl1); GO:0006508 (proteolysis), GO:0008234 (cysteine-type peptidase activity) |
| Aradu.A7YKY | 0.04 | phosphatidylinositol 3,4,5-trisphosphate 3-phosphatase and dual-specificity protein phosphatase PTEN-like isoform X2 [Glycine max] |
| Aradu.AD4KP | 0.01 | cycloartenol synthase 1; IPR008930 (Terpenoid cyclases/protein prenyltransferase alpha-alpha toroid); GO:0003824 (catalytic activity) |
| Aradu.AS126 | 0.09 | agamous-like MADS-box protein AGL15-like isoform X1 [Glycine max] |
| Aradu.B49MM | 0.02 | FAR-RED impaired response-like protein; IPR004330 (FAR1 DNA binding domain) |
| Aradu.B62PP | 0.01 | uncharacterized protein LOC100793937 [Glycine max] |
| Aradu.BBU9N | 0.04 | E3 ubiquitin-protein ligase n=5 Tax=Solanum RepID=M1BDZ1_SOLTU |
| Aradu.BK0D0 | 0.11 | acyl-CoA N-acyltransferase (NAT) family protein; IPR016181 (Acyl-CoA N-acyltransferase) |
| Aradu.BPI4P | 0.01 | Ulp1 protease family, carboxy-terminal domain protein; IPR003653 (Peptidase C48, SUMO/Sentrin/Ubl1); GO:0006508 (proteolysis), GO:0008234 (cysteine-type peptidase activity) |
| Aradu.C8QUK | 0.01 | Ulp1 protease family, carboxy-terminal domain protein; IPR003653 (Peptidase C48, SUMO/Sentrin/Ubl1); GO:0006508 (proteolysis), GO:0008234 (cysteine-type peptidase activity) |
| Aradu.CE9RS | 0.04 | Unknown protein |
| Aradu.CF9WQ | 0.1 | endonuclease 4; IPR008947 (Phospholipase C/P1 nuclease domain) |
| Aradu.CHW23 | 0.04 | Unknown protein |
| Aradu.CL77J | 0.01 | protein FAR1-RELATED SEQUENCE 6-like isoform X2 [Glycine max]; IPR007527 (Zinc finger, SWIM-type); GO:0008270 (zinc ion binding) |
| Aradu.CZY5Q | 0.07 | Unknown protein |
| Aradu.D0HDA | 0.01 | serine/threonine-protein phosphatase 7 long form homolog [Glycine max]; IPR019557 (Aminotransferase-like, plant mobile domain) |
| Aradu.D1SAY | 0.01 | protein FAR1-RELATED SEQUENCE 4-like isoform X2 [Glycine max] |
| Aradu.D3K1L | 0.01 | protein FAR1-RELATED SEQUENCE 6-like isoform X2 [Glycine max]; IPR004330 (FAR1 DNA binding domain), IPR007527 (Zinc finger, SWIM-type); GO:0008270 (zinc ion binding) |
| Aradu.D40PF | 0.02 | Unknown protein |
| Aradu.D699P | 0.03 | probable cytokinin riboside 5'-monophosphate phosphoribohydrolase LOGL5-like [Glycine max] |
| Aradu.DJ0H7 | 0.02 | uncharacterized protein LOC102659825 isoform X5 [Glycine max] |
| Aradu.DPP33 | 0.01 | Unknown protein |
| Aradu.E3XTG | 0.01 | uncharacterized protein LOC100797259 isoform X4 [Glycine max]; IPR001878 (Zinc finger, CCHC-type), IPR004332 (Transposase, MuDR, plant), IPR007527 (Zinc finger, SWIM-type); GO:0003676 (nucleic acid binding), GO:0008270 (zinc ion binding) |
| Aradu.E9MH5 | 0.11 | phosphatidylinositol/phosphatidylcholine transfer protein SFH9-like isoform X2 [Glycine max] |
| Aradu.EF3G2 | 0.02 | 2-oxoglutarate (2OG) and Fe(II)-dependent oxygenase superfamily protein; IPR005123 (Oxoglutarate/iron-dependent dioxygenase), IPR026992 (Non-haem dioxygenase N-terminal domain), IPR027443 (Isopenicillin N synthase-like); GO:0016491 (oxidoreductase activity), GO:0055114 (oxidation-reduction process) |
| Aradu.EFI0B | 0.02 | protein FAR1-RELATED SEQUENCE 5-like isoform X2 [Glycine max]; IPR004330 (FAR1 DNA binding domain) |
| Aradu.ES1P4 | 0.01 | serine/threonine-protein phosphatase 7 long form homolog [Glycine max]; IPR019557 (Aminotransferase-like, plant mobile domain) |
| Aradu.F5W69 | 0.02 | protein FAR1-RELATED SEQUENCE 6-like isoform X2 [Glycine max] |
| Aradu.F91BE | 0.03 | plant intracellular ras group-related LRR 6 |
| Aradu.FCP1P | 0.01 | uncharacterized protein LOC100778822 isoform X6 [Glycine max]; IPR004252 (Probable transposase, Ptta/En/Spm, plant) |
| Aradu.FSY41 | 0.02 | anoctamin-like protein At1g73020-like isoform X2 [Glycine max]; IPR008546 (Domain of unknown function DUF828) |
| Aradu.G0PCV | 0.01 | CTV.20 n=1 Tax=Poncirus trifoliata RepID=Q8H6Q8_PONTR |
| Aradu.G1834 | 0.01 | protein FAR1-RELATED SEQUENCE 5-like [Glycine max]; IPR004330 (FAR1 DNA binding domain) |
| Aradu.GA601 | 0.02 | uncharacterized protein LOC102666784 [Glycine max]; IPR001878 (Zinc finger, CCHC-type), IPR006564 (Zinc finger, PMZ-type); GO:0003676 (nucleic acid binding), GO:0008270 (zinc ion binding) |
| Aradu.GEE6T | 0.02 | Unknown protein |
| Aradu.GPT36 | 0.01 | aluminum-activated malate transporter 1; IPR020966 (Aluminum-activated malate transporter); GO:0015743 (malate transport) |
| Aradu.H461B | 0.02 | Unknown protein |
| Aradu.HD893 | 0.03 | protein FAR1-RELATED SEQUENCE 6-like isoform X4 [Glycine max]; IPR004330 (FAR1 DNA binding domain) |
| Aradu.HGI6B | 0.01 | serine/threonine-protein phosphatase 7 long form homolog [Glycine max]; IPR019557 (Aminotransferase-like, plant mobile domain) |
| Aradu.HXT8B | 0.01 | Ulp1 protease family, carboxy-terminal domain protein; IPR003653 (Peptidase C48, SUMO/Sentrin/Ubl1); GO:0006508 (proteolysis), GO:0008234 (cysteine-type peptidase activity) |
| Aradu.HY3BB | 0.02 | protein FAR-RED IMPAIRED RESPONSE 1-like isoform X3 [Glycine max] |
| Aradu.I8N52 | 0.02 | Myb/SANT-like DNA-binding domain protein; IPR024752 (Myb/SANT-like domain) |
| Aradu.I8NIH | 0.03 | protein FAR1-RELATED SEQUENCE 6-like isoform X2 [Glycine max] |
| Aradu.I92VD | 0.01 | Zinc knuckle family protein n=1 Tax=Oryza sativa subsp. japonica RepID=H2KWL4_ORYSJ |
| Aradu.I959C | 0.02 | Unknown protein |
| Aradu.IXU8Y | 0.1 | Unknown protein |
| Aradu.J65F4 | 0.08 | serine/threonine-protein phosphatase 7 long form homolog [Glycine max]; IPR019557 (Aminotransferase-like, plant mobile domain) |
| Aradu.J7AFC | 0.01 | uncharacterized protein LOC102659972 [Glycine max] |
| Aradu.JU6F0 | 0.07 | Unknown protein |
| Aradu.JZ1T7 | 0.02 | Unknown protein |
| Aradu.KL4PF | 0.01 | PLATZ transcription factor family protein |
| Aradu.KP12T | 0.01 | cellulose synthase 1 |
| Aradu.LPN0N | 0.02 | Zinc knuckle family protein n=1 Tax=Oryza sativa subsp. japonica RepID=H2KW38_ORYSJ |
| Aradu.LS3H9 | 0.29 | putative disease resistance RPP13-like protein 1-like isoform X2 [Glycine max] |
| Aradu.M1JE3 | 0.02 | probable zinc transporter protein DDB_G0291141 isoform 1 [Glycine max] |
| Aradu.M4T8Q | 0.02 | Unknown protein |
| Aradu.M8I4Q | 0.03 | exocyst complex component sec5 |
| Aradu.MK8AD | 0.01 | Unknown protein |
| Aradu.N7C89 | 0.06 | RWP-RK domain-containing protein; IPR003035 (RWP-RK domain) |
| Aradu.NA12J | 0.03 | Unknown protein |
| Aradu.NK0QH | 0.03 | Galactose oxidase/kelch repeat superfamily protein; IPR015916 (Galactose oxidase, beta-propeller) |
| Aradu.NT2DE | 0.04 | bZIP transcription factor family protein |
| Aradu.P7TZB | 0.1 | Unknown protein |
| Aradu.PED3Y | 0.02 | F-box protein interaction domain protein |
| Aradu.PFG5A | 0.01 | Unknown protein |
| Aradu.PJE37 | 0.02 | n=1 Tax=Oryza sativa subsp. japonica RepID=Q7XRM9_ORYSJ; IPR004332 (Transposase, MuDR, plant) |
| Aradu.PY0W5 | 0.02 | Unknown protein |
| Aradu.Q04ZM | 0.01 | ADP-ribosylation factor-like protein A1D |
| Aradu.RB1B4 | 0.01 | probable glycosyltransferase At5g03795-like isoform X2 [Glycine max] |
| Aradu.RGQ39 | 0.01 | serine/threonine-protein phosphatase 7 long form homolog [Glycine max]; IPR019557 (Aminotransferase-like, plant mobile domain) |
| Aradu.RV4NC | 0.1 | Unknown protein |
| Aradu.S9RF3 | 0.02 | putative ubiquitin-like-specific protease 1B-like isoform X2 [Glycine max]; IPR003653 (Peptidase C48, SUMO/Sentrin/Ubl1); GO:0006508 (proteolysis), GO:0008234 (cysteine-type peptidase activity) |
| Aradu.SS5XF | 0.02 | Unknown protein |
| Aradu.T2XN3 | 0.01 | Unknown protein; IPR003653 (Peptidase C48, SUMO/Sentrin/Ubl1); GO:0006508 (proteolysis), GO:0008234 (cysteine-type peptidase activity) |
| Aradu.TTL74 | 0.02 | Unknown protein |
| Aradu.TUS43 | 0.02 | coatomer subunit gamma-like protein; IPR016024 (Armadillo-type fold), IPR017106 (Coatomer gamma subunit); GO:0005488 (binding), GO:0006886 (intracellular protein transport), GO:0016192 (vesicle-mediated transport), GO:0030117 (membrane coat) |
| Aradu.U1CB9 | 0.05 | uncharacterized protein LOC100793882 isoform X2 [Glycine max]; IPR008546 (Domain of unknown function DUF828) |
| Aradu.UH16H | 0.01 | myb transcription factor; IPR009057 (Homeodomain-like); GO:0003677 (DNA binding), GO:0003682 (chromatin binding) |
| Aradu.V1RSV | 0.01 | serine/threonine-protein phosphatase 7 long form homolog [Glycine max]; IPR019557 (Aminotransferase-like, plant mobile domain) |
| Aradu.V4AWT | 0.03 | isoflavone reductase homolog [Glycine max]; IPR008030 (NmrA-like) |
| Aradu.VA5MB | 0.03 | probable serine/threonine protein phosphatase 2A regulatory subunit B''delta-like isoform X3 [Glycine max] |
| Aradu.VI60E | 0.02 | photosystem II CP43 chlorophyll apoprotein; IPR000932 (Photosystem antenna protein-like); GO:0009521 (photosystem), GO:0009767 (photosynthetic electron transport chain), GO:0016020 (membrane), GO:0016168 (chlorophyll binding) |
| Aradu.VUR0X | 0.02 | disease resistance protein (TIR-NBS-LRR class), putative; IPR002182 (NB-ARC), IPR027417 (P-loop containing nucleoside triphosphate hydrolase); GO:0043531 (ADP binding) |
| Aradu.VY5NG | 0.01 | Myb/SANT-like DNA-binding domain protein |
| Aradu.W296H | 0.03 | Unknown protein |
| Aradu.W3QNR | 0.01 | zinc finger MYM-type protein 1-like [Glycine max] |
| Aradu.W46ZZ | 0.02 | NAC transcription factor-like protein |
| Aradu.W7LE9 | 0.02 | Cold acclimation protein COR413-TM1 n=2 Tax=Medicago truncatula RepID=G7L817_MEDTR; IPR008892 (Cold acclimation WCOR413) |
| Aradu.WA79I | 0.01 | Unknown protein |
| Aradu.X0PCU | 0.02 | Ulp1 protease family, carboxy-terminal domain protein |
| Aradu.X23GX | 0.02 | Unknown protein |
| Aradu.XD9PZ | 0.01 | Unknown protein |
| Aradu.Y4K0T | 0.01 | uncharacterized protein LOC100797259 isoform X4 [Glycine max]; IPR004332 (Transposase, MuDR, plant) |
| Aradu.YI18P | 0.01 | DUF674 family protein; IPR007750 (Protein of unknown function DUF674) |
| Aradu.YQM8E | 0.02 | Unknown protein |
| Aradu.Z6X3K | 0.04 | putative nuclease HARBI1-like [Glycine max] |
| Aradu.Z7HPQ | 0.02 | protein FAR1-RELATED SEQUENCE 6-like isoform X2 [Glycine max] |
| Aradu.ZRL0U | 0.03 | Unknown protein |
| Aradu.ZW90W | 0.01 | uncharacterized protein LOC100797259 isoform X3 [Glycine max]; IPR004332 (Transposase, MuDR, plant), IPR006564 (Zinc finger, PMZ-type); GO:0008270 (zinc ion binding) |
| Araip.05U4T | 0.02 | Unknown protein |
| Araip.08LYA | 0.01 | zinc finger MYM-type protein 1-like [Glycine max]; IPR012337 (Ribonuclease H-like domain), IPR025398 (Domain of unknown function DUF4371); GO:0003676 (nucleic acid binding) |
| Araip.0I59F | 0.02 | protein kinase family protein; IPR011009 (Protein kinase-like domain) |
| Araip.0LB0Z | 0.01 | n=1 Tax=Oryza sativa subsp. japonica RepID=Q7FAE4_ORYSJ; IPR004332 (Transposase, MuDR, plant) |
| Araip.0LJ0X | 0.02 | ribonucleoside-diphosphate reductase; IPR000788 (Ribonucleotide reductase large subunit, C-terminal); GO:0006260 (DNA replication), GO:0055114 (oxidation-reduction process) |
| Araip.0T2UC | 0.01 | Polyprotein n=5 Tax=Citrus endogenous pararetrovirus RepID=V9QF33_9RETR |
| Araip.0UB4L | 0.02 | protein notum homolog isoform X2 [Glycine max]; IPR004963 (Protein notum homologue) |
| Araip.11XKS | 0.02 | E3 ubiquitin-protein ligase n=5 Tax=Solanum RepID=M1BDZ1_SOLTU |
| Araip.15LU1 | 0.01 | protein FAR1-RELATED SEQUENCE 7-like isoform X1 [Glycine max]; IPR004330 (FAR1 DNA binding domain) |
| Araip.167UL | 0.03 | serine/threonine-protein phosphatase 7 long form homolog [Glycine max]; IPR019557 (Aminotransferase-like, plant mobile domain) |
| Araip.1E51H | 0.02 | uncharacterized protein LOC100779930 isoform X2 [Glycine max] |
| Araip.1PP0A | 0.17 | protein n=1 Tax=Oryza sativa subsp. japonica RepID=Q0DJ09_ORYSJ; IPR025312 (Domain of unknown function DUF4216) |
| Araip.2DH61 | 0.01 | protein FAR1-RELATED SEQUENCE 5-like [Glycine max]; IPR004330 (FAR1 DNA binding domain), IPR007527 (Zinc finger, SWIM-type), IPR025151 (ELYS-like domain); GO:0008270 (zinc ion binding) |
| Araip.2KU0V | 0.04 | Pentatricopeptide repeat (PPR-like) superfamily protein; IPR002885 (Pentatricopeptide repeat) |
| Araip.34VJI | 0.02 | MEI2-like protein 5; IPR007201 (RNA recognition motif 2) |
| Araip.36VDQ | 0.04 | Ankyrin repeat family protein |
| Araip.38KWX | 0.01 | serine/threonine-protein phosphatase 7 long form homolog [Glycine max]; IPR019557 (Aminotransferase-like, plant mobile domain) |
| Araip.3PL9S | 0.02 | serine/threonine-protein phosphatase 7 long form homolog [Glycine max]; IPR019557 (Aminotransferase-like, plant mobile domain) |
| Araip.3XS24 | 0.05 | Unknown protein |
| Araip.4747R | 0.01 | protein n=1 Tax=Oryza sativa subsp. japonica RepID=C7J0D1_ORYSJ; IPR007527 (Zinc finger, SWIM-type); GO:0008270 (zinc ion binding) |
| Araip.4A7MJ | 0.03 | Protein kinase superfamily protein; IPR011009 (Protein kinase-like domain), IPR013320 (Concanavalin A-like lectin/glucanase, subgroup); GO:0004672 (protein kinase activity), GO:0006468 (protein phosphorylation) |
| Araip.4FT57 | 0.02 | ATP-dependent zinc metalloprotease FtsH-like isoform X2 [Glycine max] |
| Araip.4P4A1 | 0.01 | protein n=1 Tax=Oryza sativa subsp. japonica RepID=C7J9W8_ORYSJ; IPR004332 (Transposase, MuDR, plant) |
| Araip.4QP2D | 0.01 | protein n=1 Tax=Oryza sativa subsp. japonica RepID=C7J340_ORYSJ; IPR001878 (Zinc finger, CCHC-type), IPR007527 (Zinc finger, SWIM-type); GO:0003676 (nucleic acid binding), GO:0008270 (zinc ion binding) |
| Araip.51XQ9 | 0.01 | serine/threonine-protein phosphatase 7 long form homolog [Glycine max]; IPR019557 (Aminotransferase-like, plant mobile domain) |
| Araip.5ED43 | 0.05 | Unknown protein |
| Araip.60G2Q | 0.01 | n=1 Tax=Oryza sativa subsp. japonica RepID=Q7XVP9_ORYSJ; IPR019557 (Aminotransferase-like, plant mobile domain) |
| Araip.63HLP | 0.01 | Unknown protein; IPR015300 (DNA-binding pseudobarrel domain); GO:0003677 (DNA binding) |
| Araip.65QZN | 0.03 | GRF zinc finger protein; IPR010666 (Zinc finger, GRF-type); GO:0008270 (zinc ion binding) |
| Araip.735YD | 0.01 | Ulp1 protease family, carboxy-terminal domain protein; IPR003653 (Peptidase C48, SUMO/Sentrin/Ubl1); GO:0006508 (proteolysis), GO:0008234 (cysteine-type peptidase activity) |
| Araip.77JQZ | 0.02 | Unknown protein |
| Araip.7K2IH | 0.01 | n=1 Tax=Oryza sativa subsp. japonica RepID=Q7XVP9_ORYSJ |
| Araip.7LF0L | 0.02 | Unknown protein; IPR009027 (Ribosomal protein L9/RNase H1, N-terminal) |
| Araip.7P6AI | 0.03 | Unknown protein |
| Araip.83XH1 | 0.03 | Unknown protein |
| Araip.8J9QN | 0.01 | n=1 Tax=Oryza sativa subsp. japonica RepID=Q7XTA1_ORYSJ; IPR007527 (Zinc finger, SWIM-type); GO:0008270 (zinc ion binding) |
| Araip.90USA | 0.05 | Unknown protein |
| Araip.94IRW | 0.03 | Unknown protein |
| Araip.95702 | 0.01 | serine/threonine-protein phosphatase 7 long form homolog [Glycine max]; IPR019557 (Aminotransferase-like, plant mobile domain) |
| Araip.95GVY | 0.1 | Unknown protein |
| Araip.95UZY | 0.01 | n=2 Tax=Oryza sativa subsp. japonica RepID=Q7XNL9_ORYSJ |
| Araip.9BA6D | 0.01 | Unknown protein |
| Araip.9T0I7 | 0.02 | ferric-chelate reductase 1-like [Glycine max]; IPR005018 (DOMON domain) |
| Araip.9ZL05 | 0.01 | n=1 Tax=Oryza sativa subsp. japonica RepID=Q7XVP9_ORYSJ |
| Araip.A1AP8 | 0.01 | uncharacterized protein LOC102664163 isoform X5 [Glycine max] |
| Araip.A1GUV | 0.02 | receptor-like kinase; IPR001611 (Leucine-rich repeat); GO:0005515 (protein binding) |
| Araip.A1ZFD | 0.04 | Unknown protein |
| Araip.A75P3 | 0.04 | Unknown protein |
| Araip.A9C1V | 0.01 | Unknown protein; IPR003653 (Peptidase C48, SUMO/Sentrin/Ubl1); GO:0006508 (proteolysis), GO:0008234 (cysteine-type peptidase activity) |
| Araip.ABI9A | 0.01 | Protein kinase superfamily protein; IPR011009 (Protein kinase-like domain); GO:0004672 (protein kinase activity), GO:0005524 (ATP binding), GO:0006468 (protein phosphorylation) |
| Araip.AP88X | 0.01 | Unknown protein; IPR003653 (Peptidase C48, SUMO/Sentrin/Ubl1); GO:0006508 (proteolysis), GO:0008234 (cysteine-type peptidase activity) |
| Araip.BDC4Y | 0.05 | hypothetical protein |
| Araip.BI7CH | 0.01 | myo-inositol oxygenase 4; IPR007828 (Inositol oxygenase), IPR008906 (HAT dimerisation domain, C-terminal); GO:0005506 (iron ion binding), GO:0005737 (cytoplasm), GO:0019310 (inositol catabolic process), GO:0046983 (protein dimerization activity), GO:0050113 (inositol oxygenase activity), GO:0055114 (oxidation-reduction process) |
| Araip.BJK02 | 0.43 | Unknown protein |
| Araip.CNH4K | 0.01 | Unknown protein |
| Araip.D1IP2 | 0.01 | receptor-like protein kinase 4; IPR011009 (Protein kinase-like domain); GO:0004672 (protein kinase activity), GO:0006468 (protein phosphorylation) |
| Araip.D4YV6 | 0.01 | serine/threonine-protein phosphatase 7 long form homolog [Glycine max]; IPR019557 (Aminotransferase-like, plant mobile domain) |
| Araip.D824R | 0.02 | Unknown protein |
| Araip.D9W37 | 0.02 | Unknown protein; IPR004252 (Probable transposase, Ptta/En/Spm, plant) |
| Araip.DB4YN | 0.01 | serine/threonine-protein phosphatase 7 long form homolog [Glycine max]; IPR019557 (Aminotransferase-like, plant mobile domain) |
| Araip.DSQ3Q | 0.01 | Unknown protein |
| Araip.DYI0V | 0.02 | peroxisomal 3-ketoacyl-CoA thiolase 3; IPR002155 (Thiolase), IPR016039 (Thiolase-like); GO:0003824 (catalytic activity), GO:0008152 (metabolic process) |
| Araip.E3ETS | 0.04 | Cytochrome P450 superfamily protein; IPR001128 (Cytochrome P450); GO:0005506 (iron ion binding), GO:0020037 (heme binding), GO:0055114 (oxidation-reduction process) |
| Araip.E4FSH | 0.02 | Glutathione S-transferase family protein; IPR010987 (Glutathione S-transferase, C-terminal-like), IPR012336 (Thioredoxin-like fold); GO:0005515 (protein binding) |
| Araip.E4L3Y | 0.03 | ubiquitin protein ligase 6; IPR000569 (HECT); GO:0004842 (ubiquitin-protein ligase activity) |
| Araip.EP0SE | 0.03 | Unknown protein |
| Araip.ER14W | 0.02 | Unknown protein |
| Araip.ERY8X | 0.01 | uncharacterized protein LOC100807790 [Glycine max] |
| Araip.F67ML | 0.03 | Unknown protein |
| Araip.F83ZN | 0.01 | Unknown protein |
| Araip.FD86Q | 0.03 | Oxysterol-binding family protein |
| Araip.FE3A4 | 0.02 | serine/threonine-protein phosphatase 7 long form homolog [Glycine max]; IPR019557 (Aminotransferase-like, plant mobile domain) |
| Araip.FW1K9 | 0.01 | uncharacterized protein LOC102669356 [Glycine max]; IPR016135 (Ubiquitin-conjugating enzyme/RWD-like), IPR025312 (Domain of unknown function DUF4216) |
| Araip.G0GUG | 0.04 | Unknown protein |
| Araip.G2SDM | 0.02 | Unknown protein |
| Araip.GHX3I | 0.03 | E3 UFM1-protein ligase-like protein; IPR018611 (E3 UFM1-protein ligase 1) |
| Araip.GN8EF | 0.03 | Zinc finger GRF-type protein n=1 Tax=Arachis hypogaea RepID=G0Y6U2_ARAHY |
| Araip.GT3TW | 0.02 | LRR and NB-ARC domain disease resistance protein; IPR000767 (Disease resistance protein), IPR027417 (P-loop containing nucleoside triphosphate hydrolase); GO:0006952 (defense response), GO:0043531 (ADP binding) |
| Araip.H0CEM | 0.02 | protein FAR1-RELATED SEQUENCE 6-like isoform X2 [Glycine max]; IPR004330 (FAR1 DNA binding domain), IPR007527 (Zinc finger, SWIM-type); GO:0008270 (zinc ion binding) |
| Araip.H3PDQ | 0.01 | Unknown protein |
| Araip.H6K7J | 0.13 | 5-hydroxyisourate hydrolase |
| Araip.H8U0X | 0.24 | LOB domain-containing protein 18-like [Glycine max] |
| Araip.HCY8H | 0.01 | uncharacterized protein LOC100797259 isoform X3 [Glycine max]; IPR004332 (Transposase, MuDR, plant), IPR007527 (Zinc finger, SWIM-type); GO:0008270 (zinc ion binding) |
| Araip.HF5JQ | 0.02 | Unknown protein |
| Araip.HIG3P | 0.05 | subtilisin-like serine protease |
| Araip.I5544 | 1.56 | Unknown protein |
| Araip.II3G2 | 0.03 | GRF zinc finger protein; IPR010666 (Zinc finger, GRF-type); GO:0008270 (zinc ion binding) |
| Araip.IU5SB | 0.06 | Unknown protein |
| Araip.J1A84 | 0.01 | protein FAR1-RELATED SEQUENCE 7-like isoform X1 [Glycine max]; IPR004330 (FAR1 DNA binding domain) |
| Araip.J3LMW | 0.01 | protein FAR1-RELATED SEQUENCE 6-like isoform X2 [Glycine max]; IPR007527 (Zinc finger, SWIM-type); GO:0008270 (zinc ion binding) |
| Araip.J496S | 0.02 | Unknown protein |
| Araip.J4V9D | 0.01 | Unknown protein |
| Araip.J8JRL | 0.04 | expansin-like B1-like [Glycine max]; IPR007117 (Expansin, cellulose-binding-like domain) |
| Araip.JC1WY | 0.03 | Unknown protein; IPR009027 (Ribosomal protein L9/RNase H1, N-terminal) |
| Araip.JH4MY | 0.02 | serine/threonine-protein phosphatase 7 long form homolog [Glycine max]; IPR019557 (Aminotransferase-like, plant mobile domain) |
| Araip.JK7I0 | 0.01 | serine/threonine-protein phosphatase 7 long form homolog [Glycine max]; IPR019557 (Aminotransferase-like, plant mobile domain) |
| Araip.JM2HR | 0.01 | E3 ubiquitin-protein ligase n=5 Tax=Solanum RepID=M1BDZ1_SOLTU |
| Araip.JRS7H | 0.01 | Unknown protein |
| Araip.JX56Z | 0.02 | probable WRKY transcription factor 35-like [Glycine max]; IPR003657 (DNA-binding WRKY); GO:0003700 (sequence-specific DNA binding transcription factor activity), GO:0043565 (sequence-specific DNA binding) |
| Araip.K6HB2 | 0.01 | Unknown protein |
| Araip.K6WV8 | 0.01 | topless-related protein 1-like isoform X3 [Glycine max] |
| Araip.K82M7 | 0.01 | n=1 Tax=Oryza sativa subsp. japonica RepID=Q7XVP9_ORYSJ |
| Araip.KH9KQ | 0.02 | Unknown protein |
| Araip.KTA8L | 0.01 | protein FAR1-RELATED SEQUENCE 6-like isoform X2 [Glycine max] |
| Araip.KZ43I | 0.05 | protein PHYLLO, chloroplastic-like isoform X3 [Glycine max] |
| Araip.L2HWC | 0.01 | FAR1 DNA-binding domain protein; IPR004330 (FAR1 DNA binding domain) |
| Araip.L51M1 | 0.01 | LORELEI-LIKE-GPI-ANCHORED PROTEIN 1 |
| Araip.LCM3H | 0.04 | serine/threonine-protein phosphatase 7 long form homolog [Glycine max]; IPR019557 (Aminotransferase-like, plant mobile domain) |
| Araip.M0ZGA | 0.01 | Unknown protein |
| Araip.M2DKH | 0.01 | callose synthase 2-like isoform X2 [Glycine max]; IPR016040 (NAD(P)-binding domain) |
| Araip.M3SY3 | 0.01 | protein n=1 Tax=Oryza sativa subsp. japonica RepID=C7J340_ORYSJ; IPR004332 (Transposase, MuDR, plant) |
| Araip.MVL7G | 0.04 | caffeic acid O-methyltransferase; IPR001077 (O-methyltransferase, family 2); GO:0008171 (O-methyltransferase activity) |
| Araip.MWQ9H | 0.03 | RAB GTPase homolog A3; IPR001806 (Small GTPase superfamily), IPR005225 (Small GTP-binding protein domain), IPR027417 (P-loop containing nucleoside triphosphate hydrolase); GO:0005525 (GTP binding), GO:0007264 (small GTPase mediated signal transduction), GO:0015031 (protein transport) |
| Araip.N2UDW | 0.01 | protein FAR1-RELATED SEQUENCE 5-like [Glycine max]; IPR001878 (Zinc finger, CCHC-type), IPR004330 (FAR1 DNA binding domain), IPR006564 (Zinc finger, PMZ-type); GO:0003676 (nucleic acid binding), GO:0008270 (zinc ion binding) |
| Araip.N4CW3 | 0.03 | Unknown protein |
| Araip.N7MDU | 0.09 | n=1 Tax=Oryza sativa subsp. japonica RepID=Q7XVP9_ORYSJ; IPR019557 (Aminotransferase-like, plant mobile domain) |
| Araip.N98UC | 0.04 | alba DNA/RNA-binding protein |
| Araip.NA2UP | 0.01 | 2-oxoglutarate (2OG) and Fe(II)-dependent oxygenase superfamily protein; IPR005123 (Oxoglutarate/iron-dependent dioxygenase), IPR027443 (Isopenicillin N synthase-like); GO:0016491 (oxidoreductase activity), GO:0055114 (oxidation-reduction process) |
| Araip.NY97S | 0.01 | uncharacterized protein LOC102664163 isoform X5 [Glycine max]; IPR004252 (Probable transposase, Ptta/En/Spm, plant) |
| Araip.P03Z0 | 0.02 | serine/threonine-protein phosphatase 7 long form homolog [Glycine max]; IPR019557 (Aminotransferase-like, plant mobile domain) |
| Araip.P2BEY | 0.08 | Coproporphyrinogen III oxidase; IPR001260 (Coproporphyrinogen III oxidase, aerobic); GO:0004109 (coproporphyrinogen oxidase activity), GO:0006779 (porphyrin-containing compound biosynthetic process), GO:0055114 (oxidation-reduction process) |
| Araip.P31LI | 0.02 | E3 ubiquitin-protein ligase n=5 Tax=Solanum RepID=M1BDZ1_SOLTU |
| Araip.P4GTD | 0.06 | helix loop helix DNA-binding domain protein; IPR011598 (Myc-type, basic helix-loop-helix (bHLH) domain); GO:0046983 (protein dimerization activity) |
| Araip.P7XGH | 0.02 | GRF zinc finger protein; IPR010666 (Zinc finger, GRF-type); GO:0008270 (zinc ion binding) |
| Araip.P9MW5 | 0.01 | serine/threonine-protein phosphatase 7 long form homolog [Glycine max]; IPR019557 (Aminotransferase-like, plant mobile domain) |
| Araip.QR2HG | 0.01 | probable pectinesterase/pectinesterase inhibitor 21-like [Glycine max]; IPR006501 (Pectinesterase inhibitor domain), IPR011050 (Pectin lyase fold/virulence factor); GO:0004857 (enzyme inhibitor activity), GO:0005618 (cell wall), GO:0030599 (pectinesterase activity), GO:0042545 (cell wall modification) |
| Araip.QSW9C | 0.02 | glutamate receptor 2.7; IPR001828 (Extracellular ligand-binding receptor), IPR028082 (Periplasmic binding protein-like I) |
| Araip.QT6UY | 0.01 | fatty acyl-CoA reductase 3-like [Glycine max]; IPR016040 (NAD(P)-binding domain), IPR026055 (Fatty acyl-CoA reductase); GO:0080019 (fatty-acyl-CoA reductase (alcohol-forming) activity) |
| Araip.R1KI9 | 0.01 | phosphomevalonate kinase-like isoform X2 [Glycine max] |
| Araip.R3TTD | 0.02 | serine/threonine-protein phosphatase 7 long form homolog [Glycine max]; IPR019557 (Aminotransferase-like, plant mobile domain) |
| Araip.R8IWQ | 0.02 | peptide transporter 1; IPR000109 (Proton-dependent oligopeptide transporter family); GO:0005215 (transporter activity), GO:0006810 (transport), GO:0016020 (membrane) |
| Araip.RE9BT | 0.03 | Transposon protein n=1 Tax=Arachis hypogaea RepID=G0Y6V7_ARAHY |
| Araip.RK5LS | 0.02 | homeobox-leucine zipper protein ATHB-14-like [Glycine max] |
| Araip.RL2FG | 0.01 | Unknown protein |
| Araip.RP48U | 0.14 | n=1 Tax=Oryza sativa subsp. japonica RepID=Q7XVP9_ORYSJ; IPR019557 (Aminotransferase-like, plant mobile domain) |
| Araip.RX0KJ | 0.01 | UDP-Glycosyltransferase superfamily protein |
| Araip.S90FS | 0.02 | protein MOR1-like isoform X2 [Glycine max]; IPR025941 (Vacuolar protein sorting-associated protein 8, central domain) |
| Araip.SI55I | 0.03 | Unknown protein |
| Araip.SQ3RJ | 0.03 | Unknown protein |
| Araip.SUC7B | 1.19 | protein n=1 Tax=Oryza sativa subsp. japonica RepID=C7J0D1_ORYSJ; IPR007527 (Zinc finger, SWIM-type); GO:0008270 (zinc ion binding) |
| Araip.SV6WW | 0.01 | Zinc knuckle family protein n=1 Tax=Oryza sativa subsp. japonica RepID=H2KWL4_ORYSJ |
| Araip.T5XAB | 0.03 | Unknown protein |
| Araip.T9MPA | 0.03 | uncharacterized protein LOC102669491 isoform X2 [Glycine max] |
| Araip.TFS01 | 0.02 | Unknown protein |
| Araip.TII3Q | 0.01 | E3 ubiquitin-protein ligase n=5 Tax=Solanum RepID=M1BDZ1_SOLTU |
| Araip.TL5BE | 0.17 | Disease resistance protein (TIR-NBS class); IPR000157 (Toll/interleukin-1 receptor homology (TIR) domain); GO:0005515 (protein binding), GO:0007165 (signal transduction) |
| Araip.TYG4D | 0.02 | Putative lysine decarboxylase family protein |
| Araip.TZA2F | 0.01 | uncharacterized protein LOC102669491 isoform X2 [Glycine max] |
| Araip.U2EMB | 0.01 | Ulp1 protease family, carboxy-terminal domain protein; IPR003653 (Peptidase C48, SUMO/Sentrin/Ubl1); GO:0006508 (proteolysis), GO:0008234 (cysteine-type peptidase activity) |
| Araip.U4VFI | 0.03 | uncharacterized protein LOC100783475 isoform X3 [Glycine max] |
| Araip.U701X | 0.01 | serine/threonine-protein phosphatase 7 long form homolog [Glycine max]; IPR019557 (Aminotransferase-like, plant mobile domain) |
| Araip.U7TSU | 0.01 | uncharacterized protein LOC100808214 isoform X1 [Glycine max] |
| Araip.UF9PL | 0.01 | GRF zinc finger protein; IPR010666 (Zinc finger, GRF-type); GO:0008270 (zinc ion binding) |
| Araip.UK3NI | 0.03 | Calcium-binding EF-hand family protein; IPR011992 (EF-hand domain pair); GO:0005509 (calcium ion binding) |
| Araip.USB72 | 0.02 | uncharacterized protein LOC100778822 isoform X6 [Glycine max]; IPR004252 (Probable transposase, Ptta/En/Spm, plant) |
| Araip.VE5TX | 0.01 | serine/threonine-protein phosphatase 7 long form homolog [Glycine max]; IPR019557 (Aminotransferase-like, plant mobile domain) |
| Araip.VRK2M | 0.02 | beta glucosidase 40; IPR001360 (Glycoside hydrolase, family 1), IPR017853 (Glycoside hydrolase, superfamily); GO:0005975 (carbohydrate metabolic process) |
| Araip.W2YGT | 0.03 | Unknown protein |
| Araip.WJ1MN | 0.01 | receptor-like protein kinase 4; IPR011009 (Protein kinase-like domain); GO:0004672 (protein kinase activity), GO:0005524 (ATP binding), GO:0006468 (protein phosphorylation) |
| Araip.WU6BG | 0.01 | alpha/beta-Hydrolases superfamily protein |
| Araip.WZ813 | 0.05 | Unknown protein |
| Araip.X1FU1 | 0.01 | hypothetical protein |
| Araip.X1TUQ | 0.01 | E3 ubiquitin-protein ligase n=5 Tax=Solanum RepID=M1BDZ1_SOLTU |
| Araip.X7TYK | 0.03 | UPF0481 protein At3g47200-like [Glycine max]; IPR004158 (Protein of unknown function DUF247, plant) |
| Araip.X9M41 | 0.01 | Unknown protein |
| Araip.XB97Q | 0.02 | Unknown protein |
| Araip.XR7FV | 0.07 | Unknown protein |
| Araip.XWP37 | 0.01 | viral movement protein |
| Araip.Y55CK | 0.01 | geranylgeranyl transferase type-1 subunit beta-like [Glycine max] |
| Araip.YSA7B | 0.05 | serine/threonine-protein phosphatase 7 long form homolog [Glycine max]; IPR019557 (Aminotransferase-like, plant mobile domain) |
| Araip.YYN82 | 0.02 | Unknown protein |
| Araip.Z2Q8C | 0.03 | chromosome-associated kinesin KIF4A-like isoform X1 [Glycine max]; IPR000449 (Ubiquitin-associated domain/translation elongation factor EF-Ts, N-terminal), IPR002671 (Ribosomal protein L22e); GO:0003735 (structural constituent of ribosome), GO:0005515 (protein binding), GO:0005622 (intracellular), GO:0005840 (ribosome), GO:0006412 (translation) |
| Araip.ZAB0E | 0.08 | Zinc finger GRF-type protein n=1 Tax=Arachis hypogaea RepID=G0Y6U2_ARAHY |
| Araip.ZJ030 | 0.03 | Unknown protein |
| Araip.ZY979 | 0.01 | putative ubiquitin-like-specific protease 1B-like isoform X2 [Glycine max] |
| Araip.ZZQ8R | 0.03 | uncharacterized mitochondrial protein AtMg00810-like [Glycine max]; IPR004217 (Tim10/DDP family zinc finger) |
| Perianth |  |  |
| Aradu.0H427 | 0.01 | LRR and NB-ARC domain disease resistance protein |
| Aradu.16QN3 | 0.68 | Cytochrome P450 superfamily protein; IPR001128 (Cytochrome P450); GO:0005506 (iron ion binding), GO:0020037 (heme binding), GO:0055114 (oxidation-reduction process) |
| Aradu.1A85M | 0.02 | Unknown protein |
| Aradu.1E4GW | 0.01 | viral movement protein |
| Aradu.1YP56 | 0.03 | GRF zinc finger protein; IPR010666 (Zinc finger, GRF-type); GO:0008270 (zinc ion binding) |
| Aradu.25PCJ | 0.07 | Unknown protein |
| Aradu.2MW6D | 0.06 | Unknown protein |
| Aradu.4WH7N | 0.03 | pachytene checkpoint protein 2 homolog isoform X1 [Glycine max]; IPR027417 (P-loop containing nucleoside triphosphate hydrolase); GO:0005524 (ATP binding) |
| Aradu.58UG1 | 0.23 | pachytene checkpoint protein 2 homolog isoform X1 [Glycine max]; IPR027417 (P-loop containing nucleoside triphosphate hydrolase); GO:0005524 (ATP binding) |
| Aradu.6BY4N | 0.02 | dual specificity protein phosphatase 1; IPR000340 (Dual specificity phosphatase, catalytic domain), IPR020422 (Dual specificity phosphatase, subgroup, catalytic domain), IPR024950 (Dual specificity phosphatase); GO:0006470 (protein dephosphorylation), GO:0008138 (protein tyrosine/serine/threonine phosphatase activity) |
| Aradu.77FMA | 0.06 | Unknown protein |
| Aradu.8EY8P | 0.06 | pyruvate orthophosphate dikinase; IPR010121 (Pyruvate, phosphate dikinase), IPR015813 (Pyruvate/Phosphoenolpyruvate kinase-like domain); GO:0003824 (catalytic activity), GO:0006090 (pyruvate metabolic process), GO:0016310 (phosphorylation) |
| Aradu.8HV1H | 0.39 | Cytochrome P450 superfamily protein; IPR001128 (Cytochrome P450); GO:0005506 (iron ion binding), GO:0020037 (heme binding), GO:0055114 (oxidation-reduction process) |
| Aradu.9U6GJ | 0.02 | zinc finger MYM-type protein 1-like [Glycine max]; IPR006580 (Zinc finger, TTF-type), IPR025398 (Domain of unknown function DUF4371) |
| Aradu.BRU54 | 0.05 | Epstein-Barr virus EBNA-1-like protein n=1 Tax=Oryza sativa subsp. japonica RepID=Q6EPD4_ORYSJ |
| Aradu.C8CVG | 0.03 | protein FAR1-RELATED SEQUENCE 6-like isoform X2 [Glycine max]; IPR004330 (FAR1 DNA binding domain), IPR006564 (Zinc finger, PMZ-type); GO:0008270 (zinc ion binding) |
| Aradu.D4WBJ | 0.23 | 50S ribosomal protein L14; IPR000218 (Ribosomal protein L14b/L23e), IPR023571 (Ribosomal protein L14 domain); GO:0003735 (structural constituent of ribosome), GO:0005840 (ribosome), GO:0006412 (translation) |
| Aradu.D8MN6 | 0.01 | Unknown protein |
| Aradu.F6F2C | 0.02 | serine/threonine-protein phosphatase 7 long form homolog [Glycine max]; IPR019557 (Aminotransferase-like, plant mobile domain) |
| Aradu.FJI3W | 0.04 | ribosomal protein L18; IPR000039 (Ribosomal protein L18e), IPR004843 (Calcineurin-like phosphoesterase domain, apaH type); GO:0003735 (structural constituent of ribosome), GO:0005622 (intracellular), GO:0005840 (ribosome), GO:0006412 (translation), GO:0016787 (hydrolase activity) |
| Aradu.G6VUF | 0.09 | Unknown protein |
| Aradu.GZ1ZI | 0.14 | ubiquitin conjugating enzyme 8; IPR016135 (Ubiquitin-conjugating enzyme/RWD-like); GO:0016881 (acid-amino acid ligase activity) |
| Aradu.HG68U | 0.02 | Cysteine proteinases superfamily protein; IPR013128 (Peptidase C1A), IPR025660 (Cysteine peptidase, histidine active site), IPR025661 (Cysteine peptidase, asparagine active site); GO:0006508 (proteolysis), GO:0008234 (cysteine-type peptidase activity) |
| Aradu.IH1NN | 0.05 | beta-amyrin synthase-like isoform X2 [Glycine max]; IPR008930 (Terpenoid cyclases/protein prenyltransferase alpha-alpha toroid) |
| Aradu.IM7TN | 0.04 | disease resistance protein (TIR-NBS-LRR class), putative; IPR000767 (Disease resistance protein), IPR027417 (P-loop containing nucleoside triphosphate hydrolase); GO:0006952 (defense response), GO:0043531 (ADP binding) |
| Aradu.J296J | 0.1 | Cytochrome P450 superfamily protein; IPR001128 (Cytochrome P450); GO:0005506 (iron ion binding), GO:0020037 (heme binding), GO:0055114 (oxidation-reduction process) |
| Aradu.M4BU3 | 0.02 | pachytene checkpoint protein 2 homolog isoform X1 [Glycine max]; IPR027417 (P-loop containing nucleoside triphosphate hydrolase); GO:0005524 (ATP binding) |
| Aradu.MA4LY | 0.01 | Ulp1 protease family, carboxy-terminal domain protein |
| Aradu.MME7S | 0.03 | Unknown protein |
| Aradu.N5TIL | 0.04 | uncharacterized protein LOC100797101 [Glycine max] |
| Aradu.NH4VM | 0.04 | NADH-ubiquinone oxidoreductase chain n=1 Tax=Medicago truncatula RepID=G7I8X7_MEDTR |
| Aradu.NTU88 | 0.02 | Cytochrome C assembly protein chrM:256865-257476 REVERSE; IPR002541 (Cytochrome c assembly protein), IPR003567 (Cytochrome c-type biogenesis protein); GO:0006461 (protein complex assembly), GO:0008535 (respiratory chain complex IV assembly), GO:0015232 (heme transporter activity), GO:0015886 (heme transport), GO:0016020 (membrane), GO:0017004 (cytochrome complex assembly) |
| Aradu.R5HU3 | 0.09 | disease resistance protein (TIR-NBS-LRR class) |
| Aradu.S38FI | 0.02 | Protein phosphatase 2C family protein; IPR001932 (Protein phosphatase 2C (PP2C)-like domain), IPR015655 (Protein phosphatase 2C); GO:0003824 (catalytic activity) |
| Aradu.T3TT2 | 0.04 | Unknown protein |
| Aradu.T6IGM | 0.01 | Cytochrome C assembly protein; IPR002541 (Cytochrome c assembly protein), IPR003567 (Cytochrome c-type biogenesis protein); GO:0006461 (protein complex assembly), GO:0008535 (respiratory chain complex IV assembly), GO:0015232 (heme transporter activity), GO:0015886 (heme transport), GO:0016020 (membrane), GO:0017004 (cytochrome complex assembly) |
| Aradu.TKN3L | 0.05 | beta-amyrin synthase-like isoform X2 [Glycine max]; IPR008930 (Terpenoid cyclases/protein prenyltransferase alpha-alpha toroid) |
| Aradu.U2BB7 | 0.08 | uncharacterized protein LOC100797980 [Glycine max] |
| Aradu.VIQ9S | 0.01 | putative ubiquitin-like-specific protease 1B-like isoform X2 [Glycine max]; IPR003653 (Peptidase C48, SUMO/Sentrin/Ubl1); GO:0006508 (proteolysis), GO:0008234 (cysteine-type peptidase activity) |
| Aradu.VQR51 | 0.02 | putative ubiquitin-like-specific protease 1B-like isoform X3 [Glycine max] |
| Aradu.Y23I7 | 0.14 | Unknown protein |
| Aradu.YE99Z | 0.02 | zinc finger MYM-type protein 1-like [Glycine max]; IPR008906 (HAT dimerisation domain, C-terminal), IPR012337 (Ribonuclease H-like domain), IPR025398 (Domain of unknown function DUF4371); GO:0003676 (nucleic acid binding), GO:0046983 (protein dimerization activity) |
| Aradu.YWN27 | 0.04 | cellulose synthase 1 |
| Araip.1PE3B | 0.02 | Acyl-ACP thioesterase; IPR002864 (Acyl-ACP thioesterase); GO:0006633 (fatty acid biosynthetic process), GO:0016790 (thiolester hydrolase activity) |
| Araip.2AL3W | 0.08 | hypothetical protein |
| Araip.2MY4B | 0.21 | uncharacterized mitochondrial protein AtMg00810-like [Glycine max] |
| Araip.37Z3P | 0.01 | lycopene cyclase; IPR001461 (Aspartic peptidase), IPR008671 (Lycopene cyclase-type, FAD-binding), IPR021109 (Aspartic peptidase domain); GO:0004190 (aspartic-type endopeptidase activity), GO:0006508 (proteolysis), GO:0016117 (carotenoid biosynthetic process) |
| Araip.3S52C | 0.01 | serine/threonine-protein phosphatase 7 long form homolog [Glycine max]; IPR019557 (Aminotransferase-like, plant mobile domain) |
| Araip.3X0BY | 0.07 | ATP-dependent Clp protease proteolytic subunit-related protein 2, chloroplastic-like [Glycine max] |
| Araip.532HJ | 0.88 | Unknown protein |
| Araip.55PMS | 0.01 | lycopene cyclase; IPR001461 (Aspartic peptidase), IPR008671 (Lycopene cyclase-type, FAD-binding), IPR021109 (Aspartic peptidase domain); GO:0004190 (aspartic-type endopeptidase activity), GO:0006508 (proteolysis), GO:0016117 (carotenoid biosynthetic process) |
| Araip.5Z8W5 | 0.01 | uncharacterized protein LOC100776940 isoform X4 [Glycine max]; IPR004332 (Transposase, MuDR, plant) |
| Araip.61DA2 | 0.09 | Unknown protein |
| Araip.6QJ24 | 0.05 | F-box protein At1g30790-like [Glycine max] |
| Araip.6U5T6 | 0.14 | uncharacterized protein LOC100792053 isoform X3 [Glycine max] |
| Araip.763PP | 0.02 | protein FAR1-RELATED SEQUENCE 6-like isoform X2 [Glycine max]; IPR004330 (FAR1 DNA binding domain), IPR007527 (Zinc finger, SWIM-type); GO:0008270 (zinc ion binding) |
| Araip.89YCF | 0.05 | MATE efflux family protein |
| Araip.94GSC | 0.01 | protein FAR1-RELATED SEQUENCE 3-like isoform X1 [Glycine max]; IPR001878 (Zinc finger, CCHC-type), IPR004330 (FAR1 DNA binding domain); GO:0003676 (nucleic acid binding), GO:0008270 (zinc ion binding) |
| Araip.9E4VV | 0.09 | Unknown protein |
| Araip.A1C73 | 0.01 | Unknown protein |
| Araip.A31GS | 0.05 | Unknown protein |
| Araip.A32JN | 0.05 | Unknown protein |
| Araip.A66SI | 0.06 | ribulose bisphosphate carboxylase/oxygenase activase |
| Araip.AH9LW | 0.04 | SIGNAL PEPTIDE PEPTIDASE-LIKE 2; IPR007369 (Peptidase A22B, signal peptide peptidase); GO:0004190 (aspartic-type endopeptidase activity), GO:0016021 (integral component of membrane) |
| Araip.BFR2E | 0.03 | Unknown protein |
| Araip.BJ9AI | 0.08 | Unknown protein |
| Araip.C3S32 | 0.04 | Calcium-dependent lipid-binding (CaLB domain) family protein; IPR000008 (C2 domain); GO:0005515 (protein binding) |
| Araip.D56H1 | 0.03 | protein FAR1-RELATED SEQUENCE 6-like isoform X2 [Glycine max] |
| Araip.DB6BJ | 0.1 | CBL-interacting protein kinase 12 |
| Araip.DY0FE | 0.03 | serine/threonine-protein phosphatase 7 long form homolog [Glycine max]; IPR019557 (Aminotransferase-like, plant mobile domain) |
| Araip.EN1NQ | 0.05 | casein kinase 1-like protein 2; IPR011009 (Protein kinase-like domain) |
| Araip.FSM7V | 0.04 | Unknown protein |
| Araip.GN339 | 0.05 | Unknown protein; IPR010666 (Zinc finger, GRF-type); GO:0008270 (zinc ion binding) |
| Araip.H4S2W | 0.01 | zinc finger MYM-type protein 1-like [Glycine max]; IPR008906 (HAT dimerisation domain, C-terminal), IPR012337 (Ribonuclease H-like domain), IPR025398 (Domain of unknown function DUF4371); GO:0003676 (nucleic acid binding), GO:0046983 (protein dimerization activity) |
| Araip.H93F8 | 0.04 | uncharacterized protein LOC100779146 [Glycine max] |
| Araip.HW1D0 | 0.04 | uncharacterized protein LOC100807790 [Glycine max] |
| Araip.IK8G1 | 2.5 | F-box protein SKIP2-like isoform X1 [Glycine max]; IPR006553 (Leucine-rich repeat, cysteine-containing subtype) |
| Araip.J6FGQ | 1.52 | Acyl-ACP thioesterase; IPR002864 (Acyl-ACP thioesterase); GO:0006633 (fatty acid biosynthetic process), GO:0016790 (thiolester hydrolase activity) |
| Araip.J6WQD | 0.05 | Unknown protein |
| Araip.KDI4V | 0.04 | Zinc knuckle family protein n=1 Tax=Oryza sativa subsp. japonica RepID=H2KWL4_ORYSJ; IPR000588 (Peptidase A3A, cauliflower mosaic virus); GO:0004190 (aspartic-type endopeptidase activity), GO:0006508 (proteolysis) |
| Araip.LF479 | 0.03 | disease resistance protein (TIR-NBS-LRR class); IPR000767 (Disease resistance protein), IPR001611 (Leucine-rich repeat); GO:0005515 (protein binding), GO:0006952 (defense response) |
| Araip.ML8KN | 0.07 | Unknown protein |
| Araip.NH2AL | 0.03 | 3-ketoacyl-CoA synthase 2; IPR012392 (Very-long-chain 3-ketoacyl-CoA synthase), IPR016039 (Thiolase-like); GO:0003824 (catalytic activity), GO:0006633 (fatty acid biosynthetic process), GO:0008152 (metabolic process), GO:0008610 (lipid biosynthetic process), GO:0016020 (membrane) |
| Araip.PU45X | 0.01 | RNA-directed DNA polymerase homolog [Glycine max]; IPR000477 (Reverse transcriptase domain), IPR001878 (Zinc finger, CCHC-type); GO:0003676 (nucleic acid binding), GO:0003723 (RNA binding), GO:0003964 (RNA-directed DNA polymerase activity), GO:0006278 (RNA-dependent DNA replication), GO:0008270 (zinc ion binding) |
| Araip.S1J7V | 0.04 | receptor-like kinase, putative |
| Araip.S69XP | 0.04 | aluminum-activated malate transporter 1; IPR020966 (Aluminum-activated malate transporter); GO:0015743 (malate transport) |
| Araip.SJD5D | 0.04 | Protein kinase superfamily protein; IPR011009 (Protein kinase-like domain); GO:0004672 (protein kinase activity), GO:0004713 (protein tyrosine kinase activity), GO:0005524 (ATP binding), GO:0006468 (protein phosphorylation) |
| Araip.SX501 | 0.01 | serine/threonine-protein phosphatase 7 long form homolog [Glycine max]; IPR019557 (Aminotransferase-like, plant mobile domain) |
| Araip.T0PNM | 0.12 | uncharacterized protein LOC100780312 [Glycine max] |
| Araip.T80ZY | 0.8 | Cytochrome P450 superfamily protein; IPR001128 (Cytochrome P450); GO:0005506 (iron ion binding), GO:0020037 (heme binding), GO:0055114 (oxidation-reduction process) |
| Araip.U0NDB | 0.03 | Unknown protein |
| Araip.U86MN | 0.09 | Unknown protein |
| Araip.UWI6X | 0.04 | transmembrane amino acid transporter family protein; IPR013057 (Amino acid transporter, transmembrane) |
| Araip.V01CQ | 0.02 | pumilio-family RNA-binding repeatprotein; IPR016024 (Armadillo-type fold); GO:0003723 (RNA binding), GO:0005488 (binding) |
| Araip.V3KD6 | 0.02 | thyroid receptor-interacting protein; IPR027417 (P-loop containing nucleoside triphosphate hydrolase); GO:0005524 (ATP binding) |
| Araip.WS5R5 | 0.01 | uncharacterized protein LOC100776940 isoform X2 [Glycine max]; IPR004332 (Transposase, MuDR, plant) |
| Araip.X4GY5 | 0.09 | potassium channel SKOR-like [Glycine max]; IPR014710 (RmlC-like jelly roll fold) |
| Araip.Z1DGP | 0.06 | Unknown protein |
| Araip.Z68X5 | 0.04 | protein FAR-RED IMPAIRED RESPONSE 1-like isoform X4 [Glycine max]; IPR004330 (FAR1 DNA binding domain) |
| Gynoecium |  |  |
| Aradu.028CZ | 0.09 | Unknown protein |
| Aradu.06F34 | 0.08 | Unknown protein |
| Aradu.09G3F | 0.04 | Unknown protein |
| Aradu.0BJ82 | 0.16 | nuclear factor Y, subunit C1; IPR009072 (Histone-fold), IPR027170 (Transcriptional activator NFYC/HAP5 subunit); GO:0003700 (sequence-specific DNA binding transcription factor activity), GO:0005622 (intracellular), GO:0016602 (CCAAT-binding factor complex), GO:0043565 (sequence-specific DNA binding), GO:0046982 (protein heterodimerization activity) |
| Aradu.0L0FQ | 0.02 | Unknown protein |
| Aradu.0N6KW | 0.02 | receptor-like serine/threonine kinase 2; IPR011009 (Protein kinase-like domain), IPR013320 (Concanavalin A-like lectin/glucanase, subgroup), IPR021820 (S-locus receptor kinase, C-terminal); GO:0004672 (protein kinase activity), GO:0004674 (protein serine/threonine kinase activity), GO:0005524 (ATP binding), GO:0006468 (protein phosphorylation) |
| Aradu.124FF | 1.52 | Regulator of chromosome condensation (RCC1) family protein; IPR009091 (Regulator of chromosome condensation 1/beta-lactamase-inhibitor protein II), IPR013083 (Zinc finger, RING/FYVE/PHD-type), IPR013591 (Brevis radix (BRX) domain); GO:0046872 (metal ion binding) |
| Aradu.13CZ7 | 0.04 | transmembrane protein 147 [Glycine max]; IPR019164 (Protein of unknown function DUF2053, membrane) |
| Aradu.15269 | 0.04 | imidazoleglycerol-phosphate dehydratase; IPR000807 (Imidazoleglycerol-phosphate dehydratase); GO:0000105 (histidine biosynthetic process), GO:0004424 (imidazoleglycerol-phosphate dehydratase activity) |
| Aradu.1EH62 | 0.02 | disease resistance protein |
| Aradu.1GX6U | 0.06 | Unknown protein |
| Aradu.1HD8G | 0.07 | ATP synthase F1, alpha subunit; IPR005294 (ATPase, F1 complex, alpha subunit), IPR023366 (ATP synthase subunit alpha-like domain), IPR027417 (P-loop containing nucleoside triphosphate hydrolase); GO:0005524 (ATP binding), GO:0015986 (ATP synthesis coupled proton transport), GO:0015991 (ATP hydrolysis coupled proton transport), GO:0015992 (proton transport), GO:0046034 (ATP metabolic process) |
| Aradu.1UD2A | 0.02 | Unknown protein |
| Aradu.212BB | 0.02 | Helicase-like protein n=1 Tax=Medicago truncatula RepID=G7JLV8_MEDTR; IPR025476 (Helitron helicase-like domain) |
| Aradu.26WH0 | 0.01 | phosphoenolpyruvate carboxylase 1; IPR021135 (Phosphoenolpyruvate carboxylase); GO:0003824 (catalytic activity), GO:0006099 (tricarboxylic acid cycle), GO:0008964 (phosphoenolpyruvate carboxylase activity), GO:0015977 (carbon fixation) |
| Aradu.2DE36 | 0.43 | Unknown protein |
| Aradu.2I9AA | 0.02 | Unknown protein |
| Aradu.2SF5Z | 0.07 | ATP synthase F1, alpha subunit; IPR005294 (ATPase, F1 complex, alpha subunit), IPR023366 (ATP synthase subunit alpha-like domain), IPR027417 (P-loop containing nucleoside triphosphate hydrolase); GO:0005524 (ATP binding), GO:0015986 (ATP synthesis coupled proton transport), GO:0015991 (ATP hydrolysis coupled proton transport), GO:0015992 (proton transport), GO:0046034 (ATP metabolic process) |
| Aradu.34JF1 | 0.03 | Unknown protein |
| Aradu.36Y67 | 0.01 | Unknown protein |
| Aradu.38L7X | 0.05 | Helicase-like protein n=1 Tax=Oryza sativa subsp. japonica RepID=Q9AYF0_ORYSJ |
| Aradu.39J25 | 0.02 | serine/threonine-protein phosphatase 7 long form homolog [Glycine max]; IPR019557 (Aminotransferase-like, plant mobile domain) |
| Aradu.3A2RD | 0.04 | potassium transporter 1; IPR003855 (K+ potassium transporter); GO:0015079 (potassium ion transmembrane transporter activity), GO:0016020 (membrane), GO:0071805 (potassium ion transmembrane transport) |
| Aradu.3AF5S | 0.01 | Flavin-binding monooxygenase family protein; IPR013027 (FAD-dependent pyridine nucleotide-disulphide oxidoreductase), IPR020946 (Flavin monooxygenase-like); GO:0016491 (oxidoreductase activity), GO:0050660 (flavin adenine dinucleotide binding), GO:0050661 (NADP binding), GO:0055114 (oxidation-reduction process) |
| Aradu.3B6WB | 0.02 | Unknown protein |
| Aradu.3C1S7 | 0.16 | n=1 Tax=Oryza sativa subsp. japonica RepID=Q7F971_ORYSJ; IPR007527 (Zinc finger, SWIM-type); GO:0008270 (zinc ion binding) |
| Aradu.3P8PP | 0.01 | Zinc knuckle family protein n=1 Tax=Oryza sativa subsp. japonica RepID=H2KW38_ORYSJ; IPR001878 (Zinc finger, CCHC-type); GO:0003676 (nucleic acid binding), GO:0008270 (zinc ion binding) |
| Aradu.47T7Y | 0.01 | CTV.20 n=1 Tax=Poncirus trifoliata RepID=Q8H6Q8_PONTR |
| Aradu.54TTJ | 0.38 | n=1 Tax=Oryza sativa subsp. japonica RepID=Q7XTA1_ORYSJ; IPR019557 (Aminotransferase-like, plant mobile domain) |
| Aradu.5BN1V | 0.01 | Disease resistance protein (TIR-NBS-LRR class) family; IPR027417 (P-loop containing nucleoside triphosphate hydrolase) |
| Aradu.5CT6A | 0.01 | Sentrin-specific protease n=1 Tax=Morus notabilis RepID=W9QIH0_9ROSA |
| Aradu.5I8MP | 0.42 | uncharacterized protein LOC100778164 isoform X4 [Glycine max]; IPR027417 (P-loop containing nucleoside triphosphate hydrolase); GO:0005524 (ATP binding) |
| Aradu.5M40A | 0.08 | Unknown protein |
| Aradu.5R0P3 | 0.1 | 50S ribosomal protein L20; IPR005813 (Ribosomal protein L20); GO:0003735 (structural constituent of ribosome), GO:0005622 (intracellular), GO:0005840 (ribosome), GO:0006412 (translation), GO:0019843 (rRNA binding) |
| Aradu.68MP1 | 0.01 | DUF223 domain protein; IPR012340 (Nucleic acid-binding, OB-fold) |
| Aradu.6L1PR | 0.06 | uncharacterized protein LOC100780025 isoform 1 [Glycine max] |
| Aradu.6NR88 | 0.05 | General transcription factor 2-related zinc finger protein |
| Aradu.72XP3 | 0.1 | autophagy 3 (APG3) protein; IPR019461 (Autophagy-related protein 3, C-terminal) |
| Aradu.78PP9 | 0.01 | vacuolar protein sorting-associated protein 51 homolog [Glycine max] |
| Aradu.791XB | 0.08 | pentatricopeptide (PPR) repeat-containing protein |
| Aradu.79P2D | 0.29 | Unknown protein |
| Aradu.79SZB | 0.08 | Unknown protein |
| Aradu.7E510 | 0.01 | Helicase-like protein n=1 Tax=Oryza sativa subsp. japonica RepID=Q5NAA4_ORYSJ; IPR025476 (Helitron helicase-like domain) |
| Aradu.7N2U0 | 0.15 | xylose isomerase family protein; IPR001998 (Xylose isomerase), IPR013022 (Xylose isomerase-like, TIM barrel domain); GO:0005975 (carbohydrate metabolic process), GO:0009045 (xylose isomerase activity) |
| Aradu.7P61C | 0.02 | protein FAR1-RELATED SEQUENCE 6-like isoform X2 [Glycine max]; IPR004330 (FAR1 DNA binding domain), IPR006564 (Zinc finger, PMZ-type); GO:0008270 (zinc ion binding) |
| Aradu.7P7KH | 0.01 | zinc finger MYM-type protein 1-like [Glycine max]; IPR006580 (Zinc finger, TTF-type), IPR008906 (HAT dimerisation domain, C-terminal), IPR012337 (Ribonuclease H-like domain), IPR025398 (Domain of unknown function DUF4371); GO:0003676 (nucleic acid binding), GO:0046983 (protein dimerization activity) |
| Aradu.7X6Q2 | 0.05 | Unknown protein |
| Aradu.83GR3 | 0.03 | serine/threonine-protein phosphatase 7 long form homolog [Glycine max]; IPR019557 (Aminotransferase-like, plant mobile domain) |
| Aradu.87D0W | 0.01 | uncharacterized protein LOC100777165 [Glycine max] |
| Aradu.889PJ | 0.01 | Werner syndrome-like exonuclease; IPR012337 (Ribonuclease H-like domain); GO:0003676 (nucleic acid binding), GO:0006139 (nucleobase-containing compound metabolic process), GO:0008408 (3'-5' exonuclease activity) |
| Aradu.8D436 | 0.01 | uncharacterized protein LOC102670101 [Glycine max]; IPR001878 (Zinc finger, CCHC-type), IPR007527 (Zinc finger, SWIM-type); GO:0003676 (nucleic acid binding), GO:0008270 (zinc ion binding) |
| Aradu.8J2W9 | 0.05 | uncharacterized protein LOC100801254 [Glycine max] |
| Aradu.91TJN | 0.06 | serine/arginine repetitive matrix protein 2-like [Glycine max] |
| Aradu.9IJ7D | 0.03 | alcohol dehydrogenase 1; IPR002085 (Alcohol dehydrogenase superfamily, zinc-type), IPR011032 (GroES (chaperonin 10)-like), IPR016040 (NAD(P)-binding domain); GO:0008270 (zinc ion binding), GO:0016491 (oxidoreductase activity), GO:0055114 (oxidation-reduction process) |
| Aradu.AK6DG | 0.07 | Unknown protein |
| Aradu.AL65U | 0.08 | uncharacterized protein LOC100793882 isoform X2 [Glycine max]; IPR008546 (Domain of unknown function DUF828) |
| Aradu.B7QUS | 0.02 | FAR1 DNA-binding domain protein; IPR004330 (FAR1 DNA binding domain) |
| Aradu.BE5FD | 0.02 | pectinesterase 11; IPR011050 (Pectin lyase fold/virulence factor); GO:0005618 (cell wall), GO:0030599 (pectinesterase activity), GO:0042545 (cell wall modification) |
| Aradu.BH0PN | 0.07 | Unknown protein |
| Aradu.BJ6ZM | 0.01 | 17.6 kDa class II heat shock protein; IPR008978 (HSP20-like chaperone) |
| Aradu.BQF2S | 0.01 | arabinose kinase; IPR012347 (Ferritin-related) |
| Aradu.BV9FL | 0.25 | glyoxal oxidase-related protein; IPR011043 (Galactose oxidase/kelch, beta-propeller), IPR014756 (Immunoglobulin E-set), IPR015916 (Galactose oxidase, beta-propeller) |
| Aradu.BW6UM | 0.05 | myo-inositol oxygenase 2; IPR007828 (Inositol oxygenase); GO:0005506 (iron ion binding), GO:0005737 (cytoplasm), GO:0019310 (inositol catabolic process), GO:0050113 (inositol oxygenase activity), GO:0055114 (oxidation-reduction process) |
| Aradu.C4FQ7 | 0.05 | zinc finger MYM-type protein 1-like [Glycine max] |
| Aradu.C4R9R | 0.02 | Helicase-like protein n=1 Tax=Medicago truncatula RepID=G7JLV8_MEDTR; IPR025476 (Helitron helicase-like domain) |
| Aradu.C90E9 | 0.43 | F-box family protein; IPR001810 (F-box domain), IPR017451 (F-box associated interaction domain); GO:0005515 (protein binding) |
| Aradu.C9C0U | 0.18 | Integral membrane family protein n=1 Tax=Populus trichocarpa RepID=B9GRX8_POPTR; IPR005828 (General substrate transporter); GO:0016020 (membrane), GO:0016021 (integral component of membrane), GO:0022857 (transmembrane transporter activity), GO:0022891 (substrate-specific transmembrane transporter activity), GO:0055085 (transmembrane transport) |
| Aradu.CGX2S | 0.02 | animal RPA1 domain protein |
| Aradu.CK3ER | 0.01 | zinc finger MYM-type protein 1-like [Glycine max]; IPR025398 (Domain of unknown function DUF4371) |
| Aradu.CM8M9 | 0.02 | CASP-like protein RCOM_0864260-like [Glycine max]; IPR006702 (Uncharacterised protein family UPF0497, trans-membrane plant) |
| Aradu.D1PNR | 0.31 | protein LURP-one-related 15-like [Glycine max]; IPR025659 (Tubby C-terminal-like domain) |
| Aradu.D2DT6 | 0.02 | flocculation protein FLO11-like isoform X5 [Glycine max] |
| Aradu.DA9XS | 0.06 | probable polygalacturonase non-catalytic subunit JP650-like [Glycine max] |
| Aradu.DI3FC | 0.52 | Helicase-like protein n=1 Tax=Medicago truncatula RepID=G7JN81_MEDTR |
| Aradu.DP9RG | 0.31 | serine/threonine-protein phosphatase 7 long form homolog [Glycine max]; IPR019557 (Aminotransferase-like, plant mobile domain) |
| Aradu.DW8SS | 0.01 | Unknown protein |
| Aradu.E8J8G | 0.01 | Serine/Threonine kinase family protein; IPR000858 (S-locus glycoprotein), IPR001480 (Bulb-type lectin domain), IPR003609 (Apple-like), IPR011009 (Protein kinase-like domain), IPR013320 (Concanavalin A-like lectin/glucanase, subgroup), IPR024171 (S-receptor-like serine/threonine-protein kinase); GO:0004672 (protein kinase activity), GO:0004674 (protein serine/threonine kinase activity), GO:0005524 (ATP binding), GO:0006468 (protein phosphorylation), GO:0048544 (recognition of pollen) |
| Aradu.E9LNV | 0.52 | Stigma-specific Stig1 family protein; IPR006969 (Stigma-specific protein Stig1) |
| Aradu.E9PDP | 0.01 | Unknown protein |
| Aradu.EFU60 | 0.01 | probable serine/threonine-protein kinase At1g01540-like [Glycine max] |
| Aradu.EV6XS | 0.01 | protein FAR1-RELATED SEQUENCE 6-like isoform X2 [Glycine max]; IPR004330 (FAR1 DNA binding domain), IPR007527 (Zinc finger, SWIM-type); GO:0008270 (zinc ion binding) |
| Aradu.EW4PA | 0.08 | Unknown protein |
| Aradu.F30AF | 0.03 | WAT1-related protein At1g68170-like [Glycine max] |
| Aradu.F52UG | 0.01 | Unknown protein |
| Aradu.FH7D1 | 0.01 | Helicase-like protein n=1 Tax=Medicago truncatula RepID=G7KZ57_MEDTR |
| Aradu.FIA6S | 0.01 | serine/threonine-protein phosphatase 7 long form homolog [Glycine max]; IPR019557 (Aminotransferase-like, plant mobile domain) |
| Aradu.FVA73 | 0.03 | serine/threonine-protein phosphatase 7 long form homolog [Glycine max]; IPR019557 (Aminotransferase-like, plant mobile domain) |
| Aradu.G03BZ | 0.02 | Unknown protein |
| Aradu.G7NA8 | 0.02 | Zinc knuckle family protein n=1 Tax=Oryza sativa subsp. japonica RepID=H2KW38_ORYSJ; IPR017946 (PLC-like phosphodiesterase, TIM beta/alpha-barrel domain); GO:0006629 (lipid metabolic process), GO:0008081 (phosphoric diester hydrolase activity) |
| Aradu.GBV5L | 0.05 | Unknown protein |
| Aradu.GC3CE | 0.05 | cation/H+ exchanger 3; IPR006153 (Cation/H+ exchanger); GO:0006812 (cation transport), GO:0015299 (solute:hydrogen antiporter activity), GO:0016021 (integral component of membrane), GO:0055085 (transmembrane transport) |
| Aradu.GJG0V | 0.09 | Unknown protein |
| Aradu.GJM9T | 0.01 | serine/threonine-protein phosphatase 7 long form homolog [Glycine max]; IPR019557 (Aminotransferase-like, plant mobile domain) |
| Aradu.GPS6C | 0.03 | Unknown protein |
| Aradu.GV7TV | 0.04 | zinc finger MYM-type protein 1-like [Glycine max] |
| Aradu.H0CQM | 0.08 | animal RPA1 domain protein |
| Aradu.H14CC | 0.36 | serine/threonine-protein phosphatase 7 long form homolog [Glycine max]; IPR019557 (Aminotransferase-like, plant mobile domain) |
| Aradu.HI9D6 | 0.06 | serine/threonine-protein phosphatase 7 long form homolog [Glycine max]; IPR019557 (Aminotransferase-like, plant mobile domain) |
| Aradu.HPU9C | 0.02 | replication protein A 70 kDa DNA-binding subunit C-like [Glycine max]; IPR012340 (Nucleic acid-binding, OB-fold) |
| Aradu.HQ4AU | 0.01 | arabinose kinase |
| Aradu.HR8W0 | 0.01 | Helicase-like protein n=1 Tax=Medicago truncatula RepID=G7KPQ9_MEDTR |
| Aradu.HW3QJ | 0.02 | Rer1 family protein; IPR004932 (Retrieval of early ER protein Rer1); GO:0016021 (integral component of membrane) |
| Aradu.I7143 | 0.01 | dentin sialophosphoprotein-like isoform X2 [Glycine max] |
| Aradu.IJ8ZV | 0.03 | serine/threonine-protein phosphatase 7 long form homolog [Glycine max]; IPR019557 (Aminotransferase-like, plant mobile domain) |
| Aradu.IPX9R | 0.02 | Unknown protein |
| Aradu.ISZ54 | 0.7 | Unknown protein |
| Aradu.IVG3B | 0.22 | diacylglycerol kinase 4; IPR000756 (Diacylglycerol kinase, accessory domain), IPR001206 (Diacylglycerol kinase, catalytic domain), IPR016064 (ATP-NAD kinase-like domain); GO:0003951 (NAD+ kinase activity), GO:0004143 (diacylglycerol kinase activity), GO:0007205 (protein kinase C-activating G-protein coupled receptor signaling pathway), GO:0008152 (metabolic process) |
| Aradu.J8127 | 0.01 | Transposon protein, putative, Mutator sub-class n=1 Tax=Oryza sativa subsp. japonica RepID=Q2R3P6_ORYSJ |
| Aradu.JE3HH | 0.02 | glycine-rich RNA-binding protein 7 [Glycine max]; IPR012677 (Nucleotide-binding, alpha-beta plait); GO:0000166 (nucleotide binding), GO:0003676 (nucleic acid binding) |
| Aradu.JH2AX | 0.04 | Unknown protein |
| Aradu.JP5P4 | 1.05 | Protein phosphatase 2A regulatory B subunit family protein; IPR002554 (Protein phosphatase 2A, regulatory B subunit, B56), IPR016024 (Armadillo-type fold); GO:0000159 (protein phosphatase type 2A complex), GO:0005488 (binding), GO:0007165 (signal transduction), GO:0008601 (protein phosphatase type 2A regulator activity) |
| Aradu.JQ81V | 1.27 | Flavin-binding monooxygenase family protein; IPR013027 (FAD-dependent pyridine nucleotide-disulphide oxidoreductase), IPR020946 (Flavin monooxygenase-like); GO:0016491 (oxidoreductase activity), GO:0050660 (flavin adenine dinucleotide binding), GO:0050661 (NADP binding), GO:0055114 (oxidation-reduction process) |
| Aradu.JQX38 | 0.03 | serine/threonine-protein phosphatase 7 long form homolog [Glycine max]; IPR019557 (Aminotransferase-like, plant mobile domain) |
| Aradu.K2Q8R | 0.01 | serine/threonine-protein phosphatase 7 long form homolog [Glycine max]; IPR019557 (Aminotransferase-like, plant mobile domain) |
| Aradu.K41GB | 0.1 | Core-2/I-branching beta-1,6-N-acetylglucosaminyltransferase family protein; IPR003406 (Glycosyl transferase, family 14); GO:0008375 (acetylglucosaminyltransferase activity), GO:0016020 (membrane) |
| Aradu.K79IH | 0.04 | n=1 Tax=Oryza sativa subsp. japonica RepID=Q7F971_ORYSJ; IPR001878 (Zinc finger, CCHC-type), IPR007527 (Zinc finger, SWIM-type); GO:0003676 (nucleic acid binding), GO:0008270 (zinc ion binding) |
| Aradu.KFD45 | 0.37 | serine/threonine-protein phosphatase 7 long form homolog [Glycine max]; IPR019557 (Aminotransferase-like, plant mobile domain) |
| Aradu.KH93E | 0.01 | Unknown protein |
| Aradu.KL0CI | 0.02 | triose-phosphate transporter family protein; IPR004332 (Transposase, MuDR, plant) |
| Aradu.KX16C | 0.01 | cinnamyl alcohol dehydrogenase; IPR009071 (High mobility group box domain) |
| Aradu.KY80N | 0.02 | Unknown protein |
| Aradu.L2S9S | 0.06 | F-box family protein |
| Aradu.L5N5V | 0.02 | serine/threonine-protein phosphatase 7 long form homolog [Glycine max]; IPR019557 (Aminotransferase-like, plant mobile domain) |
| Aradu.LA3GQ | 0.05 | 30S ribosomal protein S4 |
| Aradu.LG0Z0 | 1.17 | Vacuole, catalytic n=1 Tax=Theobroma cacao RepID=UPI00042B20B5; IPR023346 (Lysozyme-like domain) |
| Aradu.LJI6C | 0.01 | PfpI family intracellular protease n=3 Tax=Desulfovibrio RepID=D9YED7_9DELT; IPR002818 (ThiJ/PfpI) |
| Aradu.LQ319 | 0.06 | CASP-like protein RCOM_0864260-like [Glycine max]; IPR006702 (Uncharacterised protein family UPF0497, trans-membrane plant) |
| Aradu.LV7AT | 0.67 | unknown protein; Has 1807 Blast hits to 1807 proteins in 277 species: Archae - 0; Bacteria - 0; Metazoa - 736; Fungi - 347; Plants - 385; Viruses - 0; Other Eukaryotes - 339 (source: NCBI BLink). |
| Aradu.LWL4V | 0.01 | Unknown protein |
| Aradu.LWV1C | 0.01 | Unknown protein |
| Aradu.M34BL | 0.1 | serine/threonine-protein phosphatase 7 long form homolog [Glycine max]; IPR019557 (Aminotransferase-like, plant mobile domain) |
| Aradu.M4ANC | 0.1 | replication protein A 70 kDa DNA-binding subunit C-like [Glycine max]; IPR012340 (Nucleic acid-binding, OB-fold) |
| Aradu.M5LK1 | 1.32 | SCP1-like small phosphatase 5; IPR004274 (NLI interacting factor), IPR023214 (HAD-like domain); GO:0005515 (protein binding), GO:0016791 (phosphatase activity) |
| Aradu.MU9I1 | 0.03 | Helicase-like protein n=1 Tax=Oryza sativa subsp. japonica RepID=Q5NAA4_ORYSJ; IPR025476 (Helitron helicase-like domain) |
| Aradu.N8XWL | 0.01 | zinc finger MYM-type protein 1-like [Glycine max]; IPR006580 (Zinc finger, TTF-type), IPR008906 (HAT dimerisation domain, C-terminal), IPR012337 (Ribonuclease H-like domain); GO:0003676 (nucleic acid binding), GO:0046983 (protein dimerization activity) |
| Aradu.N93Y5 | 0.39 | MBOAT (membrane bound O-acyl transferase) family protein; IPR004299 (Membrane bound O-acyl transferase, MBOAT) |
| Aradu.NHH15 | 0.02 | Far-red impaired responsive (FAR1) family protein |
| Aradu.NVS60 | 0.04 | Helicase-like protein n=1 Tax=Medicago truncatula RepID=G7JLV8_MEDTR; IPR025476 (Helitron helicase-like domain) |
| Aradu.QJ36I | 0.01 | serine/threonine-protein phosphatase 7 long form homolog [Glycine max]; IPR019557 (Aminotransferase-like, plant mobile domain) |
| Aradu.QJV7F | 0.07 | Helicase-like protein n=1 Tax=Medicago truncatula RepID=G7KPQ9_MEDTR; IPR025476 (Helitron helicase-like domain) |
| Aradu.QVC80 | 0.01 | mitochondrial ribosomal protein S14; IPR001209 (Ribosomal protein S14); GO:0003735 (structural constituent of ribosome), GO:0005622 (intracellular), GO:0005840 (ribosome), GO:0006412 (translation) |
| Aradu.QW7SF | 0.01 | putative disease resistance protein At3g14460-like [Glycine max]; IPR027417 (P-loop containing nucleoside triphosphate hydrolase) |
| Aradu.QWA84 | 0.02 | Unknown protein |
| Aradu.R5I7F | 0.02 | hypothetical protein |
| Aradu.R65GB | 0.17 | Unknown protein |
| Aradu.R8WW5 | 0.01 | Aluminium induced protein with YGL and LRDR motifs; IPR024286 (Domain of unknown function DUF3700) |
| Aradu.RRT07 | 0.28 | serine/threonine-protein phosphatase 7 long form homolog [Glycine max]; IPR019557 (Aminotransferase-like, plant mobile domain) |
| Aradu.S0NBF | 0.03 | organic cation/carnitine transporter 3; IPR005828 (General substrate transporter), IPR016196 (Major facilitator superfamily domain, general substrate transporter); GO:0005215 (transporter activity), GO:0006810 (transport), GO:0016020 (membrane), GO:0016021 (integral component of membrane), GO:0022857 (transmembrane transporter activity), GO:0055085 (transmembrane transport) |
| Aradu.S1Z3N | 0.01 | Unknown protein |
| Aradu.S3RY9 | 0.01 | serine/threonine-protein phosphatase 7 long form homolog [Glycine max]; IPR019557 (Aminotransferase-like, plant mobile domain) |
| Aradu.S6FWL | 0.29 | pantothenate kinase |
| Aradu.S7GME | 0.01 | Polyprotein n=1 Tax=Citrus endogenous pararetrovirus RepID=V9QEM3_9RETR |
| Aradu.SCC75 | 0.34 | annexin 5; IPR001464 (Annexin); GO:0005509 (calcium ion binding), GO:0005544 (calcium-dependent phospholipid binding) |
| Aradu.SD13M | 0.02 | serine/threonine-protein phosphatase 7 long form homolog [Glycine max]; IPR019557 (Aminotransferase-like, plant mobile domain) |
| Aradu.SR8AZ | 0.03 | animal RPA1 domain protein; IPR012340 (Nucleic acid-binding, OB-fold) |
| Aradu.SS4HP | 0.02 | FAR1 DNA-binding domain protein |
| Aradu.SYQ5F | 0.04 | cytosolic endo-beta-N-acetylglucosaminidase-like [Glycine max] |
| Aradu.T0LCK | 0.01 | Unknown protein |
| Aradu.T3SFT | 0.03 | RING-H2 zinc finger protein; IPR013083 (Zinc finger, RING/FYVE/PHD-type); GO:0005515 (protein binding), GO:0008270 (zinc ion binding) |
| Aradu.TC9Z0 | 0.01 | Cytochrome P450 superfamily protein; IPR001128 (Cytochrome P450); GO:0005506 (iron ion binding), GO:0020037 (heme binding), GO:0055114 (oxidation-reduction process) |
| Aradu.TJU8V | 0.02 | myosin 1; IPR027417 (P-loop containing nucleoside triphosphate hydrolase) |
| Aradu.U551K | 0.01 | villin-3-like isoform X4 [Glycine max]; IPR005516 (Remorin, C-terminal) |
| Aradu.U6EGF | 1.92 | uncharacterized protein At4g13230-like [Glycine max] |
| Aradu.U6ZP1 | 0.07 | glutamate receptor 2.7; IPR001638 (Extracellular solute-binding protein, family 3); GO:0005215 (transporter activity), GO:0006810 (transport) |
| Aradu.UD5Y2 | 0.08 | Myb/SANT-like DNA-binding domain protein; IPR024752 (Myb/SANT-like domain) |
| Aradu.UKK4J | 0.02 | hypothetical protein |
| Aradu.VM1WJ | 0.06 | ribosomal protein S15A; IPR000630 (Ribosomal protein S8); GO:0003735 (structural constituent of ribosome), GO:0005840 (ribosome), GO:0006412 (translation) |
| Aradu.VMW9M | 0.15 | sucrose-proton symporter 2 |
| Aradu.VVQ2N | 2.5 | early nodulin-like protein 1-like [Glycine max]; IPR008972 (Cupredoxin); GO:0005507 (copper ion binding), GO:0009055 (electron carrier activity) |
| Aradu.W01HH | 0.01 | serine/threonine-protein phosphatase 7 long form homolog [Glycine max]; IPR019557 (Aminotransferase-like, plant mobile domain) |
| Aradu.W0CU1 | 0.01 | serine/threonine-protein phosphatase 7 long form homolog [Glycine max]; IPR019557 (Aminotransferase-like, plant mobile domain) |
| Aradu.W37S8 | 0.02 | Unknown protein |
| Aradu.W3Q5D | 0.03 | Unknown protein |
| Aradu.W4FHL | 0.02 | Unknown protein |
| Aradu.W6UE0 | 0.04 | DNA-directed RNA polymerase subunit alpha; IPR009025 (DNA-directed RNA polymerase, RBP11-like dimerisation domain), IPR011262 (DNA-directed RNA polymerase, insert domain); GO:0003899 (DNA-directed RNA polymerase activity), GO:0046983 (protein dimerization activity) |
| Aradu.W7RDY | 0.01 | PLANT CADMIUM RESISTANCE 2; IPR006461 (Uncharacterised protein family Cys-rich) |
| Aradu.WC7AP | 0.02 | n=2 Tax=Oryza sativa subsp. japonica RepID=Q7XTP7_ORYSJ; IPR004332 (Transposase, MuDR, plant) |
| Aradu.WF2JA | 0.06 | protein FAR1-RELATED SEQUENCE 6-like isoform X2 [Glycine max]; IPR004330 (FAR1 DNA binding domain), IPR007527 (Zinc finger, SWIM-type); GO:0008270 (zinc ion binding) |
| Aradu.WI0UI | 0.02 | Flavin-binding monooxygenase family protein; IPR013027 (FAD-dependent pyridine nucleotide-disulphide oxidoreductase), IPR020946 (Flavin monooxygenase-like); GO:0016491 (oxidoreductase activity), GO:0050660 (flavin adenine dinucleotide binding), GO:0050661 (NADP binding), GO:0055114 (oxidation-reduction process) |
| Aradu.WV4DB | 0.01 | uncharacterized protein LOC100797259 isoform X4 [Glycine max] |
| Aradu.X06N9 | 0.01 | Unknown protein |
| Aradu.X31AQ | 0.01 | Polyprotein n=1 Tax=Citrus endogenous pararetrovirus RepID=V9QEM3_9RETR; IPR001878 (Zinc finger, CCHC-type); GO:0003676 (nucleic acid binding), GO:0008270 (zinc ion binding) |
| Aradu.XFA8C | 0.08 | Unknown protein |
| Aradu.XTE0X | 0.02 | receptor kinase 2; IPR011009 (Protein kinase-like domain); GO:0004672 (protein kinase activity), GO:0005524 (ATP binding), GO:0006468 (protein phosphorylation) |
| Aradu.Y20S8 | 0.02 | CHD3-type chromatin-remodeling factor PICKLE n=1 Tax=Morus notabilis RepID=W9QJP0_9ROSA; IPR019557 (Aminotransferase-like, plant mobile domain) |
| Aradu.Y243E | 0.07 | Unknown protein |
| Aradu.Y4KCH | 0.01 | serine/threonine-protein phosphatase 7 long form homolog [Glycine max]; IPR019557 (Aminotransferase-like, plant mobile domain) |
| Aradu.YBE0E | 0.03 | Unknown protein |
| Aradu.YJV80 | 0.03 | Polyprotein n=5 Tax=Citrus endogenous pararetrovirus RepID=V9QF33_9RETR |
| Aradu.YR6P3 | 0.01 | CTV.20 n=1 Tax=Poncirus trifoliata RepID=Q8H6Q8_PONTR; IPR001878 (Zinc finger, CCHC-type); GO:0003676 (nucleic acid binding), GO:0008270 (zinc ion binding) |
| Aradu.YRE5W | 0.04 | F-box/LRR protein |
| Aradu.YT6UC | 0.03 | Helicase-like protein n=1 Tax=Medicago truncatula RepID=G7KZ57_MEDTR; IPR025476 (Helitron helicase-like domain) |
| Aradu.Z0S2T | 0.03 | Bifunctional inhibitor/lipid-transfer protein/seed storage 2S albumin superfamily protein; IPR016140 (Bifunctional inhibitor/plant lipid transfer protein/seed storage helical domain) |
| Aradu.Z2S9N | 0.05 | Unknown protein |
| Aradu.Z9J76 | 0.09 | myo-inositol oxygenase 2; IPR007828 (Inositol oxygenase); GO:0005506 (iron ion binding), GO:0005737 (cytoplasm), GO:0019310 (inositol catabolic process), GO:0050113 (inositol oxygenase activity), GO:0055114 (oxidation-reduction process) |
| Aradu.ZUF8Z | 0.07 | Unknown protein |
| Araip.01M9M | 0.05 | nucleolin 1-like [Glycine max] |
| Araip.0475C | 0.01 | dehydrodolichyl diphosphate synthase 2-like [Glycine max]; IPR001441 (Decaprenyl diphosphate synthase-like) |
| Araip.060IS | 0.01 | wall-associated receptor kinase galacturonan-binding protein |
| Araip.06L93 | 0.03 | NAD(P)H-quinone oxidoreductase subunit H; IPR001135 (NADH-quinone oxidoreductase, subunit D); GO:0048038 (quinone binding), GO:0051287 (NAD binding), GO:0055114 (oxidation-reduction process) |
| Araip.084F0 | 0.03 | n=1 Tax=Oryza sativa subsp. japonica RepID=Q7XU62_ORYSJ; IPR004332 (Transposase, MuDR, plant) |
| Araip.0B2Q0 | 0.03 | CHD3-type chromatin-remodeling factor pickle protein; IPR016197 (Chromo domain-like) |
| Araip.0CJ5Z | 0.02 | Unknown protein |
| Araip.0G6YQ | 0.01 | Ulp1 protease family, carboxy-terminal domain protein |
| Araip.0PS9V | 0.02 | Unknown protein |
| Araip.0SA1A | 0.01 | Helicase-like protein n=1 Tax=Medicago truncatula RepID=G7KPQ9_MEDTR; IPR025476 (Helitron helicase-like domain) |
| Araip.0X0FS | 0.03 | viral movement protein |
| Araip.1022A | 0.02 | protein n=1 Tax=Oryza sativa subsp. japonica RepID=C7J0D1_ORYSJ; IPR001878 (Zinc finger, CCHC-type), IPR007527 (Zinc finger, SWIM-type); GO:0003676 (nucleic acid binding), GO:0008270 (zinc ion binding) |
| Araip.15BWN | 0.03 | Helicase-like protein n=1 Tax=Medicago truncatula RepID=G7JLV8_MEDTR |
| Araip.1QI05 | 0.05 | RNA polymerase beta subunit n=12 Tax=Fragaria RepID=J7IFR4_9ROSA |
| Araip.25MMJ | 0.02 | serine/threonine-protein phosphatase 7 long form homolog [Glycine max]; IPR019557 (Aminotransferase-like, plant mobile domain) |
| Araip.25Z0N | 0.04 | GRF zinc finger protein; IPR010666 (Zinc finger, GRF-type); GO:0008270 (zinc ion binding) |
| Araip.2ER8H | 0.01 | Unknown protein |
| Araip.2Q8QC | 1.35 | Unknown protein |
| Araip.2TM4W | 0.03 | Cysteine proteinases superfamily protein; IPR000668 (Peptidase C1A, papain C-terminal); GO:0006508 (proteolysis), GO:0008234 (cysteine-type peptidase activity) |
| Araip.2Z8TE | 0.03 | GRF zinc finger protein; IPR010666 (Zinc finger, GRF-type); GO:0008270 (zinc ion binding) |
| Araip.33FK6 | 0.02 | protein FAR1-RELATED SEQUENCE 5-like [Glycine max]; IPR004330 (FAR1 DNA binding domain) |
| Araip.34GI7 | 0.01 | Unknown protein |
| Araip.36Q9K | 0.02 | protein FAR1-RELATED SEQUENCE 5-like isoform X4 [Glycine max]; IPR004330 (FAR1 DNA binding domain) |
| Araip.39BPZ | 0.02 | Membrane transporter D1 n=3 Tax=Andropogoneae RepID=B6U4Q3_MAIZE; IPR005828 (General substrate transporter), IPR016196 (Major facilitator superfamily domain, general substrate transporter); GO:0016020 (membrane), GO:0016021 (integral component of membrane), GO:0022857 (transmembrane transporter activity), GO:0022891 (substrate-specific transmembrane transporter activity), GO:0055085 (transmembrane transport) |
| Araip.39E6W | 0.12 | GDP-mannose transporter GONST3 |
| Araip.39FPQ | 0.03 | Myb/SANT-like DNA-binding domain protein |
| Araip.3C72S | 0.03 | protein FAR-RED IMPAIRED RESPONSE 1-like isoform X4 [Glycine max]; IPR004330 (FAR1 DNA binding domain) |
| Araip.3F6YU | 0.02 | Unknown protein |
| Araip.3J0BB | 0.06 | serine/threonine-protein phosphatase 7 long form homolog [Glycine max]; IPR019557 (Aminotransferase-like, plant mobile domain) |
| Araip.3Z393 | 0.01 | zinc finger MYM-type protein 1-like [Glycine max] |
| Araip.40KRS | 0.04 | protein FAR1-RELATED SEQUENCE 6-like isoform X2 [Glycine max]; IPR004330 (FAR1 DNA binding domain) |
| Araip.43M64 | 0.03 | Unknown protein |
| Araip.456FU | 0.01 | aspartic proteinase A1; IPR001461 (Aspartic peptidase), IPR021109 (Aspartic peptidase domain); GO:0004190 (aspartic-type endopeptidase activity), GO:0006508 (proteolysis) |
| Araip.4FD5V | 0.14 | F-box family protein; IPR001810 (F-box domain), IPR017451 (F-box associated interaction domain); GO:0005515 (protein binding) |
| Araip.50YEL | 0.09 | golgin candidate 5 |
| Araip.51DXI | 0.01 | uncharacterized protein LOC100778822 isoform X6 [Glycine max]; IPR004252 (Probable transposase, Ptta/En/Spm, plant) |
| Araip.531CJ | 0.01 | Ulp1 protease family, carboxy-terminal domain protein; IPR003653 (Peptidase C48, SUMO/Sentrin/Ubl1); GO:0006508 (proteolysis), GO:0008234 (cysteine-type peptidase activity) |
| Araip.543RJ | 0.33 | putative F-box/LRR-repeat protein At1g56400-like [Glycine max] |
| Araip.54GWQ | 0.01 | receptor-like protein kinase 1; IPR011009 (Protein kinase-like domain); GO:0004672 (protein kinase activity), GO:0005524 (ATP binding), GO:0006468 (protein phosphorylation) |
| Araip.57Z7B | 0.02 | Aminotransferase-like, plant mobile domain family protein |
| Araip.58NXY | 0.1 | uncharacterized protein LOC102660787 [Glycine max] |
| Araip.5CJ03 | 0.1 | serine/threonine-protein phosphatase 7 long form homolog [Glycine max]; IPR019557 (Aminotransferase-like, plant mobile domain) |
| Araip.5JS0F | 0.01 | NF-kappa-B-activating protein n=2 Tax=Medicago truncatula RepID=G7KT11_MEDTR; IPR009269 (Protein of unknown function DUF926) |
| Araip.5M86B | 0.17 | Unknown protein |
| Araip.5R0BQ | 0.01 | NLI interacting factor-like phosphatase; IPR023214 (HAD-like domain) |
| Araip.6026H | 0.05 | Gibberellin-regulated family protein; IPR003854 (Gibberellin regulated protein) |
| Araip.60VHT | 0.05 | serine/threonine-protein phosphatase 7 long form homolog [Glycine max]; IPR019557 (Aminotransferase-like, plant mobile domain) |
| Araip.615VA | 0.06 | F-box protein interaction domain protein; IPR001810 (F-box domain), IPR011043 (Galactose oxidase/kelch, beta-propeller), IPR017451 (F-box associated interaction domain); GO:0005515 (protein binding) |
| Araip.628MR | 0.05 | Unknown protein |
| Araip.62LNZ | 0.01 | protein FAR1-RELATED SEQUENCE 6-like isoform X2 [Glycine max]; IPR004330 (FAR1 DNA binding domain) |
| Araip.6344A | 1.91 | ubiquitin-60S ribosomal protein L40-like isoform X1 [Glycine max]; IPR001975 (Ribosomal protein L40e); GO:0003735 (structural constituent of ribosome), GO:0005840 (ribosome), GO:0006412 (translation) |
| Araip.696HX | 0.02 | disease resistance protein (TIR-NBS-LRR class), putative |
| Araip.6B1QX | 0.01 | lecithin:cholesterol acyltransferase 3; IPR003386 (Lecithin:cholesterol/phospholipid:diacylglycerol acyltransferase); GO:0006629 (lipid metabolic process), GO:0008374 (O-acyltransferase activity) |
| Araip.6CS90 | 0.03 | Unknown protein |
| Araip.6RA6D | 0.01 | Expressed protein n=2 Tax=Oryza sativa subsp. japonica RepID=Q2R6F5_ORYSJ |
| Araip.6T0FQ | 1.29 | SCP1-like small phosphatase 5; IPR004274 (NLI interacting factor), IPR023214 (HAD-like domain); GO:0005515 (protein binding), GO:0016791 (phosphatase activity) |
| Araip.6W3XL | 0.03 | DHHC-type zinc finger protein |
| Araip.6Z5BQ | 0.06 | n=1 Tax=Oryza sativa subsp. japonica RepID=Q7F971_ORYSJ; IPR004332 (Transposase, MuDR, plant), IPR007527 (Zinc finger, SWIM-type); GO:0008270 (zinc ion binding) |
| Araip.706JC | 0.02 | magnesium transporter 7; IPR026573 (Magnesium transporter MRS2/LPE10); GO:0015095 (magnesium ion transmembrane transporter activity), GO:0015693 (magnesium ion transport) |
| Araip.714D4 | 1.04 | DNA-directed RNA polymerase III subunit RPC6-like [Glycine max]; IPR016049 (RNA polymerase Rpc34-like); GO:0003677 (DNA binding), GO:0003899 (DNA-directed RNA polymerase activity) |
| Araip.71ZJG | 0.12 | Unknown protein |
| Araip.76MVR | 0.01 | uncharacterized protein LOC100794770 isoform X2 [Glycine max] |
| Araip.787IL | 0.02 | serine/threonine-protein phosphatase 7 long form homolog [Glycine max]; IPR019557 (Aminotransferase-like, plant mobile domain) |
| Araip.7U8X4 | 0.02 | uncharacterized protein LOC102666784 [Glycine max]; IPR001878 (Zinc finger, CCHC-type), IPR007527 (Zinc finger, SWIM-type); GO:0003676 (nucleic acid binding), GO:0008270 (zinc ion binding) |
| Araip.7X4B0 | 0.46 | Cell wall protein EXP2 n=1 Tax=Mirabilis jalapa RepID=Q84L40_MIRJA; IPR007118 (Expansin/Lol pI); GO:0005576 (extracellular region), GO:0009664 (plant-type cell wall organization) |
| Araip.81VGS | 0.08 | putative ubiquitin-like-specific protease 1B-like isoform X3 [Glycine max]; IPR003653 (Peptidase C48, SUMO/Sentrin/Ubl1); GO:0006508 (proteolysis), GO:0008234 (cysteine-type peptidase activity) |
| Araip.85R94 | 0.27 | Unknown protein |
| Araip.877XD | 3.03 | Unknown protein |
| Araip.89ZIQ | 0.01 | disease resistance protein (TIR-NBS-LRR class) |
| Araip.8CP42 | 0.01 | Membrane transporter D1 n=3 Tax=Andropogoneae RepID=B6U4Q3_MAIZE; IPR005828 (General substrate transporter), IPR016196 (Major facilitator superfamily domain, general substrate transporter); GO:0016020 (membrane), GO:0016021 (integral component of membrane), GO:0022857 (transmembrane transporter activity), GO:0022891 (substrate-specific transmembrane transporter activity), GO:0055085 (transmembrane transport) |
| Araip.8W3UX | 0.08 | GDSL esterase/lipase n=1 Tax=Medicago truncatula RepID=G7JRD0_MEDTR |
| Araip.8Y945 | 0.01 | protein FAR1-RELATED SEQUENCE 5-like [Glycine max]; IPR001878 (Zinc finger, CCHC-type), IPR007527 (Zinc finger, SWIM-type); GO:0003676 (nucleic acid binding), GO:0008270 (zinc ion binding) |
| Araip.913NI | 0.01 | protein FAR1-RELATED SEQUENCE 6-like isoform X2 [Glycine max] |
| Araip.96R4L | 0.02 | ankyrin repeat-containing protein At5g02620-like isoform X6 [Glycine max]; IPR020683 (Ankyrin repeat-containing domain) |
| Araip.9702U | 0.04 | uncharacterized protein LOC102659605 [Glycine max]; IPR019557 (Aminotransferase-like, plant mobile domain) |
| Araip.978UR | 0.01 | serine/threonine-protein phosphatase 7 long form homolog [Glycine max]; IPR019557 (Aminotransferase-like, plant mobile domain) |
| Araip.9DY03 | 1.34 | uncharacterized protein At4g19900-like [Glycine max] |
| Araip.9ES73 | 0.21 | replication factor-A carboxy-terminal domain protein; IPR012340 (Nucleic acid-binding, OB-fold) |
| Araip.9S80S | 0.66 | uncharacterized protein LOC102666302 [Glycine max]; IPR004332 (Transposase, MuDR, plant) |
| Araip.9V022 | 0.06 | Unknown protein |
| Araip.A2GBD | 1.27 | nuclear factor Y, subunit C4; IPR009072 (Histone-fold), IPR027170 (Transcriptional activator NFYC/HAP5 subunit); GO:0003700 (sequence-specific DNA binding transcription factor activity), GO:0005622 (intracellular), GO:0016602 (CCAAT-binding factor complex), GO:0043565 (sequence-specific DNA binding), GO:0046982 (protein heterodimerization activity) |
| Araip.A4D56 | 0.03 | Unknown protein |
| Araip.A4RFV | 0.02 | putative nuclease HARBI1-like [Glycine max]; IPR026103 (Harbinger transposase-derived nuclease, animal) |
| Araip.A7EFG | 0.04 | Unknown protein; IPR015300 (DNA-binding pseudobarrel domain); GO:0003677 (DNA binding) |
| Araip.AD1AX | 0.01 | protein FAR1-RELATED SEQUENCE 7-like isoform X2 [Glycine max]; IPR004330 (FAR1 DNA binding domain) |
| Araip.AJ9GQ | 0.06 | Unknown protein |
| Araip.B25LT | 0.06 | Unknown protein |
| Araip.B3IE4 | 0.84 | unknown protein; Has 30201 Blast hits to 17322 proteins in 780 species: Archae - 12; Bacteria - 1396; Metazoa - 17338; Fungi - 3422; Plants - 5037; Viruses - 0; Other Eukaryotes - 2996 (source: NCBI BLink). |
| Araip.B4AQG | 0.05 | serine/threonine-protein phosphatase 7 long form homolog [Glycine max]; IPR019557 (Aminotransferase-like, plant mobile domain) |
| Araip.B4QSP | 0.02 | GRF zinc finger protein; IPR010666 (Zinc finger, GRF-type); GO:0008270 (zinc ion binding) |
| Araip.B5RUJ | 0.5 | Membrane transporter D1 n=3 Tax=Andropogoneae RepID=B6U4Q3_MAIZE; IPR005828 (General substrate transporter), IPR016196 (Major facilitator superfamily domain, general substrate transporter); GO:0016020 (membrane), GO:0016021 (integral component of membrane), GO:0022857 (transmembrane transporter activity), GO:0022891 (substrate-specific transmembrane transporter activity), GO:0055085 (transmembrane transport) |
| Araip.B6N3Z | 0.01 | Membrane transporter D1 n=3 Tax=Andropogoneae RepID=B6U4Q3_MAIZE; IPR005828 (General substrate transporter), IPR016196 (Major facilitator superfamily domain, general substrate transporter); GO:0005215 (transporter activity), GO:0006810 (transport), GO:0016020 (membrane), GO:0016021 (integral component of membrane), GO:0022857 (transmembrane transporter activity), GO:0022891 (substrate-specific transmembrane transporter activity), GO:0055085 (transmembrane transport) |
| Araip.BB229 | 0.02 | replication factor-A carboxy-terminal domain protein; IPR012340 (Nucleic acid-binding, OB-fold) |
| Araip.BJM1C | 0.9 | Protein kinase superfamily protein; IPR011009 (Protein kinase-like domain); GO:0004672 (protein kinase activity), GO:0005524 (ATP binding), GO:0006468 (protein phosphorylation) |
| Araip.BR21C | 0.01 | FAR1 DNA-binding domain protein |
| Araip.BS5A9 | 0.02 | Unknown protein |
| Araip.BZ9T9 | 0.07 | Unknown protein |
| Araip.C1TPS | 0.01 | uncharacterized protein LOC100785875 isoform X4 [Glycine max]; IPR024752 (Myb/SANT-like domain) |
| Araip.C1YUH | 0.04 | Unknown protein |
| Araip.C21PK | 0.03 | protein YLS7-like [Glycine max]; IPR026057 (PC-Esterase) |
| Araip.C2Y44 | 0.01 | RNA recognition motif (RRM) containing protein |
| Araip.C70U7 | 0.09 | E3 ubiquitin-protein ligase RING1-like [Glycine max]; IPR010543 (Domain of unknown function DUF1117) |
| Araip.C7CXI | 0.01 | protein FAR1-RELATED SEQUENCE 6-like isoform X2 [Glycine max]; IPR007527 (Zinc finger, SWIM-type); GO:0008270 (zinc ion binding) |
| Araip.CB2PZ | 0.01 | F-box protein interaction domain protein |
| Araip.CEV93 | 2.13 | Unknown protein |
| Araip.CF1QH | 0.04 | uncharacterized protein At1g04910-like isoform X1 [Glycine max]; IPR019378 (GDP-fucose protein O-fucosyltransferase) |
| Araip.CKZ00 | 0.02 | Unknown protein |
| Araip.D1704 | 0.05 | GRF zinc finger protein; IPR010666 (Zinc finger, GRF-type); GO:0008270 (zinc ion binding) |
| Araip.D5D0J | 0.03 | serine/threonine-protein phosphatase 7 long form homolog [Glycine max]; IPR019557 (Aminotransferase-like, plant mobile domain) |
| Araip.D9LDJ | 0.5 | Membrane trafficking VPS53 family protein isoform 8 n=1 Tax=Theobroma cacao RepID=UPI00042B0A8E |
| Araip.DC04Q | 0.3 | Unknown protein |
| Araip.DDU95 | 0.01 | Unknown protein |
| Araip.DI6UQ | 0.05 | Unknown protein |
| Araip.DLJ10 | 0.03 | protein YLS7-like [Glycine max]; IPR026057 (PC-Esterase) |
| Araip.DP7U9 | 4.87 | Unknown protein |
| Araip.E1AYP | 0.02 | Helicase-like protein n=1 Tax=Medicago truncatula RepID=G7KPQ9_MEDTR; IPR025476 (Helitron helicase-like domain) |
| Araip.E2JUR | 0.04 | protein FAR1-RELATED SEQUENCE 5-like [Glycine max]; IPR004330 (FAR1 DNA binding domain), IPR006564 (Zinc finger, PMZ-type); GO:0008270 (zinc ion binding) |
| Araip.E64SW | 0.21 | F-box family protein; IPR001810 (F-box domain), IPR017451 (F-box associated interaction domain); GO:0005515 (protein binding) |
| Araip.EJ34Y | 0.02 | probable pectinesterase/pectinesterase inhibitor 21-like [Glycine max]; IPR006501 (Pectinesterase inhibitor domain), IPR011050 (Pectin lyase fold/virulence factor); GO:0004857 (enzyme inhibitor activity), GO:0005618 (cell wall), GO:0030599 (pectinesterase activity), GO:0042545 (cell wall modification) |
| Araip.F1AUY | 0.03 | replication factor-A carboxy-terminal domain protein; IPR012340 (Nucleic acid-binding, OB-fold) |
| Araip.F24QZ | 0.01 | auxin response factor 2 |
| Araip.F3SNX | 0.09 | Regulator of chromosome condensation (RCC1) family protein; IPR009091 (Regulator of chromosome condensation 1/beta-lactamase-inhibitor protein II), IPR013083 (Zinc finger, RING/FYVE/PHD-type), IPR013591 (Brevis radix (BRX) domain); GO:0046872 (metal ion binding) |
| Araip.F7DS8 | 0.03 | Unknown protein |
| Araip.FN2VP | 0.06 | Unknown protein |
| Araip.FP2CV | 0.01 | disease resistance protein (TIR-NBS-LRR class), putative; IPR000157 (Toll/interleukin-1 receptor homology (TIR) domain); GO:0005515 (protein binding), GO:0007165 (signal transduction) |
| Araip.FU2CT | 0.04 | Unknown protein |
| Araip.FU7EH | 1.71 | Unknown protein |
| Araip.FUY4P | 0.02 | cellulose synthase family protein; IPR005150 (Cellulose synthase); GO:0016020 (membrane), GO:0016760 (cellulose synthase (UDP-forming) activity), GO:0030244 (cellulose biosynthetic process) |
| Araip.FUZ7T | 0.98 | thaumatin-like protein 3; IPR001938 (Thaumatin) |
| Araip.FVW1W | 0.04 | uncharacterized protein LOC102662924 [Glycine max]; IPR001878 (Zinc finger, CCHC-type), IPR007527 (Zinc finger, SWIM-type); GO:0003676 (nucleic acid binding), GO:0008270 (zinc ion binding) |
| Araip.G4TXZ | 0.02 | serine/threonine-protein phosphatase 7 long form homolog [Glycine max]; IPR019557 (Aminotransferase-like, plant mobile domain) |
| Araip.G6LTE | 0.01 | F-box/LRR-repeat protein 13-like isoform X2 [Glycine max] |
| Araip.G8VKU | 0.42 | protein LURP-one-related 15-like [Glycine max]; IPR025659 (Tubby C-terminal-like domain) |
| Araip.GF0NL | 0.02 | GRF zinc finger protein; IPR010666 (Zinc finger, GRF-type); GO:0008270 (zinc ion binding) |
| Araip.GJZ40 | 0.03 | 30S ribosomal protein S7; IPR000235 (Ribosomal protein S5/S7), IPR023798 (Ribosomal protein S7 domain); GO:0006412 (translation) |
| Araip.GPJ3R | 0.02 | zinc finger MYM-type protein 1-like [Glycine max]; IPR008906 (HAT dimerisation domain, C-terminal), IPR012337 (Ribonuclease H-like domain); GO:0003676 (nucleic acid binding), GO:0046983 (protein dimerization activity) |
| Araip.GUU9C | 0.45 | n=1 Tax=Oryza sativa subsp. japonica RepID=Q7XU62_ORYSJ |
| Araip.GWN6X | 0.01 | putative ubiquitin-like-specific protease 1B-like isoform X2 [Glycine max]; IPR003653 (Peptidase C48, SUMO/Sentrin/Ubl1); GO:0006508 (proteolysis), GO:0008234 (cysteine-type peptidase activity) |
| Araip.H0PL5 | 0.01 | FAR-RED impaired response-like protein; IPR004330 (FAR1 DNA binding domain) |
| Araip.HJM90 | 0.01 | Unknown protein |
| Araip.HM8KV | 0.01 | protein FAR1-RELATED SEQUENCE 6-like isoform X2 [Glycine max]; IPR006564 (Zinc finger, PMZ-type); GO:0008270 (zinc ion binding) |
| Araip.HP29F | 0.02 | LRR receptor-like kinase; IPR011009 (Protein kinase-like domain) |
| Araip.HSH1H | 0.01 | protein FAR1-RELATED SEQUENCE 5-like [Glycine max]; IPR004330 (FAR1 DNA binding domain), IPR007527 (Zinc finger, SWIM-type); GO:0008270 (zinc ion binding) |
| Araip.HVB78 | 0.03 | GDP-Man:Man(3)GlcNAc(2)-PP-Dol alpha-1,2-mannosyltransferase-like [Glycine max] |
| Araip.HW0UX | 0.01 | Unknown protein |
| Araip.I7335 | 0.01 | uncharacterized protein LOC100820019 isoform X4 [Glycine max] |
| Araip.I7QPL | 0.01 | Zinc knuckle family protein n=1 Tax=Oryza sativa subsp. japonica RepID=H2KW38_ORYSJ |
| Araip.I87AY | 0.06 | Unknown protein |
| Araip.IFI83 | 0.38 | serine/threonine-protein phosphatase 7 long form homolog [Glycine max]; IPR019557 (Aminotransferase-like, plant mobile domain) |
| Araip.IJ45R | 0.01 | Disease resistance protein (TIR-NBS-LRR class) family |
| Araip.INL03 | 0.04 | Ycf1 protein chrC:123884-129244 REVERSE; IPR008896 (Uncharacterised protein family Ycf1) |
| Araip.J2W2Z | 0.02 | nucleolin 1-like [Glycine max] |
| Araip.J404K | 0.1 | Ulp1 protease family, carboxy-terminal domain protein |
| Araip.J6QNL | 0.01 | Unknown protein |
| Araip.JJ6RA | 0.35 | F-box protein interaction domain protein; IPR017451 (F-box associated interaction domain) |
| Araip.JVL4R | 1.92 | uncharacterized protein At4g13230-like [Glycine max] |
| Araip.K2P5I | 0.02 | protein FAR1-RELATED SEQUENCE 5-like [Glycine max]; IPR004330 (FAR1 DNA binding domain) |
| Araip.KPY6F | 0.03 | hypothetical protein |
| Araip.KSG9E | 0.01 | serine/threonine-protein phosphatase 7 long form homolog [Glycine max]; IPR019557 (Aminotransferase-like, plant mobile domain) |
| Araip.L179T | 0.45 | Far-red impaired responsive (FAR1) family protein; IPR004330 (FAR1 DNA binding domain) |
| Araip.L2S5R | 0.02 | uncharacterized protein LOC100527109 [Glycine max] |
| Araip.L6JUW | 0.01 | protein FAR1-RELATED SEQUENCE 3-like isoform X2 [Glycine max]; IPR001878 (Zinc finger, CCHC-type), IPR006564 (Zinc finger, PMZ-type); GO:0003676 (nucleic acid binding), GO:0008270 (zinc ion binding) |
| Araip.LNG41 | 0.02 | Unknown protein |
| Araip.LS3K4 | 0.01 | protein FAR-RED IMPAIRED RESPONSE 1-like isoform X3 [Glycine max] |
| Araip.LV1DA | 0.3 | Membrane transporter D1 n=3 Tax=Andropogoneae RepID=B6U4Q3_MAIZE; IPR005828 (General substrate transporter), IPR016196 (Major facilitator superfamily domain, general substrate transporter); GO:0005215 (transporter activity), GO:0006810 (transport), GO:0016020 (membrane), GO:0016021 (integral component of membrane), GO:0022857 (transmembrane transporter activity), GO:0055085 (transmembrane transport) |
| Araip.M1KBX | 0.03 | ATP-dependent Clp protease |
| Araip.MD440 | 0.01 | blue copper protein-like [Glycine max]; IPR008972 (Cupredoxin); GO:0005507 (copper ion binding), GO:0009055 (electron carrier activity) |
| Araip.MH7E2 | 0.01 | protein FAR1-RELATED SEQUENCE 6-like isoform X2 [Glycine max]; IPR004330 (FAR1 DNA binding domain), IPR007527 (Zinc finger, SWIM-type); GO:0008270 (zinc ion binding) |
| Araip.MJ3PE | 0.01 | GRF zinc finger protein; IPR010666 (Zinc finger, GRF-type); GO:0008270 (zinc ion binding) |
| Araip.MW70S | 0.02 | Zinc knuckle family protein n=1 Tax=Oryza sativa subsp. japonica RepID=H2KWL4_ORYSJ; IPR001878 (Zinc finger, CCHC-type); GO:0003676 (nucleic acid binding), GO:0008270 (zinc ion binding) |
| Araip.MY8D9 | 0.06 | syntaxin-124-like [Glycine max]; IPR010989 (t-SNARE); GO:0005515 (protein binding), GO:0016020 (membrane), GO:0016192 (vesicle-mediated transport) |
| Araip.MYZ0C | 0.02 | myo-inositol oxygenase 4; IPR007828 (Inositol oxygenase); GO:0005506 (iron ion binding), GO:0005737 (cytoplasm), GO:0019310 (inositol catabolic process), GO:0050113 (inositol oxygenase activity), GO:0055114 (oxidation-reduction process) |
| Araip.N0EQW | 2.6 | myb transcription factor; IPR009057 (Homeodomain-like); GO:0003677 (DNA binding) |
| Araip.N548H | 0.01 | serine/threonine-protein phosphatase 7 long form homolog [Glycine max]; IPR019557 (Aminotransferase-like, plant mobile domain) |
| Araip.NDE9S | 0.11 | replication factor-A carboxy-terminal domain protein; IPR012340 (Nucleic acid-binding, OB-fold) |
| Araip.NK3CL | 0.03 | splicing regulatory glutamine/lysine-rich protein 1-like [Glycine max] |
| Araip.NPQ3A | 0.03 | hypothetical protein |
| Araip.NV0UD | 0.11 | Unknown protein |
| Araip.P020U | 0.01 | ATP sulfurylase 1; IPR002650 (Sulphate adenylyltransferase), IPR014729 (Rossmann-like alpha/beta/alpha sandwich fold), IPR015947 (PUA-like domain); GO:0000103 (sulfate assimilation), GO:0004781 (sulfate adenylyltransferase (ATP) activity) |
| Araip.P0E59 | 0.02 | transmembrane protein 194A-like [Glycine max] |
| Araip.P18YF | 0.04 | putative ubiquitin-like-specific protease 1B-like isoform X3 [Glycine max]; IPR003653 (Peptidase C48, SUMO/Sentrin/Ubl1); GO:0006508 (proteolysis), GO:0008234 (cysteine-type peptidase activity) |
| Araip.P4XV6 | 0.04 | Membrane transporter D1 n=3 Tax=Andropogoneae RepID=B6U4Q3_MAIZE; IPR005828 (General substrate transporter), IPR016196 (Major facilitator superfamily domain, general substrate transporter); GO:0016020 (membrane), GO:0016021 (integral component of membrane), GO:0022857 (transmembrane transporter activity), GO:0022891 (substrate-specific transmembrane transporter activity), GO:0055085 (transmembrane transport) |
| Araip.P551V | 0.09 | Unknown protein |
| Araip.P5YX2 | 0.08 | Unknown protein |
| Araip.P6FJH | 0.02 | Unknown protein |
| Araip.PA207 | 0.01 | ATP binding microtubule motor family protein; IPR001752 (Kinesin, motor domain), IPR027417 (P-loop containing nucleoside triphosphate hydrolase), IPR027640 (Kinesin-like protein); GO:0003777 (microtubule motor activity), GO:0005524 (ATP binding), GO:0005871 (kinesin complex), GO:0007018 (microtubule-based movement), GO:0008017 (microtubule binding) |
| Araip.PF4SQ | 0.05 | Transposon protein n=1 Tax=Arachis hypogaea RepID=G0Y6V7_ARAHY; IPR004330 (FAR1 DNA binding domain) |
| Araip.PH1Q4 | 0.28 | Helicase-like protein n=1 Tax=Medicago truncatula RepID=G7KPQ9_MEDTR; IPR025476 (Helitron helicase-like domain) |
| Araip.PKY4B | 2.62 | probable pectinesterase/pectinesterase inhibitor 41-like [Glycine max]; IPR011050 (Pectin lyase fold/virulence factor); GO:0005618 (cell wall), GO:0030599 (pectinesterase activity), GO:0042545 (cell wall modification) |
| Araip.PU0FS | 0.08 | protein FAR1-RELATED SEQUENCE 5-like [Glycine max]; IPR004330 (FAR1 DNA binding domain), IPR006564 (Zinc finger, PMZ-type); GO:0008270 (zinc ion binding) |
| Araip.Q39I8 | 0.03 | Unknown protein |
| Araip.Q40XG | 0.1 | protein FAR1-RELATED SEQUENCE 5-like [Glycine max] |
| Araip.Q86D1 | 0.04 | serine/threonine-protein phosphatase 7 long form homolog [Glycine max]; IPR019557 (Aminotransferase-like, plant mobile domain) |
| Araip.QN614 | 0.02 | Early-responsive to dehydration stress protein (ERD4); IPR003864 (Domain of unknown function DUF221); GO:0016020 (membrane) |
| Araip.QP1Y7 | 0.01 | Unknown protein |
| Araip.QP35G | 0.04 | uncharacterized protein LOC100780312 [Glycine max]; IPR012337 (Ribonuclease H-like domain); GO:0003676 (nucleic acid binding) |
| Araip.QR4LR | 0.03 | uncharacterized protein LOC100797259 isoform X3 [Glycine max]; IPR004332 (Transposase, MuDR, plant) |
| Araip.QT81S | 0.16 | protein FAR1-RELATED SEQUENCE 6-like isoform X2 [Glycine max]; IPR004330 (FAR1 DNA binding domain) |
| Araip.QV985 | 0.03 | Unknown protein |
| Araip.R08RK | 0.03 | uncharacterized protein LOC100808214 isoform X1 [Glycine max]; IPR007527 (Zinc finger, SWIM-type); GO:0008270 (zinc ion binding) |
| Araip.R3X9C | 0.03 | uncharacterized protein LOC100785875 isoform X2 [Glycine max]; IPR024752 (Myb/SANT-like domain), IPR026103 (Harbinger transposase-derived nuclease, animal) |
| Araip.RI4CM | 0.01 | Protein kinase superfamily protein; IPR011009 (Protein kinase-like domain); GO:0004672 (protein kinase activity), GO:0005524 (ATP binding), GO:0006468 (protein phosphorylation) |
| Araip.RK9DU | 0.01 | protein FAR1-RELATED SEQUENCE 6-like isoform X2 [Glycine max]; IPR004330 (FAR1 DNA binding domain), IPR007527 (Zinc finger, SWIM-type); GO:0008270 (zinc ion binding) |
| Araip.RQ6P2 | 0.01 | protein FAR1-RELATED SEQUENCE 5-like [Glycine max]; IPR004330 (FAR1 DNA binding domain), IPR007527 (Zinc finger, SWIM-type); GO:0008270 (zinc ion binding) |
| Araip.RWP2N | 0.08 | scarecrow-like protein 3-like [Glycine max]; IPR005202 (Transcription factor GRAS) |
| Araip.RZ3BX | 0.05 | uncharacterized protein LOC100782920 [Glycine max] |
| Araip.S04V5 | 0.05 | mRNA-decapping enzyme-like protein; IPR010334 (Dcp1-like decapping), IPR011993 (Pleckstrin homology-like domain) |
| Araip.S2880 | 0.58 | Regulator of chromosome condensation (RCC1) family protein; IPR009091 (Regulator of chromosome condensation 1/beta-lactamase-inhibitor protein II), IPR013083 (Zinc finger, RING/FYVE/PHD-type), IPR013591 (Brevis radix (BRX) domain); GO:0046872 (metal ion binding) |
| Araip.S2MNM | 0.03 | uncharacterized protein LOC100777206 isoform X3 [Glycine max]; IPR022227 (Protein of unknown function DUF3754) |
| Araip.S2RUD | 0.04 | protein FAR1-RELATED SEQUENCE 6-like isoform X2 [Glycine max] |
| Araip.S56XA | 3.96 | Late embryogenesis abundant protein (LEA) family protein |
| Araip.S8MSL | 0.02 | Ulp1 protease family, carboxy-terminal domain protein; IPR003653 (Peptidase C48, SUMO/Sentrin/Ubl1); GO:0006508 (proteolysis), GO:0008234 (cysteine-type peptidase activity) |
| Araip.SHD4Y | 0.09 | JHL23C09.8 protein n=1 Tax=Jatropha curcas RepID=E6NUA2_JATCU |
| Araip.SJR7X | 0.01 | cysteine synthase C1; IPR001926 (Tryptophan synthase beta subunit-like PLP-dependent enzymes superfamily) |
| Araip.SWW40 | 0.07 | Unknown protein |
| Araip.SZ2AK | 0.01 | uncharacterized protein LOC100797259 isoform X4 [Glycine max]; IPR007527 (Zinc finger, SWIM-type); GO:0008270 (zinc ion binding) |
| Araip.T0HX0 | 0.02 | protein FAR1-RELATED SEQUENCE 11-like [Glycine max]; IPR004330 (FAR1 DNA binding domain) |
| Araip.T2W2U | 1.3 | WD repeat-containing protein 44-like [Glycine max]; IPR015943 (WD40/YVTN repeat-like-containing domain), IPR020472 (G-protein beta WD-40 repeat); GO:0005515 (protein binding) |
| Araip.T4ENP | 0.12 | Helicase-like protein n=1 Tax=Oryza sativa subsp. japonica RepID=Q6YTQ6_ORYSJ |
| Araip.T7579 | 0.08 | F-box protein interaction domain protein |
| Araip.T9QAQ | 0.02 | E3 ubiquitin-protein ligase n=5 Tax=Solanum RepID=M1BDZ1_SOLTU |
| Araip.TG0L1 | 1.39 | Protein phosphatase 2A regulatory B subunit family protein; IPR002554 (Protein phosphatase 2A, regulatory B subunit, B56), IPR016024 (Armadillo-type fold); GO:0000159 (protein phosphatase type 2A complex), GO:0005488 (binding), GO:0007165 (signal transduction), GO:0008601 (protein phosphatase type 2A regulator activity) |
| Araip.TZG0T | 1.15 | Helicase-like protein n=1 Tax=Medicago truncatula RepID=G7KPQ9_MEDTR |
| Araip.U7W0W | 0.05 | serine/threonine-protein phosphatase 7 long form homolog [Glycine max]; IPR019557 (Aminotransferase-like, plant mobile domain) |
| Araip.U9BPI | 0.1 | F-box and associated interaction domains-containing protein; IPR001810 (F-box domain), IPR017451 (F-box associated interaction domain); GO:0005515 (protein binding) |
| Araip.UK5C1 | 0.6 | hypothetical protein |
| Araip.UNH79 | 0.01 | protein FAR1-RELATED SEQUENCE 11-like isoform X1 [Glycine max]; IPR004330 (FAR1 DNA binding domain) |
| Araip.V1RVE | 0.06 | Unknown protein |
| Araip.V20A0 | 0.03 | GDSL esterase/lipase n=1 Tax=Medicago truncatula RepID=G7JRD0_MEDTR |
| Araip.V23ZE | 0.02 | flowering locus protein T; IPR008914 (Phosphatidylethanolamine-binding protein PEBP) |
| Araip.V6F98 | 6.11 | unknown protein; FUNCTIONS IN: molecular_function unknown; INVOLVED IN: response to karrikin; LOCATED IN: endomembrane system; EXPRESSED IN: 23 plant structures; EXPRESSED DURING: 14 growth stages |
| Araip.VCC0R | 0.04 | DUF223 domain protein; IPR012340 (Nucleic acid-binding, OB-fold) |
| Araip.VP6D2 | 0.01 | Aminotransferase-like, plant mobile domain family protein; IPR019557 (Aminotransferase-like, plant mobile domain) |
| Araip.VRU7R | 0.02 | UDP-glucuronic acid decarboxylase 6-like isoform X3 [Glycine max] |
| Araip.VT9JT | 0.02 | Unknown protein |
| Araip.VZL0W | 0.13 | Unknown protein |
| Araip.W0J2L | 0.05 | organic cation/carnitine transporter 2; IPR016196 (Major facilitator superfamily domain, general substrate transporter) |
| Araip.W52DQ | 0.02 | 5-hydroxyisourate hydrolase; IPR018020 (Oxo-4-hydroxy-4-carboxy-5-ureidoimidazoline decarboxylase) |
| Araip.W5GFH | 0.03 | Unknown protein |
| Araip.W5PUF | 0.05 | PLANT CADMIUM RESISTANCE 2; IPR006461 (Uncharacterised protein family Cys-rich) |
| Araip.W6736 | 0.68 | Membrane transporter D1 n=3 Tax=Andropogoneae RepID=B6U4Q3_MAIZE; IPR005828 (General substrate transporter), IPR016196 (Major facilitator superfamily domain, general substrate transporter); GO:0016020 (membrane), GO:0016021 (integral component of membrane), GO:0022857 (transmembrane transporter activity), GO:0022891 (substrate-specific transmembrane transporter activity), GO:0055085 (transmembrane transport) |
| Araip.W7X5A | 0.02 | Unknown protein |
| Araip.W9PGD | 0.05 | myb transcription factor; IPR009057 (Homeodomain-like); GO:0003677 (DNA binding), GO:0003682 (chromatin binding) |
| Araip.WA0Y9 | 0.02 | Microtubule based kinesin motor protein n=1 Tax=Arabidopsis thaliana RepID=F4K4C5_ARATH; IPR001752 (Kinesin, motor domain), IPR027417 (P-loop containing nucleoside triphosphate hydrolase); GO:0003777 (microtubule motor activity), GO:0005524 (ATP binding), GO:0007018 (microtubule-based movement), GO:0008017 (microtubule binding) |
| Araip.WC9Z8 | 0.02 | Myb/SANT-like DNA-binding domain protein; IPR024752 (Myb/SANT-like domain) |
| Araip.WGY3Q | 0.02 | Unknown protein; IPR010666 (Zinc finger, GRF-type); GO:0008270 (zinc ion binding) |
| Araip.WV24P | 0.02 | Unknown protein |
| Araip.WY630 | 0.04 | hypothetical protein |
| Araip.X0LRL | 3.03 | Unknown protein |
| Araip.X3G2J | 0.01 | uncharacterized protein LOC100797259 isoform X3 [Glycine max]; IPR001878 (Zinc finger, CCHC-type), IPR007527 (Zinc finger, SWIM-type); GO:0003676 (nucleic acid binding), GO:0008270 (zinc ion binding) |
| Araip.X50BG | 0.03 | serine/threonine-protein phosphatase 7 long form homolog [Glycine max]; IPR019557 (Aminotransferase-like, plant mobile domain) |
| Araip.X9BIG | 0.97 | annexin 5; IPR001464 (Annexin); GO:0005509 (calcium ion binding), GO:0005544 (calcium-dependent phospholipid binding) |
| Araip.XCE6D | 0.02 | protein FAR1-RELATED SEQUENCE 4-like isoform X1 [Glycine max]; IPR004330 (FAR1 DNA binding domain) |
| Araip.XH5BY | 1.52 | serine/threonine-protein phosphatase 7 long form homolog [Glycine max]; IPR019557 (Aminotransferase-like, plant mobile domain) |
| Araip.XLN10 | 0.59 | Unknown protein |
| Araip.XT7ML | 0.03 | Zinc knuckle family protein n=1 Tax=Oryza sativa subsp. japonica RepID=H2KW38_ORYSJ |
| Araip.XY61G | 0.08 | protein FAR1-RELATED SEQUENCE 5-like [Glycine max]; IPR007527 (Zinc finger, SWIM-type); GO:0008270 (zinc ion binding) |
| Araip.Y0W2G | 1.36 | Calcium-binding EF-hand family protein; IPR004837 (Sodium/calcium exchanger membrane region); GO:0016021 (integral component of membrane), GO:0055085 (transmembrane transport) |
| Araip.Y2C9L | 0.01 | Zinc knuckle family protein n=1 Tax=Solanum demissum RepID=Q6L424_SOLDE; IPR001878 (Zinc finger, CCHC-type); GO:0003676 (nucleic acid binding), GO:0008270 (zinc ion binding) |
| Araip.Y62E3 | 0.04 | Protein kinase superfamily protein |
| Araip.Y6M5T | 0.36 | F-box family protein; IPR001810 (F-box domain), IPR002885 (Pentatricopeptide repeat), IPR017451 (F-box associated interaction domain); GO:0005515 (protein binding) |
| Araip.YJS7G | 0.02 | Unknown protein |
| Araip.YLT10 | 0.08 | Unknown protein |
| Araip.YN1IJ | 0.01 | protein FAR1-RELATED SEQUENCE 5-like [Glycine max]; IPR004330 (FAR1 DNA binding domain) |
| Araip.YQ272 | 1.18 | Regulator of chromosome condensation (RCC1) family protein; IPR009091 (Regulator of chromosome condensation 1/beta-lactamase-inhibitor protein II), IPR013083 (Zinc finger, RING/FYVE/PHD-type), IPR013591 (Brevis radix (BRX) domain); GO:0046872 (metal ion binding) |
| Araip.YSH4E | 0.04 | Unknown protein |
| Araip.Z28N2 | 0.02 | Unknown protein |
| Araip.Z5484 | 0.01 | uncharacterized protein LOC102664163 isoform X5 [Glycine max]; IPR004252 (Probable transposase, Ptta/En/Spm, plant) |
| Araip.Z5SSJ | 0.07 | boron transporter 4-like isoform X2 [Glycine max] |
| Araip.Z8YJ9 | 0.03 | Unknown protein |
| Araip.Z9LMA | 0.04 | Myb/SANT-like DNA-binding domain protein |
| Araip.ZKY1K | 0.03 | RING/U-box superfamily protein; IPR013083 (Zinc finger, RING/FYVE/PHD-type); GO:0046872 (metal ion binding) |
| Araip.ZMW3X | 0.01 | 30S ribosomal protein S4; IPR002942 (RNA-binding S4 domain); GO:0003723 (RNA binding) |
| Araip.ZR9MG | 0.1 | Unknown protein |
| Androecium |  |  |
| Aradu.0J322 | 0.01 | peptide transporter 1; IPR000109 (Proton-dependent oligopeptide transporter family), IPR016196 (Major facilitator superfamily domain, general substrate transporter); GO:0005215 (transporter activity), GO:0006810 (transport), GO:0016020 (membrane) |
| Aradu.391ER | 0.06 | CTV.20 n=1 Tax=Poncirus trifoliata RepID=Q8H6Q8_PONTR |
| Aradu.45R0N | 0.09 | Unknown protein |
| Aradu.4Z62M | 0.01 | Aminotransferase-like protein n=1 Tax=Oryza sativa subsp. japonica RepID=Q6K3L3_ORYSJ; IPR019557 (Aminotransferase-like, plant mobile domain) |
| Aradu.73UZR | 0.07 | Unknown protein |
| Aradu.B0E5H | 0.29 | protein FAR1-RELATED SEQUENCE 3-like isoform X2 [Glycine max] |
| Aradu.BR7YL | 0.07 | Unknown protein |
| Aradu.E0KPU | 0.02 | serine/threonine-protein phosphatase 7 long form homolog [Glycine max]; IPR019557 (Aminotransferase-like, plant mobile domain) |
| Aradu.FTM01 | 0.94 | protein n=2 Tax=Oryza sativa subsp. japonica RepID=C7IXG4_ORYSJ; IPR019557 (Aminotransferase-like, plant mobile domain) |
| Aradu.FX039 | 0.09 | DNAJ heat shock N-terminal domain-containing protein |
| Aradu.H8Q03 | 0.03 | Cytochrome P450 superfamily protein; IPR001128 (Cytochrome P450); GO:0005506 (iron ion binding), GO:0020037 (heme binding), GO:0055114 (oxidation-reduction process) |
| Aradu.I0MJX | 0.09 | expansin B2; IPR007118 (Expansin/Lol pI); GO:0005576 (extracellular region) |
| Aradu.I6CBB | 0.17 | 30S ribosomal protein S2; IPR001865 (Ribosomal protein S2), IPR023591 (Ribosomal protein S2, flavodoxin-like domain); GO:0003735 (structural constituent of ribosome), GO:0005622 (intracellular), GO:0005840 (ribosome), GO:0006412 (translation) |
| Aradu.IJE78 | 0.07 | protein disulfide isomerase-like protein; IPR012336 (Thioredoxin-like fold); GO:0045454 (cell redox homeostasis) |
| Aradu.LW2WT | 0.07 | Unknown protein |
| Aradu.MRP2T | 0.1 | Unknown protein |
| Aradu.MW9R0 | 0.07 | polygalacturonase 4; IPR000743 (Glycoside hydrolase, family 28), IPR011050 (Pectin lyase fold/virulence factor); GO:0004650 (polygalacturonase activity), GO:0005975 (carbohydrate metabolic process) |
| Aradu.NSZ11 | 0.04 | Ulp1 protease family, carboxy-terminal domain protein |
| Aradu.QLG8X | 0.1 | DNA-directed RNA polymerase subunit beta |
| Aradu.WS3G6 | 0.09 | DNAJ heat shock N-terminal domain-containing protein |
| Aradu.X56UA | 0.04 | zinc finger MYM-type protein 1-like [Glycine max]; IPR025398 (Domain of unknown function DUF4371) |
| Aradu.Y02UP | 0.04 | Unknown protein |
| Araip.0E7YN | 0.18 | Unknown protein |
| Araip.14QYU | 0.04 | IQ calmodulin-binding region; Myosin head, motor region; Prefoldin n=1 Tax=Medicago truncatula RepID=A2Q3J3_MEDTR; IPR000048 (IQ motif, EF-hand binding site), IPR001609 (Myosin head, motor domain), IPR027417 (P-loop containing nucleoside triphosphate hydrolase); GO:0003774 (motor activity), GO:0005515 (protein binding), GO:0005524 (ATP binding), GO:0016459 (myosin complex) |
| Araip.22D3C | 0.09 | probable methyltransferase PMT26-like [Glycine max]; IPR004159 (Putative S-adenosyl-L-methionine-dependent methyltransferase); GO:0008168 (methyltransferase activity) |
| Araip.2D1WZ | 0.09 | Unknown protein |
| Araip.4AU3V | 0.96 | cytochrome C biogenesis protein B |
| Araip.98TRK | 0.07 | protein FAR1-RELATED SEQUENCE 5-like isoform X2 [Glycine max]; IPR004330 (FAR1 DNA binding domain) |
| Araip.A065V | 0.05 | hypothetical protein |
| Araip.AAA43 | 0.03 | Unknown protein |
| Araip.B7RLF | 0.05 | protein FAR1-RELATED SEQUENCE 6-like isoform X2 [Glycine max] |
| Araip.B9BR3 | 1.06 | squamous cell carcinoma antigen recognized by T-cells 3-like isoform X3 [Glycine max] |
| Araip.D47VS | 0.17 | Unknown protein |
| Araip.DCW5T | 0.35 | Unknown protein; IPR004332 (Transposase, MuDR, plant) |
| Araip.H0WY3 | 0.1 | Unknown protein |
| Araip.HH4UQ | 0.04 | E3 ubiquitin-protein ligase HERC2-like [Glycine max]; IPR009091 (Regulator of chromosome condensation 1/beta-lactamase-inhibitor protein II) |
| Araip.HQZ0Y | 0.18 | Pentatricopeptide repeat (PPR) superfamily protein; IPR002885 (Pentatricopeptide repeat), IPR017451 (F-box associated interaction domain) |
| Araip.I2R6G | 0.03 | uncharacterized protein LOC102667573 [Glycine max]; IPR004332 (Transposase, MuDR, plant) |
| Araip.J1RB6 | 0.14 | AP2-like ethylene-responsive transcription factor |
| Araip.M29QE | 0.08 | Unknown protein |
| Araip.ME8TD | 0.03 | serine/threonine-protein phosphatase 7 long form homolog [Glycine max]; IPR019557 (Aminotransferase-like, plant mobile domain) |
| Araip.MUB6W | 0.06 | serine/threonine-protein phosphatase 7 long form homolog [Glycine max]; IPR019557 (Aminotransferase-like, plant mobile domain) |
| Araip.N5A2T | 0.08 | uncharacterized protein LOC100776940 isoform X3 [Glycine max] |
| Araip.NQ836 | 0.67 | UPF0481 protein At3g47200-like [Glycine max]; IPR004158 (Protein of unknown function DUF247, plant) |
| Araip.SMK7M | 0.06 | LRR receptor-like serine/threonine-protein kinase FLS2-like [Glycine max] |
| Araip.VH2R0 | 0.09 | Unknown protein |
| Araip.X0LGK | 0.09 | NAD(P)H-quinone oxidoreductase subunit 2 |
| Araip.X1ALQ | 0.02 | Unknown protein |
| Araip.XPP90 | 0.61 | senescence-associated protein |
| Araip.YVD33 | 0.07 | MATE efflux family protein; IPR002528 (Multi antimicrobial extrusion protein); GO:0006855 (drug transmembrane transport), GO:0015238 (drug transmembrane transporter activity), GO:0015297 (antiporter activity), GO:0016020 (membrane), GO:0055085 (transmembrane transport) |
| Araip.ZI3RF | 0.09 | Unknown protein |
| Araip.ZT5E3 | 0.73 | sucrose transporter 4; IPR016196 (Major facilitator superfamily domain, general substrate transporter) |
| Aerial gynophore | |  |
| Aradu.0A7D1 | 0.06 | 30S ribosomal protein S4; IPR001912 (Ribosomal protein S4/S9, N-terminal); GO:0005622 (intracellular), GO:0019843 (rRNA binding) |
| Aradu.3EE99 | 0.02 | fasciclin-like arabinogalactan protein |
| Aradu.4M07K | 0.01 | n=1 Tax=Oryza sativa subsp. japonica RepID=Q7XSJ2_ORYSJ; IPR004332 (Transposase, MuDR, plant) |
| Aradu.64782 | 0.03 | protein FAR1-RELATED SEQUENCE 6-like isoform X2 [Glycine max]; IPR004330 (FAR1 DNA binding domain) |
| Aradu.64XUQ | 0.02 | E3 ubiquitin-protein ligase n=5 Tax=Solanum RepID=M1BDZ1_SOLTU |
| Aradu.7HH6H | 0.01 | Cytochrome P450 superfamily protein; IPR001128 (Cytochrome P450); GO:0005506 (iron ion binding), GO:0020037 (heme binding), GO:0055114 (oxidation-reduction process) |
| Aradu.8F90P | 0.02 | myb family transcription factor APL-like isoform X2 [Glycine max]; IPR025756 (MYB-CC type transcription factor, LHEQLE-containing domain) |
| Aradu.8T9YS | 0.16 | ORF46f n=1 Tax=Pinus koraiensis RepID=Q85WT3_PINKO |
| Aradu.B3VU2 | 0.02 | ribose-5-phosphate isomerase A |
| Aradu.BHW4P | 0.02 | tubulin alpha-2 chain-like [Glycine max] |
| Aradu.CZE0M | 0.02 | Unknown protein |
| Aradu.D48TD | 0.01 | splicing regulatory glutamine/lysine-rich protein 1-like [Glycine max] |
| Aradu.DB189 | 0.03 | DUF674 family protein; IPR007750 (Protein of unknown function DUF674) |
| Aradu.EDA5Q | 0.08 | Protein kinase superfamily protein; IPR011009 (Protein kinase-like domain); GO:0004672 (protein kinase activity), GO:0005524 (ATP binding), GO:0006468 (protein phosphorylation) |
| Aradu.H6NIG | 0.02 | zinc finger MYM-type protein 1-like [Glycine max]; IPR012337 (Ribonuclease H-like domain), IPR025398 (Domain of unknown function DUF4371); GO:0003676 (nucleic acid binding) |
| Aradu.HF645 | 0.06 | germin-like protein 9-3-like [Glycine max]; IPR001929 (Germin); GO:0030145 (manganese ion binding), GO:0045735 (nutrient reservoir activity) |
| Aradu.HGH7Z | 0.03 | Unknown protein |
| Aradu.HSQ50 | 0.01 | Methyltransferase type 11 n=1 Tax=Nostoc sp. PCC 7107 RepID=K9QF47_9NOSO; IPR013216 (Methyltransferase type 11); GO:0008152 (metabolic process), GO:0008168 (methyltransferase activity) |
| Aradu.I8ZUP | 0.02 | uncharacterized protein LOC102666382 [Glycine max] |
| Aradu.J9NH8 | 0.01 | uncharacterized protein At3g06530-like isoform X2 [Glycine max] |
| Aradu.JC7GM | 0.01 | Unknown protein; IPR019557 (Aminotransferase-like, plant mobile domain) |
| Aradu.K5D8C | 0.01 | FUNCTIONS IN: molecular_function unknown; INVOLVED IN: oxidation reduction; EXPRESSED IN: 23 plant structures; EXPRESSED DURING: 15 growth stages ; IPR010376 (Domain of unknown function, DUF971) |
| Aradu.KXK0Y | 0.07 | Unknown protein |
| Aradu.LG4QY | 0.04 | zinc finger MYM-type protein 1-like [Glycine max] |
| Aradu.M2JJJ | 0.1 | hypothetical protein |
| Aradu.NIR3G | 0.01 | 40S ribosomal protein S13 [Glycine max]; IPR000589 (Ribosomal protein S15); GO:0003735 (structural constituent of ribosome), GO:0005622 (intracellular), GO:0005840 (ribosome), GO:0006412 (translation) |
| Aradu.QPP1Y | 0.01 | major intrinsic protein (MIP) family transporter; IPR000425 (Major intrinsic protein), IPR023271 (Aquaporin-like); GO:0005215 (transporter activity), GO:0006810 (transport), GO:0016020 (membrane) |
| Aradu.QRA31 | 0.01 | Polyprotein n=1 Tax=Citrus endogenous pararetrovirus RepID=V9QEM3_9RETR; IPR001878 (Zinc finger, CCHC-type); GO:0003676 (nucleic acid binding), GO:0008270 (zinc ion binding) |
| Aradu.UGS4Q | 0.02 | Unknown protein |
| Aradu.X2VIV | 0.03 | Unknown protein |
| Aradu.X3ZVY | 0.07 | APO RNA-binding protein; IPR023342 (APO domain); GO:0003723 (RNA binding) |
| Araip.1BJ9C | 0.01 | protein FAR1-RELATED SEQUENCE 5-like [Glycine max]; IPR007527 (Zinc finger, SWIM-type); GO:0008270 (zinc ion binding) |
| Araip.24EFM | 0.05 | Unknown protein |
| Araip.47YQM | 0.06 | Unknown protein |
| Araip.4DS8Z | 0.02 | Unknown protein |
| Araip.4W042 | 0.01 | serine/threonine-protein phosphatase 7 long form homolog [Glycine max]; IPR019557 (Aminotransferase-like, plant mobile domain) |
| Araip.60P30 | 0.02 | protein n=1 Tax=Oryza sativa subsp. japonica RepID=C7J0D1_ORYSJ; IPR007527 (Zinc finger, SWIM-type); GO:0008270 (zinc ion binding) |
| Araip.6IG3N | 0.04 | Aluminium induced protein with YGL and LRDR motifs; IPR024286 (Domain of unknown function DUF3700) |
| Araip.6M9JD | 0.05 | photosystem I assembly protein ycf3 |
| Araip.7611F | 0.02 | Unknown protein |
| Araip.7L672 | 0.02 | chalcone synthase [Glycine max]; IPR011141 (Polyketide synthase, type III), IPR016039 (Thiolase-like); GO:0003824 (catalytic activity), GO:0008152 (metabolic process), GO:0009058 (biosynthetic process) |
| Araip.93WG4 | 0.05 | response regulator 24; IPR011006 (CheY-like superfamily); GO:0000156 (phosphorelay response regulator activity), GO:0000160 (phosphorelay signal transduction system) |
| Araip.AQ828 | 0.01 | Unknown protein |
| Araip.B2L07 | 0.02 | Transposon protein n=1 Tax=Arachis hypogaea RepID=G0Y6V7_ARAHY |
| Araip.BJ28Z | 0.01 | protein n=1 Tax=Oryza sativa subsp. japonica RepID=C7J340_ORYSJ; IPR001878 (Zinc finger, CCHC-type), IPR007527 (Zinc finger, SWIM-type); GO:0003676 (nucleic acid binding), GO:0008270 (zinc ion binding) |
| Araip.E2CFA | 0.01 | serine/threonine-protein phosphatase 7 long form homolog [Glycine max]; IPR019557 (Aminotransferase-like, plant mobile domain) |
| Araip.GJR7I | 0.01 | protein kinase family protein; IPR000014 (PAS domain); GO:0004871 (signal transducer activity), GO:0007165 (signal transduction) |
| Araip.H97K5 | 0.01 | Unknown protein |
| Araip.I7XUT | 0.01 | ferric-chelate reductase 1-like [Glycine max]; IPR004877 (Cytochrome b561, eukaryote), IPR005018 (DOMON domain); GO:0016021 (integral component of membrane) |
| Araip.IQB8N | 0.01 | Uroporphyrinogen decarboxylase; IPR000257 (Uroporphyrinogen decarboxylase (URO-D)); GO:0004853 (uroporphyrinogen decarboxylase activity), GO:0006779 (porphyrin-containing compound biosynthetic process) |
| Araip.J914T | 0.01 | protein FAR1-RELATED SEQUENCE 6-like isoform X2 [Glycine max]; IPR004330 (FAR1 DNA binding domain), IPR007527 (Zinc finger, SWIM-type); GO:0008270 (zinc ion binding) |
| Araip.K216L | 0.01 | Unknown protein |
| Araip.KD5PB | 0.01 | nodulin MtN21/EamA-like transporter family protein; IPR012340 (Nucleic acid-binding, OB-fold), IPR025977 (Nuclear condensin complex subunit 3, C-terminal domain) |
| Araip.KTA3I | 0.01 | Unknown protein; IPR004252 (Probable transposase, Ptta/En/Spm, plant) |
| Araip.KWN7Q | 0.01 | Protein kinase superfamily protein; IPR011009 (Protein kinase-like domain); GO:0004672 (protein kinase activity), GO:0005524 (ATP binding), GO:0006468 (protein phosphorylation) |
| Araip.L0Y1W | 0.01 | Unknown protein |
| Araip.LK4NN | 0.01 | Unknown protein |
| Araip.M1KQL | 0.04 | serine/threonine-protein phosphatase 7 long form homolog [Glycine max]; IPR019557 (Aminotransferase-like, plant mobile domain) |
| Araip.M71XL | 0.02 | Unknown protein |
| Araip.PI3IW | 0.01 | Unknown protein |
| Araip.QL8AX | 0.03 | serine acetyltransferase 2; 2; IPR011004 (Trimeric LpxA-like); GO:0005737 (cytoplasm), GO:0006535 (cysteine biosynthetic process from serine), GO:0009001 (serine O-acetyltransferase activity) |
| Araip.RLS3A | 0.01 | RNA recognition motif (RRM)-containing protein; IPR012677 (Nucleotide-binding, alpha-beta plait); GO:0000166 (nucleotide binding), GO:0003676 (nucleic acid binding) |
| Araip.SI3CB | 0.01 | Unknown protein |
| Araip.T3NYK | 0.08 | F-box protein interaction domain protein |
| Araip.T78CP | 0.02 | Unknown protein |
| Araip.T7E18 | 0.01 | uncharacterized protein LOC100820019 isoform X4 [Glycine max] |
| Araip.TYX8Q | 0.04 | photosystem I iron-sulfur center; IPR017491 (Photosystem I protein PsaC); GO:0009055 (electron carrier activity), GO:0009522 (photosystem I), GO:0009773 (photosynthetic electron transport in photosystem I), GO:0015979 (photosynthesis), GO:0042651 (thylakoid membrane), GO:0051536 (iron-sulfur cluster binding) |
| Araip.V0EL6 | 0.02 | outward rectifying potassium channel protein; IPR013099 (Two pore domain potassium channel domain) |
| Araip.V4WFE | 0.06 | alpha-galactosidase; IPR013785 (Aldolase-type TIM barrel); GO:0003824 (catalytic activity) |
| Araip.VZZ1E | 0.02 | Unknown protein |
| Araip.X5MX1 | 0.03 | DUF241 domain protein; IPR004320 (Protein of unknown function DUF241, plant) |
| Araip.X7Y72 | 0.01 | zinc finger MYM-type protein 1-like [Glycine max]; IPR008906 (HAT dimerisation domain, C-terminal), IPR012337 (Ribonuclease H-like domain), IPR025398 (Domain of unknown function DUF4371); GO:0003676 (nucleic acid binding), GO:0046983 (protein dimerization activity) |
| Araip.YK4W9 | 0.03 | U3 small nucleolar ribonucleoprotein protein MPP10-like [Glycine max]; IPR007151 (Mpp10 protein) |
| Araip.YRP9S | 0.01 | protein FAR1-RELATED SEQUENCE 6-like isoform X2 [Glycine max]; IPR007527 (Zinc finger, SWIM-type); GO:0008270 (zinc ion binding) |
| Araip.YW80G | 0.02 | Unknown protein |
| Subterranean gynophore | |  |
| Aradu.14LAG | 0.14 | ribulose bisphosphate carboxylase large chain; IPR000685 (Ribulose bisphosphate carboxylase, large subunit, C-terminal); GO:0000287 (magnesium ion binding) |
| Aradu.25KN6 | 0.03 | serine/threonine-protein phosphatase 7 long form homolog [Glycine max]; IPR019557 (Aminotransferase-like, plant mobile domain) |
| Aradu.2K7B5 | 0.01 | serine/threonine-protein phosphatase 7 long form homolog [Glycine max]; IPR019557 (Aminotransferase-like, plant mobile domain) |
| Aradu.2L1IQ | 0.08 | Unknown protein |
| Aradu.535BG | 0.11 | probable lysine-specific demethylase JMJ14-like isoform X2 [Glycine max]; IPR003347 (JmjC domain); GO:0005515 (protein binding) |
| Aradu.96E3Y | 0.03 | uncharacterized protein LOC102665521 [Glycine max]; IPR007021 (Domain of unknown function DUF659), IPR012337 (Ribonuclease H-like domain); GO:0003676 (nucleic acid binding) |
| Aradu.AE8L1 | 0.11 | Unknown protein |
| Aradu.B0J2R | 0.08 | uncharacterized protein LOC102663263 [Glycine max] |
| Aradu.B4WS8 | 0.03 | protein FAR1-RELATED SEQUENCE 5-like [Glycine max]; IPR001878 (Zinc finger, CCHC-type), IPR007527 (Zinc finger, SWIM-type); GO:0003676 (nucleic acid binding), GO:0008270 (zinc ion binding) |
| Aradu.CJ9RT | 0.07 | Unknown protein |
| Aradu.CLS2B | 0.14 | Unknown protein |
| Aradu.DB48N | 0.07 | ORF64d n=1 Tax=Pinus koraiensis RepID=A4QMB0_PINKO |
| Aradu.EC2U8 | 0.03 | NADH-ubiquinone oxidoreductase chain n=3 Tax=Pentapetalae RepID=G7I8V1_MEDTR; IPR001694 (NADH:ubiquinone oxidoreductase, subunit 1/F420H2 oxidoreductase subunit H); GO:0016020 (membrane), GO:0055114 (oxidation-reduction process) |
| Aradu.F6EEQ | 0.03 | E3 ubiquitin-protein ligase n=5 Tax=Solanum RepID=M1BDZ1_SOLTU |
| Aradu.J2VLX | 0.08 | cell division control protein |
| Aradu.K40A2 | 0.03 | Unknown protein; IPR019557 (Aminotransferase-like, plant mobile domain) |
| Aradu.R3627 | 0.15 | putative phagocytic receptor 1b-like [Glycine max] |
| Aradu.RZ29H | 0.02 | Zinc knuckle family protein n=1 Tax=Solanum demissum RepID=Q6L424_SOLDE; IPR018247 (EF-Hand 1, calcium-binding site) |
| Aradu.SQ8NZ | 0.05 | Unknown protein |
| Aradu.XCK55 | 0.29 | HXXXD-type acyl-transferase family protein; IPR003480 (Transferase), IPR023213 (Chloramphenicol acetyltransferase-like domain) |
| Aradu.YC4LW | 0.02 | protein FAR1-RELATED SEQUENCE 6-like isoform X2 [Glycine max] |
| Araip.1168D | 0.08 | ankyrin repeat-containing protein At5g02620-like isoform X3 [Glycine max] |
| Araip.30IMT | 0.17 | Unknown protein |
| Araip.32407 | 0.05 | Unknown protein |
| Araip.6ZC3D | 0.06 | Cell wall protein EXP2 n=1 Tax=Mirabilis jalapa RepID=Q84L40_MIRJA; IPR007118 (Expansin/Lol pI); GO:0005576 (extracellular region), GO:0009664 (plant-type cell wall organization) |
| Araip.8TA01 | 0.05 | FAR1 DNA-binding domain protein; IPR004330 (FAR1 DNA binding domain) |
| Araip.DVN03 | 0.06 | ethanolamine-phosphate cytidylyltransferase-like isoform 1 [Glycine max] |
| Araip.H4E6K | 0.13 | arginine/serine-rich coiled coil protein |
| Araip.I3S7Y | 0.02 | glutamate synthase 1; IPR002634 (BolA protein), IPR013785 (Aldolase-type TIM barrel); GO:0003824 (catalytic activity), GO:0006537 (glutamate biosynthetic process), GO:0015930 (glutamate synthase activity), GO:0055114 (oxidation-reduction process) |
| Araip.I482V | 0.02 | Unknown protein |
| Araip.INZ27 | 0.04 | Unknown protein |
| Araip.J4XAL | 0.01 | putative lactoylglutathione lyase-like isoform X2 [Glycine max] |
| Araip.N4BCK | 0.08 | uncharacterized protein LOC100793937 [Glycine max] |
| Araip.PW566 | 1.34 | Cysteine proteinases superfamily protein; IPR003323 (Ovarian tumour, otubain) |
| Araip.QD4NN | 0.02 | protein FAR1-RELATED SEQUENCE 6-like isoform X2 [Glycine max] |
| Araip.QR4HQ | 0.14 | 30S ribosomal protein S19, chloroplastic n=70 Tax=Pentapetalae RepID=RR19_POPTR; IPR001280 (Photosystem I PsaA/PsaB), IPR002222 (Ribosomal protein S19/S15), IPR023575 (Ribosomal protein S19, superfamily); GO:0003735 (structural constituent of ribosome), GO:0005840 (ribosome), GO:0006412 (translation), GO:0009522 (photosystem I), GO:0009579 (thylakoid), GO:0015979 (photosynthesis), GO:0016021 (integral component of membrane) |
| Araip.R5WSP | 0.04 | TdcA1-ORF2 protein n=1 Tax=Medicago truncatula RepID=G7JDI9_MEDTR |
| Araip.RD9MC | 0.09 | 40S ribosomal protein S3-3 [Glycine max]; IPR001351 (Ribosomal protein S3, C-terminal); GO:0003735 (structural constituent of ribosome), GO:0005840 (ribosome), GO:0006412 (translation) |
| Araip.RW1H5 | 0.03 | Unknown protein |
| Araip.RX7FU | 0.02 | protein FAR1-RELATED SEQUENCE 3-like isoform X1 [Glycine max]; IPR001878 (Zinc finger, CCHC-type), IPR006564 (Zinc finger, PMZ-type); GO:0003676 (nucleic acid binding), GO:0008270 (zinc ion binding) |
| Araip.SWG9K | 0.13 | Unknown protein |
| Araip.TSC1G | 0.05 | receptor kinase 3; IPR011009 (Protein kinase-like domain); GO:0004672 (protein kinase activity), GO:0005524 (ATP binding), GO:0006468 (protein phosphorylation) |
| Araip.U5ZTR | 0.14 | Transposon protein n=1 Tax=Arachis hypogaea RepID=G0Y6V7_ARAHY |
| Araip.UW8U0 | 0.01 | protein FAR1-RELATED SEQUENCE 6-like isoform X2 [Glycine max]; IPR004330 (FAR1 DNA binding domain) |
| Araip.X7EMA | 0.02 | uncharacterized protein LOC100776940 isoform X4 [Glycine max]; IPR004332 (Transposase, MuDR, plant) |
| Araip.YX92K | 0.04 | folylpolyglutamate synthase-like isoform X2 [Glycine max]; IPR001645 (Folylpolyglutamate synthetase); GO:0004326 (tetrahydrofolylpolyglutamate synthase activity), GO:0005524 (ATP binding), GO:0009058 (biosynthetic process), GO:0009396 (folic acid-containing compound biosynthetic process) |
| Pattee 1 pod | |  |
| Aradu.1RS71 | 0.04 | zinc finger MYM-type protein 1-like [Glycine max]; IPR025398 (Domain of unknown function DUF4371) |
| Aradu.4J2R7 | 0.1 | LRR receptor-like kinase family protein |
| Aradu.5SX98 | 0.05 | AP2-like ethylene-responsive transcription factor ANT-like isoform X5 [Glycine max]; IPR016177 (DNA-binding domain); GO:0003677 (DNA binding), GO:0003700 (sequence-specific DNA binding transcription factor activity) |
| Aradu.87DC0 | 0.05 | receptor-like protein kinase 2; IPR011009 (Protein kinase-like domain); GO:0004672 (protein kinase activity), GO:0005524 (ATP binding), GO:0006468 (protein phosphorylation) |
| Aradu.9C166 | 0.04 | FAR1 DNA-binding domain protein; IPR004330 (FAR1 DNA binding domain) |
| Aradu.C4WT9 | 0.06 | cyclic nucleotide gated channel 19; IPR014710 (RmlC-like jelly roll fold) |
| Aradu.EN49C | 0.01 | magnesium transporter NIPA2-like [Glycine max]; IPR008521 (Magnesium transporter NIPA); GO:0015095 (magnesium ion transmembrane transporter activity), GO:0015693 (magnesium ion transport), GO:0016020 (membrane) |
| Aradu.G0Z82 | 0.03 | WAT1-related protein At1g68170-like [Glycine max] |
| Aradu.K4GSH | 0.12 | tubulin alpha-6 chain, putative |
| Aradu.M2CWI | 0.03 | Unknown protein |
| Aradu.MFE9K | 0.01 | Unknown protein |
| Aradu.RS2S8 | 0.04 | serine/threonine-protein phosphatase 7 long form homolog [Glycine max]; IPR019557 (Aminotransferase-like, plant mobile domain) |
| Aradu.SYG8A | 0.01 | protein FAR1-RELATED SEQUENCE 6-like isoform X2 [Glycine max]; IPR004330 (FAR1 DNA binding domain), IPR007527 (Zinc finger, SWIM-type); GO:0008270 (zinc ion binding) |
| Aradu.T554D | 0.03 | serine/threonine-protein phosphatase 7 long form homolog [Glycine max]; IPR019557 (Aminotransferase-like, plant mobile domain) |
| Aradu.TYS9T | 0.03 | serine/threonine-protein phosphatase 7 long form homolog [Glycine max]; IPR019557 (Aminotransferase-like, plant mobile domain) |
| Aradu.UD9XR | 0.03 | FAR1 DNA-binding domain protein; IPR004330 (FAR1 DNA binding domain) |
| Aradu.VC95R | 0.04 | WRKY transcription factor 44-like [Glycine max] |
| Aradu.W7JB7 | 0.03 | UDP-D-glucose/UDP-D-galactose 4-epimerase 5; IPR001509 (NAD-dependent epimerase/dehydratase), IPR016040 (NAD(P)-binding domain); GO:0003824 (catalytic activity), GO:0044237 (cellular metabolic process), GO:0050662 (coenzyme binding) |
| Aradu.W9W4G | 0.01 | zinc finger MYM-type protein 1-like [Glycine max]; IPR008906 (HAT dimerisation domain, C-terminal), IPR012337 (Ribonuclease H-like domain), IPR025398 (Domain of unknown function DUF4371); GO:0003676 (nucleic acid binding), GO:0046983 (protein dimerization activity) |
| Aradu.Y6V5V | 0.01 | Unknown protein; IPR015300 (DNA-binding pseudobarrel domain) |
| Aradu.YT8VJ | 0.03 | nodulin MtN21 /EamA-like transporter family protein |
| Araip.06A50 | 0.01 | uncharacterized protein LOC100778822 isoform X6 [Glycine max]; IPR004252 (Probable transposase, Ptta/En/Spm, plant) |
| Araip.4H1ZU | 0.03 | Unknown protein |
| Araip.CM6EV | 0.14 | RAB GTPase-like protein A5B |
| Araip.DX3U9 | 0.03 | protein n=1 Tax=Oryza sativa subsp. japonica RepID=C7J9W8_ORYSJ; IPR004332 (Transposase, MuDR, plant) |
| Araip.E2EFZ | 0.03 | U5 small nuclear ribonucleoprotein helicase, putative; IPR027417 (P-loop containing nucleoside triphosphate hydrolase) |
| Araip.FY40B | 0.07 | Unknown protein |
| Araip.HP3C4 | 0.06 | Unknown protein |
| Araip.K116B | 0.01 | serine/threonine-protein phosphatase 7 long form homolog [Glycine max]; IPR019557 (Aminotransferase-like, plant mobile domain) |
| Araip.P5RKY | 0.02 | plasma membrane H+-ATPase; IPR008250 (P-type ATPase, A domain), IPR023299 (P-type ATPase, cytoplasmic domain N); GO:0000166 (nucleotide binding), GO:0046872 (metal ion binding) |
| Araip.S2QR5 | 0.04 | PIF1-like helicase |
| Araip.SZI4S | 0.05 | Pentatricopeptide repeat (PPR) superfamily protein |
| Araip.VX1RZ | 0.04 | Unknown protein |
| Araip.W0MYP | 0.03 | zinc finger MYM-type protein 1-like [Glycine max]; IPR008906 (HAT dimerisation domain, C-terminal), IPR012337 (Ribonuclease H-like domain), IPR025398 (Domain of unknown function DUF4371); GO:0003676 (nucleic acid binding), GO:0046983 (protein dimerization activity) |
| Araip.WF1YB | 0.04 | Unknown protein |
| Araip.XXM5U | 0.03 | Werner Syndrome-like exonuclease-like [Glycine max]; IPR012337 (Ribonuclease H-like domain); GO:0003676 (nucleic acid binding), GO:0006139 (nucleobase-containing compound metabolic process), GO:0008408 (3'-5' exonuclease activity) |
| Araip.Y2XWI | 0.04 | tRNA/rRNA methyltransferase (SpoU) family protein |
| Araip.Z21UF | 0.03 | Werner syndrome-like exonuclease; IPR012337 (Ribonuclease H-like domain); GO:0003676 (nucleic acid binding), GO:0006139 (nucleobase-containing compound metabolic process), GO:0008408 (3'-5' exonuclease activity) |
| Araip.ZFS8S | 0.03 | ribosomal protein L18; IPR018108 (Mitochondrial substrate/solute carrier), IPR023395 (Mitochondrial carrier domain) |
| Pattee 1 stalk | |  |
| Aradu.3571R | 0.03 | beta-carotene isomerase D27, chloroplastic-like isoform X1 [Glycine max]; IPR025114 (Domain of unknown function DUF4033) |
| Aradu.3V5SK | 0.03 | Ribosomal protein S11 family protein; IPR000630 (Ribosomal protein S8), IPR001971 (Ribosomal protein S11); GO:0003735 (structural constituent of ribosome), GO:0005840 (ribosome), GO:0006412 (translation) |
| Aradu.4EV79 | 0.11 | Orf214 n=2 Tax=Glycine max RepID=Q01925_SOYBN |
| Aradu.54C9X | 0.01 | protein n=1 Tax=Oryza sativa subsp. japonica RepID=C7IXT5_ORYSJ |
| Aradu.57H9V | 0.08 | Unknown protein |
| Aradu.66N80 | 0.02 | serine/threonine-protein phosphatase 7 long form homolog [Glycine max]; IPR019557 (Aminotransferase-like, plant mobile domain) |
| Aradu.756NB | 0.04 | zinc finger MYM-type protein 1-like [Glycine max]; IPR025398 (Domain of unknown function DUF4371) |
| Aradu.75T65 | 0.03 | NADH-ubiquinone oxidoreductase chain n=1 Tax=Medicago truncatula RepID=G7I8X7_MEDTR |
| Aradu.7FN0C | 0.01 | serine/threonine-protein phosphatase 7 long form homolog [Glycine max]; IPR019557 (Aminotransferase-like, plant mobile domain) |
| Aradu.AE1R4 | 0.04 | hypothetical protein |
| Aradu.AE2VJ | 0.01 | Acetyl-coenzyme A carboxylase carboxyl transferase subunit beta, chloroplastic n=35 Tax=Mesangiospermae RepID=D3WBE7_9AQUA; IPR000438 (Acetyl-CoA carboxylase carboxyl transferase, beta subunit); GO:0003989 (acetyl-CoA carboxylase activity), GO:0006633 (fatty acid biosynthetic process), GO:0009317 (acetyl-CoA carboxylase complex), GO:0016874 (ligase activity) |
| Aradu.AX7EV | 0.02 | ATP synthase F1, alpha subunit; IPR000793 (ATPase, F1/V1/A1 complex, alpha/beta subunit, C-terminal), IPR020003 (ATPase, alpha/beta subunit, nucleotide-binding domain, active site); GO:0005524 (ATP binding), GO:0015991 (ATP hydrolysis coupled proton transport) |
| Aradu.B3CTG | 0.01 | serine/threonine-protein phosphatase 7 long form homolog [Glycine max]; IPR019557 (Aminotransferase-like, plant mobile domain) |
| Aradu.B7XEZ | 0.08 | Ycf2 [Glycine max]; IPR008543 (Uncharacterised protein family Ycf2); GO:0005524 (ATP binding), GO:0009507 (chloroplast) |
| Aradu.B876B | 0.02 | ATP synthase F1, alpha subunit; IPR000793 (ATPase, F1/V1/A1 complex, alpha/beta subunit, C-terminal), IPR020003 (ATPase, alpha/beta subunit, nucleotide-binding domain, active site); GO:0005524 (ATP binding), GO:0015991 (ATP hydrolysis coupled proton transport) |
| Aradu.BST2V | 0.02 | NADH-ubiquinone oxidoreductase chain n=1 Tax=Medicago truncatula RepID=G7I8X7_MEDTR |
| Aradu.C4XC4 | 0.02 | NADH dehydrogenase subunit 2 [Glycine max]; IPR001750 (NADH:ubiquinone/plastoquinone oxidoreductase); GO:0008137 (NADH dehydrogenase (ubiquinone) activity), GO:0055114 (oxidation-reduction process) |
| Aradu.CS3IZ | 0.03 | delta-aminolevulinic acid dehydratase; IPR001731 (Porphobilinogen synthase), IPR013785 (Aldolase-type TIM barrel); GO:0003824 (catalytic activity), GO:0004655 (porphobilinogen synthase activity), GO:0033014 (tetrapyrrole biosynthetic process), GO:0046872 (metal ion binding) |
| Aradu.CT66K | 0.11 | Orf214 n=2 Tax=Glycine max RepID=Q01925_SOYBN |
| Aradu.CX9XP | 0.01 | Acetyl-coenzyme A carboxylase carboxyl transferase subunit beta, chloroplastic n=35 Tax=Mesangiospermae RepID=D3WBE7_9AQUA; IPR000438 (Acetyl-CoA carboxylase carboxyl transferase, beta subunit); GO:0003989 (acetyl-CoA carboxylase activity), GO:0006633 (fatty acid biosynthetic process), GO:0009317 (acetyl-CoA carboxylase complex), GO:0016874 (ligase activity) |
| Aradu.E47VL | 0.01 | UDP-Glycosyltransferase superfamily protein; IPR002213 (UDP-glucuronosyl/UDP-glucosyltransferase); GO:0008152 (metabolic process) |
| Aradu.E555B | 0.05 | E3 SUMO-protein ligase SIZ1-like isoform X1 [Glycine max]; IPR013083 (Zinc finger, RING/FYVE/PHD-type) |
| Aradu.EEA88 | 0.01 | NADH-ubiquinone oxidoreductase chain n=309 Tax=Magnoliophyta RepID=G7IB69_MEDTR; IPR003945 (NADH-plastoquinone oxidoreductase, chain 5); GO:0008137 (NADH dehydrogenase (ubiquinone) activity), GO:0042773 (ATP synthesis coupled electron transport), GO:0055114 (oxidation-reduction process) |
| Aradu.F2D3W | 0.03 | E3 ubiquitin-protein ligase n=5 Tax=Solanum RepID=M1BDZ1_SOLTU |
| Aradu.F6U6T | 0.07 | Zinc finger MYM-type protein n=1 Tax=Medicago truncatula RepID=G7IA75_MEDTR; IPR025398 (Domain of unknown function DUF4371) |
| Aradu.F83KP | 0.01 | Cytochrome c oxidase subunit 2 n=6 Tax=Mesangiospermae RepID=B9T9N2_RICCO; IPR008972 (Cupredoxin), IPR014222 (Cytochrome c oxidase, subunit II); GO:0004129 (cytochrome-c oxidase activity), GO:0005507 (copper ion binding), GO:0016020 (membrane), GO:0016021 (integral component of membrane), GO:0016491 (oxidoreductase activity), GO:0022900 (electron transport chain) |
| Aradu.HY686 | 0.04 | Unknown protein; IPR010666 (Zinc finger, GRF-type); GO:0008270 (zinc ion binding) |
| Aradu.I2IT9 | 0.07 | uncharacterized protein LOC102669969 isoform X2 [Glycine max]; IPR026103 (Harbinger transposase-derived nuclease, animal) |
| Aradu.IDL6T | 0.06 | diphthamide biosynthesis protein 1-like [Glycine max]; IPR002728 (Diphthamide synthesis, DPH1/DPH2); GO:0005737 (cytoplasm), GO:0017183 (peptidyl-diphthamide biosynthetic process from peptidyl-histidine) |
| Aradu.IZ5QI | 0.01 | Alpha-L RNA-binding motif/Ribosomal protein S4 family protein; IPR002942 (RNA-binding S4 domain); GO:0003723 (RNA binding) |
| Aradu.KE9LX | 0.1 | serine/threonine-protein phosphatase 7 long form homolog [Glycine max]; IPR019557 (Aminotransferase-like, plant mobile domain) |
| Aradu.L7IQ5 | 0.01 | hypothetical protein |
| Aradu.LHP11 | 0.04 | Unknown protein |
| Aradu.M32JW | 0.03 | Unknown protein |
| Aradu.M7AQA | 0.01 | FAR-RED impaired response-like protein, putative; IPR001878 (Zinc finger, CCHC-type); GO:0003676 (nucleic acid binding), GO:0008270 (zinc ion binding) |
| Aradu.NKK1T | 0.09 | Unknown protein; IPR007527 (Zinc finger, SWIM-type); GO:0008270 (zinc ion binding) |
| Aradu.RB0D2 | 0.04 | pentatricopeptide repeat-containing protein At5g10690-like [Glycine max] |
| Aradu.RCA9S | 0.04 | Unknown protein |
| Aradu.S3PRJ | 0.02 | Alpha-L RNA-binding motif/Ribosomal protein S4 family protein; IPR002942 (RNA-binding S4 domain); GO:0003723 (RNA binding) |
| Aradu.SV9VB | 0.01 | NADH-ubiquinone oxidoreductase chain n=309 Tax=Magnoliophyta RepID=G7IB69_MEDTR; IPR003945 (NADH-plastoquinone oxidoreductase, chain 5); GO:0008137 (NADH dehydrogenase (ubiquinone) activity), GO:0042773 (ATP synthesis coupled electron transport), GO:0055114 (oxidation-reduction process) |
| Aradu.UY7F2 | 0.02 | zinc finger MYM-type protein 1-like [Glycine max]; IPR008906 (HAT dimerisation domain, C-terminal), IPR012337 (Ribonuclease H-like domain); GO:0003676 (nucleic acid binding), GO:0046983 (protein dimerization activity) |
| Aradu.V9MBP | 0.04 | photosystem II CP47 chlorophyll A apoprotein; IPR000932 (Photosystem antenna protein-like); GO:0009521 (photosystem), GO:0009767 (photosynthetic electron transport chain), GO:0016020 (membrane), GO:0016168 (chlorophyll binding) |
| Aradu.VKF10 | 0.1 | Unknown protein |
| Aradu.VS85S | 0.01 | uncharacterized protein LOC100797259 isoform X4 [Glycine max]; IPR004332 (Transposase, MuDR, plant), IPR006564 (Zinc finger, PMZ-type); GO:0008270 (zinc ion binding) |
| Aradu.WYP5T | 0.01 | Cytochrome c oxidase subunit 2 n=6 Tax=Mesangiospermae RepID=B9T9N2_RICCO; IPR008972 (Cupredoxin), IPR014222 (Cytochrome c oxidase, subunit II); GO:0004129 (cytochrome-c oxidase activity), GO:0005507 (copper ion binding), GO:0016020 (membrane), GO:0016021 (integral component of membrane), GO:0016491 (oxidoreductase activity), GO:0022900 (electron transport chain) |
| Aradu.XEJ5N | 0.04 | Photosystem II D2 protein n=17 Tax=Eukaryota RepID=G7JG11_MEDTR; IPR000484 (Photosynthetic reaction centre, L/M); GO:0009772 (photosynthetic electron transport in photosystem II) |
| Aradu.XNF8L | 0.02 | Unknown protein |
| Aradu.Z19X2 | 0.07 | NADH dehydrogenase subunit F n=10 Tax=Euphorbia RepID=H6TTD5_9ROSI; IPR002128 (NADH:ubiquinone/plastoquinone oxidoreductase, chloroplast chain 5, C-terminal); GO:0008137 (NADH dehydrogenase (ubiquinone) activity), GO:0042773 (ATP synthesis coupled electron transport), GO:0055114 (oxidation-reduction process) |
| Aradu.Z9HPE | 0.04 | Unknown protein |
| Araip.0299L | 0.04 | serine/threonine-protein phosphatase 7 long form homolog [Glycine max]; IPR019557 (Aminotransferase-like, plant mobile domain) |
| Araip.4Y2LM | 0.03 | Unknown protein |
| Araip.5X1Y4 | 0.03 | replication factor-A carboxy-terminal domain protein; IPR012340 (Nucleic acid-binding, OB-fold) |
| Araip.5YX35 | 0.1 | Unknown protein |
| Araip.66C7B | 0.07 | cytochrome P450 94A1-like [Glycine max] |
| Araip.68UDD | 0.02 | Pentatricopeptide repeat (PPR) superfamily protein; IPR002885 (Pentatricopeptide repeat), IPR011990 (Tetratricopeptide-like helical); GO:0005515 (protein binding) |
| Araip.71HJP | 0.02 | uncharacterized protein LOC100820019 isoform X4 [Glycine max]; IPR025724 (GAG-pre-integrase domain) |
| Araip.77JHK | 0.05 | respiratory burst oxidase homologue D; IPR013623 (NADPH oxidase Respiratory burst); GO:0004601 (peroxidase activity), GO:0055114 (oxidation-reduction process) |
| Araip.B8YGG | 0.07 | S-adenosyl-L-methionine-dependent methyltransferases superfamily protein |
| Araip.BSC2V | 0.06 | putative disease resistance RPP13-like protein 1-like [Glycine max] |
| Araip.C8XD3 | 0.04 | probable methyltransferase PMT11-like [Glycine max]; IPR004159 (Putative S-adenosyl-L-methionine-dependent methyltransferase); GO:0008168 (methyltransferase activity) |
| Araip.CA1LV | 0.02 | uncharacterized protein LOC102666662 [Glycine max]; IPR007527 (Zinc finger, SWIM-type); GO:0008270 (zinc ion binding) |
| Araip.CH767 | 0.06 | Unknown protein |
| Araip.CTR7R | 0.01 | serine/threonine-protein phosphatase 7 long form homolog [Glycine max]; IPR019557 (Aminotransferase-like, plant mobile domain) |
| Araip.DVP0M | 0.02 | NAD(P)H-quinone oxidoreductase subunit H; IPR001135 (NADH-quinone oxidoreductase, subunit D); GO:0048038 (quinone binding), GO:0051287 (NAD binding), GO:0055114 (oxidation-reduction process) |
| Araip.F7B6F | 0.05 | Unknown protein |
| Araip.FZ889 | 0.68 | Unknown protein |
| Araip.G7JHT | 0.04 | Helicase, putative n=1 Tax=Oryza sativa subsp. japonica RepID=Q2R017_ORYSJ |
| Araip.H41XT | 0.02 | probable dolichyl pyrophosphate Man9GlcNAc2 alpha-1,3-glucosyltransferase-like [Glycine max]; IPR004856 (Glycosyl transferase, ALG6/ALG8); GO:0005789 (endoplasmic reticulum membrane) |
| Araip.HA1XL | 0.02 | serine/threonine-protein phosphatase 7 long form homolog [Glycine max]; IPR019557 (Aminotransferase-like, plant mobile domain) |
| Araip.I3L3F | 0.04 | Unknown protein |
| Araip.I8RB5 | 0.07 | Unknown protein; IPR008896 (Uncharacterised protein family Ycf1) |
| Araip.K7S8X | 0.06 | Unknown protein |
| Araip.KCC1D | 0.07 | probable fatty acyl-CoA reductase 5-like [Glycine max]; IPR016040 (NAD(P)-binding domain), IPR026055 (Fatty acyl-CoA reductase); GO:0080019 (fatty-acyl-CoA reductase (alcohol-forming) activity) |
| Araip.KXN07 | 0.07 | hypothetical protein |
| Araip.L0SID | 0.08 | organic cation/carnitine transporter 4-like [Glycine max] |
| Araip.NS35C | 0.04 | Unknown protein; IPR019557 (Aminotransferase-like, plant mobile domain) |
| Araip.PJ8TX | 0.04 | uncharacterized protein LOC102668833 isoform X3 [Glycine max] |
| Araip.PNR1L | 0.03 | receptor-like kinase |
| Araip.Q99P5 | 0.04 | Unknown protein |
| Araip.RMA8G | 0.09 | hypothetical protein |
| Araip.RT6IA | 0.05 | Ribosomal protein L2 family; IPR002171 (Ribosomal protein L2), IPR002222 (Ribosomal protein S19/S15), IPR023575 (Ribosomal protein S19, superfamily); GO:0003735 (structural constituent of ribosome), GO:0005622 (intracellular), GO:0005840 (ribosome), GO:0006412 (translation) |
| Araip.S9W0Q | 0.04 | Unknown protein |
| Araip.SW5JU | 0.04 | replication factor-A carboxy-terminal domain protein; IPR012340 (Nucleic acid-binding, OB-fold) |
| Araip.U4EKP | 0.04 | protein FAR1-RELATED SEQUENCE 9-like isoform X5 [Glycine max]; IPR001878 (Zinc finger, CCHC-type); GO:0003676 (nucleic acid binding), GO:0008270 (zinc ion binding) |
| Araip.UEB18 | 0.04 | Unknown protein |
| Araip.UJ1YW | 0.01 | serine/threonine-protein phosphatase 7 long form homolog [Glycine max]; IPR019557 (Aminotransferase-like, plant mobile domain) |
| Araip.W1EUC | 0.01 | uncharacterized protein LOC100800865 isoform X3 [Glycine max]; IPR004332 (Transposase, MuDR, plant) |
| Araip.WF4P7 | 0.03 | GPI transamidase component PIG-S-related; IPR019540 (Phosphatidylinositol-glycan biosynthesis class S protein); GO:0016255 (attachment of GPI anchor to protein), GO:0042765 (GPI-anchor transamidase complex) |
| Araip.X8WV7 | 0.07 | uncharacterized protein LOC100787145 isoform X9 [Glycine max] |
| Araip.XDM00 | 0.04 | Unknown protein |
| Araip.XN3DQ | 0.03 | lactoylglutathione lyase; IPR004360 (Glyoxalase/fosfomycin resistance/dioxygenase domain), IPR004361 (Glyoxalase I); GO:0004462 (lactoylglutathione lyase activity), GO:0046872 (metal ion binding) |
| Araip.YD644 | 0.03 | protein FAR1-RELATED SEQUENCE 6-like isoform X2 [Glycine max]; IPR004330 (FAR1 DNA binding domain) |
| Araip.YG1LK | 0.04 | F-box family protein; IPR001810 (F-box domain), IPR017451 (F-box associated interaction domain); GO:0005515 (protein binding) |
| Araip.YIL95 | 0.07 | vacuolar iron transporter homolog 1-like [Glycine max] |
| Araip.YU8KT | 0.03 | poly(rC)-binding protein 1-like [Glycine max]; IPR004088 (K Homology domain, type 1); GO:0003723 (RNA binding) |
| Araip.ZF8EV | 0.02 | protein FAR1-RELATED SEQUENCE 6-like isoform X2 [Glycine max]; IPR007527 (Zinc finger, SWIM-type); GO:0008270 (zinc ion binding) |
| Araip.ZWD56 | 0.07 | NADH-ubiquinone oxidoreductase chain 4 n=2 Tax=Spermatophyta RepID=N0DNX9_PHAAN; IPR001750 (NADH:ubiquinone/plastoquinone oxidoreductase); GO:0008137 (NADH dehydrogenase (ubiquinone) activity), GO:0055114 (oxidation-reduction process) |
| Pattee 3 pod | |  |
| Aradu.0JS7R | 0.01 | ALG-2 interacting protein X-like [Glycine max]; IPR004328 (BRO1 domain) |
| Aradu.12ZQ2 | 0.02 | PttB n=1 Tax=Medicago truncatula RepID=G7K5I5_MEDTR |
| Aradu.151MH | 0.01 | disease resistance protein (TIR-NBS-LRR class), putative; IPR000157 (Toll/interleukin-1 receptor homology (TIR) domain), IPR000767 (Disease resistance protein), IPR027417 (P-loop containing nucleoside triphosphate hydrolase); GO:0005515 (protein binding), GO:0006952 (defense response), GO:0007165 (signal transduction), GO:0043531 (ADP binding) |
| Aradu.50KMN | 0.04 | Unknown protein |
| Aradu.5WY8M | 0.01 | protein FAR1-RELATED SEQUENCE 6-like isoform X2 [Glycine max]; IPR004330 (FAR1 DNA binding domain) |
| Aradu.66YC3 | 0.02 | zinc ion binding; IPR010666 (Zinc finger, GRF-type); GO:0008270 (zinc ion binding) |
| Aradu.6X9U5 | 0.06 | uncharacterized protein LOC100804482 isoform X3 [Glycine max] |
| Aradu.7AR47 | 0.04 | Unknown protein |
| Aradu.7U9GT | 0.02 | Werner Syndrome-like exonuclease-like [Glycine max]; IPR012337 (Ribonuclease H-like domain); GO:0003676 (nucleic acid binding), GO:0006139 (nucleobase-containing compound metabolic process), GO:0008408 (3'-5' exonuclease activity) |
| Aradu.8TT29 | 0.01 | UPF0481 protein At3g47200-like [Glycine max]; IPR004158 (Protein of unknown function DUF247, plant) |
| Aradu.8XF51 | 0.02 | zinc finger MYM-type protein 1-like [Glycine max]; IPR008906 (HAT dimerisation domain, C-terminal), IPR012337 (Ribonuclease H-like domain); GO:0003676 (nucleic acid binding), GO:0046983 (protein dimerization activity) |
| Aradu.9F13E | 0.01 | uncharacterized protein LOC102664163 isoform X7 [Glycine max]; IPR004252 (Probable transposase, Ptta/En/Spm, plant) |
| Aradu.9UH7N | 0.06 | plasma membrane H+-ATPase |
| Aradu.A3JGJ | 0.06 | squalene monooxygenase 2 |
| Aradu.AQ9AW | 0.05 | Ycf1 n=12 Tax=Theaceae RepID=L0E7K8_CAMSI; IPR008896 (Uncharacterised protein family Ycf1) |
| Aradu.BFU4E | 0.01 | Unknown protein; IPR015300 (DNA-binding pseudobarrel domain) |
| Aradu.BH0FU | 0.04 | Unknown protein |
| Aradu.BV0EF | 0.03 | myosin 1 |
| Aradu.C6Z0L | 0.03 | zinc finger MYM-type protein 1-like [Glycine max] |
| Aradu.CK8F4 | 0.03 | Galactose oxidase/kelch repeat superfamily protein; IPR015916 (Galactose oxidase, beta-propeller) |
| Aradu.CQI3F | 0.04 | uncharacterized protein LOC102660738 isoform X3 [Glycine max] |
| Aradu.DX6PW | 0.03 | Unknown protein |
| Aradu.EL08S | 0.05 | Unknown protein |
| Aradu.G3X0J | 0.12 | Protein phosphatase 2C family protein; IPR001932 (Protein phosphatase 2C (PP2C)-like domain), IPR015655 (Protein phosphatase 2C); GO:0003824 (catalytic activity) |
| Aradu.H0FSY | 0.03 | serine/threonine-protein phosphatase 7 long form homolog [Glycine max]; IPR019557 (Aminotransferase-like, plant mobile domain) |
| Aradu.I0Y06 | 0.04 | DNA/RNA polymerases superfamily protein n=1 Tax=Theobroma cacao RepID=UPI00042B5D03; IPR001878 (Zinc finger, CCHC-type); GO:0003676 (nucleic acid binding), GO:0008270 (zinc ion binding) |
| Aradu.I6NY4 | 0.01 | protein FAR1-RELATED SEQUENCE 6-like isoform X2 [Glycine max]; IPR004330 (FAR1 DNA binding domain), IPR007527 (Zinc finger, SWIM-type); GO:0008270 (zinc ion binding) |
| Aradu.JP3EF | 0.03 | quinone oxidoreductase; IPR005025 (NADPH-dependent FMN reductase-like), IPR010089 (Flavoprotein WrbA); GO:0010181 (FMN binding), GO:0016491 (oxidoreductase activity) |
| Aradu.KA2N1 | 0.03 | eukaryotic translation initiation factor 3B-2; IPR011400 (Eukaryotic translation initiation factor 3 subunit B); GO:0003723 (RNA binding), GO:0003743 (translation initiation factor activity), GO:0005515 (protein binding), GO:0005852 (eukaryotic translation initiation factor 3 complex), GO:0006413 (translational initiation), GO:0031369 (translation initiation factor binding) |
| Aradu.KH3GJ | 0.04 | Unknown protein |
| Aradu.M6NC2 | 0.11 | Transposon protein n=1 Tax=Arachis hypogaea RepID=G0Y6V7_ARAHY |
| Aradu.M9FDP | 0.04 | FAR1 DNA-binding domain protein |
| Aradu.P28V8 | 0.03 | Unknown protein |
| Aradu.U0HR0 | 0.02 | serine/threonine-protein phosphatase 7 long form homolog isoform X2 [Glycine max] |
| Aradu.UTN5Z | 0.04 | Unknown protein |
| Aradu.V3782 | 0.07 | 60S ribosomal protein L18-3; IPR000039 (Ribosomal protein L18e), IPR021131 (Ribosomal protein L18e/L15P); GO:0003735 (structural constituent of ribosome), GO:0005622 (intracellular), GO:0005840 (ribosome), GO:0006412 (translation) |
| Aradu.V9DPL | 0.02 | serine/threonine-protein phosphatase 7 long form homolog [Glycine max]; IPR019557 (Aminotransferase-like, plant mobile domain) |
| Aradu.X2RMT | 0.07 | Unknown protein |
| Aradu.XJ1WR | 0.01 | white-brown-complex ABC transporter family protein |
| Aradu.Z73VS | 0.02 | uncharacterized protein LOC100778164 isoform X2 [Glycine max]; IPR027417 (P-loop containing nucleoside triphosphate hydrolase); GO:0005524 (ATP binding) |
| Aradu.Z8JLA | 0.06 | Ankyrin repeat family protein |
| Araip.2G0N0 | 0.04 | serine/threonine-protein phosphatase 7 long form homolog [Glycine max]; IPR019557 (Aminotransferase-like, plant mobile domain) |
| Araip.3JS0F | 0.02 | pectinesterase 11; IPR011050 (Pectin lyase fold/virulence factor); GO:0005618 (cell wall), GO:0030599 (pectinesterase activity), GO:0042545 (cell wall modification) |
| Araip.4J2X2 | 0.04 | Unknown protein |
| Araip.565UA | 0.04 | 125 kDa kinesin-like protein |
| Araip.60USQ | 0.02 | expansin B2; IPR007118 (Expansin/Lol pI); GO:0005576 (extracellular region), GO:0019953 (sexual reproduction) |
| Araip.67HNL | 0.02 | Unknown protein |
| Araip.6Q2T6 | 0.05 | Unknown protein |
| Araip.9WZ3H | 0.04 | plasma membrane H+-ATPase; IPR001757 (Cation-transporting P-type ATPase), IPR023214 (HAD-like domain), IPR023298 (P-type ATPase, transmembrane domain); GO:0006812 (cation transport), GO:0016021 (integral component of membrane), GO:0019829 (cation-transporting ATPase activity) |
| Araip.C5U3A | 0.02 | glucan endo-1,3-beta-glucosidase 11-like [Glycine max]; IPR000490 (Glycoside hydrolase, family 17), IPR017853 (Glycoside hydrolase, superfamily); GO:0005975 (carbohydrate metabolic process) |
| Araip.DEM20 | 0.01 | protein FAR1-RELATED SEQUENCE 6-like isoform X2 [Glycine max]; IPR004330 (FAR1 DNA binding domain), IPR007527 (Zinc finger, SWIM-type); GO:0008270 (zinc ion binding) |
| Araip.DKQ1J | 0.02 | ribosomal RNA small subunit methyltransferase G; IPR003682 (rRNA small subunit methyltransferase G); GO:0005737 (cytoplasm), GO:0006364 (rRNA processing), GO:0008649 (rRNA methyltransferase activity) |
| Araip.E9VC4 | 0.02 | zinc finger MYM-type protein 1-like [Glycine max]; IPR008906 (HAT dimerisation domain, C-terminal), IPR012337 (Ribonuclease H-like domain); GO:0003676 (nucleic acid binding), GO:0046983 (protein dimerization activity) |
| Araip.FKZ6T | 0.01 | serine/threonine-protein phosphatase 7 long form homolog [Glycine max]; IPR019557 (Aminotransferase-like, plant mobile domain) |
| Araip.FX3SL | 0.11 | Pentatricopeptide repeat (PPR-like) superfamily protein; IPR002885 (Pentatricopeptide repeat) |
| Araip.G1HKM | 0.07 | Mitochondrial import inner membrane translocase subunit Tim17/Tim22/Tim23 family protein |
| Araip.GI6B7 | 0.03 | Eukaryotic aspartyl protease family protein; IPR001461 (Aspartic peptidase), IPR021109 (Aspartic peptidase domain); GO:0004190 (aspartic-type endopeptidase activity), GO:0006508 (proteolysis) |
| Araip.H1BIY | 0.03 | CTV.20 n=1 Tax=Poncirus trifoliata RepID=Q8H6Q8_PONTR |
| Araip.H2BM2 | 0.03 | Helicase-like protein n=1 Tax=Oryza sativa subsp. japonica RepID=Q6YTQ6_ORYSJ |
| Araip.H5T14 | 0.01 | Pentatricopeptide repeat (PPR) superfamily protein; IPR002885 (Pentatricopeptide repeat) |
| Araip.H63GE | 0.03 | NADP-dependent alkenal double bond reductase P1; IPR002085 (Alcohol dehydrogenase superfamily, zinc-type), IPR013149 (Alcohol dehydrogenase, C-terminal), IPR016040 (NAD(P)-binding domain); GO:0008270 (zinc ion binding), GO:0016491 (oxidoreductase activity), GO:0055114 (oxidation-reduction process) |
| Araip.HG9KX | 0.04 | ankyrin repeat plant-like protein |
| Araip.HMA4X | 0.03 | Unknown protein |
| Araip.HU375 | 0.01 | pleiotropic drug resistance 4; IPR003210 (Signal recognition particle, SRP14 subunit), IPR009018 (Signal recognition particle, SRP9/SRP14 subunit), IPR027417 (P-loop containing nucleoside triphosphate hydrolase); GO:0005524 (ATP binding), GO:0006614 (SRP-dependent cotranslational protein targeting to membrane), GO:0008312 (7S RNA binding), GO:0016887 (ATPase activity), GO:0030942 (endoplasmic reticulum signal peptide binding), GO:0048500 (signal recognition particle) |
| Araip.I5JFE | 0.04 | chromosome-associated kinesin KIF4A-like isoform X2 [Glycine max] |
| Araip.IU3P3 | 0.02 | Peroxidase superfamily protein; IPR010255 (Haem peroxidase); GO:0004601 (peroxidase activity), GO:0006979 (response to oxidative stress), GO:0020037 (heme binding), GO:0055114 (oxidation-reduction process) |
| Araip.KLZ0I | 0.03 | 2-oxoglutarate (2OG) and Fe(II)-dependent oxygenase superfamily protein |
| Araip.LV1NA | 0.03 | GRF zinc finger protein; IPR010666 (Zinc finger, GRF-type); GO:0008270 (zinc ion binding) |
| Araip.M3BT5 | 0.08 | mediator of RNA polymerase II transcription subunit 16 |
| Araip.P4BIR | 0.1 | uncharacterized protein LOC102666784 [Glycine max]; IPR007527 (Zinc finger, SWIM-type); GO:0008270 (zinc ion binding) |
| Araip.P89M7 | 0.02 | uncharacterized protein LOC102666784 [Glycine max]; IPR007527 (Zinc finger, SWIM-type); GO:0008270 (zinc ion binding) |
| Araip.S2YLH | 0.02 | E3 ubiquitin-protein ligase n=5 Tax=Solanum RepID=M1BDZ1_SOLTU |
| Araip.SXN7B | 0.03 | Unknown protein |
| Araip.TCH3N | 0.02 | RNA-binding LUC7-like protein; IPR004882 (Luc7-related); GO:0003729 (mRNA binding), GO:0005685 (U1 snRNP), GO:0006376 (mRNA splice site selection) |
| Araip.TL0B5 | 0.03 | NAC domain protein,; IPR003441 (NAC domain); GO:0003677 (DNA binding) |
| Araip.VJ4A8 | 0.01 | Unknown protein |
| Araip.Y7C0R | 0.04 | Pyruvate kinase family protein; IPR001697 (Pyruvate kinase); GO:0000287 (magnesium ion binding), GO:0003824 (catalytic activity), GO:0004743 (pyruvate kinase activity), GO:0006096 (glycolysis), GO:0030955 (potassium ion binding) |
| Araip.YF5UN | 0.03 | Zinc finger GRF-type protein n=1 Tax=Arachis hypogaea RepID=G0Y6U2_ARAHY; IPR010666 (Zinc finger, GRF-type); GO:0008270 (zinc ion binding) |
| Araip.ZV274 | 0.01 | 1-deoxy-D-xylulose 5-phosphate synthase 3; IPR005476 (Transketolase, C-terminal), IPR009014 (Transketolase, C-terminal/Pyruvate-ferredoxin oxidoreductase, domain II); GO:0003824 (catalytic activity), GO:0008152 (metabolic process) |
| Pattee 5 pericarp | |  |
| Aradu.035M0 | 0.04 | ATP phosphoribosyl transferase 1 |
| Aradu.0J08R | 0.04 | F-box/FBD-like domain protein, putative |
| Aradu.0X2PN | 0.01 | Ulp1 protease family, carboxy-terminal domain protein; IPR003653 (Peptidase C48, SUMO/Sentrin/Ubl1); GO:0006508 (proteolysis), GO:0008234 (cysteine-type peptidase activity) |
| Aradu.3T9SJ | 0.01 | protein FAR1-RELATED SEQUENCE 6-like isoform X2 [Glycine max]; IPR004330 (FAR1 DNA binding domain), IPR006564 (Zinc finger, PMZ-type); GO:0008270 (zinc ion binding) |
| Aradu.4HW4H | 0.01 | serine/threonine-protein phosphatase 7 long form homolog [Glycine max]; IPR019557 (Aminotransferase-like, plant mobile domain) |
| Aradu.81SR8 | 0.01 | Protein kinase superfamily protein; IPR011009 (Protein kinase-like domain); GO:0004672 (protein kinase activity), GO:0005524 (ATP binding), GO:0006468 (protein phosphorylation) |
| Aradu.87AIU | 0.04 | ethylene-responsive transcription factor 1B; IPR016177 (DNA-binding domain); GO:0003677 (DNA binding), GO:0003700 (sequence-specific DNA binding transcription factor activity) |
| Aradu.9W5KV | 0.01 | serine/threonine-protein phosphatase 7 long form homolog [Glycine max]; IPR019557 (Aminotransferase-like, plant mobile domain) |
| Aradu.9Y8SB | 0.03 | disease resistance protein (TIR-NBS-LRR class), putative |
| Aradu.DP96I | 0.02 | ER membrane protein complex subunit-like protein |
| Aradu.E8C62 | 0.01 | protein FAR1-RELATED SEQUENCE 6-like isoform X2 [Glycine max] |
| Aradu.GAR5M | 0.02 | Cytochrome P450 superfamily protein; IPR001128 (Cytochrome P450); GO:0005506 (iron ion binding), GO:0020037 (heme binding), GO:0055114 (oxidation-reduction process) |
| Aradu.H4UD2 | 0.1 | dynamin-like protein; IPR001401 (Dynamin, GTPase domain), IPR022812 (Dynamin superfamily), IPR027417 (P-loop containing nucleoside triphosphate hydrolase); GO:0003924 (GTPase activity), GO:0005525 (GTP binding) |
| Aradu.I1SE2 | 0.01 | serine/threonine-protein phosphatase 7 long form homolog [Glycine max]; IPR019557 (Aminotransferase-like, plant mobile domain) |
| Aradu.J1HX8 | 0.03 | GRF zinc finger protein; IPR010666 (Zinc finger, GRF-type); GO:0008270 (zinc ion binding) |
| Aradu.L12SK | 0.02 | Unknown protein |
| Aradu.LWM6R | 0.02 | nucleolin 1-like [Glycine max] |
| Aradu.NR27U | 0.01 | protein FAR1-RELATED SEQUENCE 6-like isoform X2 [Glycine max]; IPR006564 (Zinc finger, PMZ-type); GO:0008270 (zinc ion binding) |
| Aradu.NXH1R | 0.01 | pollen-specific protein SF21-like [Glycine max]; IPR004142 (NDRG) |
| Aradu.P4Q1J | 0.04 | Unknown protein |
| Aradu.Q0XNV | 0.01 | Cysteine proteinases superfamily protein; IPR013128 (Peptidase C1A); GO:0006508 (proteolysis), GO:0008234 (cysteine-type peptidase activity) |
| Aradu.R70YZ | 0.01 | Replication factor-A protein 1-related; IPR012340 (Nucleic acid-binding, OB-fold) |
| Aradu.S283I | 0.02 | protein FAR1-RELATED SEQUENCE 6-like isoform X2 [Glycine max]; IPR004330 (FAR1 DNA binding domain), IPR007527 (Zinc finger, SWIM-type); GO:0008270 (zinc ion binding) |
| Aradu.S5R9L | 0.05 | MADS-box transcription factor; IPR002487 (Transcription factor, K-box); GO:0003700 (sequence-specific DNA binding transcription factor activity), GO:0005634 (nucleus) |
| Aradu.T8YI8 | 0.01 | protein FAR1-RELATED SEQUENCE 5-like [Glycine max]; IPR001878 (Zinc finger, CCHC-type), IPR004330 (FAR1 DNA binding domain); GO:0003676 (nucleic acid binding), GO:0008270 (zinc ion binding) |
| Aradu.U1QSJ | 0.01 | zinc finger MYM-type protein 1-like [Glycine max]; IPR012337 (Ribonuclease H-like domain), IPR025398 (Domain of unknown function DUF4371); GO:0003676 (nucleic acid binding) |
| Aradu.YCF1P | 0.01 | protein FAR1-RELATED SEQUENCE 6-like isoform X2 [Glycine max]; IPR004330 (FAR1 DNA binding domain), IPR007527 (Zinc finger, SWIM-type); GO:0008270 (zinc ion binding) |
| Aradu.Z4C55 | 0.01 | Unknown protein |
| Araip.22E3Q | 0.05 | DnaJ / Sec63 Brl domains-containing protein; IPR004179 (Sec63 domain) |
| Araip.24AAU | 0.01 | Ulp1 protease family, carboxy-terminal domain protein |
| Araip.356RR | 0.07 | Unknown protein |
| Araip.44MN6 | 0.01 | Unknown protein |
| Araip.44P1W | 0.02 | alpha/beta-Hydrolases superfamily protein |
| Araip.4M17K | 0.02 | Unknown protein |
| Araip.4N16E | 0.04 | Superoxide dismutase [Cu-Zn] n=5 Tax=Populus RepID=B9; IPR001424 (Superoxide dismutase, copper/zinc binding domain), IPR003347 (JmjC domain); GO:0005515 (protein binding), GO:0006801 (superoxide metabolic process), GO:0046872 (metal ion binding), GO:0055114 (oxidation-reduction process) |
| Araip.66198 | 0.04 | syntaxin, putative; IPR010989 (t-SNARE); GO:0016020 (membrane), GO:0016192 (vesicle-mediated transport) |
| Araip.7Q8EK | 0.02 | Unknown protein |
| Araip.9E1QG | 0.12 | LRR and NB-ARC domain disease resistance protein |
| Araip.9I0IM | 0.05 | Unknown protein |
| Araip.E5QEE | 0.01 | Unknown protein |
| Araip.H1YPT | 0.02 | 4-coumarate--CoA ligase-like 9-like [Glycine max] |
| Araip.HW8PR | 0.01 | serine/threonine-protein phosphatase 7 long form homolog [Glycine max]; IPR019557 (Aminotransferase-like, plant mobile domain) |
| Araip.I1DI3 | 0.01 | cytochrome C biogenesis protein ccsA, putative; IPR001810 (F-box domain); GO:0005515 (protein binding) |
| Araip.I2LNQ | 0.05 | protein YLS9-like [Glycine max] |
| Araip.IB68Z | 0.07 | Unknown protein |
| Araip.M0HLL | 0.01 | protein FAR1-RELATED SEQUENCE 6-like isoform X2 [Glycine max]; IPR004330 (FAR1 DNA binding domain), IPR006564 (Zinc finger, PMZ-type); GO:0008270 (zinc ion binding) |
| Araip.M9QAF | 0.07 | Galactosyltransferase family protein; IPR002659 (Glycosyl transferase, family 31); GO:0006486 (protein glycosylation), GO:0008378 (galactosyltransferase activity), GO:0016020 (membrane) |
| Araip.MH0EC | 0.05 | Unknown protein |
| Araip.Q22J4 | 0.03 | Protein kinase superfamily protein; IPR011009 (Protein kinase-like domain); GO:0004672 (protein kinase activity), GO:0006468 (protein phosphorylation) |
| Araip.Q8BQ9 | 0.04 | hypothetical protein |
| Araip.RMA3D | 0.03 | zinc finger MYM-type protein 1-like [Glycine max]; IPR025398 (Domain of unknown function DUF4371) |
| Araip.SCE0X | 0.05 | zinc finger MYM-type protein 1-like [Glycine max] |
| Araip.T7TLT | 0.04 | Unknown protein |
| Araip.UYE2H | 0.04 | zinc finger MYM-type protein 1-like [Glycine max]; IPR008906 (HAT dimerisation domain, C-terminal), IPR012337 (Ribonuclease H-like domain); GO:0003676 (nucleic acid binding), GO:0046983 (protein dimerization activity) |
| Araip.VK5CJ | 0.04 | Unknown protein |
| Araip.WA8YD | 0.04 | Plant self-incompatibility protein S1 family; IPR010264 (Plant self-incompatibility S1) |
| Araip.WQG5H | 0.02 | Unknown protein |
| Araip.XC4CW | 0.01 | Ulp1 protease family, carboxy-terminal domain protein; IPR003653 (Peptidase C48, SUMO/Sentrin/Ubl1); GO:0006508 (proteolysis), GO:0008234 (cysteine-type peptidase activity) |
| Araip.XG71D | 0.02 | uncharacterized protein LOC102668833 isoform X2 [Glycine max] |
| Pattee 5 seed | |  |
| Aradu.01PFD | 0.13 | Unknown protein |
| Aradu.08DL3 | 0.04 | serine/threonine-protein phosphatase 7 long form homolog [Glycine max]; IPR019557 (Aminotransferase-like, plant mobile domain) |
| Aradu.0L0WF | 0.09 | uncharacterized protein LOC100786942 [Glycine max] |
| Aradu.0Z5TM | 0.06 | deoxyuridine 5'-triphosphate nucleotidohydrolase; IPR008180 (Deoxyuridine triphosphate nucleotidohydrolase/Deoxycytidine triphosphate deaminase); GO:0016787 (hydrolase activity), GO:0046080 (dUTP metabolic process) |
| Aradu.1W88T | 0.21 | Unknown protein |
| Aradu.2IJ75 | 0.14 | Replication factor A protein n=1 Tax=Medicago truncatula RepID=G7K1P7_MEDTR |
| Aradu.2M0JM | 0.01 | GDSL esterase/lipase [Glycine max]; IPR013831 (SGNH hydrolase-type esterase domain), IPR022742 (Putative lysophospholipase); GO:0016787 (hydrolase activity) |
| Aradu.2ZD5V | 0.1 | Unknown protein |
| Aradu.3D651 | 0.02 | ribosomal RNA small subunit methyltransferase G; IPR003682 (rRNA small subunit methyltransferase G); GO:0005737 (cytoplasm), GO:0006364 (rRNA processing), GO:0008649 (rRNA methyltransferase activity) |
| Aradu.3HN8P | 0.01 | Unknown protein |
| Aradu.3Q8BC | 0.06 | Unknown protein |
| Aradu.41DQD | 0.06 | ARM REPEAT PROTEIN INTERACTING WITH ABF2-like isoform X2 [Glycine max]; IPR016024 (Armadillo-type fold); GO:0005488 (binding) |
| Aradu.48HYY | 0.03 | RING finger protein 38-like [Glycine max]; IPR013083 (Zinc finger, RING/FYVE/PHD-type); GO:0005515 (protein binding), GO:0008270 (zinc ion binding) |
| Aradu.4E759 | 0.04 | Unknown protein |
| Aradu.4XZ1U | 0.02 | Ulp1 protease family, carboxy-terminal domain protein |
| Aradu.536QG | 0.03 | protein n=1 Tax=Oryza sativa subsp. japonica RepID=C7IY26_ORYSJ; IPR007527 (Zinc finger, SWIM-type); GO:0008270 (zinc ion binding) |
| Aradu.58C5A | 0.11 | Unknown protein; IPR010851 (S locus-related glycoprotein 1 binding pollen coat) |
| Aradu.5E6Y1 | 0.01 | WD repeat-containing protein 48-like isoform X1 [Glycine max]; IPR015943 (WD40/YVTN repeat-like-containing domain); GO:0005515 (protein binding) |
| Aradu.5QB68 | 0.01 | animal RPA1 domain protein; IPR012340 (Nucleic acid-binding, OB-fold) |
| Aradu.5Y8V4 | 0.01 | drug resistance transporter-like ABC domain protein |
| Aradu.68TD6 | 0.04 | Unknown protein |
| Aradu.6Q40C | 0.04 | Protein kinase superfamily protein; IPR011009 (Protein kinase-like domain), IPR013320 (Concanavalin A-like lectin/glucanase, subgroup); GO:0004672 (protein kinase activity), GO:0006468 (protein phosphorylation) |
| Aradu.75565 | 0.01 | E3 ubiquitin-protein ligase n=5 Tax=Solanum RepID=M1BDZ1_SOLTU |
| Aradu.7SJ8W | 0.03 | zinc finger CCCH domain-containing protein 34-like [Glycine max]; IPR000571 (Zinc finger, CCCH-type); GO:0046872 (metal ion binding) |
| Aradu.80VNE | 0.02 | Unknown protein |
| Aradu.88JR3 | 0.02 | beta-galactosidase 8; IPR001251 (CRAL-TRIO domain), IPR001944 (Glycoside hydrolase, family 35), IPR017853 (Glycoside hydrolase, superfamily); GO:0005975 (carbohydrate metabolic process) |
| Aradu.8IR6G | 0.04 | FAR-RED impaired response-like protein; IPR004330 (FAR1 DNA binding domain) |
| Aradu.9BQ6M | 0.1 | FKBP-like peptidyl-prolyl cis-trans isomerase family protein |
| Aradu.9Y96E | 0.02 | expansin-B1-like protein; IPR007117 (Expansin, cellulose-binding-like domain), IPR015898 (G-protein gamma-like domain); GO:0004871 (signal transducer activity), GO:0005834 (heterotrimeric G-protein complex), GO:0007186 (G-protein coupled receptor signaling pathway) |
| Aradu.A4MFG | 0.04 | Cell wall-associated hydrolase n=3 Tax=Streptomyces RepID=B5HM15_9ACTO |
| Aradu.AJ0BB | 0.04 | FAR1 DNA-binding domain protein |
| Aradu.B1IPV | 0.01 | Unknown protein |
| Aradu.B1PIM | 0.06 | unknown protein; FUNCTIONS IN: molecular_function unknown; INVOLVED IN: biological_process unknown; LOCATED IN: cellular_component unknown |
| Aradu.B7ZN8 | 0.03 | protein FAR1-RELATED SEQUENCE 6-like isoform 1 [Glycine max]; IPR004330 (FAR1 DNA binding domain) |
| Aradu.BG871 | 0.04 | uncharacterized protein LOC100527109 [Glycine max] |
| Aradu.BYN2Y | 0.05 | protein disulfide isomerase (PDI)-like protein |
| Aradu.C6JCF | 0.04 | lysine ketoglutarate reductase trans-splicing-like protein; IPR007877 (Protein of unknown function DUF707) |
| Aradu.C8HB7 | 0.03 | putative ubiquitin-like-specific protease 1B-like isoform X2 [Glycine max]; IPR003653 (Peptidase C48, SUMO/Sentrin/Ubl1); GO:0006508 (proteolysis), GO:0008234 (cysteine-type peptidase activity) |
| Aradu.C8WPL | 0.04 | Unknown protein |
| Aradu.C9GS0 | 0.04 | Unknown protein |
| Aradu.CK1D7 | 0.18 | B3 domain transcription factor VRN1-like protein; IPR015300 (DNA-binding pseudobarrel domain) |
| Aradu.CR4J4 | 0.04 | Unknown protein |
| Aradu.CVT5N | 0.03 | blue copper protein-like [Glycine max]; IPR008972 (Cupredoxin); GO:0005507 (copper ion binding), GO:0009055 (electron carrier activity) |
| Aradu.E5ND6 | 0.04 | replication factor-A carboxy-terminal domain protein; IPR012340 (Nucleic acid-binding, OB-fold) |
| Aradu.EL8GM | 0.05 | Unknown protein |
| Aradu.F3JW2 | 0.03 | Pentatricopeptide repeat (PPR-like) superfamily protein; IPR000571 (Zinc finger, CCCH-type), IPR002885 (Pentatricopeptide repeat), IPR015943 (WD40/YVTN repeat-like-containing domain); GO:0005515 (protein binding), GO:0046872 (metal ion binding) |
| Aradu.F4TB9 | 0.01 | replication factor-A carboxy-terminal domain protein; IPR012340 (Nucleic acid-binding, OB-fold) |
| Aradu.F79GF | 0.07 | Unknown protein |
| Aradu.G00RX | 0.07 | Unknown protein |
| Aradu.G13NV | 0.01 | B3 DNA-binding domain protein; IPR015300 (DNA-binding pseudobarrel domain); GO:0003677 (DNA binding) |
| Aradu.GK38A | 0.03 | isocitrate dehydrogenase; IPR004790 (Isocitrate dehydrogenase NADP-dependent), IPR024084 (Isopropylmalate dehydrogenase-like domain); GO:0004450 (isocitrate dehydrogenase (NADP+) activity), GO:0006102 (isocitrate metabolic process), GO:0055114 (oxidation-reduction process) |
| Aradu.HS2F0 | 0.01 | replication factor-A carboxy-terminal domain protein; IPR012340 (Nucleic acid-binding, OB-fold) |
| Aradu.I45ZR | 0.05 | protein FAR1-RELATED SEQUENCE 5-like [Glycine max] |
| Aradu.IZ7EQ | 0.03 | plant-specific B3-DNA-binding domain protein; IPR015300 (DNA-binding pseudobarrel domain) |
| Aradu.J2384 | 0.02 | PIF1-like helicase |
| Aradu.J4WN1 | 0.07 | Unknown protein |
| Aradu.JTI8T | 0.02 | exocyst subunit exo70 family protein B2; IPR004140 (Exocyst complex protein Exo70), IPR016159 (Cullin repeat-like-containing domain); GO:0000145 (exocyst), GO:0006887 (exocytosis) |
| Aradu.K08VH | 0.09 | PsbP-like protein 2; IPR016123 (Mog1/PsbP, alpha/beta/alpha sandwich) |
| Aradu.KG23Q | 0.01 | putative ubiquitin-like-specific protease 1B-like isoform X3 [Glycine max]; IPR003653 (Peptidase C48, SUMO/Sentrin/Ubl1); GO:0006508 (proteolysis), GO:0008234 (cysteine-type peptidase activity) |
| Aradu.KKR6V | 0.01 | Unknown protein |
| Aradu.KXS0C | 0.04 | Unknown protein |
| Aradu.L3EXW | 0.04 | Unknown protein |
| Aradu.M0KHJ | 0.02 | protein FAR1-RELATED SEQUENCE 5-like [Glycine max]; IPR001878 (Zinc finger, CCHC-type), IPR004330 (FAR1 DNA binding domain), IPR006564 (Zinc finger, PMZ-type), IPR015813 (Pyruvate/Phosphoenolpyruvate kinase-like domain); GO:0003676 (nucleic acid binding), GO:0003824 (catalytic activity), GO:0006725 (cellular aromatic compound metabolic process), GO:0008270 (zinc ion binding), GO:0016830 (carbon-carbon lyase activity) |
| Aradu.M9SGQ | 0.07 | RING-H2 finger protein [Glycine max]; IPR013083 (Zinc finger, RING/FYVE/PHD-type); GO:0005515 (protein binding), GO:0008270 (zinc ion binding) |
| Aradu.MC55G | 0.14 | probable transcription factor PosF21-like [Glycine max]; IPR004827 (Basic-leucine zipper domain); GO:0003700 (sequence-specific DNA binding transcription factor activity), GO:0043565 (sequence-specific DNA binding) |
| Aradu.MY047 | 0.09 | MADS-box transcription factor family protein; IPR002100 (Transcription factor, MADS-box); GO:0003677 (DNA binding), GO:0046983 (protein dimerization activity) |
| Aradu.N2WUV | 0.03 | Myb/SANT-like DNA-binding domain protein; IPR024752 (Myb/SANT-like domain) |
| Aradu.N55MN | 0.04 | Unknown protein; IPR011989 (Armadillo-like helical) |
| Aradu.N5L6U | 0.2 | Unknown protein |
| Aradu.NA64A | 0.14 | Unknown protein |
| Aradu.NIH31 | 0.01 | F-box protein interaction domain protein; IPR001810 (F-box domain), IPR017451 (F-box associated interaction domain); GO:0005515 (protein binding) |
| Aradu.NJ2KD | 0.39 | protein disulfide isomerase (PDI)-like protein |
| Aradu.NKS1T | 0.04 | Unknown protein |
| Aradu.NSW78 | 0.12 | serine/threonine-protein phosphatase 7 long form homolog [Glycine max]; IPR019557 (Aminotransferase-like, plant mobile domain) |
| Aradu.P68ES | 0.07 | Unknown protein |
| Aradu.PHY87 | 0.03 | sucrose transporter 4 |
| Aradu.PQQ6F | 0.06 | Unknown protein |
| Aradu.QD8D8 | 0.04 | Unknown protein |
| Aradu.QW73F | 0.12 | Unknown protein |
| Aradu.R3QAL | 0.02 | serine/threonine-protein phosphatase 7 long form homolog [Glycine max]; IPR019557 (Aminotransferase-like, plant mobile domain) |
| Aradu.R9DNV | 0.23 | Unknown protein |
| Aradu.RBF8P | 0.03 | Transposon protein, putative, CACTA, En/Spm sub-class, expressed n=1 Tax=Oryza sativa subsp. japonica RepID=Q7G4H8_ORYSJ |
| Aradu.RH00F | 0.02 | zinc finger CCCH domain-containing protein 48-like isoform X2 [Glycine max] |
| Aradu.RYK4N | 0.04 | putative serine/threonine-protein kinase-like protein CCR3-like [Glycine max] |
| Aradu.S3Q6Y | 0.01 | serine/threonine-protein phosphatase 7 long form homolog [Glycine max]; IPR019557 (Aminotransferase-like, plant mobile domain) |
| Aradu.SI01H | 0.01 | Disease resistance protein (TIR-NBS class); IPR000767 (Disease resistance protein), IPR027417 (P-loop containing nucleoside triphosphate hydrolase); GO:0006952 (defense response) |
| Aradu.SN6SG | 0.04 | unknown protein |
| Aradu.TL54T | 0.06 | Unknown protein |
| Aradu.TZ4CE | 0.09 | Unknown protein |
| Aradu.UH3N5 | 0.4 | Unknown protein |
| Aradu.V7UXH | 0.01 | protein FAR1-RELATED SEQUENCE 5-like [Glycine max]; IPR001878 (Zinc finger, CCHC-type), IPR004330 (FAR1 DNA binding domain), IPR006564 (Zinc finger, PMZ-type); GO:0003676 (nucleic acid binding), GO:0008270 (zinc ion binding) |
| Aradu.V9ADB | 0.03 | cystathionine beta-lyase; IPR000277 (Cys/Met metabolism, pyridoxal phosphate-dependent enzyme); GO:0003824 (catalytic activity), GO:0030170 (pyridoxal phosphate binding) |
| Aradu.VNL2Q | 0.07 | Zinc finger GRF-type protein n=1 Tax=Arachis hypogaea RepID=G0Y6U2_ARAHY |
| Aradu.VTW1V | 0.02 | cell division control protein 48 homolog C-like isoform X1 [Glycine max]; IPR015425 (Formin, FH2 domain), IPR027417 (P-loop containing nucleoside triphosphate hydrolase); GO:0000166 (nucleotide binding), GO:0005524 (ATP binding), GO:0017111 (nucleoside-triphosphatase activity) |
| Aradu.VZ1DU | 0.02 | Ulp1 protease family, carboxy-terminal domain protein |
| Aradu.W5VJT | 0.01 | uncharacterized mitochondrial protein AtMg00860-like [Glycine max] |
| Aradu.W7ZP2 | 0.07 | Unknown protein |
| Aradu.W9AGV | 0.05 | Myb/SANT-like DNA-binding domain protein; IPR024752 (Myb/SANT-like domain) |
| Aradu.WW4W8 | 0.02 | protein FAR1-RELATED SEQUENCE 5-like [Glycine max] |
| Aradu.WX9EY | 0.06 | Unknown protein; IPR001568 (Ribonuclease T2-like); GO:0003723 (RNA binding), GO:0033897 (ribonuclease T2 activity) |
| Aradu.XC0HA | 0.25 | Unknown protein |
| Aradu.XG1KZ | 0.01 | heat shock protein 70; IPR013126 (Heat shock protein 70 family) |
| Aradu.Y1FLP | 0.07 | 40s ribosomal protein SA; IPR001865 (Ribosomal protein S2), IPR023591 (Ribosomal protein S2, flavodoxin-like domain); GO:0003735 (structural constituent of ribosome), GO:0005622 (intracellular), GO:0005840 (ribosome), GO:0006412 (translation), GO:0015935 (small ribosomal subunit) |
| Aradu.YP3L5 | 0.04 | cellulose synthase family protein; IPR005150 (Cellulose synthase); GO:0016020 (membrane), GO:0016760 (cellulose synthase (UDP-forming) activity), GO:0030244 (cellulose biosynthetic process) |
| Aradu.YT60S | 0.32 | Unknown protein |
| Aradu.Z0XWC | 0.08 | prohibitin 2 |
| Aradu.Z9PNV | 0.1 | uridylate kinase; IPR001048 (Aspartate/glutamate/uridylate kinase) |
| Araip.027N6 | 0.02 | MATE efflux family protein; IPR002528 (Multi antimicrobial extrusion protein); GO:0006855 (drug transmembrane transport), GO:0015238 (drug transmembrane transporter activity), GO:0015297 (antiporter activity), GO:0016020 (membrane), GO:0055085 (transmembrane transport) |
| Araip.04M1L | 0.39 | Unknown protein |
| Araip.0A4TN | 0.07 | Unknown protein; IPR019557 (Aminotransferase-like, plant mobile domain) |
| Araip.0YK8G | 0.02 | serine/threonine-protein phosphatase 7 long form homolog [Glycine max]; IPR019557 (Aminotransferase-like, plant mobile domain) |
| Araip.1P1FF | 0.01 | 60S ribosomal protein L2, mitochondrial-like [Glycine max]; IPR012340 (Nucleic acid-binding, OB-fold) |
| Araip.22DHI | 0.03 | RAC-like GTP binding protein 5 |
| Araip.22RFM | 0.03 | F-box/LRR protein; IPR006553 (Leucine-rich repeat, cysteine-containing subtype) |
| Araip.254U2 | 0.01 | Unknown protein |
| Araip.266E4 | 0.03 | Unknown protein |
| Araip.2B3QX | 0.15 | Unknown protein |
| Araip.2BR22 | 0.03 | protein FAR1-RELATED SEQUENCE 5-like [Glycine max]; IPR004330 (FAR1 DNA binding domain), IPR007527 (Zinc finger, SWIM-type); GO:0008270 (zinc ion binding) |
| Araip.2CR87 | 0.02 | drug resistance transporter-like ABC domain protein |
| Araip.2W5X5 | 0.02 | Unknown protein |
| Araip.32JR1 | 0.04 | nodulin MtN21 /EamA-like transporter family protein |
| Araip.35J1H | 0.06 | PTEN 2 |
| Araip.39TX8 | 0.02 | Unknown protein |
| Araip.3KW9Q | 0.01 | serine/threonine-protein phosphatase 7 long form homolog [Glycine max]; IPR019557 (Aminotransferase-like, plant mobile domain) |
| Araip.3NQ7F | 0.07 | LAG1 longevity assurance homolog 3; IPR006634 (TRAM/LAG1/CLN8 homology domain); GO:0016021 (integral component of membrane) |
| Araip.3T6NQ | 0.05 | F-box/LRR protein, putative |
| Araip.3YR2W | 0.02 | Ulp1 protease family, carboxy-terminal domain protein; IPR003653 (Peptidase C48, SUMO/Sentrin/Ubl1); GO:0006508 (proteolysis), GO:0008234 (cysteine-type peptidase activity) |
| Araip.4517V | 0.04 | RING-H2 finger protein 2B; IPR013083 (Zinc finger, RING/FYVE/PHD-type); GO:0005515 (protein binding), GO:0008270 (zinc ion binding) |
| Araip.46IJE | 0.14 | Unknown protein |
| Araip.4F8KA | 0.03 | F-box/kelch-repeat protein At3g06240-like [Glycine max] |
| Araip.4VJ7J | 0.33 | Unknown protein |
| Araip.57I8A | 0.04 | Unknown protein; IPR015300 (DNA-binding pseudobarrel domain); GO:0003677 (DNA binding) |
| Araip.592E0 | 0.01 | protein FAR-RED IMPAIRED RESPONSE 1-like isoform X3 [Glycine max]; IPR004330 (FAR1 DNA binding domain) |
| Araip.59SDT | 0.12 | serine/threonine-protein phosphatase 7 long form homolog isoform X2 [Glycine max] |
| Araip.5B1J1 | 0.03 | B3 domain-containing transcription factor VRN1-like isoform X1 [Glycine max]; IPR015300 (DNA-binding pseudobarrel domain) |
| Araip.5DJ4L | 0.07 | AP2-like ethylene-responsive transcription factor ANT-like [Glycine max] |
| Araip.5N4GR | 0.02 | polygalacturonase 4; IPR000743 (Glycoside hydrolase, family 28), IPR011050 (Pectin lyase fold/virulence factor); GO:0004650 (polygalacturonase activity), GO:0005975 (carbohydrate metabolic process) |
| Araip.621Z6 | 0.01 | protein FAR1-RELATED SEQUENCE 6-like isoform X2 [Glycine max]; IPR004330 (FAR1 DNA binding domain), IPR006564 (Zinc finger, PMZ-type); GO:0008270 (zinc ion binding) |
| Araip.6SW19 | 0.04 | Unknown protein |
| Araip.6WK0R | 0.07 | Unknown protein |
| Araip.742BB | 0.01 | uncharacterized protein LOC100820019 isoform X4 [Glycine max] |
| Araip.76RRN | 0.1 | cell wall / vacuolar inhibitor of fructosidase 1-like [Glycine max]; IPR006501 (Pectinesterase inhibitor domain); GO:0004857 (enzyme inhibitor activity), GO:0030599 (pectinesterase activity) |
| Araip.77K0V | 0.03 | CYCLIN D3; 1; IPR015451 (Cyclin D); GO:0007049 (cell cycle) |
| Araip.77KY5 | 0.01 | replication factor-A carboxy-terminal domain protein; IPR012340 (Nucleic acid-binding, OB-fold) |
| Araip.79CHH | 0.01 | protein FAR1-RELATED SEQUENCE 6-like isoform X2 [Glycine max]; IPR006564 (Zinc finger, PMZ-type); GO:0008270 (zinc ion binding) |
| Araip.7GN10 | 0.66 | LCR n=1 Tax=Medicago truncatula RepID=G8A2D4_MEDTR |
| Araip.7PQ7V | 0.02 | replication factor-A carboxy-terminal domain protein; IPR012340 (Nucleic acid-binding, OB-fold) |
| Araip.88CK0 | 0.02 | pyruvate orthophosphate dikinase; IPR010121 (Pyruvate, phosphate dikinase), IPR015813 (Pyruvate/Phosphoenolpyruvate kinase-like domain); GO:0003824 (catalytic activity), GO:0006090 (pyruvate metabolic process), GO:0016310 (phosphorylation) |
| Araip.8K9TD | 0.03 | Unknown protein |
| Araip.93DW8 | 0.05 | FAR1 DNA-binding domain protein; IPR004330 (FAR1 DNA binding domain) |
| Araip.93H5W | 0.08 | Unknown protein |
| Araip.9GN6Z | 0.04 | blue copper protein-like [Glycine max]; IPR008972 (Cupredoxin); GO:0005507 (copper ion binding), GO:0009055 (electron carrier activity) |
| Araip.9H3J2 | 0.01 | replication factor-A carboxy-terminal domain protein; IPR012340 (Nucleic acid-binding, OB-fold) |
| Araip.9JM8H | 0.09 | Unknown protein |
| Araip.9MQ0I | 0.05 | Unknown protein |
| Araip.9Q4TK | 0.03 | Unknown protein |
| Araip.A0C0D | 0.11 | Unknown protein; IPR004252 (Probable transposase, Ptta/En/Spm, plant) |
| Araip.A0NUW | 0.01 | serine/threonine-protein phosphatase 7 long form homolog isoform X2 [Glycine max] |
| Araip.A10I8 | 0.04 | Glycine dehydrogenase decarboxylating protein n=3 Tax=Rosaceae RepID=W8SQT8_9ROSA; IPR020581 (Glycine cleavage system P protein); GO:0004375 (glycine dehydrogenase (decarboxylating) activity), GO:0006546 (glycine catabolic process), GO:0055114 (oxidation-reduction process) |
| Araip.A37SQ | 0.03 | protein FAR1-RELATED SEQUENCE 5-like [Glycine max] |
| Araip.A527M | 0.01 | serine/threonine-protein phosphatase 7 long form homolog [Glycine max]; IPR019557 (Aminotransferase-like, plant mobile domain) |
| Araip.A5JAV | 0.03 | RING/U-box superfamily protein |
| Araip.A9KP0 | 0.1 | probable cytokinin riboside 5'-monophosphate phosphoribohydrolase LOGL5-like [Glycine max] |
| Araip.AGM8D | 0.05 | Helicase-like protein n=1 Tax=Medicago truncatula RepID=G7JLV8_MEDTR; IPR025476 (Helitron helicase-like domain) |
| Araip.AVY5P | 0.05 | Ulp1 protease family, carboxy-terminal domain protein |
| Araip.B4NZ9 | 0.01 | Protein kinase superfamily protein; IPR001881 (EGF-like calcium-binding domain), IPR011009 (Protein kinase-like domain), IPR013320 (Concanavalin A-like lectin/glucanase, subgroup), IPR025287 (Wall-associated receptor kinase galacturonan-binding domain); GO:0005509 (calcium ion binding), GO:0030247 (polysaccharide binding) |
| Araip.B8ZC0 | 0.02 | Pentatricopeptide repeat (PPR) superfamily protein |
| Araip.C20VH | 0.01 | uncharacterized protein LOC100778822 isoform X6 [Glycine max]; IPR004252 (Probable transposase, Ptta/En/Spm, plant) |
| Araip.CQL95 | 0.01 | Ulp1 protease family, carboxy-terminal domain protein |
| Araip.D3IBN | 0.01 | protein FAR1-RELATED SEQUENCE 5-like [Glycine max]; IPR001878 (Zinc finger, CCHC-type), IPR004330 (FAR1 DNA binding domain), IPR007527 (Zinc finger, SWIM-type); GO:0003676 (nucleic acid binding), GO:0008270 (zinc ion binding) |
| Araip.D5359 | 0.04 | replication protein A 70 kDa DNA-binding subunit C-like [Glycine max]; IPR012340 (Nucleic acid-binding, OB-fold) |
| Araip.D7NWY | 0.01 | protein FAR1-RELATED SEQUENCE 5-like [Glycine max] |
| Araip.DFS0G | 0.02 | protein n=1 Tax=Oryza sativa subsp. japonica RepID=C7J0D1_ORYSJ; IPR001878 (Zinc finger, CCHC-type), IPR007527 (Zinc finger, SWIM-type); GO:0003676 (nucleic acid binding), GO:0008270 (zinc ion binding) |
| Araip.DWZ17 | 0.07 | Unknown protein |
| Araip.E5TT0 | 0.01 | protein FAR1-RELATED SEQUENCE 5-like [Glycine max]; IPR001878 (Zinc finger, CCHC-type), IPR004330 (FAR1 DNA binding domain), IPR006564 (Zinc finger, PMZ-type); GO:0003676 (nucleic acid binding), GO:0008270 (zinc ion binding) |
| Araip.E6Y7U | 0.03 | n=2 Tax=Oryza sativa subsp. japonica RepID=Q7X7Y4_ORYSJ |
| Araip.ET1VS | 0.02 | RNA helicase family protein; IPR010666 (Zinc finger, GRF-type), IPR027417 (P-loop containing nucleoside triphosphate hydrolase); GO:0008270 (zinc ion binding) |
| Araip.F3V4B | 0.03 | Myb/SANT-like DNA-binding domain protein |
| Araip.F6LRT | 0.04 | Unknown protein |
| Araip.FFN83 | 0.03 | Unknown protein |
| Araip.FQJ47 | 0.08 | ABC transporter-related |
| Araip.FXX59 | 0.1 | probable WRKY transcription factor 23-like [Glycine max]; IPR003657 (DNA-binding WRKY); GO:0003700 (sequence-specific DNA binding transcription factor activity), GO:0043565 (sequence-specific DNA binding) |
| Araip.G8K2J | 0.04 | hypothetical protein |
| Araip.GJZ58 | 0.01 | replication protein A 70 kDa DNA-binding subunit C-like [Glycine max]; IPR012340 (Nucleic acid-binding, OB-fold) |
| Araip.GM98D | 0.08 | F-box plant-like protein, putative; IPR027949 (Petal formation-expressed) |
| Araip.GP33L | 0.04 | IST1 homolog isoform X1 [Glycine max] |
| Araip.GP4C6 | 0.28 | 60S ribosomal protein L38-like [Glycine max]; IPR002675 (Ribosomal protein L38e); GO:0003735 (structural constituent of ribosome), GO:0005622 (intracellular), GO:0005840 (ribosome), GO:0006412 (translation) |
| Araip.H8ABA | 0.02 | Unknown protein |
| Araip.HF1ER | 0.07 | receptor kinase 3; IPR011009 (Protein kinase-like domain); GO:0004672 (protein kinase activity), GO:0006468 (protein phosphorylation) |
| Araip.HFK72 | 0.05 | hypothetical protein |
| Araip.HQ20L | 0.04 | gibberellin-regulated family protein |
| Araip.HTN3R | 0.03 | hypothetical protein |
| Araip.I0RMF | 0.07 | uncharacterized protein LOC100781836 [Glycine max] |
| Araip.I2LD4 | 0.07 | Unknown protein |
| Araip.IC29B | 0.01 | Pentatricopeptide repeat (PPR) superfamily protein; IPR002885 (Pentatricopeptide repeat), IPR011990 (Tetratricopeptide-like helical); GO:0005515 (protein binding) |
| Araip.J0E3T | 0.01 | serine/threonine-protein phosphatase 7 long form homolog [Glycine max]; IPR019557 (Aminotransferase-like, plant mobile domain) |
| Araip.J9A9K | 0.03 | receptor-like kinase, putative; IPR001611 (Leucine-rich repeat), IPR003591 (Leucine-rich repeat, typical subtype); GO:0005515 (protein binding) |
| Araip.JE2T8 | 0.03 | Unknown protein |
| Araip.JF4H4 | 0.05 | Unknown protein |
| Araip.JK92J | 0.03 | Unknown protein |
| Araip.JNA2K | 0.04 | serine/threonine-protein phosphatase 7 long form homolog [Glycine max]; IPR019557 (Aminotransferase-like, plant mobile domain) |
| Araip.JP9Q5 | 0.07 | Unknown protein |
| Araip.JW0IJ | 0.03 | Peptidyl-tRNA hydrolase family protein; IPR001328 (Peptidyl-tRNA hydrolase); GO:0004045 (aminoacyl-tRNA hydrolase activity) |
| Araip.JY62W | 0.01 | uncharacterized protein LOC100801905 isoform X5 [Glycine max] |
| Araip.K5F8T | 0.01 | serine/threonine-protein phosphatase 7 long form homolog [Glycine max]; IPR019557 (Aminotransferase-like, plant mobile domain) |
| Araip.K80WT | 0.01 | TdcA1-ORF2 protein n=1 Tax=Medicago truncatula RepID=G7JDI9_MEDTR |
| Araip.K96U9 | 0.03 | protein FAR1-RELATED SEQUENCE 6-like isoform X2 [Glycine max]; IPR007527 (Zinc finger, SWIM-type); GO:0008270 (zinc ion binding) |
| Araip.KDC0M | 0.03 | Rad23 UV excision repair protein family; IPR004806 (UV excision repair protein Rad23), IPR009060 (UBA-like); GO:0005515 (protein binding), GO:0005634 (nucleus), GO:0006289 (nucleotide-excision repair) |
| Araip.KK13V | 0.03 | Unknown protein |
| Araip.KKI42 | 0.04 | Unknown protein; IPR012336 (Thioredoxin-like fold) |
| Araip.KPB1S | 0.1 | PLATZ transcription factor family protein; IPR006734 (Protein of unknown function DUF597) |
| Araip.KQC7G | 0.01 | Pentatricopeptide repeat (PPR) superfamily protein; IPR002885 (Pentatricopeptide repeat) |
| Araip.KR88K | 0.04 | RWP-RK domain-containing protein; IPR003035 (RWP-RK domain) |
| Araip.L5JJM | 0.07 | Unknown protein |
| Araip.LR8SY | 0.02 | Unknown protein |
| Araip.LUC7R | 0.03 | B3 domain transcription factor VRN1-like protein; IPR015300 (DNA-binding pseudobarrel domain) |
| Araip.LW83W | 0.05 | serine carboxypeptidase-like 46; IPR001563 (Peptidase S10, serine carboxypeptidase); GO:0004185 (serine-type carboxypeptidase activity), GO:0006508 (proteolysis) |
| Araip.M052T | 0.04 | Unknown protein |
| Araip.MB7JR | 0.14 | Unknown protein |
| Araip.MB9W4 | 0.01 | Unknown protein; IPR009027 (Ribosomal protein L9/RNase H1, N-terminal) |
| Araip.MG896 | 0.02 | Pyridoxal phosphate (PLP)-dependent transferases superfamily protein n=1 Tax=Theobroma cacao RepID=UPI00042B3A8C; IPR002129 (Pyridoxal phosphate-dependent decarboxylase), IPR015424 (Pyridoxal phosphate-dependent transferase); GO:0003824 (catalytic activity), GO:0016831 (carboxy-lyase activity), GO:0019752 (carboxylic acid metabolic process), GO:0030170 (pyridoxal phosphate binding) |
| Araip.MI0G9 | 0.02 | serine/threonine-protein phosphatase 7 long form homolog [Glycine max]; IPR019557 (Aminotransferase-like, plant mobile domain) |
| Araip.MR96L | 0.01 | uncharacterized protein LOC102660738 isoform X3 [Glycine max] |
| Araip.MT86A | 0.03 | disease resistance protein; IPR002182 (NB-ARC), IPR027417 (P-loop containing nucleoside triphosphate hydrolase); GO:0043531 (ADP binding) |
| Araip.N44JY | 0.04 | Amino acid dehydrogenase family protein; IPR016040 (NAD(P)-binding domain); GO:0006520 (cellular amino acid metabolic process), GO:0016491 (oxidoreductase activity), GO:0055114 (oxidation-reduction process) |
| Araip.NF8W0 | 0.25 | Regulator of chromosome condensation (RCC1) family protein |
| Araip.NHV7V | 0.04 | squalene monooxygenase 2; IPR013698 (Squalene epoxidase); GO:0004506 (squalene monooxygenase activity), GO:0016021 (integral component of membrane), GO:0050660 (flavin adenine dinucleotide binding), GO:0055114 (oxidation-reduction process) |
| Araip.NQD8I | 0.02 | n=1 Tax=Oryza sativa subsp. japonica RepID=Q7XU62_ORYSJ |
| Araip.NQT6T | 0.07 | Unknown protein |
| Araip.P25KR | 0.14 | blue copper protein-like [Glycine max]; IPR008972 (Cupredoxin); GO:0005507 (copper ion binding), GO:0009055 (electron carrier activity) |
| Araip.P5D6Y | 0.02 | protein FAR1-RELATED SEQUENCE 6-like isoform X2 [Glycine max]; IPR007527 (Zinc finger, SWIM-type); GO:0008270 (zinc ion binding) |
| Araip.P9U24 | 0.09 | Unknown protein |
| Araip.PBP93 | 0.03 | serine/threonine-protein phosphatase 7 long form homolog [Glycine max]; IPR019557 (Aminotransferase-like, plant mobile domain) |
| Araip.PIN8N | 0.01 | replication factor-A carboxy-terminal domain protein; IPR012340 (Nucleic acid-binding, OB-fold) |
| Araip.PKK3G | 0.03 | serine/threonine-protein phosphatase 7 long form homolog [Glycine max]; IPR019557 (Aminotransferase-like, plant mobile domain) |
| Araip.Q961N | 0.11 | Unknown protein |
| Araip.R1IG3 | 0.03 | protein FAR1-RELATED SEQUENCE 5-like [Glycine max] |
| Araip.RB35J | 0.01 | serine/threonine-protein phosphatase 7 long form homolog [Glycine max]; IPR019557 (Aminotransferase-like, plant mobile domain) |
| Araip.RC0T2 | 0.1 | B3 domain-containing transcription factor VRN1-like isoform X3 [Glycine max]; IPR015300 (DNA-binding pseudobarrel domain) |
| Araip.RK21R | 0.02 | Regulator of chromosome condensation (RCC1) family protein; IPR009091 (Regulator of chromosome condensation 1/beta-lactamase-inhibitor protein II) |
| Araip.S23A3 | 0.06 | serine/threonine-protein phosphatase 7 long form homolog [Glycine max]; IPR019557 (Aminotransferase-like, plant mobile domain) |
| Araip.S3VDQ | 0.17 | Unknown protein |
| Araip.S6RBI | 0.02 | nudix hydrolase homolog 15 |
| Araip.SB820 | 0.05 | DUF223 domain protein; IPR012340 (Nucleic acid-binding, OB-fold) |
| Araip.SK7LI | 0.04 | Unknown protein |
| Araip.T7L11 | 0.01 | formin 8; IPR015425 (Formin, FH2 domain) |
| Araip.TD549 | 0.06 | Unknown protein |
| Araip.TG72Q | 0.07 | 3-ketoacyl-CoA synthase 4; IPR016039 (Thiolase-like); GO:0003824 (catalytic activity), GO:0008152 (metabolic process) |
| Araip.U09A3 | 0.03 | Unknown protein |
| Araip.U6JBH | 0.03 | Helicase-like protein n=1 Tax=Oryza sativa subsp. japonica RepID=Q5NAA4_ORYSJ; IPR025476 (Helitron helicase-like domain) |
| Araip.U9787 | 0.14 | Unknown protein |
| Araip.UGD52 | 0.01 | Unknown protein |
| Araip.UH81C | 0.05 | Unknown protein |
| Araip.UKS4W | 0.03 | Aminotransferase-like, plant mobile domain family protein |
| Araip.V5T1R | 0.07 | Unknown protein |
| Araip.V8YPW | 0.01 | phosphatidylinositol 3,4,5-trisphosphate 3-phosphatase and dual-specificity protein phosphatase PTEN-like isoform X2 [Glycine max] |
| Araip.VB5WD | 0.01 | FAD-binding Berberine family protein; IPR012951 (Berberine/berberine-like), IPR016166 (FAD-binding, type 2); GO:0003824 (catalytic activity), GO:0008762 (UDP-N-acetylmuramate dehydrogenase activity), GO:0016491 (oxidoreductase activity), GO:0050660 (flavin adenine dinucleotide binding), GO:0055114 (oxidation-reduction process) |
| Araip.VL1SL | 0.04 | Unknown protein |
| Araip.VS0AP | 0.01 | uncharacterized protein LOC100797259 isoform X3 [Glycine max]; IPR004332 (Transposase, MuDR, plant), IPR008160 (Collagen triple helix repeat) |
| Araip.W1HEM | 0.06 | caffeic acid O-methyltransferase; IPR011991 (Winged helix-turn-helix DNA-binding domain) |
| Araip.W3KGE | 0.04 | Regulator of chromosome condensation (RCC1) family protein; IPR009091 (Regulator of chromosome condensation 1/beta-lactamase-inhibitor protein II) |
| Araip.W6XFD | 0.03 | Pentatricopeptide repeat (PPR-like) superfamily protein; IPR002885 (Pentatricopeptide repeat) |
| Araip.W817P | 0.01 | cycloartenol synthase 1; IPR008930 (Terpenoid cyclases/protein prenyltransferase alpha-alpha toroid) |
| Araip.WT37P | 0.04 | GTP binding |
| Araip.WUR16 | 0.07 | Myosin heavy chain-related protein |
| Araip.WWE9M | 0.07 | nucleoside diphosphate kinase 3; IPR001564 (Nucleoside diphosphate kinase); GO:0004550 (nucleoside diphosphate kinase activity), GO:0005524 (ATP binding), GO:0006165 (nucleoside diphosphate phosphorylation), GO:0006183 (GTP biosynthetic process), GO:0006228 (UTP biosynthetic process), GO:0006241 (CTP biosynthetic process) |
| Araip.WZW9W | 0.08 | deoxynucleoside triphosphate triphosphohydrolase SAMHD1 homolog isoform X2 [Glycine max] |
| Araip.X0M31 | 0.01 | organic cation/carnitine transporter 2; IPR005828 (General substrate transporter), IPR016196 (Major facilitator superfamily domain, general substrate transporter); GO:0016021 (integral component of membrane), GO:0022857 (transmembrane transporter activity), GO:0055085 (transmembrane transport) |
| Araip.X1AHC | 0.01 | serine/threonine-protein phosphatase 7 long form homolog [Glycine max]; IPR019557 (Aminotransferase-like, plant mobile domain) |
| Araip.X1NZ4 | 0.1 | transcription factor GAMYB-like [Glycine max] |
| Araip.XC6R9 | 0.01 | Myb/SANT-like DNA-binding domain protein |
| Araip.XFX9Y | 0.03 | animal RPA1 domain protein; IPR012340 (Nucleic acid-binding, OB-fold) |
| Araip.XT73T | 0.12 | cell wall / vacuolar inhibitor of fructosidase 1-like [Glycine max]; IPR006501 (Pectinesterase inhibitor domain); GO:0004857 (enzyme inhibitor activity), GO:0030599 (pectinesterase activity) |
| Araip.Y3R0X | 0.01 | uncharacterized protein At2g29880-like [Glycine max]; IPR024752 (Myb/SANT-like domain), IPR026103 (Harbinger transposase-derived nuclease, animal) |
| Araip.YN7KH | 0.03 | 2-hydroxyacyl-CoA lyase-like [Glycine max] |
| Araip.Z7XVL | 0.03 | F-box/LRR-repeat protein 13-like isoform X3 [Glycine max] |
| Araip.ZEQ62 | 0.02 | Unknown protein |
| Araip.ZN6UK | 0.65 | mediator of RNA polymerase II transcription subunit 25-like isoform X6 [Glycine max]; IPR021419 (Mediator complex, subunit Med25, von Willebrand factor type A) |
| Araip.ZWQ3R | 0.1 | Unknown protein |
| Pattee 6 pericarp | |  |
| Aradu.03JW6 | 0.01 | zinc finger MYM-type protein 1-like [Glycine max]; IPR008906 (HAT dimerisation domain, C-terminal), IPR012337 (Ribonuclease H-like domain); GO:0003676 (nucleic acid binding), GO:0046983 (protein dimerization activity) |
| Aradu.2J2S8 | 0.01 | RNA-binding protein Luc7-like 2, putative, expressed n=6 Tax=Oryza RepID=Q10AS2_ORYSJ; IPR004882 (Luc7-related); GO:0003729 (mRNA binding), GO:0005685 (U1 snRNP), GO:0006376 (mRNA splice site selection) |
| Aradu.3VW4U | 0.01 | protein FAR1-RELATED SEQUENCE 3-like isoform X1 [Glycine max]; IPR001878 (Zinc finger, CCHC-type), IPR004330 (FAR1 DNA binding domain), IPR006564 (Zinc finger, PMZ-type); GO:0003676 (nucleic acid binding), GO:0008270 (zinc ion binding) |
| Aradu.7CS2D | 0.03 | Ran guanine nucleotide release factor-like protein; IPR007681 (Ran-interacting Mog1 protein) |
| Aradu.88C37 | 0.16 | Unknown protein |
| Aradu.AD5J0 | 0.02 | Unknown protein |
| Aradu.B2TV7 | 0.05 | Unknown protein |
| Aradu.BVE4Y | 0.01 | serine/threonine-protein phosphatase 7 long form homolog [Glycine max]; IPR019557 (Aminotransferase-like, plant mobile domain) |
| Aradu.CP89C | 0.01 | tubulin alpha-2 chain-like [Glycine max] |
| Aradu.CV6Z5 | 0.03 | E3 ubiquitin-protein ligase n=5 Tax=Solanum RepID=M1BDZ1_SOLTU |
| Aradu.DGS8E | 0.03 | Unknown protein |
| Aradu.IRF9C | 0.01 | Ulp1 protease family, carboxy-terminal domain protein |
| Aradu.JYS2D | 0.02 | Unknown protein |
| Aradu.NK858 | 0.05 | Unknown protein |
| Aradu.QWS86 | 0.02 | 1-deoxy-D-xylulose 5-phosphate synthase 1; IPR005475 (Transketolase-like, pyrimidine-binding domain), IPR005476 (Transketolase, C-terminal), IPR009014 (Transketolase, C-terminal/Pyruvate-ferredoxin oxidoreductase, domain II); GO:0003824 (catalytic activity), GO:0008152 (metabolic process) |
| Aradu.TU6IS | 0.05 | uncharacterized protein LOC100797259 isoform X4 [Glycine max]; IPR007527 (Zinc finger, SWIM-type); GO:0008270 (zinc ion binding) |
| Aradu.XAQ5U | 0.01 | protein FAR1-RELATED SEQUENCE 6-like isoform X2 [Glycine max]; IPR004330 (FAR1 DNA binding domain) |
| Araip.0GZ9E | 0.06 | F-box/RNI-like superfamily protein; IPR001810 (F-box domain); GO:0005515 (protein binding) |
| Araip.1A8AW | 0.01 | coenzyme Q-binding protein COQ10 homolog B, mitochondrial isoform X1 [Glycine max]; IPR023393 (START-like domain) |
| Araip.31H00 | 0.04 | Endoplasmic reticulum vesicle transporter protein; IPR005050 (Early nodulin 93 ENOD93 protein) |
| Araip.40KW4 | 0.05 | zinc finger MYM-type protein 1-like [Glycine max]; IPR025398 (Domain of unknown function DUF4371) |
| Araip.47JE2 | 0.03 | Unknown protein |
| Araip.55ZLZ | 0.01 | Unknown protein |
| Araip.5I6LE | 0.02 | Unknown protein |
| Araip.5PB4V | 0.04 | peptide transporter 2; IPR000109 (Proton-dependent oligopeptide transporter family); GO:0005215 (transporter activity), GO:0006810 (transport), GO:0016020 (membrane) |
| Araip.5XQ3P | 0.02 | Unknown protein |
| Araip.6M41J | 0.11 | Mitovirus RNA-dependent RNA polymerase; IPR008686 (RNA-dependent RNA polymerase, mitoviral) |
| Araip.79F04 | 0.01 | 40s ribosomal protein SA; IPR005707 (Ribosomal protein S2, eukaryotic/archaeal), IPR023591 (Ribosomal protein S2, flavodoxin-like domain); GO:0003735 (structural constituent of ribosome), GO:0006412 (translation), GO:0015935 (small ribosomal subunit) |
| Araip.8N8PB | 0.07 | Unknown protein |
| Araip.95PC4 | 0.02 | Unknown protein |
| Araip.AHX9S | 0.02 | Ulp1 protease family, carboxy-terminal domain protein; IPR003653 (Peptidase C48, SUMO/Sentrin/Ubl1); GO:0006508 (proteolysis), GO:0008234 (cysteine-type peptidase activity) |
| Araip.BKV85 | 0.09 | serine/threonine-protein phosphatase 7 long form homolog [Glycine max]; IPR019557 (Aminotransferase-like, plant mobile domain) |
| Araip.H3L59 | 0.01 | Aminotransferase-like, plant mobile domain family protein; IPR019557 (Aminotransferase-like, plant mobile domain) |
| Araip.HAM0F | 0.02 | serine/threonine-protein phosphatase 7 long form homolog [Glycine max]; IPR019557 (Aminotransferase-like, plant mobile domain) |
| Araip.IZ7X4 | 0.02 | uncharacterized protein LOC100800865 isoform X3 [Glycine max] |
| Araip.JDY2N | 0.1 | serine/threonine-protein phosphatase 7 long form homolog [Glycine max]; IPR019557 (Aminotransferase-like, plant mobile domain) |
| Araip.QGL9N | 0.06 | Unknown protein |
| Araip.R7U9P | 0.01 | Zinc knuckle family protein n=1 Tax=Oryza sativa subsp. japonica RepID=H2KW38_ORYSJ |
| Araip.U5JVD | 0.03 | zinc finger MYM-type protein 1-like [Glycine max]; IPR008906 (HAT dimerisation domain, C-terminal); GO:0046983 (protein dimerization activity) |
| Araip.WV5H0 | 0.03 | Unknown protein |
| Araip.XF945 | 0.12 | F-box/LRR-repeat protein 13-like isoform X3 [Glycine max] |
| Araip.XS1F4 | 0.01 | protein FAR1-RELATED SEQUENCE 5-like [Glycine max]; IPR004330 (FAR1 DNA binding domain) |
| Araip.ZBM6D | 0.05 | zinc finger MYM-type protein 1-like [Glycine max]; IPR025398 (Domain of unknown function DUF4371) |
| Pattee 6 seed | |  |
| Aradu.04MMS | 0.03 | Unknown protein |
| Aradu.0JJ3S | 0.04 | hypothetical protein |
| Aradu.18BLS | 0.01 | isocitrate dehydrogenase; IPR004790 (Isocitrate dehydrogenase NADP-dependent), IPR018786 (Protein of unknown function DUF2343), IPR024084 (Isopropylmalate dehydrogenase-like domain); GO:0004450 (isocitrate dehydrogenase (NADP+) activity), GO:0006102 (isocitrate metabolic process), GO:0055114 (oxidation-reduction process) |
| Aradu.18PBT | 0.08 | GRF zinc finger protein; IPR010666 (Zinc finger, GRF-type); GO:0008270 (zinc ion binding) |
| Aradu.1E8XD | 0.04 | animal RPA1 domain protein; IPR012340 (Nucleic acid-binding, OB-fold) |
| Aradu.1E9A5 | 0.04 | Unknown protein |
| Aradu.1R26G | 0.03 | NAD(P)H-quinone oxidoreductase subunit H; IPR001450 (4Fe-4S binding domain), IPR001694 (NADH:ubiquinone oxidoreductase, subunit 1/F420H2 oxidoreductase subunit H); GO:0016020 (membrane), GO:0051536 (iron-sulfur cluster binding), GO:0055114 (oxidation-reduction process) |
| Aradu.25BVS | 0.02 | serine/threonine-protein phosphatase 7 long form homolog [Glycine max]; IPR019557 (Aminotransferase-like, plant mobile domain) |
| Aradu.3L851 | 0.07 | Unknown protein |
| Aradu.44B7T | 0.03 | uncharacterized protein LOC102669519 [Glycine max]; IPR006564 (Zinc finger, PMZ-type); GO:0008270 (zinc ion binding) |
| Aradu.45LF4 | 0.01 | exostosin family protein |
| Aradu.46UHL | 0.15 | NAD(P)-linked oxidoreductase superfamily protein; IPR023210 (NADP-dependent oxidoreductase domain) |
| Aradu.4K9XW | 0.01 | uncharacterized protein LOC100793937 [Glycine max]; IPR025312 (Domain of unknown function DUF4216) |
| Aradu.511YW | 0.01 | Polyprotein n=1 Tax=Citrus endogenous pararetrovirus RepID=V9QEM3_9RETR |
| Aradu.54TMM | 0.01 | dihydroflavonol 4-reductase; IPR001509 (NAD-dependent epimerase/dehydratase), IPR016040 (NAD(P)-binding domain); GO:0003824 (catalytic activity), GO:0044237 (cellular metabolic process), GO:0050662 (coenzyme binding) |
| Aradu.5ZE0V | 0.06 | Unknown protein |
| Aradu.6MT8W | 0.1 | replication factor-A carboxy-terminal domain protein; IPR012340 (Nucleic acid-binding, OB-fold) |
| Aradu.6R588 | 0.03 | Unknown protein |
| Aradu.7AR36 | 0.01 | uncharacterized protein LOC100778822 isoform X6 [Glycine max]; IPR004252 (Probable transposase, Ptta/En/Spm, plant) |
| Aradu.88GDF | 0.03 | zinc finger MYM-type protein 1-like [Glycine max]; IPR012337 (Ribonuclease H-like domain), IPR025398 (Domain of unknown function DUF4371); GO:0003676 (nucleic acid binding) |
| Aradu.88P13 | 0.04 | serine/threonine-protein phosphatase 7 long form homolog [Glycine max]; IPR019557 (Aminotransferase-like, plant mobile domain) |
| Aradu.8G2XG | 0.05 | Unknown protein |
| Aradu.91S78 | 0.01 | protein FAR1-RELATED SEQUENCE 6-like isoform X2 [Glycine max]; IPR004330 (FAR1 DNA binding domain), IPR007527 (Zinc finger, SWIM-type); GO:0008270 (zinc ion binding) |
| Aradu.92ZTB | 0.03 | Unknown protein |
| Aradu.930Q2 | 0.01 | serine/threonine-protein phosphatase 7 long form homolog [Glycine max]; IPR019557 (Aminotransferase-like, plant mobile domain) |
| Aradu.943HH | 0.07 | Unknown protein |
| Aradu.9JI68 | 0.02 | Unknown protein |
| Aradu.A3D6P | 0.02 | Unknown protein |
| Aradu.AF4E5 | 0.07 | Unknown protein |
| Aradu.AK2Z5 | 0.01 | Zinc knuckle family protein n=1 Tax=Oryza sativa subsp. japonica RepID=H2KW38_ORYSJ |
| Aradu.AKB61 | 0.05 | serine hydroxymethyltransferase 2; IPR001085 (Serine hydroxymethyltransferase), IPR015424 (Pyridoxal phosphate-dependent transferase); GO:0003824 (catalytic activity), GO:0004372 (glycine hydroxymethyltransferase activity), GO:0006544 (glycine metabolic process), GO:0006563 (L-serine metabolic process), GO:0030170 (pyridoxal phosphate binding) |
| Aradu.AT4F1 | 0.05 | Unknown protein |
| Aradu.AYR8X | 0.01 | replication factor-A carboxy-terminal domain protein |
| Aradu.B039Q | 0.01 | serine/threonine-protein phosphatase 7 long form homolog [Glycine max]; IPR019557 (Aminotransferase-like, plant mobile domain) |
| Aradu.B2WAQ | 0.01 | uncharacterized protein LOC100776940 isoform X4 [Glycine max]; IPR004332 (Transposase, MuDR, plant), IPR007527 (Zinc finger, SWIM-type); GO:0008270 (zinc ion binding) |
| Aradu.B3TRC | 0.02 | Ulp1 protease family, carboxy-terminal domain protein; IPR003653 (Peptidase C48, SUMO/Sentrin/Ubl1); GO:0006508 (proteolysis), GO:0008234 (cysteine-type peptidase activity) |
| Aradu.D7XHL | 0.01 | peroxidase 47 [Glycine max]; IPR010255 (Haem peroxidase); GO:0004601 (peroxidase activity), GO:0006979 (response to oxidative stress), GO:0020037 (heme binding), GO:0055114 (oxidation-reduction process) |
| Aradu.D9NSE | 0.01 | serine/threonine-protein phosphatase 7 long form homolog [Glycine max]; IPR019557 (Aminotransferase-like, plant mobile domain) |
| Aradu.DF6G2 | 0.02 | Zinc knuckle family protein n=1 Tax=Oryza sativa subsp. japonica RepID=H2KWL4_ORYSJ |
| Aradu.DYF52 | 0.01 | replication factor-A carboxy-terminal domain protein; IPR012340 (Nucleic acid-binding, OB-fold) |
| Aradu.EJ9ZS | 0.01 | FAR-RED impaired response-like protein, putative; IPR001878 (Zinc finger, CCHC-type); GO:0003676 (nucleic acid binding), GO:0008270 (zinc ion binding) |
| Aradu.EK988 | 0.01 | serine/threonine-protein phosphatase 7 long form homolog [Glycine max]; IPR019557 (Aminotransferase-like, plant mobile domain) |
| Aradu.EQ7W8 | 0.07 | Unknown protein |
| Aradu.EV16F | 0.01 | replication protein A 1A; IPR012340 (Nucleic acid-binding, OB-fold) |
| Aradu.F3U3G | 0.05 | Unknown protein |
| Aradu.FN7TZ | 0.04 | DUF223 domain protein; IPR012340 (Nucleic acid-binding, OB-fold) |
| Aradu.G73UV | 0.03 | Unknown protein; IPR004252 (Probable transposase, Ptta/En/Spm, plant) |
| Aradu.GFM22 | 0.01 | zinc finger MYM-type protein 1-like [Glycine max]; IPR006580 (Zinc finger, TTF-type), IPR008906 (HAT dimerisation domain, C-terminal), IPR025398 (Domain of unknown function DUF4371); GO:0046983 (protein dimerization activity) |
| Aradu.H0VWB | 0.03 | Unknown protein |
| Aradu.I6C6R | 0.04 | Unknown protein |
| Aradu.IZK56 | 0.03 | UDP-Glycosyltransferase superfamily protein |
| Aradu.K270P | 0.03 | MORC family CW-type zinc finger protein 3-like [Glycine max]; IPR003594 (Histidine kinase-like ATPase, ATP-binding domain); GO:0005524 (ATP binding) |
| Aradu.K7STL | 0.02 | Ulp1 protease family, carboxy-terminal domain protein |
| Aradu.K8R4G | 0.01 | Unknown protein |
| Aradu.M1GLN | 0.04 | GRF zinc finger protein; IPR010666 (Zinc finger, GRF-type); GO:0008270 (zinc ion binding) |
| Aradu.MU0MV | 0.01 | Ulp1 protease family, carboxy-terminal domain protein; IPR003653 (Peptidase C48, SUMO/Sentrin/Ubl1); GO:0006508 (proteolysis), GO:0008234 (cysteine-type peptidase activity) |
| Aradu.N6X0V | 0.06 | receptor-like protein kinase-like [Glycine max] |
| Aradu.NM0P3 | 0.07 | Unknown protein |
| Aradu.P12FR | 0.19 | Unknown protein |
| Aradu.PSN4Y | 0.04 | Unknown protein |
| Aradu.QH2NX | 0.01 | protein FAR1-RELATED SEQUENCE 5-like [Glycine max]; IPR004330 (FAR1 DNA binding domain), IPR007527 (Zinc finger, SWIM-type); GO:0008270 (zinc ion binding) |
| Aradu.QR7J5 | 0.03 | Unknown protein |
| Aradu.QY135 | 0.05 | Unknown protein |
| Aradu.R3MAC | 0.01 | zinc finger MYM-type protein 1-like [Glycine max]; IPR025398 (Domain of unknown function DUF4371) |
| Aradu.RA5V5 | 0.03 | Unknown protein |
| Aradu.RJG6Q | 0.01 | uncharacterized mitochondrial protein AtMg00860-like [Glycine max] |
| Aradu.SBS3H | 0.02 | serine/threonine-protein phosphatase 7 long form homolog [Glycine max]; IPR019557 (Aminotransferase-like, plant mobile domain) |
| Aradu.SCW7L | 0.01 | serine/threonine-protein phosphatase 7 long form homolog [Glycine max]; IPR019557 (Aminotransferase-like, plant mobile domain) |
| Aradu.TBR6D | 0.04 | Unknown protein |
| Aradu.TRQ75 | 0.01 | Unknown protein |
| Aradu.TT25S | 0.03 | isoflavone reductase-like protein-like [Glycine max]; IPR008030 (NmrA-like) |
| Aradu.UC9WV | 0.04 | Unknown protein |
| Aradu.VMR7Q | 0.01 | Unknown protein |
| Aradu.WQ8WR | 0.09 | Unknown protein |
| Aradu.X9Q7D | 0.01 | replication factor-A carboxy-terminal domain protein; IPR012340 (Nucleic acid-binding, OB-fold) |
| Aradu.XZB4T | 0.1 | replication factor-A carboxy-terminal domain protein; IPR012340 (Nucleic acid-binding, OB-fold) |
| Aradu.Y2TPC | 0.1 | Transposon protein n=1 Tax=Arachis hypogaea RepID=G0Y6V7_ARAHY |
| Aradu.Y4NA5 | 0.03 | transcription factor bHLH25-like [Glycine max] |
| Aradu.YL0G7 | 0.01 | serine/threonine-protein phosphatase 7 long form homolog [Glycine max]; IPR019557 (Aminotransferase-like, plant mobile domain) |
| Aradu.YY1SC | 0.02 | GRF zinc finger protein |
| Aradu.YZW9J | 0.03 | Unknown protein |
| Aradu.ZM7WC | 0.04 | uncharacterized protein LOC100800997 isoform X1 [Glycine max] |
| Araip.00PHC | 0.01 | n=1 Tax=Oryza sativa subsp. japonica RepID=Q7F971_ORYSJ; IPR007527 (Zinc finger, SWIM-type); GO:0008270 (zinc ion binding) |
| Araip.0E204 | 0.04 | Unknown protein |
| Araip.0IT7K | 0.04 | serine/threonine-protein phosphatase 7 long form homolog [Glycine max]; IPR019557 (Aminotransferase-like, plant mobile domain) |
| Araip.0N9JC | 0.04 | F-box protein interaction domain protein; IPR001810 (F-box domain), IPR017451 (F-box associated interaction domain); GO:0005515 (protein binding) |
| Araip.0Q2AU | 0.03 | homeobox-leucine zipper protein ANTHOCYANINLESS 2-like isoform X1 [Glycine max] |
| Araip.0X4NL | 0.04 | Unknown protein |
| Araip.11U57 | 0.04 | sulfate transporter 1; 2; IPR011547 (Sulphate transporter); GO:0008272 (sulfate transport), GO:0015116 (sulfate transmembrane transporter activity), GO:0016021 (integral component of membrane) |
| Araip.141LD | 0.06 | phosphoenolpyruvate carboxylase 2 |
| Araip.16K5I | 0.01 | uncharacterized protein LOC100800865 isoform X3 [Glycine max]; IPR004332 (Transposase, MuDR, plant) |
| Araip.184L3 | 0.01 | serine/threonine-protein phosphatase 7 long form homolog [Glycine max]; IPR019557 (Aminotransferase-like, plant mobile domain) |
| Araip.18DX7 | 0.07 | glutathione S-transferase F3; IPR012336 (Thioredoxin-like fold); GO:0005515 (protein binding) |
| Araip.1K8QB | 0.01 | Unknown protein; IPR009027 (Ribosomal protein L9/RNase H1, N-terminal) |
| Araip.1T0P0 | 0.01 | protein FAR1-RELATED SEQUENCE 6-like isoform X2 [Glycine max]; IPR007527 (Zinc finger, SWIM-type); GO:0008270 (zinc ion binding) |
| Araip.208KG | 0.01 | ABC transporter C family member 14-like isoform X5 [Glycine max] |
| Araip.21EHL | 0.03 | Unknown protein |
| Araip.253VM | 0.04 | Ulp1 protease family, carboxy-terminal domain protein |
| Araip.2ND4Q | 0.01 | Unknown protein; IPR001878 (Zinc finger, CCHC-type); GO:0003676 (nucleic acid binding), GO:0008270 (zinc ion binding) |
| Araip.38LZ0 | 0.06 | Unknown protein |
| Araip.39GFX | 0.04 | isochorismatase hydrolase family protein; IPR000868 (Isochorismatase-like); GO:0003824 (catalytic activity), GO:0008152 (metabolic process) |
| Araip.402GB | 0.01 | Unknown protein |
| Araip.4277N | 0.12 | alpha/beta-Hydrolases superfamily protein |
| Araip.43UTQ | 0.01 | serine/threonine-protein phosphatase 7 long form homolog [Glycine max]; IPR019557 (Aminotransferase-like, plant mobile domain) |
| Araip.48VJL | 0.01 | uncharacterized protein LOC100793937 [Glycine max]; IPR025312 (Domain of unknown function DUF4216) |
| Araip.49KMP | 0.1 | Unknown protein |
| Araip.4E4KL | 0.02 | serine/threonine-protein phosphatase 7 long form homolog [Glycine max]; IPR019557 (Aminotransferase-like, plant mobile domain) |
| Araip.4E806 | 0.03 | Unknown protein |
| Araip.4F106 | 0.06 | TatD related DNase |
| Araip.4S4HU | 0.04 | Unknown protein |
| Araip.4YG6Z | 0.04 | cinnamyl alcohol dehydrogenase 6; IPR002085 (Alcohol dehydrogenase superfamily, zinc-type), IPR013149 (Alcohol dehydrogenase, C-terminal), IPR016040 (NAD(P)-binding domain); GO:0008270 (zinc ion binding), GO:0016491 (oxidoreductase activity), GO:0055114 (oxidation-reduction process) |
| Araip.50HAM | 0.01 | 2-aminoethanethiol dioxygenase-like [Glycine max]; IPR012864 (Cysteamine dioxygenase); GO:0047800 (cysteamine dioxygenase activity), GO:0055114 (oxidation-reduction process) |
| Araip.594LP | 0.01 | serine/threonine-protein phosphatase 7 long form homolog [Glycine max]; IPR019557 (Aminotransferase-like, plant mobile domain) |
| Araip.5R41R | 0.03 | regulatory protein NPR1; IPR021094 (NPR1/NIM1-like, C-terminal) |
| Araip.5TM9A | 0.01 | serine/threonine-protein phosphatase 7 long form homolog [Glycine max]; IPR019557 (Aminotransferase-like, plant mobile domain) |
| Araip.629T4 | 0.04 | Unknown protein |
| Araip.69YPH | 0.01 | PfpI family intracellular protease n=3 Tax=Desulfovibrio RepID=D9YED7_9DELT; IPR002818 (ThiJ/PfpI) |
| Araip.6Q6V5 | 0.03 | Cysteine proteinases superfamily protein; IPR013128 (Peptidase C1A), IPR025660 (Cysteine peptidase, histidine active site), IPR025661 (Cysteine peptidase, asparagine active site); GO:0006508 (proteolysis), GO:0008234 (cysteine-type peptidase activity) |
| Araip.6QJ4E | 0.03 | sucrose transporter 4; IPR016196 (Major facilitator superfamily domain, general substrate transporter) |
| Araip.74LBQ | 0.03 | protein n=1 Tax=Oryza sativa subsp. japonica RepID=C7J340_ORYSJ |
| Araip.78IGG | 0.04 | Helicase-like protein n=1 Tax=Medicago truncatula RepID=G7JN81_MEDTR; IPR025476 (Helitron helicase-like domain) |
| Araip.797BH | 0.01 | n=2 Tax=Oryza sativa subsp. japonica RepID=Q7XS42_ORYSJ; IPR003653 (Peptidase C48, SUMO/Sentrin/Ubl1); GO:0006508 (proteolysis), GO:0008234 (cysteine-type peptidase activity) |
| Araip.7K2XW | 0.07 | GRF zinc finger protein; IPR010666 (Zinc finger, GRF-type); GO:0008270 (zinc ion binding) |
| Araip.96X4S | 0.04 | Unknown protein; IPR003653 (Peptidase C48, SUMO/Sentrin/Ubl1); GO:0006508 (proteolysis), GO:0008234 (cysteine-type peptidase activity) |
| Araip.9A8CM | 0.07 | ATPase, F0/V0 complex, subunit C protein; IPR002379 (V-ATPase proteolipid subunit C-like domain); GO:0015078 (hydrogen ion transmembrane transporter activity), GO:0015991 (ATP hydrolysis coupled proton transport) |
| Araip.9NZ8C | 0.02 | n=1 Tax=Oryza sativa subsp. japonica RepID=Q7F971_ORYSJ; IPR001878 (Zinc finger, CCHC-type), IPR007527 (Zinc finger, SWIM-type); GO:0003676 (nucleic acid binding), GO:0008270 (zinc ion binding) |
| Araip.9S769 | 0.01 | protein FAR1-RELATED SEQUENCE 6-like isoform X2 [Glycine max]; IPR007527 (Zinc finger, SWIM-type); GO:0008270 (zinc ion binding) |
| Araip.A4R5T | 0.05 | ycf68 protein |
| Araip.A6KBC | 0.05 | Unknown protein |
| Araip.AEH38 | 0.03 | receptor-like serine/threonine kinase 2; IPR001611 (Leucine-rich repeat), IPR011009 (Protein kinase-like domain); GO:0004672 (protein kinase activity), GO:0004674 (protein serine/threonine kinase activity), GO:0005515 (protein binding), GO:0005524 (ATP binding), GO:0006468 (protein phosphorylation) |
| Araip.ALZ80 | 0.06 | homeodomain transcriptional regulator |
| Araip.ANF1Q | 0.05 | serine/threonine-protein kinase ATM-like [Glycine max] |
| Araip.AQP37 | 0.01 | Unknown protein |
| Araip.B17D6 | 0.04 | Unknown protein |
| Araip.B88VS | 0.07 | Unknown protein |
| Araip.BC5UQ | 0.04 | Helicase-like protein n=1 Tax=Medicago truncatula RepID=G7JN81_MEDTR; IPR025476 (Helitron helicase-like domain) |
| Araip.C5TBJ | 0.04 | shaggy-related kinase 11; IPR011009 (Protein kinase-like domain); GO:0004672 (protein kinase activity), GO:0005524 (ATP binding), GO:0006468 (protein phosphorylation) |
| Araip.C6FKE | 0.01 | serine/threonine-protein phosphatase 7 long form homolog [Glycine max]; IPR019557 (Aminotransferase-like, plant mobile domain) |
| Araip.CB5U0 | 0.09 | uncharacterized protein LOC100798581 [Glycine max] |
| Araip.CG626 | 0.05 | Pentatricopeptide repeat (PPR-like) superfamily protein; IPR002885 (Pentatricopeptide repeat), IPR011990 (Tetratricopeptide-like helical); GO:0005515 (protein binding) |
| Araip.CI52L | 0.09 | Unknown protein |
| Araip.D99JE | 0.02 | serine/threonine-protein phosphatase 7 long form homolog [Glycine max]; IPR019557 (Aminotransferase-like, plant mobile domain) |
| Araip.D9S6G | 0.04 | Unknown protein |
| Araip.DG1MW | 0.01 | Ulp1 protease family, carboxy-terminal domain protein; IPR003653 (Peptidase C48, SUMO/Sentrin/Ubl1); GO:0006508 (proteolysis), GO:0008234 (cysteine-type peptidase activity) |
| Araip.DH2D0 | 0.02 | probable calcium-binding protein CML20 [Glycine max]; IPR011992 (EF-hand domain pair); GO:0005509 (calcium ion binding) |
| Araip.E3USN | 0.03 | Transposon protein n=1 Tax=Arachis hypogaea RepID=G0Y6V7_ARAHY |
| Araip.E6LFC | 0.04 | Unknown protein |
| Araip.F7SDX | 0.09 | replication factor-A carboxy-terminal domain protein; IPR012340 (Nucleic acid-binding, OB-fold) |
| Araip.FF4DS | 0.04 | potassium transporter 2; IPR003855 (K+ potassium transporter); GO:0015079 (potassium ion transmembrane transporter activity), GO:0016020 (membrane), GO:0071805 (potassium ion transmembrane transport) |
| Araip.G51E7 | 0.05 | Unknown protein |
| Araip.G5QQ3 | 0.04 | FKBP-type peptidyl-prolyl cis-trans isomerase family protein; IPR023566 (Peptidyl-prolyl cis-trans isomerase, FKBP-type) |
| Araip.G83AU | 0.04 | Unknown protein |
| Araip.GI9RY | 0.04 | uncharacterized protein LOC100776940 isoform X2 [Glycine max] |
| Araip.GLG2U | 0.01 | serine/threonine-protein phosphatase 7 long form homolog [Glycine max]; IPR019557 (Aminotransferase-like, plant mobile domain) |
| Araip.GTG8K | 0.04 | GRF zinc finger protein; IPR010666 (Zinc finger, GRF-type); GO:0008270 (zinc ion binding) |
| Araip.H3LGE | 0.01 | replication factor-A carboxy-terminal domain protein; IPR012340 (Nucleic acid-binding, OB-fold) |
| Araip.HD2KS | 0.02 | n=1 Tax=Oryza sativa subsp. japonica RepID=Q7XSJ2_ORYSJ; IPR019557 (Aminotransferase-like, plant mobile domain) |
| Araip.HL3F8 | 0.03 | hypothetical protein |
| Araip.HYB44 | 0.03 | 1-acyl-sn-glycerol-3-phosphate acyltransferase 2-like [Glycine max] |
| Araip.I2817 | 0.02 | receptor lectin kinase; IPR008985 (Concanavalin A-like lectin/glucanases superfamily), IPR013320 (Concanavalin A-like lectin/glucanase, subgroup), IPR016363 (Lectin); GO:0030246 (carbohydrate binding) |
| Araip.I5EYD | 0.01 | Ulp1 protease family, carboxy-terminal domain protein; IPR003653 (Peptidase C48, SUMO/Sentrin/Ubl1); GO:0006508 (proteolysis), GO:0008234 (cysteine-type peptidase activity) |
| Araip.I9S33 | 0.01 | Unknown protein |
| Araip.IK4QZ | 0.14 | Unknown protein |
| Araip.J8TP1 | 0.03 | Tetraspanin family protein |
| Araip.JQK1V | 0.04 | 2-dehydro-3-deoxyphosphooctonate aldolase; IPR006269 (3-deoxy-8-phosphooctulonate synthase), IPR013785 (Aldolase-type TIM barrel); GO:0003824 (catalytic activity), GO:0005737 (cytoplasm), GO:0008152 (metabolic process), GO:0008676 (3-deoxy-8-phosphooctulonate synthase activity), GO:0009058 (biosynthetic process) |
| Araip.JQP2I | 0.03 | glucomannan 4-beta-mannosyltransferase 2-like [Glycine max] |
| Araip.JRY9B | 0.01 | Unknown protein |
| Araip.JUT38 | 0.1 | Unknown protein |
| Araip.K3LML | 0.03 | Unknown protein |
| Araip.L8ZUN | 0.06 | Eukaryotic aspartyl protease family protein; IPR021109 (Aspartic peptidase domain) |
| Araip.L9JMI | 0.06 | protein COBRA-like [Glycine max] |
| Araip.LL0K9 | 0.02 | replication factor-A carboxy-terminal domain protein |
| Araip.LZZ87 | 0.02 | protein MOR1-like isoform X2 [Glycine max]; IPR016024 (Armadillo-type fold); GO:0005488 (binding) |
| Araip.M5Y2Z | 0.01 | Unknown protein |
| Araip.MA0LF | 0.4 | protein DEK-like [Glycine max] |
| Araip.NEJ0R | 0.01 | multiple inositol polyphosphate phosphatase PhyIIa protein |
| Araip.NGE1E | 0.04 | Unknown protein |
| Araip.NU3SS | 0.01 | arogenate dehydrogenase; IPR016040 (NAD(P)-binding domain) |
| Araip.P0SZ5 | 0.01 | protein FAR1-RELATED SEQUENCE 6-like isoform X2 [Glycine max]; IPR004330 (FAR1 DNA binding domain), IPR006564 (Zinc finger, PMZ-type); GO:0008270 (zinc ion binding) |
| Araip.PV815 | 0.04 | NBS-LRR type disease resistance protein |
| Araip.Q6WTW | 0.02 | Unknown protein |
| Araip.QR7NH | 0.03 | replication factor-A carboxy-terminal domain protein |
| Araip.QUZ4L | 0.03 | Unknown protein |
| Araip.QZP1C | 0.07 | Unknown protein; IPR010666 (Zinc finger, GRF-type); GO:0008270 (zinc ion binding) |
| Araip.R8KWD | 0.02 | UDP-D-glucose/UDP-D-galactose 4-epimerase 5; IPR001509 (NAD-dependent epimerase/dehydratase), IPR016040 (NAD(P)-binding domain); GO:0003824 (catalytic activity), GO:0044237 (cellular metabolic process), GO:0050662 (coenzyme binding) |
| Araip.RV2ZZ | 0.02 | Unknown protein |
| Araip.RW074 | 0.03 | Unknown protein |
| Araip.S2QVQ | 0.06 | LysM receptor kinase K1B |
| Araip.S44X0 | 0.01 | CTV.20 n=1 Tax=Poncirus trifoliata RepID=Q8H6Q8_PONTR; IPR001878 (Zinc finger, CCHC-type); GO:0003676 (nucleic acid binding), GO:0008270 (zinc ion binding) |
| Araip.T63LZ | 0.01 | arginase; IPR000905 (Gcp-like domain), IPR006035 (Ureohydrolase), IPR023696 (Ureohydrolase domain); GO:0046872 (metal ion binding) |
| Araip.T9IY6 | 0.02 | Iron-sulfur cluster assembly accessory protein n=2 Tax=Cyanothece RepID=B7JUC7_CYAP8; IPR000361 (FeS cluster biogenesis), IPR016092 (FeS cluster insertion protein); GO:0005198 (structural molecule activity), GO:0016226 (iron-sulfur cluster assembly), GO:0051536 (iron-sulfur cluster binding) |
| Araip.TB7SW | 0.07 | uncharacterized protein At1g04910-like [Glycine max] |
| Araip.TRG04 | 0.01 | uncharacterized protein LOC100797259 isoform X3 [Glycine max]; IPR001878 (Zinc finger, CCHC-type), IPR004332 (Transposase, MuDR, plant), IPR007527 (Zinc finger, SWIM-type); GO:0003676 (nucleic acid binding), GO:0008270 (zinc ion binding) |
| Araip.U1B7Q | 0.01 | subtilisin-like serine protease 2; IPR015500 (Peptidase S8, subtilisin-related); GO:0004252 (serine-type endopeptidase activity), GO:0006508 (proteolysis), GO:0042802 (identical protein binding), GO:0043086 (negative regulation of catalytic activity) |
| Araip.U4091 | 0.04 | protein FAR1-RELATED SEQUENCE 5-like isoform X2 [Glycine max]; IPR004330 (FAR1 DNA binding domain) |
| Araip.U70G7 | 0.04 | unknown protein |
| Araip.UD3GJ | 0.03 | Unknown protein |
| Araip.V1KLK | 0.04 | GDP-mannose transporter GONST3-like isoform X2 [Glycine max] |
| Araip.V2S1C | 0.01 | Ulp1 protease family, carboxy-terminal domain protein |
| Araip.V70V3 | 0.01 | isoflavone reductase-like protein-like [Glycine max]; IPR008030 (NmrA-like), IPR016040 (NAD(P)-binding domain) |
| Araip.V9TZS | 0.03 | Unknown protein |
| Araip.VZP9J | 0.01 | stress up-regulated Nod 19 protein; IPR011692 (Stress up-regulated Nod 19) |
| Araip.W2968 | 0.03 | Myb/SANT-like DNA-binding domain protein; IPR024752 (Myb/SANT-like domain) |
| Araip.W5JAG | 0.07 | MYB transcription factor MYB64 [Glycine max]; IPR009057 (Homeodomain-like); GO:0003677 (DNA binding), GO:0003682 (chromatin binding) |
| Araip.WDN4K | 0.04 | Unknown protein |
| Araip.X58QR | 0.09 | alpha/beta-Hydrolases superfamily protein |
| Araip.X5L3I | 0.03 | uncharacterized protein LOC100785875 isoform X2 [Glycine max]; IPR024752 (Myb/SANT-like domain) |
| Araip.X62AY | 0.01 | Unknown protein; IPR015300 (DNA-binding pseudobarrel domain) |
| Araip.Y6M26 | 0.02 | Unknown protein |
| Araip.YMP06 | 0.17 | LysM receptor kinase K1B |
| Araip.YV6UI | 0.03 | uncharacterized protein LOC100778822 isoform X6 [Glycine max]; IPR004252 (Probable transposase, Ptta/En/Spm, plant) |
| Araip.YW6X3 | 0.03 | Unknown protein; IPR009057 (Homeodomain-like); GO:0003677 (DNA binding) |
| Araip.Z01V1 | 0.01 | Zinc knuckle family protein n=1 Tax=Oryza sativa subsp. japonica RepID=H2KWL4_ORYSJ; IPR001878 (Zinc finger, CCHC-type); GO:0003676 (nucleic acid binding), GO:0008270 (zinc ion binding) |
| Araip.ZPC3N | 0.04 | Unknown protein |
| Araip.ZX027 | 0.04 | Unknown protein |
| Pattee 7 seed | |  |
| Aradu.00U80 | 0.03 | Unknown protein |
| Aradu.10AEZ | 0.04 | caffeoylshikimate esterase-like isoform X3 [Glycine max]; IPR022742 (Putative lysophospholipase) |
| Aradu.347IZ | 0.02 | bifunctional protein FolD 2-like [Glycine max]; IPR020630 (Tetrahydrofolate dehydrogenase/cyclohydrolase, catalytic domain); GO:0003824 (catalytic activity), GO:0004488 (methylenetetrahydrofolate dehydrogenase (NADP+) activity), GO:0009396 (folic acid-containing compound biosynthetic process), GO:0055114 (oxidation-reduction process) |
| Aradu.3CF4E | 0.01 | kanadaptin-like isoform X1 [Glycine max] |
| Aradu.5B90X | 0.01 | replication protein A 70 kDa DNA-binding subunit C-like [Glycine max]; IPR012340 (Nucleic acid-binding, OB-fold) |
| Aradu.639LQ | 0.02 | Unknown protein |
| Aradu.6AU1U | 0.02 | unknown protein; FUNCTIONS IN: molecular_function unknown; INVOLVED IN: biological_process unknown; LOCATED IN: endomembrane system; EXPRESSED IN: 23 plant structures; EXPRESSED DURING: 13 growth stages |
| Aradu.6L1TM | 0.04 | Unknown protein |
| Aradu.7M22X | 0.01 | aluminum-activated malate transporter 1; IPR020966 (Aluminum-activated malate transporter); GO:0015743 (malate transport) |
| Aradu.7ZI8V | 0.01 | hippocampus abundant transcript-like protein |
| Aradu.8Y97R | 0.07 | LRR receptor-like kinase family protein |
| Aradu.9JQ04 | 0.07 | GRF zinc finger protein; IPR010666 (Zinc finger, GRF-type); GO:0008270 (zinc ion binding) |
| Aradu.A0AJG | 0.01 | serine/threonine-protein phosphatase 7 long form homolog [Glycine max]; IPR019557 (Aminotransferase-like, plant mobile domain) |
| Aradu.AIM62 | 0.04 | putative cyclin-D6-1-like isoform X1 [Glycine max] |
| Aradu.AQZ8D | 0.55 | Unknown protein |
| Aradu.B2V75 | 0.03 | Unknown protein |
| Aradu.BR5NX | 0.01 | Helicase-like protein n=1 Tax=Medicago truncatula RepID=G7JP74_MEDTR; IPR025476 (Helitron helicase-like domain) |
| Aradu.BW3MN | 0.01 | serine/threonine-protein phosphatase 7 long form homolog [Glycine max]; IPR019557 (Aminotransferase-like, plant mobile domain) |
| Aradu.D9M4W | 0.01 | DEAD-box ATP-dependent RNA helicase; IPR011545 (DNA/RNA helicase, DEAD/DEAH box type, N-terminal), IPR027417 (P-loop containing nucleoside triphosphate hydrolase); GO:0003676 (nucleic acid binding), GO:0005524 (ATP binding), GO:0008026 (ATP-dependent helicase activity) |
| Aradu.DDP86 | 0.06 | Unknown protein |
| Aradu.F9EMG | 0.02 | Polynucleotidyl transferase, ribonuclease H-like superfamily protein; IPR006941 (Ribonuclease CAF1), IPR012337 (Ribonuclease H-like domain); GO:0003676 (nucleic acid binding), GO:0005634 (nucleus) |
| Aradu.G7LFF | 0.01 | Ulp1 protease family, carboxy-terminal domain protein; IPR003653 (Peptidase C48, SUMO/Sentrin/Ubl1); GO:0006508 (proteolysis), GO:0008234 (cysteine-type peptidase activity) |
| Aradu.I3402 | 0.03 | cysteine-rich receptor-like protein kinase 10-like [Glycine max]; IPR002902 (Gnk2-homologous domain) |
| Aradu.K59WK | 0.01 | Unknown protein |
| Aradu.K6QVA | 0.04 | Unknown protein |
| Aradu.L1BAD | 0.04 | Unknown protein |
| Aradu.MI1YM | 0.05 | Unknown protein |
| Aradu.PQI0M | 0.03 | replication protein A 70 kDa DNA-binding subunit C-like [Glycine max]; IPR012340 (Nucleic acid-binding, OB-fold) |
| Aradu.Q04NV | 0.06 | mitochondrial adenine nucleotide transporter ADNT1-like isoform 2 [Glycine max]; IPR023395 (Mitochondrial carrier domain) |
| Aradu.QP6WZ | 0.07 | Unknown protein |
| Aradu.RHZ8Q | 0.03 | protein n=1 Tax=Oryza sativa subsp. japonica RepID=C7J9W8_ORYSJ |
| Aradu.RJ4SP | 0.03 | Unknown protein |
| Aradu.T7AAZ | 0.02 | replication factor-A carboxy-terminal domain protein |
| Aradu.W3HAE | 0.01 | zinc finger MYM-type protein 1-like [Glycine max]; IPR013763 (Cyclin-like), IPR025398 (Domain of unknown function DUF4371); GO:0005634 (nucleus) |
| Aradu.W9541 | 0.01 | arogenate dehydrogenase; IPR003099 (Prephenate dehydrogenase), IPR016040 (NAD(P)-binding domain); GO:0004665 (prephenate dehydrogenase (NADP+) activity), GO:0006571 (tyrosine biosynthetic process), GO:0008977 (prephenate dehydrogenase activity), GO:0055114 (oxidation-reduction process) |
| Aradu.X1IG8 | 0.28 | Zinc finger GRF-type protein n=1 Tax=Arachis hypogaea RepID=G0Y6U2_ARAHY; IPR010666 (Zinc finger, GRF-type); GO:0008270 (zinc ion binding) |
| Aradu.YX4GA | 0.01 | CTV.20 n=1 Tax=Poncirus trifoliata RepID=Q8H6Q8_PONTR |
| Aradu.Z7B17 | 0.01 | serine/threonine-protein phosphatase 7 long form homolog [Glycine max]; IPR019557 (Aminotransferase-like, plant mobile domain) |
| Araip.0B93Z | 0.01 | Helicase-like protein n=1 Tax=Medicago truncatula RepID=G7JC08_MEDTR |
| Araip.0G6XP | 0.03 | Unknown protein |
| Araip.0WR7D | 0.05 | Aminotransferase-like, plant mobile domain family protein; IPR019557 (Aminotransferase-like, plant mobile domain) |
| Araip.0YA6C | 0.03 | Unknown protein |
| Araip.2G38G | 0.02 | replication protein A 70 kDa DNA-binding subunit C-like [Glycine max]; IPR003871 (Domain of unknown function DUF223) |
| Araip.38VG0 | 0.01 | PfpI family intracellular protease n=3 Tax=Desulfovibrio RepID=D9YED7_9DELT; IPR002818 (ThiJ/PfpI) |
| Araip.49RA3 | 0.04 | Unknown protein |
| Araip.58SXG | 0.03 | Unknown protein |
| Araip.6F48R | 0.01 | Replication factor-A protein 1-related; IPR012340 (Nucleic acid-binding, OB-fold) |
| Araip.70G92 | 0.04 | myo-inositol oxygenase 2; IPR007828 (Inositol oxygenase); GO:0005506 (iron ion binding), GO:0005737 (cytoplasm), GO:0019310 (inositol catabolic process), GO:0050113 (inositol oxygenase activity), GO:0055114 (oxidation-reduction process) |
| Araip.7L1DI | 0.04 | Unknown protein; IPR010666 (Zinc finger, GRF-type); GO:0008270 (zinc ion binding) |
| Araip.7RE50 | 0.01 | Unknown protein |
| Araip.8123Q | 0.01 | protein FAR1-RELATED SEQUENCE 5-like [Glycine max] |
| Araip.94PWT | 0.01 | Unknown protein |
| Araip.B2X3C | 0.07 | protein pelota-like [Glycine max]; IPR004405 (Translation release factor pelota) |
| Araip.B3VTP | 0.01 | cellulose synthase 1; IPR013083 (Zinc finger, RING/FYVE/PHD-type) |
| Araip.D5EQP | 0.06 | Unknown protein |
| Araip.GH7S8 | 0.01 | Ulp1 protease family, carboxy-terminal domain protein; IPR003653 (Peptidase C48, SUMO/Sentrin/Ubl1); GO:0006508 (proteolysis), GO:0008234 (cysteine-type peptidase activity) |
| Araip.I3VJG | 0.01 | Ankyrin repeat family protein; IPR020683 (Ankyrin repeat-containing domain), IPR022771 (Wings apart-like protein); GO:0005515 (protein binding) |
| Araip.I96AN | 0.01 | serine/threonine-protein phosphatase 7 long form homolog [Glycine max]; IPR019557 (Aminotransferase-like, plant mobile domain) |
| Araip.J8KA7 | 0.08 | 4-coumarate:CoA ligase 3 |
| Araip.JKQ4I | 0.27 | Unknown protein |
| Araip.JSE66 | 0.05 | Unknown protein |
| Araip.JW9MM | 0.12 | Unknown protein |
| Araip.KY2XW | 0.02 | probable receptor protein kinase TMK1-like [Glycine max] |
| Araip.LXK1R | 0.01 | uncharacterized protein LOC102661513 isoform X2 [Glycine max]; IPR024752 (Myb/SANT-like domain) |
| Araip.MKV8R | 0.01 | protein FAR1-RELATED SEQUENCE 3-like isoform X1 [Glycine max]; IPR001878 (Zinc finger, CCHC-type); GO:0003676 (nucleic acid binding), GO:0008270 (zinc ion binding) |
| Araip.N9CKL | 0.01 | uncharacterized protein LOC100797259 isoform X3 [Glycine max]; IPR004332 (Transposase, MuDR, plant) |
| Araip.P4E2D | 0.04 | E3 UFM1-protein ligase-like protein |
| Araip.QI5FU | 0.03 | Helicase-like protein n=1 Tax=Medicago truncatula RepID=G7KCN8_MEDTR |
| Araip.QPS8L | 0.04 | Unknown protein |
| Araip.R42E8 | 0.02 | uncharacterized protein LOC100815031 isoform X1 [Glycine max] |
| Araip.R8KW3 | 0.01 | serine/threonine-protein phosphatase 7 long form homolog [Glycine max]; IPR019557 (Aminotransferase-like, plant mobile domain) |
| Araip.RS2EU | 0.03 | n=1 Tax=Oryza sativa subsp. japonica RepID=Q7F971_ORYSJ; IPR001878 (Zinc finger, CCHC-type), IPR007527 (Zinc finger, SWIM-type); GO:0003676 (nucleic acid binding), GO:0008270 (zinc ion binding) |
| Araip.S3FBK | 0.03 | Unknown protein |
| Araip.SAB0T | 0.01 | Helicase-like protein n=1 Tax=Medicago truncatula RepID=G7IIE5_MEDTR; IPR025476 (Helitron helicase-like domain) |
| Araip.SB1US | 0.06 | Unknown protein |
| Araip.TI4SH | 0.02 | Unknown protein |
| Araip.TR7CC | 0.03 | protein FAR1-RELATED SEQUENCE 6-like isoform X4 [Glycine max]; IPR004330 (FAR1 DNA binding domain) |
| Araip.U5MA9 | 0.01 | Unknown protein |
| Araip.VBD5R | 0.04 | Unknown protein |
| Araip.XB2Z7 | 0.18 | ubiquitin-protein ligase, cullin 4; IPR001373 (Cullin, N-terminal); GO:0006511 (ubiquitin-dependent protein catabolic process), GO:0031461 (cullin-RING ubiquitin ligase complex), GO:0031625 (ubiquitin protein ligase binding) |
| Araip.XJ7WN | 0.03 | Unknown protein |
| Araip.XJM91 | 0.03 | Peroxidase superfamily protein; IPR010255 (Haem peroxidase); GO:0004601 (peroxidase activity), GO:0006979 (response to oxidative stress), GO:0020037 (heme binding), GO:0055114 (oxidation-reduction process) |
| Araip.XUD06 | 0.08 | ubiquitin-protein ligase, cullin 4; IPR001373 (Cullin, N-terminal); GO:0006511 (ubiquitin-dependent protein catabolic process), GO:0031461 (cullin-RING ubiquitin ligase complex), GO:0031625 (ubiquitin protein ligase binding) |
| Araip.Y37QJ | 0.02 | zinc finger MYM-type protein 1-like [Glycine max]; IPR008906 (HAT dimerisation domain, C-terminal), IPR012337 (Ribonuclease H-like domain); GO:0003676 (nucleic acid binding), GO:0046983 (protein dimerization activity) |
| Araip.Y3X5F | 0.02 | zinc ion binding; IPR010666 (Zinc finger, GRF-type); GO:0008270 (zinc ion binding) |
| Pattee 8 seed | |  |
| Aradu.1TL3P | 0.01 | uncharacterized protein LOC100778909 isoform X7 [Glycine max]; IPR026103 (Harbinger transposase-derived nuclease, animal) |
| Aradu.40KHK | 0.03 | Ycf2 [Glycine max]; IPR008543 (Uncharacterised protein family Ycf2); GO:0005524 (ATP binding), GO:0009507 (chloroplast) |
| Aradu.56AVW | 0.12 | Unknown protein |
| Aradu.6E9LJ | 0.01 | replication factor-A carboxy-terminal domain protein; IPR012340 (Nucleic acid-binding, OB-fold) |
| Aradu.8KC25 | 0.06 | Cytochrome P450 superfamily protein; IPR001128 (Cytochrome P450); GO:0005506 (iron ion binding), GO:0020037 (heme binding), GO:0055114 (oxidation-reduction process) |
| Aradu.8W5Y8 | 0.03 | Unknown protein |
| Aradu.90LGN | 0.06 | Unknown protein |
| Aradu.955D1 | 0.09 | UDP-N-acetylglucosamine transferase subunit ALG14 isoform X7 [Glycine max] |
| Aradu.9R4TF | 0.01 | CTV.20 n=1 Tax=Poncirus trifoliata RepID=Q8H6Q8_PONTR |
| Aradu.9U4B9 | 0.14 | Unknown protein |
| Aradu.A5SUY | 0.03 | prohibitin 2 |
| Aradu.A7L9N | 0.01 | uncharacterized protein LOC100776940 isoform X2 [Glycine max]; IPR004332 (Transposase, MuDR, plant), IPR007527 (Zinc finger, SWIM-type); GO:0008270 (zinc ion binding) |
| Aradu.AB2YQ | 0.15 | C2H2-like zinc finger protein |
| Aradu.C2DIQ | 0.01 | CTV.20 n=1 Tax=Poncirus trifoliata RepID=Q8H6Q8_PONTR |
| Aradu.D73AE | 0.01 | Unknown protein |
| Aradu.D8AV4 | 0.04 | Unknown protein |
| Aradu.EVX4S | 0.04 | Unknown protein |
| Aradu.G4KWV | 0.12 | Unknown protein |
| Aradu.I1W70 | 0.44 | uncharacterized protein LOC102667982 [Glycine max] |
| Aradu.I7A3B | 0.02 | Unknown protein |
| Aradu.J4ZMT | 0.06 | S-adenosyl-L-methionine-dependent methyltransferases superfamily protein |
| Aradu.JA4ZV | 0.03 | isoflavone reductase homolog [Glycine max]; IPR008030 (NmrA-like), IPR016040 (NAD(P)-binding domain) |
| Aradu.LXL9C | 0.04 | Zinc knuckle family protein n=1 Tax=Oryza sativa subsp. japonica RepID=H2KW38_ORYSJ |
| Aradu.M8MLA | 0.07 | Unknown protein |
| Aradu.MQY39 | 0.04 | Unknown protein |
| Aradu.N7L1J | 0.03 | peroxisomal membrane carrier protein; IPR018108 (Mitochondrial substrate/solute carrier), IPR023395 (Mitochondrial carrier domain) |
| Aradu.NPW5H | 0.01 | Unknown protein |
| Aradu.P0M1K | 0.23 | hypothetical protein |
| Aradu.P0P34 | 0.04 | Rubisco methyltransferase family protein |
| Aradu.P13UP | 0.03 | Protein kinase superfamily protein; IPR011009 (Protein kinase-like domain); GO:0004672 (protein kinase activity), GO:0006468 (protein phosphorylation) |
| Aradu.QV14S | 0.01 | Ethylene insensitive 3 family protein; IPR023278 (Ethylene insensitive 3-like protein, DNA-binding domain); GO:0003700 (sequence-specific DNA binding transcription factor activity), GO:0005634 (nucleus) |
| Aradu.QWZ7V | 0.04 | protein FAR1-RELATED SEQUENCE 5-like isoform X4 [Glycine max]; IPR004330 (FAR1 DNA binding domain) |
| Aradu.R2W61 | 0.01 | Zinc knuckle family protein n=1 Tax=Oryza sativa subsp. japonica RepID=H2KW38_ORYSJ |
| Aradu.RVW5B | 0.01 | Polyprotein n=1 Tax=Citrus endogenous pararetrovirus RepID=V9QEM3_9RETR; IPR001878 (Zinc finger, CCHC-type); GO:0003676 (nucleic acid binding), GO:0008270 (zinc ion binding) |
| Aradu.TEI5G | 0.04 | ATP-dependent zinc metalloprotease FTSH-like protein; IPR027417 (P-loop containing nucleoside triphosphate hydrolase); GO:0005524 (ATP binding) |
| Aradu.TI91L | 0.01 | serine/threonine-protein phosphatase 7 long form homolog [Glycine max]; IPR019557 (Aminotransferase-like, plant mobile domain) |
| Aradu.TT16G | 0.03 | uncharacterized protein LOC102666958 [Glycine max] |
| Aradu.U9AJJ | 0.12 | Unknown protein |
| Aradu.W8H9Q | 0.01 | Phosphatidylinositol-4-phosphate 5-kinase family protein; IPR002498 (Phosphatidylinositol-4-phosphate 5-kinase, core), IPR023610 (Phosphatidylinositol-4-phosphate 5-kinase), IPR027484 (Phosphatidylinositol-4-phosphate 5-kinase, N-terminal domain); GO:0016307 (phosphatidylinositol phosphate kinase activity), GO:0046488 (phosphatidylinositol metabolic process) |
| Aradu.XP6LZ | 0.08 | NAD(P)H-quinone oxidoreductase subunit 2; IPR001750 (NADH:ubiquinone/plastoquinone oxidoreductase); GO:0008137 (NADH dehydrogenase (ubiquinone) activity), GO:0055114 (oxidation-reduction process) |
| Aradu.XT4FC | 0.01 | Unknown protein |
| Aradu.Y0QWH | 0.01 | RING/FYVE/PHD-type zinc finger family protein; IPR007461 (Ysc84 actin-binding domain) |
| Aradu.Y29GF | 0.07 | LRR receptor-like kinase family protein |
| Aradu.Z89VJ | 0.03 | Unknown protein |
| Aradu.ZE0CV | 0.05 | Unknown protein |
| Aradu.ZG5UW | 0.01 | disease resistance protein (TIR-NBS-LRR class), putative; IPR000157 (Toll/interleukin-1 receptor homology (TIR) domain), IPR002182 (NB-ARC), IPR027417 (P-loop containing nucleoside triphosphate hydrolase); GO:0005515 (protein binding), GO:0007165 (signal transduction), GO:0043531 (ADP binding) |
| Aradu.ZPX6J | 0.01 | Unknown protein; IPR015300 (DNA-binding pseudobarrel domain) |
| Aradu.ZV6IK | 0.04 | Unknown protein |
| Araip.3IR89 | 0.01 | RNA-binding protein 40-like [Glycine max]; IPR012677 (Nucleotide-binding, alpha-beta plait); GO:0000166 (nucleotide binding), GO:0003676 (nucleic acid binding) |
| Araip.3KF2T | 0.04 | nitrate transporter 1.2-like [Glycine max] |
| Araip.43LVD | 0.01 | putative nuclease HARBI1-like [Glycine max]; IPR026103 (Harbinger transposase-derived nuclease, animal), IPR027806 (Harbinger transposase-derived nuclease domain) |
| Araip.53WJE | 0.04 | cinnamyl alcohol dehydrogenase homolog 3; IPR002085 (Alcohol dehydrogenase superfamily, zinc-type), IPR011032 (GroES (chaperonin 10)-like); GO:0008270 (zinc ion binding), GO:0016491 (oxidoreductase activity), GO:0055114 (oxidation-reduction process) |
| Araip.72F4V | 0.01 | serine/threonine-protein phosphatase 7 long form homolog [Glycine max]; IPR019557 (Aminotransferase-like, plant mobile domain) |
| Araip.739PK | 0.01 | protein FAR1-RELATED SEQUENCE 6-like isoform X2 [Glycine max]; IPR004330 (FAR1 DNA binding domain), IPR006564 (Zinc finger, PMZ-type); GO:0008270 (zinc ion binding) |
| Araip.76U5P | 0.01 | minor allergen Alt a 7-like [Glycine max] |
| Araip.7J9AR | 0.01 | zinc finger MYM-type protein 1-like [Glycine max]; IPR008906 (HAT dimerisation domain, C-terminal), IPR012337 (Ribonuclease H-like domain), IPR025398 (Domain of unknown function DUF4371); GO:0003676 (nucleic acid binding), GO:0046983 (protein dimerization activity) |
| Araip.83CWY | 0.01 | Unknown protein |
| Araip.85CEU | 0.05 | Unknown protein |
| Araip.9T076 | 0.01 | Myb/SANT-like DNA-binding domain protein |
| Araip.A8BYM | 0.04 | Unknown protein |
| Araip.A97AI | 0.03 | RNA recognition motif (RRM) containing protein |
| Araip.ASY67 | 0.02 | replication factor-A carboxy-terminal domain protein; IPR012340 (Nucleic acid-binding, OB-fold) |
| Araip.B28JG | 0.05 | Unknown protein |
| Araip.B8CJT | 0.02 | Unknown protein; IPR010666 (Zinc finger, GRF-type); GO:0008270 (zinc ion binding) |
| Araip.CPM56 | 0.07 | serine acetyltransferase 1; 1; IPR011004 (Trimeric LpxA-like) |
| Araip.D7G59 | 0.04 | protein FAR1-RELATED SEQUENCE 12-like isoform X1 [Glycine max]; IPR004330 (FAR1 DNA binding domain) |
| Araip.E19VN | 0.13 | Unknown protein |
| Araip.EU94M | 0.03 | Unknown protein |
| Araip.GEU6Q | 0.03 | Unknown protein |
| Araip.GS2DK | 0.01 | Unknown protein |
| Araip.H7G2N | 0.01 | putative ubiquitin-like-specific protease 1B-like isoform X3 [Glycine max]; IPR003653 (Peptidase C48, SUMO/Sentrin/Ubl1); GO:0006508 (proteolysis), GO:0008234 (cysteine-type peptidase activity) |
| Araip.I6CDI | 0.04 | uncharacterized protein LOC100776940 isoform X3 [Glycine max]; IPR007527 (Zinc finger, SWIM-type); GO:0008270 (zinc ion binding) |
| Araip.I9MMX | 0.01 | uncharacterized protein LOC102662356 [Glycine max] |
| Araip.IHF1M | 0.03 | lysosomal Pro-X carboxypeptidase-like [Glycine max]; IPR008758 (Peptidase S28); GO:0006508 (proteolysis), GO:0008236 (serine-type peptidase activity) |
| Araip.J6YXX | 0.04 | GRF zinc finger protein; IPR010666 (Zinc finger, GRF-type); GO:0008270 (zinc ion binding) |
| Araip.JJ2NB | 0.01 | Zinc knuckle family protein n=1 Tax=Solanum demissum RepID=Q6L424_SOLDE |
| Araip.K0PT4 | 0.01 | protein FAR1-RELATED SEQUENCE 6-like isoform X2 [Glycine max]; IPR004330 (FAR1 DNA binding domain) |
| Araip.KM3LH | 0.03 | Calcium-dependent lipid-binding (CaLB domain) family protein; IPR000008 (C2 domain); GO:0005515 (protein binding) |
| Araip.LSE6T | 0.01 | Unknown protein |
| Araip.NM7R0 | 0.05 | Zinc finger MYM-type protein n=1 Tax=Medicago truncatula RepID=G7LFJ4_MEDTR |
| Araip.P08C3 | 0.03 | Myb/SANT-like DNA-binding domain protein; IPR024752 (Myb/SANT-like domain) |
| Araip.PN00V | 0.04 | pentatricopeptide (PPR) repeat-containing protein |
| Araip.R4CRQ | 0.12 | Unknown protein |
| Araip.RU9MC | 0.01 | Unknown protein |
| Araip.S18C9 | 0.02 | protein FAR1-RELATED SEQUENCE 6-like isoform X2 [Glycine max]; IPR006564 (Zinc finger, PMZ-type); GO:0008270 (zinc ion binding) |
| Araip.S9E29 | 0.01 | protein FAR1-RELATED SEQUENCE 6-like isoform X2 [Glycine max]; IPR007527 (Zinc finger, SWIM-type); GO:0008270 (zinc ion binding) |
| Araip.SN6C5 | 0.06 | Zinc finger GRF-type protein n=1 Tax=Arachis hypogaea RepID=G0Y6U2_ARAHY |
| Araip.SQ2UA | 0.03 | serine/threonine-protein phosphatase 7 long form homolog [Glycine max]; IPR019557 (Aminotransferase-like, plant mobile domain) |
| Araip.T6BHE | 0.03 | Unknown protein; IPR019557 (Aminotransferase-like, plant mobile domain) |
| Araip.TB8D8 | 0.04 | Peptidyl prolyl cis/trans isomerase, putative n=4 Tax=Candida RepID=B9W7L5_CANDC; IPR000297 (Peptidyl-prolyl cis-trans isomerase, PpiC-type); GO:0016853 (isomerase activity) |
| Araip.TS8P6 | 0.01 | Unknown protein; IPR003653 (Peptidase C48, SUMO/Sentrin/Ubl1); GO:0006508 (proteolysis), GO:0008234 (cysteine-type peptidase activity) |
| Araip.U4VN0 | 0.01 | protein FAR1-RELATED SEQUENCE 3-like isoform X1 [Glycine max] |
| Araip.U5I4Z | 0.04 | Unknown protein |
| Araip.UTZ6Y | 0.07 | Unknown protein |
| Araip.V2BVM | 0.01 | DNA-directed RNA polymerase subunit beta; IPR015712 (DNA-directed RNA polymerase, subunit 2); GO:0003677 (DNA binding), GO:0003899 (DNA-directed RNA polymerase activity), GO:0032549 (ribonucleoside binding) |
| Araip.V4WT8 | 0.02 | Unknown protein |
| Araip.X2I6E | 0.05 | trichohyalin-like isoform X4 [Glycine max] |
| Araip.X8FZQ | 0.07 | protein kinase family protein; IPR011009 (Protein kinase-like domain) |
| Araip.YG3K5 | 0.04 | Unknown protein |
| Araip.YIK7B | 0.03 | cinnamyl alcohol dehydrogenase homolog 3; IPR002085 (Alcohol dehydrogenase superfamily, zinc-type), IPR011032 (GroES (chaperonin 10)-like); GO:0008270 (zinc ion binding), GO:0016491 (oxidoreductase activity), GO:0055114 (oxidation-reduction process) |
| Araip.YQC4X | 0.05 | Unknown protein |
| Pattee 10 seed | |  |
| Aradu.1NC3L | 0.02 | Helicase-like protein n=1 Tax=Medicago truncatula RepID=G7J1F9_MEDTR; IPR025476 (Helitron helicase-like domain) |
| Aradu.6R6RB | 0.2 | AT-rich interactive domain protein |
| Aradu.81HJM | 0.03 | serine/threonine-protein phosphatase 7 long form homolog [Glycine max]; IPR019557 (Aminotransferase-like, plant mobile domain) |
| Aradu.8I40K | 0.01 | NADH dehydrogenase subunit 7, putative; IPR001135 (NADH-quinone oxidoreductase, subunit D); GO:0048038 (quinone binding), GO:0051287 (NAD binding), GO:0055114 (oxidation-reduction process) |
| Aradu.AL952 | 0.12 | Unknown protein |
| Aradu.HZA5U | 0.12 | ARM-repeat/Tetratricopeptide repeat (TPR)-like protein |
| Aradu.LI0S0 | 0.02 | serine/threonine-protein phosphatase 7 long form homolog [Glycine max]; IPR019557 (Aminotransferase-like, plant mobile domain) |
| Aradu.N79IX | 0.29 | Unknown protein |
| Aradu.NB07R | 0.03 | replication protein A 1A; IPR012340 (Nucleic acid-binding, OB-fold) |
| Aradu.Q6R61 | 0.04 | uncharacterized protein LOC100794856 [Glycine max] |
| Aradu.R0A9L | 0.04 | early nodulin-like protein 1-like [Glycine max]; IPR008972 (Cupredoxin); GO:0005507 (copper ion binding), GO:0009055 (electron carrier activity) |
| Aradu.R62M8 | 0.1 | Unknown protein |
| Aradu.TG7J0 | 0.03 | uncharacterized protein LOC100820019 isoform X4 [Glycine max] |
| Aradu.W47IW | 0.04 | serine/threonine-protein phosphatase 7 long form homolog [Glycine max]; IPR019557 (Aminotransferase-like, plant mobile domain) |
| Araip.4T95H | 0.18 | protein FAR1-RELATED SEQUENCE 11-like isoform X1 [Glycine max]; IPR004330 (FAR1 DNA binding domain) |
| Araip.5W8S7 | 0.04 | U-box domain-containing protein 33-like [Glycine max] |
| Araip.715N4 | 0.04 | Unknown protein |
| Araip.9PP86 | 0.03 | Unknown protein; IPR009027 (Ribosomal protein L9/RNase H1, N-terminal) |
| Araip.D54B6 | 0.02 | serine/threonine-protein phosphatase 7 long form homolog [Glycine max]; IPR019557 (Aminotransferase-like, plant mobile domain) |
| Araip.D5DLF | 0.1 | Unknown protein |
| Araip.I34RY | 0.12 | Unknown protein |
| Araip.JRD6J | 0.21 | serine/threonine-protein phosphatase 7 long form homolog [Glycine max]; IPR019557 (Aminotransferase-like, plant mobile domain) |
| Araip.L9X3W | 0.02 | CASP-like protein RCOM_0864260-like [Glycine max]; IPR006702 (Uncharacterised protein family UPF0497, trans-membrane plant) |
| Araip.M1G48 | 0.04 | serine/threonine-protein phosphatase 7 long form homolog [Glycine max]; IPR019557 (Aminotransferase-like, plant mobile domain) |
| Araip.MQ6LT | 0.14 | Unknown protein |
| Araip.P3YN7 | 0.15 | Protein kinase superfamily protein; IPR011009 (Protein kinase-like domain); GO:0004672 (protein kinase activity), GO:0004674 (protein serine/threonine kinase activity), GO:0005524 (ATP binding), GO:0006468 (protein phosphorylation) |
| Araip.RPI6Q | 0.34 | salicylic acid methyl transferase-like protein [Glycine max]; IPR005299 (SAM dependent carboxyl methyltransferase); GO:0008168 (methyltransferase activity) |
| Araip.W2C9M | 0.03 | DnaJ / Sec63 Brl domains-containing protein |
| Araip.Z770I | 0.03 | DnaJ / Sec63 Brl domains-containing protein |

Note: a, The log_2_-transformed FPKM values were considered as normalised the gene expression levels in these various tissues.

Table S2 Specific expressed genes of different developmental stages in leaf, shoot, gynophores, pod, pericarp, and seed tissues

| Gene name | Annotation |
| --- | --- |
| Leaf |  |
| Aradu.660J9 | protein FAR1-RELATED SEQUENCE 6-like isoform 1 [Glycine max]; IPR004330 (FAR1 DNA binding domain) |
| Aradu.8J77Z | Unknown protein |
| Aradu.J6JAI | protein FAR1-RELATED SEQUENCE 5-like [Glycine max]; IPR004330 (FAR1 DNA binding domain) |
| Aradu.P0E5I | uncharacterized protein LOC102664072 [Glycine max]; IPR025312 (Domain of unknown function DUF4216) |
| Aradu.W8T1W | histidine kinase 5 |
| Aradu.Z9W3C | Polyprotein n=5 Tax=Citrus endogenous pararetrovirus RepID=V9QF33_9RETR |
| Araip.00RKE | serine/threonine-protein phosphatase 7 long form homolog [Glycine max]; IPR019557 (Aminotransferase-like, plant mobile domain) |
| Araip.348W7 | unknown protein DS12 from 2D-PAGE of leaf, chloroplastic [Glycine max] |
| Araip.R526D | Ulp1 protease family, carboxy-terminal domain protein |
|  |  |
| Shoot |  |
| Aradu.JAU6Z | myo-inositol oxygenase 1; IPR007828 (Inositol oxygenase); GO:0005506 (iron ion binding), GO:0005737 (cytoplasm), GO:0019310 (inositol catabolic process), GO:0050113 (inositol oxygenase activity), GO:0055114 (oxidation-reduction process) |
| Aradu.M8JHF | Unknown protein |
| Aradu.R48AD | Chitinase family protein; IPR000726 (Glycoside hydrolase, family 19, catalytic), IPR023346 (Lysozyme-like domain); GO:0004568 (chitinase activity), GO:0006032 (chitin catabolic process), GO:0016998 (cell wall macromolecule catabolic process) |
| Aradu.SP4V4 | Unknown protein |
| Aradu.V9CTD | CTV.20 n=1 Tax=Poncirus trifoliata RepID=Q8H6Q8_PONTR |
| Aradu.X763N | 1-aminocyclopropane-1-carboxylate synthase 9; IPR015424 (Pyridoxal phosphate-dependent transferase); GO:0003824 (catalytic activity), GO:0009058 (biosynthetic process), GO:0030170 (pyridoxal phosphate binding) |
| Araip.05GPA | uncharacterized protein LOC100820019 isoform X4 [Glycine max]; IPR001878 (Zinc finger, CCHC-type), IPR025724 (GAG-pre-integrase domain); GO:0003676 (nucleic acid binding), GO:0008270 (zinc ion binding) |
| Araip.7G9C4 | hypothetical protein |
| Araip.85DXU | delta(7)-sterol-C5(6)-desaturase-like protein; IPR006694 (Fatty acid hydroxylase); GO:0005506 (iron ion binding), GO:0006633 (fatty acid biosynthetic process), GO:0016491 (oxidoreductase activity), GO:0055114 (oxidation-reduction process) |
| Araip.B42N3 | GDSL-like Lipase/Acylhydrolase superfamily protein; IPR001087 (Lipase, GDSL); GO:0006629 (lipid metabolic process), GO:0016787 (hydrolase activity) |
| Araip.C7JM9 | terpene synthase family, metal-binding domain protein; IPR008930 (Terpenoid cyclases/protein prenyltransferase alpha-alpha toroid), IPR008949 (Terpenoid synthase); GO:0000287 (magnesium ion binding), GO:0008152 (metabolic process), GO:0010333 (terpene synthase activity), GO:0016829 (lyase activity) |
| Araip.CT04S | serine/threonine-protein phosphatase 7 long form homolog [Glycine max]; IPR019557 (Aminotransferase-like, plant mobile domain) |
| Araip.FP1DJ | purine permease 4; IPR013657 (UAA transporter), IPR016196 (Major facilitator superfamily domain, general substrate transporter); GO:0055085 (transmembrane transport) |
| Araip.G23J4 | MATE efflux family protein; IPR002528 (Multi antimicrobial extrusion protein); GO:0006855 (drug transmembrane transport), GO:0015238 (drug transmembrane transporter activity), GO:0015297 (antiporter activity), GO:0016020 (membrane), GO:0055085 (transmembrane transport) |
| Araip.G4LIH | purine permease 4; IPR013657 (UAA transporter); GO:0055085 (transmembrane transport) |
| Araip.S7WEN | Unknown protein |
| Araip.T74VP | GRF zinc finger protein; IPR010666 (Zinc finger, GRF-type); GO:0008270 (zinc ion binding) |
|  |  |
| Gynophore |  |
| Aradu.UF6GR | Haloacid dehalogenase-like hydrolase (HAD) superfamily protein; IPR023214 (HAD-like domain) |
| Aradu.EJE04 | Unknown protein |
| Araip.4L2JI | Unknown protein |
|  |  |
| Pod |  |
| Aradu.EDM0A | protein FAR1-RELATED SEQUENCE 6-like isoform X2 [Glycine max]; IPR004330 (FAR1 DNA binding domain), IPR007527 (Zinc finger, SWIM-type); GO:0008270 (zinc ion binding) |
| Araip.CDX9M | Actin-binding FH2 (Formin Homology) protein; IPR015425 (Formin, FH2 domain), IPR027643 (Formin-like family, plant); GO:0005884 (actin filament), GO:0045010 (actin nucleation) |
| Aradu.F5Y3A | Unknown protein |
| Araip.YK6CS | Unknown protein |
|  |  |
| Pericarp |  |
| Aradu.E1VV0 | uncharacterized protein LOC100797259 isoform X4 [Glycine max]; IPR001878 (Zinc finger, CCHC-type), IPR004332 (Transposase, MuDR, plant); GO:0003676 (nucleic acid binding), GO:0008270 (zinc ion binding) |
| Araip.IBV65 | serine/threonine-protein phosphatase 7 long form homolog [Glycine max]; IPR019557 (Aminotransferase-like, plant mobile domain) |
| Araip.JUF6I | Unknown protein |
|  |  |
| Seed |  |
| Aradu.0B4Z4 | Late embryogenesis abundant protein, group 1 protein; IPR005513 (Late embryogenesis abundant protein, LEA-25/LEA-D113); GO:0009790 (embryo development) |
| Aradu.3E1ZE | Helicase-like protein n=1 Tax=Medicago truncatula RepID=G7J0A2_MEDTR; IPR012340 (Nucleic acid-binding, OB-fold) |
| Aradu.3U42M | beta-galactosidase 8; IPR001944 (Glycoside hydrolase, family 35); GO:0005975 (carbohydrate metabolic process) |
| Aradu.EE03A | Unknown protein |
| Aradu.FD81T | glycine-rich RNA-binding, abscisic acid-inducible protein-like [Glycine max]; IPR010800 (Glycine rich protein) |
| Aradu.M3MXB | leguminosin group486 secreted peptide; IPR010264 (Plant self-incompatibility S1) |
| Aradu.SIH82 | DUF223 domain protein; IPR012340 (Nucleic acid-binding, OB-fold) |
| Aradu.V12MU | Unknown protein |
| Aradu.WJ8MB | hypothetical protein; IPR007527 (Zinc finger, SWIM-type); GO:0008270 (zinc ion binding) |
| Aradu.WM0EZ | Lipid transfer protein; IPR016140 (Bifunctional inhibitor/plant lipid transfer protein/seed storage helical domain) |
| Aradu.Y48ZX | LOB domain-containing protein 18 |
| Araip.837LF | Adenine nucleotide alpha hydrolases-like superfamily protein; IPR014729 (Rossmann-like alpha/beta/alpha sandwich fold) |
| Araip.88WRZ | replication factor-A carboxy-terminal domain protein |
| Araip.CIT63 | short-chain dehydrogenase/reductase; IPR016040 (NAD(P)-binding domain) |
| Araip.D7VXB | ATP-dependent RNA helicase |
| Araip.DB4TT | serine/threonine-protein phosphatase 7 long form homolog [Glycine max]; IPR019557 (Aminotransferase-like, plant mobile domain) |
| Araip.EJ2V2 | serine/threonine-protein phosphatase 7 long form homolog [Glycine max]; IPR019557 (Aminotransferase-like, plant mobile domain) |
| Araip.F87A5 | Unknown protein |
| Araip.FUI9M | Ribonuclease HI n=2 Tax=Peptoniphilus RepID=E0NJN4_9FIRM; IPR009027 (Ribosomal protein L9/RNase H1, N-terminal) |
| Araip.G5T2B | putative ubiquitin-like-specific protease 1B-like isoform X3 [Glycine max]; IPR003653 (Peptidase C48, SUMO/Sentrin/Ubl1); GO:0006508 (proteolysis), GO:0008234 (cysteine-type peptidase activity) |
| Araip.I7UMT | ankyrin repeat protein; IPR026961 (PGG domain) |
| Araip.NH28H | Unknown protein |
| Araip.P6LRS | protein COBRA-like [Glycine max] |
| Araip.Y7ARM | Unknown protein |
| Araip.ZH9GA | replication factor-A carboxy-terminal domain protein; IPR012340 (Nucleic acid-binding, OB-fold) |

Table S3 The detailed GO annotation in tissue-specific gene

| GO ID | Number | Name | Ontology | Definition |
| --- | --- | --- | --- | --- |
| GO:0000015 | 1 | Phosphopyruvate hydratase complex | Cellular Component | A multimeric enzyme complex, usually a dimer or an octamer, that catalyzes the conversion of 2-phospho-D-glycerate to phosphoenolpyruvate and water. |
| GO:0000027 | 1 | Ribosomal large subunit assembly | Biological Process | The aggregation, arrangement and bonding together of constituent RNAs and proteins to form the large ribosomal subunit. |
| GO:0000028 | 5 | Ribosomal small subunit assembly | Biological Process | The aggregation, arrangement and bonding together of constituent RNAs and proteins to form the small ribosomal subunit. |
| GO:0000082 | 1 | G1/S transition of mitotic cell cycle | Biological Process | The mitotic cell cycle transition by which a cell in G1 commits to S phase. The process begins with the build up of G1 cyclin-dependent kinase (G1 CDK), resulting in the activation of transcription of G1 cyclins. The process ends with the positive feedback of the G1 cyclins on the G1 CDK which commits the cell to S phase, in which DNA replication is initiated. |
| GO:0000103 | 2 | Sulfate assimilation | Biological Process | The pathways by which inorganic sulfate is processed and incorporated into sulfated compounds. |
| GO:0000105 | 2 | Histidine biosynthetic process | Biological Process | The chemical reactions and pathways resulting in the formation of histidine, 2-amino-3-(1H-imidazol-4-yl)propanoic acid. |
| GO:0000139 | 1 | Golgi membrane | Cellular Component | The lipid bilayer surrounding any of the compartments of the Golgi apparatus. |
| GO:0000145 | 2 | Exocyst | Cellular Component | A protein complex peripherally associated with the plasma membrane that determines where vesicles dock and fuse. At least eight complex components are conserved between yeast and mammals. |
| GO:0000149 | 2 | SNARE binding | Molecular Function | Interacting selectively and non-covalently with a SNARE (soluble N-ethylmaleimide-sensitive factor attached protein receptor) protein. |
| GO:0000151 | 4 | Ubiquitin ligase complex | Cellular Component | A protein complex that includes a ubiquitin-protein ligase and enables ubiquitin protein ligase activity. The complex also contains other proteins that may confer substrate specificity on the complex. |
| GO:0000159 | 2 | Protein phosphatase type 2A complex | Cellular Component | A protein complex that has protein serine/threonine phosphatase activity that is polycation-stimulated (PCS), being directly stimulated by protamine, polylysine, or histone H1; it constitutes a subclass of several enzymes activated by different histones and polylysine, and consists of catalytic, scaffolding, and regulatory subunits. The catalytic and scaffolding subunits form the core enzyme, and the holoenzyme also includes the regulatory subunit. |
| GO:0000160 | 1 | Phosphorelay signal transduction system | Biological Process | A conserved series of molecular signals found in prokaryotes and eukaryotes; involves autophosphorylation of a histidine kinase and the transfer of the phosphate group to an aspartate that then acts as a phospho-donor to response regulator proteins. |
| GO:0000166 | 23 | Nucleotide binding | Molecular Function | Interacting selectively and non-covalently with a nucleotide, any compound consisting of a nucleoside that is esterified with (ortho)phosphate or an oligophosphate at any hydroxyl group on the ribose or deoxyribose. |
| GO:0000209 | 3 | Protein polyubiquitination | Biological Process | Addition of multiple ubiquitin groups to a protein, forming a ubiquitin chain. |
| GO:0000254 | 1 | C-4 methylsterol oxidase activity | Molecular Function | Catalysis of the reaction: 4,4-dimethyl-5-alpha-cholesta-8,24-dien-3-beta-ol + NAD(P)H + H(+) + O2 = 4-beta-hydroxymethyl-4-alpha-methyl-5-alpha-cholesta-8,24-dien-3-beta-ol + NAD(P)+ + H2O. |
| GO:0000287 | 8 | Magnesium ion binding | Molecular Function | Interacting selectively and non-covalently with magnesium (Mg) ions. |
| GO:0000289 | 1 | Nuclear-transcribed mRNA poly(A) tail shortening | Biological Process | Shortening of the poly(A) tail of a nuclear-transcribed mRNA from full length to an oligo(A) length. |
| GO:0000290 | 1 | Deadenylation-dependent decapping of nuclear-transcribed mRNA | Biological Process | Cleavage of the 5'-cap of a nuclear mRNA triggered by shortening of the poly(A) tail to below a minimum functional length. |
| GO:0000398 | 1 | mRNA splicing, via spliceosome | Biological Process | The joining together of exons from one or more primary transcripts of messenger RNA (mRNA) and the excision of intron sequences, via a spliceosomal mechanism, so that mRNA consisting only of the joined exons is produced. |
| GO:0000413 | 2 | Protein peptidyl-prolyl isomerization | Biological Process | The modification of a protein by cis-trans isomerization of a proline residue. |
| GO:0000428 | 1 | DNA-directed RNA polymerase complex | Cellular Component | A protein complex that possesses DNA-directed RNA polymerase activity. |
| GO:0000447 | 1 | Endonucleolytic cleavage in ITS1 to separate SSU-rRNA from 5.8S rRNA and LSU-rRNA from tricistronic rRNA transcript (SSU-rRNA, 5.8S rRNA, LSU-rRNA) | Biological Process | Endonucleolytic cleavage between the SSU-rRNA and the 5.8S rRNA of an rRNA molecule originally produced as a tricistronic rRNA transcript that contained the Small SubUnit (SSU) rRNA, the 5.8S rRNA, and the Large SubUnit (LSU) rRNA, in that order, from 5' to 3' along the primary transcript. |
| GO:0000461 | 1 | Endonucleolytic cleavage to generate mature 3'-end of SSU-rRNA from (SSU-rRNA, 5.8S rRNA, LSU-rRNA) | Biological Process | Endonucleolytic cleavage at the 3'-end of the SSU-rRNA from an originally tricistronic rRNA transcript that contained the Small Subunit (SSU) rRNA, the 5.8S rRNA, and the Large Subunit (LSU) rRNA in that order from 5' to 3' along the primary transcript, to produce the mature end of the SSU-rRNA. |
| GO:0000462 | 1 | Maturation of SSU-rRNA from tricistronic rRNA transcript (SSU-rRNA, 5.8S rRNA, LSU-rRNA) | Biological Process | Any process involved in the maturation of a precursor Small SubUnit (SSU) ribosomal RNA (rRNA) molecule into a mature SSU-rRNA molecule from the pre-rRNA molecule originally produced as a tricistronic rRNA transcript that contains the Small Subunit (SSU) rRNA, 5.8S rRNA, and the Large Subunit (LSU) in that order from 5' to 3' along the primary transcript. |
| GO:0000723 | 3 | Telomere maintenance | Biological Process | Any process that contributes to the maintenance of proper telomeric length and structure by affecting and monitoring the activity of telomeric proteins, the length of telomeric DNA and the replication and repair of the DNA. These processes includes those that shorten, lengthen, replicate and repair the telomeric DNA sequences. |
| GO:0000785 | 2 | Chromatin | Cellular Component | The ordered and organized complex of DNA, protein, and sometimes RNA, that forms the chromosome. |
| GO:0000786 | 1 | Nucleosome | Cellular Component | A complex comprised of DNA wound around a multisubunit core and associated proteins, which forms the primary packing unit of DNA into higher order structures. |
| GO:0000795 | 1 | Synaptonemal complex | Cellular Component | A proteinaceous scaffold found between homologous chromosomes during meiosis. It consists of 2 lateral elements and a central element, all running parallel to each other. Transverse filaments connect the lateral elements to the central element. |
| GO:0000796 | 2 | Condensin complex | Cellular Component | A multisubunit protein complex that plays a central role in chromosome condensation. |
| GO:0000914 | 1 | Phragmoplast assembly | Biological Process | The formation of a structure composed of actin, myosin, and associated proteins that will function in cytokinesis in cells that perform cytokinesis by cell plate formation. The structure usually contains antiparallel microtubules and membrane (often visible as vesicles). |
| GO:0000932 | 1 | P-body | Cellular Component | A focus in the cytoplasm where mRNAs may become inactivated by decapping or some other mechanism. Protein and RNA localized to these foci are involved in mRNA degradation, nonsense-mediated mRNA decay (NMD), translational repression, and RNA-mediated gene silencing. |
| GO:0000938 | 1 | GARP complex | Cellular Component | A quatrefoil tethering complex required for retrograde traffic from the early endosome back to the late Golgi and biogenesis of cytoplasmic vesicles. |
| GO:0000976 | 1 | Transcription regulatory region sequence-specific DNA binding | Molecular Function | Interacting selectively and non-covalently with a specific sequence of DNA that is part of a regulatory region that controls transcription of that section of the DNA. The transcribed region might be described as a gene, cistron, or operon. |
| GO:0000977 | 1 | RNA polymerase II regulatory region sequence-specific DNA binding | Molecular Function | Interacting selectively and non-covalently with a specific sequence of DNA that is part of a regulatory region that controls the transcription of a gene or cistron by RNA polymerase II. |
| GO:0000981 | 9 | RNA polymerase II transcription factor activity, sequence-specific DNA binding | Molecular Function | Interacting selectively and non-covalently with a specific DNA sequence in order to modulate transcription by RNA polymerase II. The transcription factor may or may not also interact selectively with a protein or macromolecular complex. |
| GO:0000982 | 1 | Transcription factor activity, RNA polymerase II core promoter proximal region sequence-specific binding | Molecular Function | Interacting selectively and non-covalently with a sequence of DNA that is in cis with and relatively close to a core promoter for RNA polymerase II (RNAP II) in order to modulate transcription by RNAP II. |
| GO:0000987 | 1 | Core promoter proximal region sequence-specific DNA binding | Molecular Function | Interacting selectively and non-covalently with a sequence of DNA that is in cis with and relatively close to the core promoter. The transcribed region might be described as a gene, cistron, or operon. |
| GO:0000989 | 1 | Transcription factor activity, transcription factor binding | Molecular Function | Interacting selectively and non-covalently with a specific transcription factor, which may be a single protein or a complex, in order to modulate transcription. A protein binding transcription factor may or may not also interact with the template nucleic acid (either DNA or RNA) as well. |
| GO:0001056 | 2 | RNA polymerase III activity | Molecular Function | Catalysis of the reaction: nucleoside triphosphate + RNA(n) = diphosphate + RNA(n+1). Utilizes a DNA template that contains an RNA polymerase III specific promoter to direct initiation and catalyses DNA-template-directed extension of the 3'-end of an RNA strand by one nucleotide at a time. Can initiate a chain 'de novo'. |
| GO:0001077 | 1 | Transcriptional activator activity, RNA polymerase II core promoter proximal region sequence-specific binding | Molecular Function | Interacting selectively and non-covalently with a sequence of DNA that is in cis with and relatively close to a core promoter for RNA polymerase II (RNAP II) in order to activate or increase the frequency, rate or extent of transcription from the RNAP II promoter. |
| GO:0001510 | 1 | RNA methylation | Biological Process | Posttranscriptional addition of a methyl group to either a nucleotide or 2'-O ribose in a polyribonucleotide. Usually uses S-adenosylmethionine as a cofactor. |
| GO:0001676 | 1 | Long-chain fatty acid metabolic process | Biological Process | The chemical reactions and pathways involving long-chain fatty acids, A long-chain fatty acid is a fatty acid with a chain length between C13 and C22. |
| GO:0001758 | 1 | Retinal dehydrogenase activity | Molecular Function | Catalysis of the reaction: retinal + NAD+ + H2O = retinoate + NADH. Acts on both 11-trans and 13-cis forms of retinal. |
| GO:0002181 | 2 | Cytoplasmic translation | Biological Process | The chemical reactions and pathways resulting in the formation of a protein in the cytoplasm. This is a ribosome-mediated process in which the information in messenger RNA (mRNA) is used to specify the sequence of amino acids in the protein. |
| GO:0003333 | 1 | Amino acid transmembrane transport | Biological Process | The directed movement of amino acids, organic acids containing one or more amino substituents across a membrane by means of some agent such as a transporter or pore. |
| GO:0003676 | 128 | Nucleic acid binding | Molecular Function | Interacting selectively and non-covalently with any nucleic acid. |
| GO:0003677 | 57 | DNA binding | Molecular Function | Any molecular function by which a gene product interacts selectively and non-covalently with DNA (deoxyribonucleic acid). |
| GO:0003678 | 1 | DNA helicase activity | Molecular Function | Catalysis of the reaction: NTP + H2O = NDP + phosphate, to drive the unwinding of a DNA helix. |
| GO:0003682 | 1 | Chromatin binding | Molecular Function | Interacting selectively and non-covalently with chromatin, the network of fibers of DNA, protein, and sometimes RNA, that make up the chromosomes of the eukaryotic nucleus during interphase. |
| GO:0003684 | 2 | Damaged DNA binding | Molecular Function | Interacting selectively and non-covalently with damaged DNA. |
| GO:0003700 | 14 | Transcription factor activity, sequence-specific DNA binding | Molecular Function | Interacting selectively and non-covalently with a specific DNA sequence in order to modulate transcription. The transcription factor may or may not also interact selectively with a protein or macromolecular complex. |
| GO:0003712 | 1 | Transcription cofactor activity | Molecular Function | Interacting selectively and non-covalently with a regulatory transcription factor and also with the basal transcription machinery in order to modulate transcription. Cofactors generally do not bind the template nucleic acid, but rather mediate protein-protein interactions between regulatory transcription factors and the basal transcription machinery. |
| GO:0003723 | 11 | RNA binding | Molecular Function | Interacting selectively and non-covalently with an RNA molecule or a portion thereof. |
| GO:0003729 | 2 | mRNA binding | Molecular Function | Interacting selectively and non-covalently with messenger RNA (mRNA), an intermediate molecule between DNA and protein. mRNA includes UTR and coding sequences, but does not contain introns. |
| GO:0003735 | 32 | Structural constituent of ribosome | Molecular Function | The action of a molecule that contributes to the structural integrity of the ribosome. |
| GO:0003743 | 2 | Translation initiation factor activity | Molecular Function | Functions in the initiation of ribosome-mediated translation of mRNA into a polypeptide. |
| GO:0003746 | 1 | Translation elongation factor activity | Molecular Function | Functions in chain elongation during polypeptide synthesis at the ribosome. |
| GO:0003755 | 3 | Peptidyl-prolyl cis-trans isomerase activity | Molecular Function | Catalysis of the reaction: peptidyl-proline (omega=180) = peptidyl-proline (omega=0). |
| GO:0003774 | 1 | Motor activity | Molecular Function | Catalysis of the generation of force resulting either in movement along a microfilament or microtubule, or in torque resulting in membrane scission, coupled to the hydrolysis of a nucleoside triphosphate. |
| GO:0003777 | 4 | Microtubule motor activity | Molecular Function | Catalysis of movement along a microtubule, coupled to the hydrolysis of a nucleoside triphosphate (usually ATP). |
| GO:0003824 | 18 | Catalytic activity | Molecular Function | Catalysis of a biochemical reaction at physiological temperatures. In biologically catalyzed reactions, the reactants are known as substrates, and the catalysts are naturally occurring macromolecular substances known as enzymes. Enzymes possess specific binding sites for substrates, and are usually composed wholly or largely of protein, but RNA that has catalytic activity (ribozyme) is often also regarded as enzymatic. |
| GO:0003841 | 2 | 1-acylglycerol-3-phosphate O-acyltransferase activity | Molecular Function | Catalysis of the reaction: acyl-CoA + 1-acyl-sn-glycerol-3-phosphate = CoA + 1,2-diacyl-sn-glycerol-3-phosphate. |
| GO:0003844 | 1 | 1,4-alpha-glucan branching enzyme activity | Molecular Function | Catalysis of the transfer of a segment of a (1->4)-alpha-D-glucan chain to a primary hydroxyl group in a similar glucan chain. |
| GO:0003854 | 1 | 3-beta-hydroxy-delta5-steroid dehydrogenase activity | Molecular Function | Catalysis of the reaction: a 3-beta-hydroxy-delta(5)-steroid + NAD+ = a 3-oxo-delta(5)-steroid + NADH + H(+). |
| GO:0003872 | 1 | 6-phosphofructokinase activity | Molecular Function | Catalysis of the reaction: ATP + D-fructose-6-phosphate = ADP + D-fructose 1,6-bisphosphate. |
| GO:0003876 | 2 | AMP deaminase activity | Molecular Function | Catalysis of the reaction: AMP + H2O = IMP + NH3. |
| GO:0003879 | 1 | ATP phosphoribosyltransferase activity | Molecular Function | Catalysis of the reaction: 1-(5-phospho-D-ribosyl)-ATP + diphosphate = ATP + 5-phospho-alpha-D-ribose 1-diphosphate. |
| GO:0003887 | 2 | DNA-directed DNA polymerase activity | Molecular Function | Catalysis of the reaction: deoxynucleoside triphosphate + DNA(n) = diphosphate + DNA(n+1); the synthesis of DNA from deoxyribonucleotide triphosphates in the presence of a DNA template and a 3'hydroxyl group. |
| GO:0003899 | 5 | DNA-directed 5'-3' RNA polymerase activity | Molecular Function | Catalysis of the reaction: nucleoside triphosphate + RNA(n) = diphosphate + RNA(n+1). Utilizes a DNA template, i.e. the catalysis of DNA-template-directed extension of the 3'-end of an RNA strand by one nucleotide at a time. Can initiate a chain 'de novo'. |
| GO:0003910 | 2 | DNA ligase (ATP) activity | Molecular Function | Catalysis of the reaction: ATP + deoxyribonucleotide(n) + deoxyribonucleotide(m) = AMP + diphosphate + deoxyribonucleotide(n+m). |
| GO:0003924 | 1 | GTPase activity | Molecular Function | Catalysis of the reaction: GTP + H2O = GDP + phosphate. |
| GO:0003955 | 1 | NAD(P)H dehydrogenase (quinone) activity | Molecular Function | Catalysis of the reaction: NAD(P)H + H+ + a quinone = NAD(P)+ + a hydroquinone. |
| GO:0003978 | 2 | UDP-glucose 4-epimerase activity | Molecular Function | Catalysis of the reaction: UDP-glucose = UDP-galactose. |
| GO:0003985 | 1 | Acetyl-CoA C-acetyltransferase activity | Molecular Function | Catalysis of the reaction: 2 acetyl-CoA = CoA + acetoacetyl-CoA. |
| GO:0003989 | 3 | Acetyl-CoA carboxylase activity | Molecular Function | Catalysis of the reaction: ATP + acetyl-CoA + HCO3- = ADP + phosphate + malonyl-CoA. |
| GO:0003999 | 1 | Adenine phosphoribosyltransferase activity | Molecular Function | Catalysis of the reaction: AMP + diphosphate = adenine + 5-phospho-alpha-D-ribose 1-diphosphate. |
| GO:0004003 | 1 | ATP-dependent DNA helicase activity | Molecular Function | Catalysis of the reaction: ATP + H2O = ADP + phosphate; this reaction drives the unwinding of the DNA helix. |
| GO:0004004 | 1 | ATP-dependent RNA helicase activity | Molecular Function | Catalysis of the reaction: ATP + H2O = ADP + phosphate; this reaction drives the unwinding of an RNA helix. |
| GO:0004020 | 1 | Adenylylsulfate kinase activity | Molecular Function | Catalysis of the reaction: ATP + adenylylsulfate = ADP + 3'-phosphoadenosine 5'-phosphosulfate. |
| GO:0004022 | 2 | Alcohol dehydrogenase (NAD) activity | Molecular Function | Catalysis of the reaction: an alcohol + NAD+ = an aldehyde or ketone + NADH + H+. |
| GO:0004028 | 2 | 3-chloroallyl aldehyde dehydrogenase activity | Molecular Function | Catalysis of the reaction: 3-chloroallyl aldehyde + H2O = 2 H+ + 2 e- + 3-chloroacrylic acid. |
| GO:0004029 | 3 | Aldehyde dehydrogenase (NAD) activity | Molecular Function | Catalysis of the reaction: an aldehyde + NAD+ + H2O = an acid + NADH + H+. |
| GO:0004030 | 2 | Aldehyde dehydrogenase [NAD(P)+] activity | Molecular Function | Catalysis of the reaction: an aldehyde + NAD(P)+ + H2O = an acid + NAD(P)H + H+. |
| GO:0004045 | 2 | Aminoacyl-tRNA hydrolase activity | Molecular Function | Catalysis of the reaction: N-substituted aminoacyl-tRNA + H2O = N-substituted amino acid + tRNA. |
| GO:0004057 | 1 | Arginyltransferase activity | Molecular Function | Catalysis of the reaction: L-arginyl-tRNA + protein = tRNA + L-arginyl-protein. |
| GO:0004066 | 2 | Asparagine synthase (glutamine-hydrolyzing) activity | Molecular Function | Catalysis of the reaction: ATP + L-aspartate + L-glutamine = AMP + diphosphate + L-asparagine + L-glutamate. |
| GO:0004096 | 1 | Catalase activity | Molecular Function | Catalysis of the reaction: 2 hydrogen peroxide = O2 + 2 H2O. |
| GO:0004109 | 1 | Coproporphyrinogen oxidase activity | Molecular Function | Catalysis of the reaction: coproporphyrinogen III + 2 H(+) + O(2) = 2 CO(2) + 2 H(2)O + protoporphyrinogen IX. |
| GO:0004121 | 1 | Cystathionine beta-lyase activity | Molecular Function | Catalysis of the reaction: cystathionine + H2O = L-homocysteine + NH3 + pyruvate. |
| GO:0004124 | 1 | Cysteine synthase activity | Molecular Function | Catalysis of the reaction: O3-acetyl-L-serine + hydrogen sulfide = L-cysteine + acetate. |
| GO:0004129 | 2 | Cytochrome-c oxidase activity | Molecular Function | Catalysis of the reaction: 4 ferrocytochrome c + O2 + 4 H+ = 4 ferricytochrome c + 2 H2O. |
| GO:0004134 | 1 | 4-alpha-glucanotransferase activity | Molecular Function | Catalysis of the transfer of a segment of a (1->4)-alpha-D-glucan to a new 4-position in an acceptor, which may be glucose or (1->4)-alpha-D-glucan. |
| GO:0004143 | 1 | Diacylglycerol kinase activity | Molecular Function | Catalysis of the reaction: NTP + 1,2-diacylglycerol = NDP + 1,2-diacylglycerol-3-phosphate. |
| GO:0004170 | 1 | dUTP diphosphatase activity | Molecular Function | Catalysis of the reaction: dUTP + H2O = dUMP + diphosphate. |
| GO:0004176 | 2 | ATP-dependent peptidase activity | Molecular Function | Catalysis of the reaction: ATP + H2O = ADP + phosphate, to drive the hydrolysis of peptide bonds. |
| GO:0004177 | 1 | Aminopeptidase activity | Molecular Function | Catalysis of the hydrolysis of N-terminal amino acid residues from in a polypeptide chain. |
| GO:0004185 | 1 | Serine-type carboxypeptidase activity | Molecular Function | Catalysis of the hydrolysis of a peptide bond not more than three residues from the C-terminus of a polypeptide chain by a catalytic mechanism that involves a catalytic triad consisting of a serine nucleophile that is activated by a proton relay involving an acidic residue (e.g. aspartate or glutamate) and a basic residue (usually histidine). |
| GO:0004190 | 7 | Aspartic-type endopeptidase activity | Molecular Function | Catalysis of the hydrolysis of internal, alpha-peptide bonds in a polypeptide chain by a mechanism in which a water molecule bound by the side chains of aspartic residues at the active center acts as a nucleophile. |
| GO:0004197 | 4 | Cysteine-type endopeptidase activity | Molecular Function | Catalysis of the hydrolysis of internal, alpha-peptide bonds in a polypeptide chain by a mechanism in which the sulfhydryl group of a cysteine residue at the active center acts as a nucleophile. |
| GO:0004252 | 4 | Serine-type endopeptidase activity | Molecular Function | Catalysis of the hydrolysis of internal, alpha-peptide bonds in a polypeptide chain by a catalytic mechanism that involves a catalytic triad consisting of a serine nucleophile that is activated by a proton relay involving an acidic residue (e.g. aspartate or glutamate) and a basic residue (usually histidine). |
| GO:0004326 | 1 | Tetrahydrofolylpolyglutamate synthase activity | Molecular Function | Catalysis of the reaction: ATP + tetrahydrofolyl-(Glu)(n) + L-glutamate = ADP + phosphate + tetrahydrofolyl-(Glu)(n+1). |
| GO:0004335 | 2 | Galactokinase activity | Molecular Function | Catalysis of the reaction: D-galactose + ATP = alpha-D-galactose 1-phosphate + ADP + 2 H(+). |
| GO:0004345 | 1 | Glucose-6-phosphate dehydrogenase activity | Molecular Function | Catalysis of the reaction: D-glucose 6-phosphate + NADP+ = D-glucono-1,5-lactone 6-phosphate + NADPH + H+. |
| GO:0004347 | 2 | Glucose-6-phosphate isomerase activity | Molecular Function | Catalysis of the reaction: D-glucose 6-phosphate = D-fructose 6-phosphate. |
| GO:0004364 | 5 | Glutathione transferase activity | Molecular Function | Catalysis of the reaction: R-X + glutathione = H-X + R-S-glutathione. R may be an aliphatic, aromatic or heterocyclic group; X may be a sulfate, nitrile or halide group. |
| GO:0004372 | 2 | Glycine hydroxymethyltransferase activity | Molecular Function | Catalysis of the reaction: 5,10-methylenetetrahydrofolate + glycine + H2O = tetrahydrofolate + L-serine. |
| GO:0004375 | 1 | Glycine dehydrogenase (decarboxylating) activity | Molecular Function | Catalysis of the reaction: glycine + lipoylprotein = S-aminomethyldihydrolipoylprotein + CO2. |
| GO:0004377 | 1 | GDP-Man:Man3GlcNAc2-PP-Dol alpha-1,2-mannosyltransferase activity | Molecular Function | Catalysis of the reaction: an alpha-D-Man-(1->3)-[alpha-D-Man-(1->6)]-beta-D-Man-(1->4)-beta-D-GlcNAc-(1->4)-D-GlcNAc-diphosphodolichol + 2 GDP-alpha-D-mannose = an alpha-D-Man-(1->2)-alpha-D-Man-(1->2)-alpha-D-Man-(1->3)-[alpha-D-Man-(1->6)]-beta-D-Man-(1->4)-beta-D-GlcNAc-(1->4)-D-GlcNAc-diphosphodolichol + 2 GDP + 2 H+. This reaction is the transfer of an alpha-D-mannosyl residue from GDP-mannose into lipid-linked oligosaccharide, forming an alpha-(1->2)-D-mannosyl-D-mannose linkage. |
| GO:0004378 | 1 | GDP-Man:Man1GlcNAc2-PP-Dol alpha-1,3-mannosyltransferase activity | Molecular Function | Catalysis of the reaction: beta-D-Man-(1->4)-beta-D-GlcNAc-(1->4)-D-GlcNAc-diphosphodolichol + GDP-alpha-D-mannose = alpha-D-Man-(1->3)-beta-D-Man-(1->4)-beta-D-GlcNAc-(1->4)-D-GlcNAc-diphosphodolichol + GDP + H+. This reaction is the transfer of an alpha-D-mannosyl residue from GDP-mannose into lipid-linked oligosaccharide, forming an alpha-(1->3)-D-mannosyl-D-mannose linkage. |
| GO:0004386 | 2 | Helicase activity | Molecular Function | Catalysis of the reaction: NTP + H2O = NDP + phosphate, to drive the unwinding of a DNA or RNA helix. |
| GO:0004398 | 1 | Histidine decarboxylase activity | Molecular Function | Catalysis of the reaction: L-histidine = histamine + CO2. |
| GO:0004402 | 1 | Histone acetyltransferase activity | Molecular Function | Catalysis of the reaction: acetyl-CoA + histone = CoA + acetyl-histone. |
| GO:0004424 | 1 | Imidazoleglycerol-phosphate dehydratase activity | Molecular Function | Catalysis of the reaction: D-erythro-1-(imidazol-4-yl)glycerol 3-phosphate = 3-(imidazol-4-yl)-2-oxopropyl phosphate + H(2)O. |
| GO:0004439 | 1 | Phosphatidylinositol-4,5-bisphosphate 5-phosphatase activity | Molecular Function | Catalysis of the reaction: 1-phosphatidyl-1D-myo-inositol 4,5-bisphosphate + H(2)O = 1-phosphatidyl-1D-myo-inositol 4-phosphate + phosphate. |
| GO:0004446 | 1 | Inositol-hexakisphosphate phosphatase activity | Molecular Function | Catalysis of the reaction: myo-inositol hexakisphosphate + H2O = myo-inositol pentakisphosphate (mixed isomers) + phosphate. |
| GO:0004449 | 1 | Isocitrate dehydrogenase (NAD+) activity | Molecular Function | Catalysis of the reaction: isocitrate + NAD+ = 2-oxoglutarate + CO2 + NADH + H+. |
| GO:0004450 | 1 | Isocitrate dehydrogenase (NADP+) activity | Molecular Function | Catalysis of the reaction: isocitrate + NADP+ = 2-oxoglutarate + CO2 + NADPH + H+. |
| GO:0004462 | 4 | Lactoylglutathione lyase activity | Molecular Function | Catalysis of the reaction: (R)-S-lactoylglutathione = glutathione + methylglyoxal. |
| GO:0004467 | 1 | Long-chain fatty acid-CoA ligase activity | Molecular Function | Catalysis of the reaction: ATP + a long-chain fatty acid + CoA = AMP + diphosphate + an acyl-CoA; a long-chain fatty acid is a fatty acid with a chain length between C13 and C22. |
| GO:0004471 | 1 | Malate dehydrogenase (decarboxylating) (NAD+) activity | Molecular Function | Catalysis of the reaction: (S)-malate + NAD+ = pyruvate + CO2 + NADH + H+. |
| GO:0004477 | 1 | Methenyltetrahydrofolate cyclohydrolase activity | Molecular Function | Catalysis of the reaction: 5,10-methenyltetrahydrofolate + H2O = 10-formyltetrahydrofolate. |
| GO:0004488 | 1 | Methylenetetrahydrofolate dehydrogenase (NADP+) activity | Molecular Function | Catalysis of the reaction: 5,10-methylenetetrahydrofolate + NADP(+) = 5,10-methenyltetrahydrofolate + NADPH. |
| GO:0004491 | 2 | Methylmalonate-semialdehyde dehydrogenase (acylating) activity | Molecular Function | Catalysis of the reaction: 2-methyl-3-oxopropanoate + CoA + NAD+ = propanoyl-CoA + CO2 + NADH + H+. |
| GO:0004497 | 5 | Monooxygenase activity | Molecular Function | Catalysis of the incorporation of one atom from molecular oxygen into a compound and the reduction of the other atom of oxygen to water. |
| GO:0004499 | 3 | N,N-dimethylaniline monooxygenase activity | Molecular Function | Catalysis of the reaction: N,N-dimethylaniline + NADPH + H+ + O2 = N,N-dimethylaniline N-oxide + NADP+ + H2O. |
| GO:0004506 | 2 | Squalene monooxygenase activity | Molecular Function | Catalysis of the reaction: H(+) + NADPH + O(2) + squalene = (S)-2,3-epoxysqualene + H(2)O + NADP(+). |
| GO:0004535 | 1 | Poly(A)-specific ribonuclease activity | Molecular Function | Catalysis of the exonucleolytic cleavage of poly(A) to 5'-AMP. |
| GO:0004536 | 1 | Deoxyribonuclease activity | Molecular Function | Catalysis of the hydrolysis of ester linkages within deoxyribonucleic acid. |
| GO:0004553 | 4 | Hydrolase activity, hydrolyzing O-glycosyl compounds | Molecular Function | Catalysis of the hydrolysis of any O-glycosyl bond. |
| GO:0004565 | 1 | Beta-galactosidase activity | Molecular Function | Catalysis of the hydrolysis of terminal, non-reducing beta-D-galactose residues in beta-D-galactosides. |
| GO:0004577 | 1 | N-acetylglucosaminyldiphosphodolichol N-acetylglucosaminyltransferase activity | Molecular Function | Catalysis of the reaction: UDP-N-acetyl-D-glucosamine + N-acetyl-D-glucosaminyl-diphosphodolichol = UDP + N,N''-diacetylchitobiosyldiphosphodolichol. |
| GO:0004579 | 1 | Dolichyl-diphosphooligosaccharide-protein glycotransferase activity | Molecular Function | Catalysis of the reaction: dolichyl diphosphooligosaccharide + protein L-asparagine = dolichyl diphosphate + a glycoprotein with the oligosaccharide chain attached by glycosylamine linkage to protein L-asparagine. |
| GO:0004601 | 7 | Peroxidase activity | Molecular Function | Catalysis of the reaction: donor + hydrogen peroxide = oxidized donor + 2 H2O. |
| GO:0004614 | 1 | Phosphoglucomutase activity | Molecular Function | Catalysis of the reaction: alpha-D-glucose 1-phosphate = alpha-D-glucose 6-phosphate. |
| GO:0004634 | 1 | Phosphopyruvate hydratase activity | Molecular Function | Catalysis of the reaction: 2-phospho-D-glycerate = phosphoenolpyruvate + H2O. |
| GO:0004650 | 2 | Polygalacturonase activity | Molecular Function | Catalysis of the random hydrolysis of (1->4)-alpha-D-galactosiduronic linkages in pectate and other galacturonans. |
| GO:0004655 | 2 | Porphobilinogen synthase activity | Molecular Function | Catalysis of the reaction: 2 5-aminolevulinate = 2 H(2)O + H(+) + porphobilinogen. |
| GO:0004665 | 1 | Prephenate dehydrogenase (NADP+) activity | Molecular Function | Catalysis of the reaction: NADP(+) + prephenate = (4-hydroxyphenyl)pyruvate + CO(2) + NADPH. |
| GO:0004672 | 31 | Protein kinase activity | Molecular Function | Catalysis of the phosphorylation of an amino acid residue in a protein, usually according to the reaction: a protein + ATP = a phosphoprotein + ADP. |
| GO:0004674 | 22 | Protein serine/threonine kinase activity | Molecular Function | Catalysis of an oxidation-reduction (redox) reaction, a reversible chemical reaction in which the oxidation state of an atom or atoms within a molecule is altered. One substrate acts as a hydrogen or electron donor and becomes oxidized, while the other acts as hydrogen or electron acceptor and becomes reduced |
| GO:0004691 | 1 | cAMP-dependent protein kinase activity | Molecular Function | Catalysis of the reaction: ATP + a protein = ADP + a phosphoprotein. This reaction requires the presence of cAMP. |
| GO:0004702 | 2 | Signal transducer, downstream of receptor, with serine/threonine kinase activity | Molecular Function | Conveys a signal from an upstream receptor or intracellular signal transducer by catalysis of the reaction: ATP protein serine = ADP + protein serine phosphate, and ATP + protein threonine = ADP + protein threonine phosphate. |
| GO:0004713 | 2 | Protein tyrosine kinase activity | Molecular Function | Catalysis of the reaction: ATP + a protein tyrosine = ADP + protein tyrosine phosphate. |
| GO:0004714 | 2 | Transmembrane receptor protein tyrosine kinase activity | Molecular Function | Combining with a signal and transmitting the signal from one side of the membrane to the other to initiate a change in cell activity by catalysis of the reaction: ATP + a protein-L-tyrosine = ADP + a protein-L-tyrosine phosphate. |
| GO:0004715 | 4 | Non-membrane spanning protein tyrosine kinase activity | Molecular Function | Catalysis of the reaction: ATP + protein L-tyrosine = ADP + protein L-tyrosine phosphate by a non-membrane spanning protein. |
| GO:0004721 | 2 | Phosphoprotein phosphatase activity | Molecular Function | Catalysis of the reaction: a phosphoprotein + H2O = a protein + phosphate. Together with protein kinases, these enzymes control the state of phosphorylation of cell proteins and thereby provide an important mechanism for regulating cellular activity. |
| GO:0004722 | 4 | Protein serine/threonine phosphatase activity | Molecular Function | Catalysis of the reaction: protein serine phosphate + H2O = protein serine + phosphate, and protein threonine phosphate + H2O = protein threonine + phosphate. |
| GO:0004725 | 3 | Protein tyrosine phosphatase activity | Molecular Function | Catalysis of the reaction: protein tyrosine phosphate + H2O = protein tyrosine + phosphate. |
| GO:0004743 | 1 | Pyruvate kinase activity | Molecular Function | Catalysis of the reaction: ATP + pyruvate = ADP + phosphoenolpyruvate. |
| GO:0004748 | 1 | Ribonucleoside-diphosphate reductase activity, thioredoxin disulfide as acceptor | Molecular Function | Catalysis of the reaction: 2'-deoxyribonucleoside diphosphate + thioredoxin disulfide + H2O = ribonucleoside diphosphate + thioredoxin. Thioredoxin disulfide is the oxidized form of thioredoxin. |
| GO:0004781 | 1 | Sulfate adenylyltransferase (ATP) activity | Molecular Function | Catalysis of the reaction: ATP + sulfate = diphosphate + adenylylsulfate. |
| GO:0004784 | 1 | Superoxide dismutase activity | Molecular Function | Catalysis of the reaction: 2 superoxide + 2 H+ = O2 + hydrogen peroxide. |
| GO:0004812 | 1 | Aminoacyl-tRNA ligase activity | Molecular Function | Catalysis of the formation of aminoacyl-tRNA from ATP, amino acid, and tRNA with the release of diphosphate and AMP. |
| GO:0004842 | 7 | Ubiquitin-protein transferase activity | Molecular Function | Catalysis of the transfer of ubiquitin from one protein to another via the reaction X-Ub + Y --> Y-Ub + X, where both X-Ub and Y-Ub are covalent linkages. |
| GO:0004853 | 2 | Uroporphyrinogen decarboxylase activity | Molecular Function | Catalysis of the reaction: uroporphyrinogen-III = coproporphyrinogen + 4 CO2. |
| GO:0004857 | 3 | Enzyme inhibitor activity | Molecular Function | Binds to and stops, prevents or reduces the activity of an enzyme. |
| GO:0004871 | 2 | Signal transducer activity | Molecular Function | Conveys a signal across a cell to trigger a change in cell function or state. A signal is a physical entity or change in state that is used to transfer information in order to trigger a response. |
| GO:0004872 | 1 | Receptor activity | Molecular Function | Combining with an extracellular or intracellular messenger to initiate a change in cell activity. |
| GO:0004970 | 2 | Ionotropic glutamate receptor activity | Molecular Function | Catalysis of the transmembrane transfer of an ion by a channel that opens when glutamate has been bound by the channel complex or one of its constituent parts. |
| GO:0005096 | 3 | GTPase activator activity | Molecular Function | Binds to and increases the activity of a GTPase, an enzyme that catalyzes the hydrolysis of GTP. |
| GO:0005198 | 1 | Structural molecule activity | Molecular Function | The action of a molecule that contributes to the structural integrity of a complex or its assembly within or outside a cell. |
| GO:0005215 | 6 | Transporter activity | Molecular Function | Enables the directed movement of substances (such as macromolecules, small molecules, ions) into, out of or within a cell, or between cells. |
| GO:0005216 | 1 | Ion channel activity | Molecular Function | Enables the facilitated diffusion of an ion (by an energy-independent process) by passage through a transmembrane aqueous pore or channel without evidence for a carrier-mediated mechanism. May be either selective (it enables passage of a specific ion only) or non-selective (it enables passage of two or more ions of same charge but different size). |
| GO:0005249 | 1 | Voltage-gated potassium channel activity | Molecular Function | Enables the transmembrane transfer of a potassium ion by a voltage-gated channel. A voltage-gated channel is a channel whose open state is dependent on the voltage across the membrane in which it is embedded. |
| GO:0005267 | 1 | Potassium channel activity | Molecular Function | Enables the facilitated diffusion of a potassium ion (by an energy-independent process) involving passage through a transmembrane aqueous pore or channel without evidence for a carrier-mediated mechanism. |
| GO:0005315 | 1 | Inorganic phosphate transmembrane transporter activity | Molecular Function | Enables the transfer of a inorganic phosphate from one side of a membrane to the other, up its concentration gradient. The transporter binds the solute and undergoes a series of conformational changes. Transport works equally well in either direction and is driven by a chemiosmotic source of energy. Chemiosmotic sources of energy include uniport, symport or antiport. |
| GO:0005319 | 1 | Lipid transporter activity | Molecular Function | Enables the directed movement of lipids into, out of or within a cell, or between cells. |
| GO:0005381 | 1 | Iron ion transmembrane transporter activity | Molecular Function | Catalysis of the transfer of iron (Fe) ions from one side of a membrane to the other. |
| GO:0005384 | 1 | Manganese ion transmembrane transporter activity | Molecular Function | Catalysis of the transfer of manganese (Mn) ions from one side of a membrane to the other. |
| GO:0005432 | 1 | Calcium:sodium antiporter activity | Molecular Function | Catalysis of the transfer of a solute or solutes from one side of a membrane to the other according to the reaction: Ca2+(in) + Na+(out) = Ca2+(out) + Na+(in). |
| GO:0005451 | 1 | Monovalent cation:proton antiporter activity | Molecular Function | Catalysis of the transfer of a solute or solutes from one side of a membrane to the other according to the reaction: monovalent cation(out) + H+(in) = monovalent cation(in) + H+(out). |
| GO:0005452 | 1 | Inorganic anion exchanger activity | Molecular Function | Catalysis of the transfer of a solute or solutes from one side of a membrane to the other according to the reaction: inorganic anion A(out) + inorganic anion B(in) = inorganic anion A(in) + inorganic anion B(out). |
| GO:0005484 | 2 | SNAP receptor activity | Molecular Function | Acting as a marker to identify a membrane and interacting selectively with one or more SNAREs on another membrane to mediate membrane fusion. |
| GO:0005488 | 18 | Binding | Molecular Function | The selective, non-covalent, often stoichiometric, interaction of a molecule with one or more specific sites on another molecule. |
| GO:0005506 | 28 | Iron ion binding | Molecular Function | Interacting selectively and non-covalently with iron (Fe) ions. |
| GO:0005507 | 3 | Copper ion binding | Molecular Function | Interacting selectively and non-covalently with copper (Cu) ions. |
| GO:0005509 | 11 | Calcium ion binding | Molecular Function | Interacting selectively and non-covalently with calcium ions (Ca2+). |
| GO:0005515 | 78 | Protein binding | Molecular Function | Interacting selectively and non-covalently with any protein or protein complex (a complex of two or more proteins that may include other nonprotein molecules). |
| GO:0005516 | 1 | Calmodulin binding | Molecular Function | Interacting selectively and non-covalently with calmodulin, a calcium-binding protein with many roles, both in the calcium-bound and calcium-free states. |
| GO:0005524 | 111 | ATP binding | Molecular Function | Interacting selectively and non-covalently with ATP, adenosine 5'-triphosphate, a universally important coenzyme and enzyme regulator. |
| GO:0005525 | 5 | GTP binding | Molecular Function | Interacting selectively and non-covalently with GTP, guanosine triphosphate. |
| GO:0005528 | 2 | FK506 binding | Molecular Function | Interacting selectively and non-covalently with the 23-membered macrolide lactone FK506. |
| GO:0005544 | 2 | Calcium-dependent phospholipid binding | Molecular Function | Interacting selectively and non-covalently with phospholipids, a class of lipids containing phosphoric acid as a mono- or diester, in the presence of calcium. |
| GO:0005576 | 14 | Extracellular region | Cellular Component | The space external to the outermost structure of a cell. For cells without external protective or external encapsulating structures this refers to space outside of the plasma membrane. This term covers the host cell environment outside an intracellular parasite. |
| GO:0005615 | 5 | Extracellular space | Cellular Component | That part of a multicellular organism outside the cells proper, usually taken to be outside the plasma membranes, and occupied by fluid. |
| GO:0005618 | 17 | Cell wall | Cellular Component | The rigid or semi-rigid envelope lying outside the cell membrane of plant, fungal, most prokaryotic cells and some protozoan parasites, maintaining their shape and protecting them from osmotic lysis. In plants it is made of cellulose and, often, lignin; in fungi it is composed largely of polysaccharides; in bacteria it is composed of peptidoglycan; in protozoan parasites such as Giardia species, it's made of carbohydrates and proteins. |
| GO:0005622 | 7 | Intracellular | Cellular Component | The living contents of a cell; the matter contained within (but not including) the plasma membrane, usually taken to exclude large vacuoles and masses of secretory or ingested material. In eukaryotes it includes the nucleus and cytoplasm. |
| GO:0005623 | 2 | Cell | Cellular Component | The basic structural and functional unit of all organisms. Includes the plasma membrane and any external encapsulating structures such as the cell wall and cell envelope. |
| GO:0005634 | 44 | Nucleus | Cellular Component | A membrane-bounded organelle of eukaryotic cells in which chromosomes are housed and replicated. In most cells, the nucleus contains all of the cell's chromosomes except the organellar chromosomes, and is the site of RNA synthesis and processing. In some species, or in specialized cell types, RNA metabolism or DNA replication may be absent. |
| GO:0005654 | 9 | Nucleoplasm | Cellular Component | That part of the nuclear content other than the chromosomes or the nucleolus. |
| GO:0005666 | 2 | DNA-directed RNA polymerase III complex | Cellular Component | RNA polymerase III, one of three nuclear DNA-directed RNA polymerases found in all eukaryotes, is a multisubunit complex; typically it produces 5S rRNA, tRNAs and some of the small nuclear RNAs. Two large subunits comprise the most conserved portion including the catalytic site and share similarity with other eukaryotic and bacterial multisubunit RNA polymerases. The remainder of the complex is composed of smaller subunits (generally ten or more), some of which are also found in RNA polymerase I and others of which are also found in RNA polymerases I and II. Although the core is competent to mediate ribonucleic acid synthesis, it requires additional factors to select the appropriate template. |
| GO:0005677 | 1 | Chromatin silencing complex | Cellular Component | Any protein complex that mediates changes in chromatin structure that result in transcriptional silencing. |
| GO:0005685 | 1 | U1 snRNP | Cellular Component | A ribonucleoprotein complex that contains small nuclear RNA U1. |
| GO:0005732 | 1 | Small nucleolar ribonucleoprotein complex | Cellular Component | A ribonucleoprotein complex that contains an RNA molecule of the small nucleolar RNA (snoRNA) family and associated proteins. Most are involved in a step of processing of rRNA: cleavage, 2'-O-methylation, or pseudouridylation. The majority, though not all, fall into one of two classes, box C/D type or box H/ACA type. |
| GO:0005737 | 55 | Cytoplasm | Cellular Component | All of the contents of a cell excluding the plasma membrane and nucleus, but including other subcellular structures. |
| GO:0005739 | 31 | Mitochondrion | Cellular Component | A semiautonomous, self replicating organelle that occurs in varying numbers, shapes, and sizes in the cytoplasm of virtually all eukaryotic cells. It is notably the site of tissue respiration. |
| GO:0005743 | 12 | Mitochondrial inner membrane | Cellular Component | The inner, i.e. lumen-facing, lipid bilayer of the mitochondrial envelope. It is highly folded to form cristae. |
| GO:0005759 | 1 | Mitochondrial matrix | Cellular Component | The gel-like material, with considerable fine structure, that lies in the matrix space, or lumen, of a mitochondrion. It contains the enzymes of the tricarboxylic acid cycle and, in some organisms, the enzymes concerned with fatty acid oxidation. |
| GO:0005761 | 1 | Mitochondrial ribosome | Cellular Component | A ribosome found in the mitochondrion of a eukaryotic cell; contains a characteristic set of proteins distinct from those of cytosolic ribosomes. |
| GO:0005763 | 3 | Mitochondrial small ribosomal subunit | Cellular Component | The smaller of the two subunits of a mitochondrial ribosome. |
| GO:0005764 | 4 | Lysosome | Cellular Component | A small lytic vacuole that has cell cycle-independent morphology and is found in most animal cells and that contains a variety of hydrolases, most of which have their maximal activities in the pH range 5-6. The contained enzymes display latency if properly isolated. About 40 different lysosomal hydrolases are known and lysosomes have a great variety of morphologies and functions. |
| GO:0005765 | 3 | Lysosomal membrane | Cellular Component | The lipid bilayer surrounding the lysosome and separating its contents from the cell cytoplasm. |
| GO:0005768 | 2 | Endosome | Cellular Component | A vacuole to which materials ingested by endocytosis are delivered. |
| GO:0005773 | 4 | Vacuole | Cellular Component | A closed structure, found only in eukaryotic cells, that is completely surrounded by unit membrane and contains liquid material. Cells contain one or several vacuoles, that may have different functions from each other. Vacuoles have a diverse array of functions. They can act as a storage organelle for nutrients or waste products, as a degradative compartment, as a cost-effective way of increasing cell size, and as a homeostatic regulator controlling both turgor pressure and pH of the cytosol. |
| GO:0005774 | 5 | Vacuolar membrane | Cellular Component | The lipid bilayer surrounding the vacuole and separating its contents from the cytoplasm of the cell. |
| GO:0005777 | 2 | Peroxisome | Cellular Component | A small organelle enclosed by a single membrane, and found in most eukaryotic cells. Contains peroxidases and other enzymes involved in a variety of metabolic processes including free radical detoxification, lipid catabolism and biosynthesis, and hydrogen peroxide metabolism. |
| GO:0005783 | 4 | Endoplasmic reticulum | Cellular Component | The irregular network of unit membranes, visible only by electron microscopy, that occurs in the cytoplasm of many eukaryotic cells. The membranes form a complex meshwork of tubular channels, which are often expanded into slitlike cavities called cisternae. The ER takes two forms, rough (or granular), with ribosomes adhering to the outer surface, and smooth (with no ribosomes attached). |
| GO:0005786 | 1 | Signal recognition particle, endoplasmic reticulum targeting | Cellular Component | A ribonucleoprotein particle of 325 kDa composed of a 7S (300 nucleotide) RNA molecule and a complex of six different polypeptides. This binds both to the N-terminal signal peptide for proteins destined for the endoplasmic reticulum as they emerge from the large ribosomal subunit and also to the ribosome. This binding arrests further translation thereby preventing the proteins from being released into the cytosol. The SRP-ribosome complex then diffuses to the endoplasmic reticulum where it is bound to the signal recognition particle receptor, which allows resumption of protein synthesis and facilitates the passage of the growing polypeptide chain through the translocon. Through a process involving GTP hydrolysis, the SRP-SRP receptor complex dissociates and SRP returns to the cytosol. Of the six polypeptides of SRP the 54 kDa subunit (SRP54) is the central player. It contains an N-terminal GTPase domain and a C-terminal domain that binds directly to the signal peptide and the SRP RNA. Examples of this component are found in Mus musculus, Saccharomyces cerevisiae and Arabidopsis thaliana. |
| GO:0005789 | 4 | Endoplasmic reticulum membrane | Cellular Component | The lipid bilayer surrounding the endoplasmic reticulum. |
| GO:0005794 | 7 | Golgi apparatus | Cellular Component | A compound membranous cytoplasmic organelle of eukaryotic cells, consisting of flattened, ribosome-free vesicles arranged in a more or less regular stack. The Golgi apparatus differs from the endoplasmic reticulum in often having slightly thicker membranes, appearing in sections as a characteristic shallow semicircle so that the convex side (cis or entry face) abuts the endoplasmic reticulum, secretory vesicles emerging from the concave side (trans or exit face). In vertebrate cells there is usually one such organelle, while in invertebrates and plants, where they are known usually as dictyosomes, there may be several scattered in the cytoplasm. The Golgi apparatus processes proteins produced on the ribosomes of the rough endoplasmic reticulum; such processing includes modification of the core oligosaccharides of glycoproteins, and the sorting and packaging of proteins for transport to a variety of cellular locations. Three different regions of the Golgi are now recognized both in terms of structure and function: cis, in the vicinity of the cis face, trans, in the vicinity of the trans face, and medial, lying between the cis and trans regions. |
| GO:0005802 | 1 | Trans-Golgi network | Cellular Component | The network of interconnected tubular and cisternal structures located within the Golgi apparatus on the side distal to the endoplasmic reticulum, from which secretory vesicles emerge. The trans-Golgi network is important in the later stages of protein secretion where it is thought to play a key role in the sorting and targeting of secreted proteins to the correct destination. |
| GO:0005813 | 1 | Centrosome | Cellular Component | A structure comprised of a core structure (in most organisms, a pair of centrioles) and peripheral material from which a microtubule-based structure, such as a spindle apparatus, is organized. Centrosomes occur close to the nucleus during interphase in many eukaryotic cells, though in animal cells it changes continually during the cell-division cycle. |
| GO:0005829 | 20 | Cytosol | Cellular Component | The part of the cytoplasm that does not contain organelles but which does contain other particulate matter, such as protein complexes. |
| GO:0005834 | 1 | Heterotrimeric G-protein complex | Cellular Component | Any of a family of heterotrimeric GTP-binding and hydrolyzing proteins; they belong to a superfamily of GTPases that includes monomeric proteins such as EF-Tu and RAS. Heterotrimeric G-proteins consist of three subunits; the alpha subunit contains the guanine nucleotide binding site and possesses GTPase activity; the beta and gamma subunits are tightly associated and function as a beta-gamma heterodimer; extrinsic plasma membrane proteins (cytoplasmic face) that function as a complex to transduce signals from G-protein coupled receptors to an effector protein. |
| GO:0005840 | 9 | Ribosome | Cellular Component | An intracellular organelle, about 200 A in diameter, consisting of RNA and protein. It is the site of protein biosynthesis resulting from translation of messenger RNA (mRNA). It consists of two subunits, one large and one small, each containing only protein and RNA. Both the ribosome and its subunits are characterized by their sedimentation coefficients, expressed in Svedberg units (symbol: S). Hence, the prokaryotic ribosome (70S) comprises a large (50S) subunit and a small (30S) subunit, while the eukaryotic ribosome (80S) comprises a large (60S) subunit and a small (40S) subunit. Two sites on the ribosomal large subunit are involved in translation, namely the aminoacyl site (A site) and peptidyl site (P site). Ribosomes from prokaryotes, eukaryotes, mitochondria, and chloroplasts have characteristically distinct ribosomal proteins. |
| GO:0005849 | 1 | mRNA cleavage factor complex | Cellular Component | Any macromolecular complex involved in cleavage or polyadenylation of mRNA molecules. |
| GO:0005852 | 1 | Eukaryotic translation initiation factor 3 complex | Cellular Component | A complex of several polypeptides that plays at least two important roles in protein synthesis: First, eIF3 binds to the 40S ribosome and facilitates loading of the Met-tRNA/eIF2.GTP ternary complex to form the 43S preinitiation complex. Subsequently, eIF3 apparently assists eIF4 in recruiting mRNAs to the 43S complex. The eIF3 complex contains five conserved core subunits, and may contain several additional proteins; the non-core subunits are thought to mediate association of the complex with specific sets of mRNAs. |
| GO:0005871 | 2 | Kinesin complex | Cellular Component | Any complex that includes a dimer of molecules from the kinesin superfamily, a group of related proteins that contain an extended region of predicted alpha-helical coiled coil in the main chain that likely produces dimerization. The native complexes of several kinesin family members have also been shown to contain additional peptides, often designated light chains as all of the noncatalytic subunits that are currently known are smaller than the chain that contains the motor unit. Kinesin complexes generally possess a force-generating enzymatic activity, or motor, which converts the free energy of the gamma phosphate bond of ATP into mechanical work. |
| GO:0005874 | 1 | Microtubule | Cellular Component | Any of the long, generally straight, hollow tubes of internal diameter 12-15 nm and external diameter 24 nm found in a wide variety of eukaryotic cells; each consists (usually) of 13 protofilaments of polymeric tubulin, staggered in such a manner that the tubulin monomers are arranged in a helical pattern on the microtubular surface, and with the alpha/beta axes of the tubulin subunits parallel to the long axis of the tubule; exist in equilibrium with pool of tubulin monomers and can be rapidly assembled or disassembled in response to physiological stimuli; concerned with force generation, e.g. in the spindle. |
| GO:0005886 | 37 | Plasma membrane | Cellular Component | The membrane surrounding a cell that separates the cell from its external environment. It consists of a phospholipid bilayer and associated proteins. |
| GO:0005887 | 6 | Integral component of plasma membrane | Cellular Component | The component of the plasma membrane consisting of the gene products and protein complexes having at least some part of their peptide sequence embedded in the hydrophobic region of the membrane. |
| GO:0005960 | 1 | Glycine cleavage complex | Cellular Component | A protein complex that catalyzes the reversible oxidation of glycine. In E. coli, it has four components: dihydrolipoamide dehydrogenase, glycine dehydrogenase (decarboxylating), lipoyl-GcvH-protein and aminomethyltransferase, also known as L, P, H, and T. |
| GO:0005971 | 1 | Ribonucleoside-diphosphate reductase complex | Cellular Component | An enzyme complex composed of 2-4 or more subunits, which usually contains nonheme iron and requires ATP for catalysis. Catalyzes the formation of 2'-deoxyribonucleoside diphosphate from ribonucleoside diphosphate, using either thioredoxin disulfide or glutaredoxin disulfide as an acceptor. |
| GO:0005975 | 10 | Carbohydrate metabolic process | Biological Process | The chemical reactions and pathways involving carbohydrates, any of a group of organic compounds based of the general formula Cx(H2O)y. Includes the formation of carbohydrate derivatives by the addition of a carbohydrate residue to another molecule. |
| GO:0005977 | 1 | Glycogen metabolic process | Biological Process | The chemical reactions and pathways involving glycogen, a polydisperse, highly branched glucan composed of chains of D-glucose residues in alpha-(1->4) glycosidic linkage, joined together by alpha-(1->6) glycosidic linkages. |
| GO:0005978 | 2 | Glycogen biosynthetic process | Biological Process | The chemical reactions and pathways resulting in the formation of glycogen, a polydisperse, highly branched glucan composed of chains of D-glucose residues. |
| GO:0006006 | 2 | Glucose metabolic process | Biological Process | The chemical reactions and pathways involving glucose, the aldohexose gluco-hexose. D-glucose is dextrorotatory and is sometimes known as dextrose; it is an important source of energy for living organisms and is found free as well as combined in homo- and hetero-oligosaccharides and polysaccharides. |
| GO:0006012 | 2 | Galactose metabolic process | Biological Process | The chemical reactions and pathways involving galactose, the aldohexose galacto-hexose. D-galactose is widely distributed in combined form in plants, animals and microorganisms as a constituent of oligo- and polysaccharides; it also occurs in galactolipids and as its glucoside in lactose and melibiose. |
| GO:0006081 | 2 | Cellular aldehyde metabolic process | Biological Process | The chemical reactions and pathways involving aldehydes, any organic compound with the formula R-CH=O, as carried out by individual cells. |
| GO:0006090 | 1 | Pyruvate metabolic process | Biological Process | The chemical reactions and pathways involving pyruvate, 2-oxopropanoate. |
| GO:0006094 | 2 | Gluconeogenesis | Biological Process | The formation of glucose from noncarbohydrate precursors, such as pyruvate, amino acids and glycerol. |
| GO:0006096 | 5 | Glycolytic process | Biological Process | The chemical reactions and pathways resulting in the breakdown of a carbohydrate into pyruvate, with the concomitant production of a small amount of ATP and the reduction of NAD(P) to NAD(P)H. Glycolysis begins with the metabolism of a carbohydrate to generate products that can enter the pathway and ends with the production of pyruvate. Pyruvate may be converted to acetyl-coenzyme A, ethanol, lactate, or other small molecules. |
| GO:0006098 | 1 | Pentose-phosphate shunt | Biological Process | The glucose-6-phosphate catabolic process in which, coupled to NADPH synthesis, glucose-6-P is oxidized with the formation of carbon dioxide (CO2) and ribulose 5-phosphate; ribulose 5-P then enters a series of reactions interconverting sugar phosphates. The pentose phosphate pathway is a major source of reducing equivalents for biosynthesis reactions and is also important for the conversion of hexoses to pentoses. |
| GO:0006099 | 3 | Tricarboxylic acid cycle | Biological Process | A nearly universal metabolic pathway in which the acetyl group of acetyl coenzyme A is effectively oxidized to two CO2 and four pairs of electrons are transferred to coenzymes. The acetyl group combines with oxaloacetate to form citrate, which undergoes successive transformations to isocitrate, 2-oxoglutarate, succinyl-CoA, succinate, fumarate, malate, and oxaloacetate again, thus completing the cycle. In eukaryotes the tricarboxylic acid is confined to the mitochondria. See also glyoxylate cycle. |
| GO:0006102 | 1 | Isocitrate metabolic process | Biological Process | The chemical reactions and pathways involving isocitrate, the anion of isocitric acid, 1-hydroxy-1,2,3-propanetricarboxylic acid. Isocitrate is an important intermediate in the TCA cycle and the glycoxylate cycle. |
| GO:0006120 | 2 | Mitochondrial electron transport, NADH to ubiquinone | Biological Process | The transfer of electrons from NADH to ubiquinone that occurs during oxidative phosphorylation, mediated by the multisubunit enzyme known as complex I. |
| GO:0006139 | 3 | Nucleobase-containing compound metabolic process | Biological Process | Any cellular metabolic process involving nucleobases, nucleosides, nucleotides and nucleic acids. |
| GO:0006168 | 1 | Adenine salvage | Biological Process | Any process that generates adenine, 6-aminopurine, from derivatives of it without de novo synthesis. |
| GO:0006203 | 1 | dGTP catabolic process | Biological Process | The chemical reactions and pathways resulting in the breakdown of dGTP, guanosine triphosphate. |
| GO:0006226 | 1 | dUMP biosynthetic process | Biological Process | The chemical reactions and pathways resulting in the formation of dUMP, deoxyuridine monophosphate (2'-deoxyuridine 5'-phosphate). |
| GO:0006259 | 1 | DNA metabolic process | Biological Process | Any cellular metabolic process involving deoxyribonucleic acid. This is one of the two main types of nucleic acid, consisting of a long, unbranched macromolecule formed from one, or more commonly, two, strands of linked deoxyribonucleotides. |
| GO:0006260 | 4 | DNA replication | Biological Process | The cellular metabolic process in which a cell duplicates one or more molecules of DNA. DNA replication begins when specific sequences, known as origins of replication, are recognized and bound by initiation proteins, and ends when the original DNA molecule has been completely duplicated and the copies topologically separated. The unit of replication usually corresponds to the genome of the cell, an organelle, or a virus. The template for replication can either be an existing DNA molecule or RNA. |
| GO:0006273 | 2 | Lagging strand elongation | Biological Process | The synthesis of DNA from a template strand in a net 3' to 5' direction. Lagging strand DNA elongation proceeds by discontinuous synthesis of short stretches of DNA, known as Okazaki fragments, from RNA primers; these fragments are then joined by DNA ligase. Although each segment of nascent DNA is synthesized in the 5' to 3' direction, the overall direction of lagging strand synthesis is 3' to 5', mirroring the progress of the replication fork. |
| GO:0006281 | 2 | DNA repair | Biological Process | The process of restoring DNA after damage. Genomes are subject to damage by chemical and physical agents in the environment (e.g. UV and ionizing radiations, chemical mutagens, fungal and bacterial toxins, etc.) and by free radicals or alkylating agents endogenously generated in metabolism. DNA is also damaged because of errors during its replication. A variety of different DNA repair pathways have been reported that include direct reversal, base excision repair, nucleotide excision repair, photoreactivation, bypass, double-strand break repair pathway, and mismatch repair pathway. |
| GO:0006289 | 1 | Nucleotide-excision repair | Biological Process | A DNA repair process in which a small region of the strand surrounding the damage is removed from the DNA helix as an oligonucleotide. The small gap left in the DNA helix is filled in by the sequential action of DNA polymerase and DNA ligase. Nucleotide excision repair recognizes a wide range of substrates, including damage caused by UV irradiation (pyrimidine dimers and 6-4 photoproducts) and chemicals (intrastrand cross-links and bulky adducts). |
| GO:0006302 | 1 | Double-strand break repair | Biological Process | The repair of double-strand breaks in DNA via homologous and nonhomologous mechanisms to reform a continuous DNA helix. |
| GO:0006303 | 2 | Double-strand break repair via nonhomologous end joining | Biological Process | The repair of a double-strand break in DNA in which the two broken ends are rejoined with little or no sequence complementarity. Information at the DNA ends may be lost due to the modification of broken DNA ends. This term covers instances of separate pathways, called classical (or canonical) and alternative nonhomologous end joining (C-NHEJ and A-NHEJ). These in turn may further branch into sub-pathways, but evidence is still unclear. |
| GO:0006308 | 1 | DNA catabolic process | Biological Process | The cellular DNA metabolic process resulting in the breakdown of DNA, deoxyribonucleic acid, one of the two main types of nucleic acid, consisting of a long unbranched macromolecule formed from one or two strands of linked deoxyribonucleotides, the 3'-phosphate group of each constituent deoxyribonucleotide being joined in 3',5'-phosphodiester linkage to the 5'-hydroxyl group of the deoxyribose moiety of the next one. |
| GO:0006310 | 2 | DNA recombination | Biological Process | Any process in which a new genotype is formed by reassortment of genes resulting in gene combinations different from those that were present in the parents. In eukaryotes genetic recombination can occur by chromosome assortment, intrachromosomal recombination, or nonreciprocal interchromosomal recombination. Intrachromosomal recombination occurs by crossing over. In bacteria it may occur by genetic transformation, conjugation, transduction, or F-duction. |
| GO:0006334 | 1 | Nucleosome assembly | Biological Process | The aggregation, arrangement and bonding together of a nucleosome, the beadlike structural units of eukaryotic chromatin composed of histones and DNA. |
| GO:0006342 | 1 | Chromatin silencing | Biological Process | Repression of transcription by altering the structure of chromatin, e.g. by conversion of large regions of DNA into an inaccessible state often called heterochromatin. |
| GO:0006351 | 5 | Transcription, DNA-templated | Biological Process | The cellular synthesis of RNA on a template of DNA. |
| GO:0006352 | 1 | DNA-templated transcription, initiation | Biological Process | Any process involved in the assembly of the RNA polymerase preinitiation complex (PIC) at the core promoter region of a DNA template, resulting in the subsequent synthesis of RNA from that promoter. The initiation phase includes PIC assembly and the formation of the first few bonds in the RNA chain, including abortive initiation, which occurs when the first few nucleotides are repeatedly synthesized and then released. The initiation phase ends just before and does not include promoter clearance, or release, which is the transition between the initiation and elongation phases of transcription. |
| GO:0006355 | 213 | Regulation of transcription, DNA-templated | Biological Process | Any process that modulates the frequency, rate or extent of cellular DNA-templated transcription. |
| GO:0006357 | 2 | Regulation of transcription from RNA polymerase II promoter | Biological Process | Any process that modulates the frequency, rate or extent of transcription from an RNA polymerase II promoter. |
| GO:0006359 | 1 | Regulation of transcription from RNA polymerase III promoter | Biological Process | Any process that modulates the frequency, rate or extent of transcription from an RNA ploymerase III promoter. |
| GO:0006364 | 1 | rRNA processing | Biological Process | Any process involved in the conversion of a primary ribosomal RNA (rRNA) transcript into one or more mature rRNA molecules. |
| GO:0006376 | 1 | mRNA splice site selection | Biological Process | Selection of a splice site by components of the assembling spliceosome. |
| GO:0006378 | 1 | mRNA polyadenylation | Biological Process | The enzymatic addition of a sequence of 40-200 adenylyl residues at the 3' end of a eukaryotic mRNA primary transcript. |
| GO:0006379 | 1 | mRNA cleavage | Biological Process | Any process in which a pre-mRNA or mRNA molecule is cleaved at specific sites or in a regulated manner. |
| GO:0006384 | 1 | Transcription initiation from RNA polymerase III promoter | Biological Process | Any process involved in the assembly of the RNA polymerase III preinitiation complex (PIC) at an RNA polymerase III promoter region of a DNA template, resulting in the subsequent synthesis of RNA from that promoter. The initiation phase includes PIC assembly and the formation of the first few bonds in the RNA chain, including abortive initiation, which occurs when the first few nucleotides are repeatedly synthesized and then released. Promoter clearance, or release, is the transition between the initiation and elongation phases of transcription. |
| GO:0006388 | 1 | tRNA splicing, via endonucleolytic cleavage and ligation | Biological Process | Splicing of tRNA substrates via recognition of the folded RNA structure that brings the 5' and 3' splice sites into proximity and cleavage of the RNA at both the 3' and 5' splice sites by an endonucleolytic mechanism, followed by ligation of the exons. |
| GO:0006396 | 2 | RNA processing | Biological Process | Any process involved in the conversion of one or more primary RNA transcripts into one or more mature RNA molecules. |
| GO:0006405 | 1 | RNA export from nucleus | Biological Process | The directed movement of RNA from the nucleus to the cytoplasm. |
| GO:0006407 | 1 | rRNA export from nucleus | Biological Process | The directed movement of rRNA from the nucleus to the cytoplasm; the rRNA is usually in the form of ribonucleoproteins. |
| GO:0006412 | 26 | Translation | Biological Process | The cellular metabolic process in which a protein is formed, using the sequence of a mature mRNA or circRNA molecule to specify the sequence of amino acids in a polypeptide chain. Translation is mediated by the ribosome, and begins with the formation of a ternary complex between aminoacylated initiator methionine tRNA, GTP, and initiation factor 2, which subsequently associates with the small subunit of the ribosome and an mRNA or circRNA. Translation ends with the release of a polypeptide chain from the ribosome. |
| GO:0006413 | 2 | Translational initiation | Biological Process | The process preceding formation of the peptide bond between the first two amino acids of a protein. This includes the formation of a complex of the ribosome, mRNA or circRNA, and an initiation complex that contains the first aminoacyl-tRNA. |
| GO:0006414 | 1 | Translational elongation | Biological Process | The successive addition of amino acid residues to a nascent polypeptide chain during protein biosynthesis. |
| GO:0006418 | 1 | tRNA aminoacylation for protein translation | Biological Process | The synthesis of aminoacyl tRNA by the formation of an ester bond between the 3'-hydroxyl group of the most 3' adenosine of the tRNA, to be used in ribosome-mediated polypeptide synthesis. |
| GO:0006457 | 2 | Protein folding | Biological Process | The process of assisting in the covalent and noncovalent assembly of single chain polypeptides or multisubunit complexes into the correct tertiary structure. |
| GO:0006465 | 2 | Signal peptide processing | Biological Process | The proteolytic removal of a signal peptide from a protein during or after transport to a specific location in the cell. |
| GO:0006468 | 40 | Protein phosphorylation | Biological Process | The process of introducing a phosphate group on to a protein. |
| GO:0006470 | 7 | Protein dephosphorylation | Biological Process | The process of removing one or more phosphoric residues from a protein. |
| GO:0006475 | 1 | Internal protein amino acid acetylation | Biological Process | The addition of an acetyl group to a non-terminal amino acid in a protein. |
| GO:0006486 | 2 | Protein glycosylation | Biological Process | A protein modification process that results in the addition of a carbohydrate or carbohydrate derivative unit to a protein amino acid, e.g. the addition of glycan chains to proteins. |
| GO:0006487 | 2 | Protein N-linked glycosylation | Biological Process | A protein glycosylation process in which a carbohydrate or carbohydrate derivative unit is added to a protein via the N4 atom of peptidyl-asparagine, the omega-N of arginine, or the N1' atom peptidyl-tryptophan. |
| GO:0006490 | 2 | Oligosaccharide-lipid intermediate biosynthetic process | Biological Process | The chemical reactions and pathways resulting in the formation of an oligosaccharide-lipid intermediate, such as a molecule of dolichol-P-man or dolicol-P-Glc used in N-linked glycosylation. |
| GO:0006491 | 1 | N-glycan processing | Biological Process | The conversion of N-linked glycan (N = nitrogen) structures from the initially transferred oligosaccharide to a mature form, by the actions of glycosidases and glycosyltransferases. The early processing steps are conserved and play roles in glycoprotein folding and trafficking. |
| GO:0006508 | 76 | Proteolysis | Biological Process | The hydrolysis of proteins into smaller polypeptides and/or amino acids by cleavage of their peptide bonds. |
| GO:0006511 | 6 | Ubiquitin-dependent protein catabolic process | Biological Process | The chemical reactions and pathways resulting in the breakdown of a protein or peptide by hydrolysis of its peptide bonds, initiated by the covalent attachment of a ubiquitin group, or multiple ubiquitin groups, to the protein. |
| GO:0006520 | 1 | Cellular amino acid metabolic process | Biological Process | The chemical reactions and pathways involving amino acids, carboxylic acids containing one or more amino groups, as carried out by individual cells. |
| GO:0006529 | 2 | Asparagine biosynthetic process | Biological Process | The chemical reactions and pathways resulting in the formation of asparagine, 2-amino-3-carbamoylpropanoic acid. |
| GO:0006535 | 3 | Cysteine biosynthetic process from serine | Biological Process | The chemical reactions and pathways resulting in the formation of cysteine from other compounds, including serine. |
| GO:0006537 | 1 | Glutamate biosynthetic process | Biological Process | The chemical reactions and pathways resulting in the formation of glutamate, the anion of 2-aminopentanedioic acid. |
| GO:0006541 | 2 | Glutamine metabolic process | Biological Process | The chemical reactions and pathways involving glutamine, 2-amino-4-carbamoylbutanoic acid. |
| GO:0006544 | 2 | Glycine metabolic process | Biological Process | The chemical reactions and pathways involving glycine, aminoethanoic acid. |
| GO:0006546 | 1 | Glycine catabolic process | Biological Process | The chemical reactions and pathways resulting in the breakdown of glycine, aminoethanoic acid. |
| GO:0006563 | 2 | L-serine metabolic process | Biological Process | The chemical reactions and pathways involving L-serine, the L-enantiomer of serine, i.e. (2S)-2-amino-3-hydroxypropanoic acid. |
| GO:0006571 | 2 | Tyrosine biosynthetic process | Biological Process | The chemical reactions and pathways resulting in the formation of tyrosine, an aromatic amino acid, 2-amino-3-(4-hydroxyphenyl)propanoic acid. |
| GO:0006606 | 1 | Protein import into nucleus | Biological Process | The directed movement of a protein from the cytoplasm to the nucleus. |
| GO:0006614 | 1 | SRP-dependent cotranslational protein targeting to membrane | Biological Process | The targeting of proteins to a membrane that occurs during translation and is dependent upon two key components, the signal-recognition particle (SRP) and the SRP receptor. SRP is a cytosolic particle that transiently binds to the endoplasmic reticulum (ER) signal sequence in a nascent protein, to the large ribosomal unit, and to the SRP receptor in the ER membrane. |
| GO:0006629 | 9 | Lipid metabolic process | Biological Process | The chemical reactions and pathways involving lipids, compounds soluble in an organic solvent but not, or sparingly, in an aqueous solvent. Includes fatty acids; neutral fats, other fatty-acid esters, and soaps; long-chain (fatty) alcohols and waxes; sphingoids and other long-chain bases; glycolipids, phospholipids and sphingolipids; and carotenes, polyprenols, sterols, terpenes and other isoprenoids. |
| GO:0006633 | 7 | Fatty acid biosynthetic process | Biological Process | The chemical reactions and pathways resulting in the formation of a fatty acid, any of the aliphatic monocarboxylic acids that can be liberated by hydrolysis from naturally occurring fats and oils. Fatty acids are predominantly straight-chain acids of 4 to 24 carbon atoms, which may be saturated or unsaturated; branched fatty acids and hydroxy fatty acids also occur, and very long chain acids of over 30 carbons are found in waxes. |
| GO:0006635 | 1 | Fatty acid beta-oxidation | Biological Process | A fatty acid oxidation process that results in the complete oxidation of a long-chain fatty acid. Fatty acid beta-oxidation begins with the addition of coenzyme A to a fatty acid, and occurs by successive cycles of reactions during each of which the fatty acid is shortened by a two-carbon fragment removed as acetyl coenzyme A; the cycle continues until only two or three carbons remain (as acetyl-CoA or propionyl-CoA respectively). |
| GO:0006662 | 1 | Glycerol ether metabolic process | Biological Process | The chemical reactions and pathways involving glycerol ethers, any anhydride formed between two organic hydroxy compounds, one of which is glycerol. |
| GO:0006694 | 1 | Steroid biosynthetic process | Biological Process | The chemical reactions and pathways resulting in the formation of steroids, compounds with a 1,2,cyclopentanoperhydrophenanthrene nucleus; includes de novo formation and steroid interconversion by modification. |
| GO:0006725 | 1 | Cellular aromatic compound metabolic process | Biological Process | The chemical reactions and pathways involving aromatic compounds, any organic compound characterized by one or more planar rings, each of which contains conjugated double bonds and delocalized pi electrons, as carried out by individual cells. |
| GO:0006730 | 1 | One-carbon metabolic process | Biological Process | The chemical reactions and pathways involving the transfer of one-carbon units in various oxidation states. |
| GO:0006749 | 4 | Glutathione metabolic process | Biological Process | The chemical reactions and pathways involving glutathione, the tripeptide glutamylcysteinylglycine, which acts as a coenzyme for some enzymes and as an antioxidant in the protection of sulfhydryl groups in enzymes and other proteins; it has a specific role in the reduction of hydrogen peroxide (H2O2) and oxidized ascorbate, and it participates in the gamma-glutamyl cycle. |
| GO:0006754 | 2 | ATP biosynthetic process | Biological Process | The chemical reactions and pathways resulting in the formation of ATP, adenosine 5'-triphosphate, a universally important coenzyme and enzyme regulator. |
| GO:0006777 | 1 | Mo-molybdopterin cofactor biosynthetic process | Biological Process | The chemical reactions and pathways resulting in the formation of the Mo-molybdopterin cofactor, essential for the catalytic activity of some enzymes. The cofactor consists of a mononuclear molybdenum (Mo) ion coordinated by one or two molybdopterin ligands. |
| GO:0006782 | 2 | Protoporphyrinogen IX biosynthetic process | Biological Process | The chemical reactions and pathways resulting in the formation of protoporphyrinogen IX. |
| GO:0006783 | 2 | Heme biosynthetic process | Biological Process | The chemical reactions and pathways resulting in the formation of heme, any compound of iron complexed in a porphyrin (tetrapyrrole) ring, from less complex precursors. |
| GO:0006796 | 1 | Phosphate-containing compound metabolic process | Biological Process | The chemical reactions and pathways involving the phosphate group, the anion or salt of any phosphoric acid. |
| GO:0006810 | 14 | Transport | Biological Process | The directed movement of substances (such as macromolecules, small molecules, ions) or cellular components (such as complexes and organelles) into, out of or within a cell, or between cells, or within a multicellular organism by means of some agent such as a transporter, pore or motor protein. |
| GO:0006811 | 3 | Ion transport | Biological Process | The directed movement of charged atoms or small charged molecules into, out of or within a cell, or between cells, by means of some agent such as a transporter or pore. |
| GO:0006812 | 1 | Cation transport | Biological Process | The directed movement of cations, atoms or small molecules with a net positive charge, into, out of or within a cell, or between cells, by means of some agent such as a transporter or pore. |
| GO:0006817 | 1 | Phosphate ion transport | Biological Process | The directed movement of phosphate into, out of or within a cell, or between cells, by means of some agent such as a transporter or pore. |
| GO:0006850 | 1 | Mitochondrial pyruvate transport | Biological Process | The directed movement of pyruvate, 2-oxopropanoate, into, out of or within a mitochondrion. |
| GO:0006855 | 5 | Drug transmembrane transport | Biological Process | The process in which a drug is transported from one side of a membrane to the other by means of some agent such as a transporter or pore. |
| GO:0006857 | 1 | Oligopeptide transport | Biological Process | The directed movement of oligopeptides into, out of or within a cell, or between cells, by means of some agent such as a transporter or pore. Oligopeptides are molecules that contain a small number (2 to 20) of amino-acid residues connected by peptide linkages. |
| GO:0006869 | 1 | Lipid transport | Biological Process | The directed movement of lipids into, out of or within a cell, or between cells, by means of some agent such as a transporter or pore. Lipids are compounds soluble in an organic solvent but not, or sparingly, in an aqueous solvent. |
| GO:0006880 | 1 | Intracellular sequestering of iron ion | Biological Process | The process of binding or confining iron ions in an intracellular area such that they are separated from other components of a biological system. |
| GO:0006885 | 2 | Regulation of pH | Biological Process | Any process involved in the maintenance of an internal equilibrium of hydrogen ions, thereby modulating the internal pH, within an organism or cell. |
| GO:0006886 | 6 | Intracellular protein transport | Biological Process | The directed movement of proteins in a cell, including the movement of proteins between specific compartments or structures within a cell, such as organelles of a eukaryotic cell. |
| GO:0006887 | 3 | Exocytosis | Biological Process | A process of secretion by a cell that results in the release of intracellular molecules (e.g. hormones, matrix proteins) contained within a membrane-bounded vesicle. Exocytosis can occur either by full fusion, when the vesicle collapses into the plasma membrane, or by a kiss-and-run mechanism that involves the formation of a transient contact, a pore, between a granule (for exemple of chromaffin cells) and the plasma membrane. The latter process most of the time leads to only partial secretion of the granule content. Exocytosis begins with steps that prepare vesicles for fusion with the membrane (tethering and docking) and ends when molecules are secreted from the cell. |
| GO:0006890 | 1 | Retrograde vesicle-mediated transport, Golgi to ER | Biological Process | The directed movement of substances from the Golgi back to the endoplasmic reticulum, mediated by vesicles bearing specific protein coats such as COPI or COG. |
| GO:0006891 | 1 | Intra-Golgi vesicle-mediated transport | Biological Process | The directed movement of substances within the Golgi, mediated by small transport vesicles. These either fuse with the cis-Golgi or with each other to form the membrane stacks known as the cis-Golgi reticulum (network). |
| GO:0006897 | 1 | Endocytosis | Biological Process | A vesicle-mediated transport process in which cells take up external materials or membrane constituents by the invagination of a small region of the plasma membrane to form a new membrane-bounded vesicle. |
| GO:0006898 | 1 | Receptor-mediated endocytosis | Biological Process | An endocytosis process in which cell surface receptors ensure specificity of transport. A specific receptor on the cell surface binds tightly to the extracellular macromolecule (the ligand) that it recognizes; the plasma-membrane region containing the receptor-ligand complex then undergoes endocytosis, forming a transport vesicle containing the receptor-ligand complex and excluding most other plasma-membrane proteins. Receptor-mediated endocytosis generally occurs via clathrin-coated pits and vesicles. |
| GO:0006906 | 2 | Vesicle fusion | Biological Process | Fusion of the membrane of a transport vesicle with its target membrane. |
| GO:0006950 | 1 | Response to stress | Biological Process | Any process that results in a change in state or activity of a cell or an organism (in terms of movement, secretion, enzyme production, gene expression, etc.) as a result of a disturbance in organismal or cellular homeostasis, usually, but not necessarily, exogenous (e.g. temperature, humidity, ionizing radiation). |
| GO:0006952 | 7 | Defense response | Biological Process | Reactions, triggered in response to the presence of a foreign body or the occurrence of an injury, which result in restriction of damage to the organism attacked or prevention/recovery from the infection caused by the attack. |
| GO:0006970 | 2 | Response to osmotic stress | Biological Process | Any process that results in a change in state or activity of a cell or an organism (in terms of movement, secretion, enzyme production, gene expression, etc.) as a result of a stimulus indicating an increase or decrease in the concentration of solutes outside the organism or cell. |
| GO:0006979 | 6 | Response to oxidative stress | Biological Process | Any process that results in a change in state or activity of a cell or an organism (in terms of movement, secretion, enzyme production, gene expression, etc.) as a result of oxidative stress, a state often resulting from exposure to high levels of reactive oxygen species, e.g. superoxide anions, hydrogen peroxide (H2O2), and hydroxyl radicals. |
| GO:0007018 | 5 | Microtubule-based movement | Biological Process | A microtubule-based process that results in the movement of organelles, other microtubules, or other cellular components. Examples include motor-driven movement along microtubules and movement driven by polymerization or depolymerization of microtubules. |
| GO:0007033 | 2 | Vacuole organization | Biological Process | A process that is carried out at the cellular level which results in the assembly, arrangement of constituent parts, or disassembly of a vacuole. |
| GO:0007051 | 1 | Spindle organization | Biological Process | A process that is carried out at the cellular level which results in the assembly, arrangement of constituent parts, or disassembly of the spindle, the array of microtubules and associated molecules that forms between opposite poles of a eukaryotic cell during DNA segregation and serves to move the duplicated chromosomes apart. |
| GO:0007076 | 2 | Mitotic chromosome condensation | Biological Process | The cell cycle process in which chromatin structure is compacted prior to and during mitosis in eukaryotic cells. |
| GO:0007130 | 1 | Synaptonemal complex assembly | Biological Process | The cell cycle process in which the synaptonemal complex is formed. This is a structure that holds paired chromosomes together during prophase I of meiosis and that promotes genetic recombination. |
| GO:0007165 | 11 | Signal transduction | Biological Process | The cellular process in which a signal is conveyed to trigger a change in the activity or state of a cell. Signal transduction begins with reception of a signal (e.g. a ligand binding to a receptor or receptor activation by a stimulus such as light), or for signal transduction in the absence of ligand, signal-withdrawal or the activity of a constitutively active receptor. Signal transduction ends with regulation of a downstream cellular process, e.g. regulation of transcription or regulation of a metabolic process. Signal transduction covers signaling from receptors located on the surface of the cell and signaling via molecules located within the cell. For signaling between cells, signal transduction is restricted to events at and within the receiving cell. |
| GO:0007186 | 1 | G-protein coupled receptor signaling pathway | Biological Process | A series of molecular signals that proceeds with an activated receptor promoting the exchange of GDP for GTP on the alpha-subunit of an associated heterotrimeric G-protein complex. The GTP-bound activated alpha-G-protein then dissociates from the beta- and gamma-subunits to further transmit the signal within the cell. The pathway begins with receptor-ligand interaction, or for basal GPCR signaling the pathway begins with the receptor activating its G protein in the absence of an agonist, and ends with regulation of a downstream cellular process, e.g. transcription. The pathway can start from the plasma membrane, Golgi or nuclear membrane (PMID:24568158 and PMID:16902576). |
| GO:0007205 | 1 | Protein kinase C-activating G-protein coupled receptor signaling pathway | Biological Process | The series of molecular signals generated as a consequence of a G-protein coupled receptor binding to its physiological ligand, where the pathway proceeds with activation of protein kinase C (PKC). PKC is activated by second messengers including diacylglycerol (DAG). |
| GO:0007264 | 4 | Small GTPase mediated signal transduction | Biological Process | Any series of molecular signals in which a small monomeric GTPase relays one or more of the signals. |
| GO:0007275 | 2 | Multicellular organism development | Biological Process | The biological process whose specific outcome is the progression of a multicellular organism over time from an initial condition (e.g. a zygote or a young adult) to a later condition (e.g. a multicellular animal or an aged adult). |
| GO:0008017 | 7 | Microtubule binding | Molecular Function | Interacting selectively and non-covalently with microtubules, filaments composed of tubulin monomers. |
| GO:0008047 | 1 | Enzyme activator activity | Molecular Function | Binds to and increases the activity of an enzyme. |
| GO:0008081 | 1 | Phosphoric diester hydrolase activity | Molecular Function | Catalysis of the hydrolysis of a phosphodiester to give a phosphomonoester and a free hydroxyl group. |
| GO:0008106 | 1 | Alcohol dehydrogenase (NADP+) activity | Molecular Function | Catalysis of the reaction: an alcohol + NADP+ = an aldehyde + NADPH + H+. |
| GO:0008121 | 1 | Ubiquinol-cytochrome-c reductase activity | Molecular Function | Catalysis of the transfer of a solute or solutes from one side of a membrane to the other according to the reaction: CoQH2 + 2 ferricytochrome c = CoQ + 2 ferrocytochrome c + 2 H+. |
| GO:0008137 | 13 | NADH dehydrogenase (ubiquinone) activity | Molecular Function | Catalysis of the reaction: NADH + H+ + ubiquinone = NAD+ + ubiquinol. |
| GO:0008138 | 2 | Protein tyrosine/serine/threonine phosphatase activity | Molecular Function | Catalysis of the reactions: protein serine + H2O = protein serine + phosphate; protein threonine phosphate + H2O = protein threonine + phosphate; and protein tyrosine phosphate + H2O = protein tyrosine + phosphate. |
| GO:0008152 | 9 | Metabolic process | Biological Process | The chemical reactions and pathways, including anabolism and catabolism, by which living organisms transform chemical substances. Metabolic processes typically transform small molecules, but also include macromolecular processes such as DNA repair and replication, and protein synthesis and degradation. |
| GO:0008168 | 9 | Methyltransferase activity | Molecular Function | Catalysis of the transfer of a methyl group to an acceptor molecule. |
| GO:0008171 | 2 | O-methyltransferase activity | Molecular Function | Catalysis of the transfer of a methyl group to the oxygen atom of an acceptor molecule. |
| GO:0008173 | 1 | RNA methyltransferase activity | Molecular Function | Catalysis of the transfer of a methyl group from a donor to a nucleoside residue in an RNA molecule. |
| GO:0008198 | 2 | Ferrous iron binding | Molecular Function | Interacting selectively and non-covalently with ferrous iron, Fe(II). |
| GO:0008233 | 4 | Peptidase activity | Molecular Function | Catalysis of the hydrolysis of a peptide bond. A peptide bond is a covalent bond formed when the carbon atom from the carboxyl group of one amino acid shares electrons with the nitrogen atom from the amino group of a second amino acid. |
| GO:0008234 | 58 | Cysteine-type peptidase activity | Molecular Function | Catalysis of the hydrolysis of peptide bonds in a polypeptide chain by a mechanism in which the sulfhydryl group of a cysteine residue at the active center acts as a nucleophile. |
| GO:0008236 | 1 | Serine-type peptidase activity | Molecular Function | Catalysis of the hydrolysis of peptide bonds in a polypeptide chain by a catalytic mechanism that involves a catalytic triad consisting of a serine nucleophile that is activated by a proton relay involving an acidic residue (e.g. aspartate or glutamate) and a basic residue (usually histidine). |
| GO:0008237 | 2 | Metallopeptidase activity | Molecular Function | Catalysis of the hydrolysis of peptide bonds by a mechanism in which water acts as a nucleophile, one or two metal ions hold the water molecule in place, and charged amino acid side chains are ligands for the metal ions. |
| GO:0008238 | 1 | Exopeptidase activity | Molecular Function | Catalysis of the hydrolysis of a peptide bond not more than three residues from the N- or C-terminus of a polypeptide chain, in a reaction that requires a free N-terminal amino group, C-terminal carboxyl group or both. |
| GO:0008250 | 1 | Oligosaccharyltransferase complex | Cellular Component | A protein complex that is found in the endoplasmic reticulum membrane of eukaryotes and transfers lipid-linked oligosaccharide precursor to asparagine residues on nascent proteins. In yeast, the complex includes at least nine different subunits, whereas in mammalian cells at least three different forms of the complex have been detected. |
| GO:0008265 | 1 | Mo-molybdopterin cofactor sulfurase activity | Molecular Function | Catalysis of the sulfurylation of the desulfo form of molybdenum cofactor (MoCo), a cofactor required for the activity of some enzymes, such as aldehyde oxidase. |
| GO:0008270 | 315 | Zinc ion binding | Molecular Function | Interacting selectively and non-covalently with zinc (Zn) ions. |
| GO:0008271 | 2 | Secondary active sulfate transmembrane transporter activity | Molecular Function | Catalysis of the secondary active transfer of sulfate from one side of the membrane to the other. Secondary active transport is catalysis of the transfer of a solute from one side of a membrane to the other, up its concentration gradient. The transporter binds the solute and undergoes a series of conformational changes. Transport works equally well in either direction and is driven by a chemiosmotic source of energy. Chemiosmotic sources of energy include uniport, symport or antiport. |
| GO:0008272 | 2 | Sulfate transport | Biological Process | The directed movement of sulfate into, out of or within a cell, or between cells, by means of some agent such as a transporter or pore. |
| GO:0008283 | 1 | Cell proliferation | Biological Process | The multiplication or reproduction of cells, resulting in the expansion of a cell population. |
| GO:0008289 | 1 | Lipid binding | Molecular Function | Interacting selectively and non-covalently with a lipid. |
| GO:0008312 | 1 | 7S RNA binding | Molecular Function | Interacting selectively and non-covalently with 7S RNA, the RNA component of the signal recognition particle (SRP). |
| GO:0008360 | 1 | Regulation of cell shape | Biological Process | Any process that modulates the surface configuration of a cell. |
| GO:0008374 | 2 | O-acyltransferase activity | Molecular Function | Catalysis of the transfer of an acyl group to an oxygen atom on the acceptor molecule. |
| GO:0008375 | 1 | Acetylglucosaminyltransferase activity | Molecular Function | Catalysis of the transfer of an N-acetylglucosaminyl residue from UDP-N-acetyl-glucosamine to a sugar. |
| GO:0008378 | 2 | Galactosyltransferase activity | Molecular Function | Catalysis of the transfer of a galactosyl group to an acceptor molecule, typically another carbohydrate or a lipid. |
| GO:0008408 | 7 | 3'-5' exonuclease activity | Molecular Function | Catalysis of the hydrolysis of ester linkages within nucleic acids by removing nucleotide residues from the 3' end. |
| GO:0008422 | 1 | Beta-glucosidase activity | Molecular Function | Catalysis of the hydrolysis of terminal, non-reducing beta-D-glucose residues with release of beta-D-glucose. |
| GO:0008429 | 1 | Phosphatidylethanolamine binding | Molecular Function | Interacting selectively and non-covalently with phosphatidylethanolamine, any of a class of glycerophospholipids in which a phosphatidyl group is esterified to the hydroxyl group of ethanolamine. |
| GO:0008515 | 1 | Sucrose transmembrane transporter activity | Molecular Function | Enables the transfer of sucrose from one side of the membrane to the other. Sucrose is the disaccharide O-beta-D-fructofuranosyl-(2->1)-alpha-D-glucopyranoside, a sweet-tasting, non-reducing sugar isolated industrially from sugar beet or sugar cane. |
| GO:0008519 | 1 | Ammonium transmembrane transporter activity | Molecular Function | Enables the transfer of ammonium from one side of a membrane to the other. Ammonium is the cation NH4+ which is formed from N2 by root-nodule bacteria in leguminous plants and is an excretory product in ammonotelic animals. |
| GO:0008534 | 1 | Oxidized purine nucleobase lesion DNA N-glycosylase activity | Molecular Function | Catalysis of the removal of oxidized purine bases by cleaving the N-C1' glycosidic bond between the oxidized purine and the deoxyribose sugar. The reaction involves the formation of a covalent enzyme-substrate intermediate. Release of the enzyme and free base by a beta-elimination or a beta, gamma-elimination mechanism results in the cleavage of the DNA backbone 3' of the apurinic (AP) site. |
| GO:0008536 | 1 | Ran GTPase binding | Molecular Function | Interacting selectively and non-covalently with Ran, a conserved Ras-like GTP-binding protein, implicated in nucleocytoplasmic transport, cell cycle progression, spindle assembly, nuclear organization and nuclear envelope (NE) assembly. |
| GO:0008553 | 3 | Hydrogen-exporting ATPase activity, phosphorylative mechanism | Molecular Function | Catalysis of the transfer of protons from one side of a membrane to the other according to the reaction: ATP + H2O + H+(in) -> ADP + phosphate + H+(out). These transporters use a phosphorylative mechanism, which have a phosphorylated intermediate state during the ion transport cycle. |
| GO:0008565 | 2 | Protein transporter activity | Molecular Function | Enables the directed movement of proteins into, out of or within a cell, or between cells. |
| GO:0008568 | 11 | Microtubule-severing ATPase activity | Molecular Function | Catalysis of the reaction: ATP + H2O = ADP + phosphate. Catalysis of the severing of a microtubule at a specific spot along its length, coupled to the hydrolysis of ATP. |
| GO:0008574 | 1 | ATP-dependent microtubule motor activity, plus-end-directed | Molecular Function | Catalysis of movement along a microtubule toward the plus end, coupled to the hydrolysis of ATP. |
| GO:0008643 | 1 | Carbohydrate transport | Biological Process | The directed movement of carbohydrate into, out of or within a cell, or between cells, by means of some agent such as a transporter or pore. Carbohydrates are any of a group of organic compounds based of the general formula Cx(H2O)y. |
| GO:0008649 | 2 | rRNA methyltransferase activity | Molecular Function | Catalysis of the transfer of a methyl group from S-adenosyl-L-methionine to a nucleoside residue in an rRNA molecule. |
| GO:0008661 | 2 | 1-deoxy-D-xylulose-5-phosphate synthase activity | Molecular Function | Catalysis of the reaction: D-glyceraldehyde 3-phosphate + H(+) + pyruvate = 1-deoxy-D-xylulose 5-phosphate + CO(2). |
| GO:0008676 | 1 | 3-deoxy-8-phosphooctulonate synthase activity | Molecular Function | Catalysis of the reaction: D-arabinose 5-phosphate + H(2)O + phosphoenolpyruvate = 8-phospho-3-deoxy-D-manno-oct-2-ulosonate + 2 H(+) + phosphate. |
| GO:0008757 | 2 | S-adenosylmethionine-dependent methyltransferase activity | Molecular Function | Catalysis of the transfer of a methyl group from S-adenosyl-L-methionine to a substrate. |
| GO:0008832 | 1 | dGTPase activity | Molecular Function | Catalysis of the reaction: dGTP + H(2)O = 2'-deoxyguanosine + 2 H(+) + triphosphate. |
| GO:0008836 | 1 | Diaminopimelate decarboxylase activity | Molecular Function | Catalysis of the reaction: meso-2,6-diaminopimelate + H(+) = L-lysine + CO(2). |
| GO:0008964 | 4 | Phosphoenolpyruvate carboxylase activity | Molecular Function | Catalysis of the reaction: phosphate + oxaloacetate = phosphoenolpyruvate + HCO3-. |
| GO:0008977 | 2 | Prephenate dehydrogenase (NAD+) activity | Molecular Function | Catalysis of the reaction: NAD(+) + prephenate = (4-hydroxyphenyl)pyruvate + CO(2) + NADH. |
| GO:0009001 | 2 | Serine O-acetyltransferase activity | Molecular Function | Catalysis of the reaction: L-serine + acetyl-CoA = O-acetyl-L-serine + CoA. |
| GO:0009045 | 1 | Xylose isomerase activity | Molecular Function | Catalysis of the reaction: D-xylose = D-xylulose. |
| GO:0009055 | 11 | Electron carrier activity | Molecular Function | Any molecular entity that serves as an electron acceptor and electron donor in an electron transport chain. An electron transport chain is a process in which a series of electron carriers operate together to transfer electrons from donors to any of several different terminal electron acceptors to generate a transmembrane electrochemical gradient. |
| GO:0009058 | 2 | Biosynthetic process | Biological Process | The chemical reactions and pathways resulting in the formation of substances; typically the energy-requiring part of metabolism in which simpler substances are transformed into more complex ones. |
| GO:0009061 | 2 | Anaerobic respiration | Biological Process | The enzymatic release of energy from inorganic and organic compounds (especially carbohydrates and fats) which uses compounds other than oxygen (e.g. nitrate, sulfate) as the terminal electron acceptor. |
| GO:0009072 | 1 | Aromatic amino acid family metabolic process | Biological Process | The chemical reactions and pathways involving aromatic amino acid family, amino acids with aromatic ring (phenylalanine, tyrosine, tryptophan). |
| GO:0009089 | 1 | Lysine biosynthetic process via diaminopimelate | Biological Process | The chemical reactions and pathways resulting in the formation of lysine, via the intermediate diaminopimelate. |
| GO:0009116 | 1 | Nucleoside metabolic process | Biological Process | The chemical reactions and pathways involving a nucleoside, a nucleobase linked to either beta-D-ribofuranose (a ribonucleoside) or 2-deoxy-beta-D-ribofuranose, (a deoxyribonucleoside), e.g. adenosine, guanosine, inosine, cytidine, uridine and deoxyadenosine, deoxyguanosine, deoxycytidine and thymidine (= deoxythymidine). |
| GO:0009117 | 1 | Nucleotide metabolic process | Biological Process | The chemical reactions and pathways involving a nucleotide, a nucleoside that is esterified with (ortho)phosphate or an oligophosphate at any hydroxyl group on the glycose moiety; may be mono-, di- or triphosphate; this definition includes cyclic nucleotides (nucleoside cyclic phosphates). |
| GO:0009142 | 1 | Nucleoside triphosphate biosynthetic process | Biological Process | The chemical reactions and pathways resulting in the formation of a nucleoside triphosphate, a compound consisting of a nucleobase linked to a deoxyribose or ribose sugar esterified with triphosphate on the sugar. |
| GO:0009263 | 1 | Deoxyribonucleotide biosynthetic process | Biological Process | The chemical reactions and pathways resulting in the formation of a deoxyribonucleotide, a compound consisting of deoxyribonucleoside (a base linked to a deoxyribose sugar) esterified with a phosphate group at either the 3' or 5'-hydroxyl group of the sugar. |
| GO:0009317 | 2 | Acetyl-CoA carboxylase complex | Cellular Component | A protein complex that catalyzes the first step in long-chain fatty acid biosynthesis. For example, in E. coli the complex is heterohexameric and composed of biotin carbonyl carrier protein, biotin carboxylase and the acetate CoA-transferase complex. |
| GO:0009396 | 2 | Folic acid-containing compound biosynthetic process | Biological Process | The chemical reactions and pathways resulting in the formation of folic acid and its derivatives. |
| GO:0009407 | 2 | Toxin catabolic process | Biological Process | The chemical reactions and pathways resulting in the breakdown of toxin, a poisonous compound (typically a protein) that is produced by cells or organisms and that can cause disease when introduced into the body or tissues of an organism. |
| GO:0009411 | 1 | Response to UV | Biological Process | Any process that results in a change in state or activity of a cell or an organism (in terms of movement, secretion, enzyme production, gene expression, etc.) as a result of an ultraviolet radiation (UV light) stimulus. Ultraviolet radiation is electromagnetic radiation with a wavelength in the range of 10 to 380 nanometers. |
| GO:0009414 | 2 | Response to water deprivation | Biological Process | Any process that results in a change in state or activity of a cell or an organism (in terms of movement, secretion, enzyme production, gene expression, etc.) as a result of a water deprivation stimulus, prolonged deprivation of water. |
| GO:0009501 | 1 | Amyloplast | Cellular Component | A plastid whose main function is to synthesize and store starch. |
| GO:0009505 | 2 | Plant-type cell wall | Cellular Component | A more or less rigid stucture lying outside the cell membrane of a cell and composed of cellulose and pectin and other organic and inorganic substances. |
| GO:0009506 | 10 | Plasmodesma | Cellular Component | A fine cytoplasmic channel, found in all higher plants, that connects the cytoplasm of one cell to that of an adjacent cell. |
| GO:0009507 | 42 | Chloroplast | Cellular Component | A chlorophyll-containing plastid with thylakoids organized into grana and frets, or stroma thylakoids, and embedded in a stroma. |
| GO:0009521 | 1 | Photosystem | Cellular Component | A complex located in a photosynthetic membrane that consists of a photoreaction center associated with accessory pigments and electron carriers. Examples of this component are found in Arabidopsis thaliana and in photosynthetic bacterial and archaeal species. |
| GO:0009522 | 3 | Photosystem I | Cellular Component | A photosystem that contains an iron-sulfur reaction center associated with accessory pigments and electron carriers. In cyanobacteria and chloroplasts, photosystem I functions as a light-dependent plastocyanin-ferredoxin oxidoreductase, transferring electrons from plastocyanin to ferredoxin; in photosynthetic bacteria that have only a single type I photosystem, such as the green sulfur bacteria, electrons can go either to ferredoxin (Fd) -> NAD+ or to menaquinone (MK) -> Cytb/FeS -> Cytc555 -> photosystem I (cyclic photophosphorylation). |
| GO:0009523 | 4 | Photosystem II | Cellular Component | A photosystem that contains a pheophytin-quinone reaction center with associated accessory pigments and electron carriers. In cyanobacteria and chloroplasts, in the presence of light, PSII functions as a water-plastoquinone oxidoreductase, transferring electrons from water to plastoquinone, whereas other photosynthetic bacteria carry out anoxygenic photosynthesis and oxidize other compounds to re-reduce the photoreaction center. |
| GO:0009526 | 1 | Plastid envelope | Cellular Component | The double lipid bilayer enclosing a plastid and separating its contents from the rest of the cytoplasm; includes the intermembrane space. |
| GO:0009533 | 1 | Chloroplast stromal thylakoid | Cellular Component | Unstacked thylakoids that connect the grana stacks through the stroma. |
| GO:0009535 | 12 | Chloroplast thylakoid membrane | Cellular Component | The pigmented membrane of a chloroplast thylakoid. An example of this component is found in Arabidopsis thaliana. |
| GO:0009536 | 9 | Plastid | Cellular Component | Any member of a family of organelles found in the cytoplasm of plants and some protists, which are membrane-bounded and contain DNA. Plant plastids develop from a common type, the proplastid. |
| GO:0009543 | 1 | Chloroplast thylakoid lumen | Cellular Component | The cavity enclosed within the chloroplast thylakoid membrane. An example of this component is found in Arabidopsis thaliana. |
| GO:0009555 | 1 | Pollen development | Biological Process | The process whose specific outcome is the progression of the pollen grain over time, from its formation to the mature structure. The process begins with the meiosis of the microsporocyte to form four haploid microspores. The nucleus of each microspore then divides by mitosis to form a two-celled organism, the pollen grain, that contains a tube cell as well as a smaller generative cell. The pollen grain is surrounded by an elaborate cell wall. In some species, the generative cell immediately divides again to give a pair of sperm cells. In most flowering plants, however this division takes place later, in the tube that develops when a pollen grain germinates. |
| GO:0009570 | 19 | Chloroplast stroma | Cellular Component | The space enclosed by the double membrane of a chloroplast but excluding the thylakoid space. It contains DNA, ribosomes and some temporary products of photosynthesis. |
| GO:0009585 | 1 | Red, far-red light phototransduction | Biological Process | The sequence of reactions within a cell required to convert absorbed photons from red or far-red light into a molecular signal; the red, far-red light range is defined as having a wavelength within the range 660-730 nm. |
| GO:0009590 | 1 | Detection of gravity | Biological Process | The series of events in which a gravitational stimulus is received and converted into a molecular signal. |
| GO:0009611 | 1 | Response to wounding | Biological Process | Any process that results in a change in state or activity of a cell or an organism (in terms of movement, secretion, enzyme production, gene expression, etc.) as a result of a stimulus indicating damage to the organism. |
| GO:0009651 | 3 | Response to salt stress | Biological Process | Any process that results in a change in state or activity of a cell or an organism (in terms of movement, secretion, enzyme production, gene expression, etc.) as a result of a stimulus indicating an increase or decrease in the concentration of salt (particularly but not exclusively sodium and chloride ions) in the environment. |
| GO:0009654 | 1 | Photosystem II oxygen evolving complex | Cellular Component | A complex, composed of a cluster of manganese, calcium and chloride ions bound to extrinsic proteins, that catalyzes the splitting of water to O2 and 4 H+. In cyanobacteria there are five extrinsic proteins in OEC (PsbO, PsbP-like, PsbQ-like, PsbU and PsbV), while in plants there are only three (PsbO, PsbP and PsbQ). |
| GO:0009664 | 3 | Plant-type cell wall organization | Biological Process | A process that results in the assembly and arrangement of constituent parts of the cellulose and pectin-containing cell wall, or in the disassembly of the cellulose and pectin-containing cell wall. This process is carried out at the cellular level. An example of this process is found in Arabidopsis thaliana. |
| GO:0009702 | 2 | L-arabinokinase activity | Molecular Function | Catalysis of the reaction: L-arabinose + ATP = beta-L-arabinose 1-phosphate + ADP + 2 H(+). |
| GO:0009705 | 13 | Plant-type vacuole membrane | Cellular Component | The lipid bilayer surrounding a vacuole that retains the same shape regardless of cell cycle phase. The membrane separates its contents from the cytoplasm of the cell. An example of this component is found in Arabidopsis thaliana. |
| GO:0009706 | 1 | Chloroplast inner membrane | Cellular Component | The inner, i.e. lumen-facing, lipid bilayer of the chloroplast envelope; also faces the chloroplast stroma. |
| GO:0009725 | 1 | Response to hormone | Biological Process | Any process that results in a change in state or activity of a cell or an organism (in terms of movement, secretion, enzyme production, gene expression, etc.) as a result of a hormone stimulus. |
| GO:0009733 | 1 | Response to auxin | Biological Process | Any process that results in a change in state or activity of a cell or an organism (in terms of movement, secretion, enzyme production, gene expression, etc.) as a result of an auxin stimulus. |
| GO:0009734 | 2 | Auxin-activated signaling pathway | Biological Process | A series of molecular signals generated by the binding of the plant hormone auxin to a receptor, and ending with modulation of a downstream cellular process, e.g. transcription. |
| GO:0009735 | 1 | Response to cytokinin | Biological Process | Any process that results in a change in state or activity of a cell or an organism (in terms of movement, secretion, enzyme production, gene expression, etc.) as a result of a cytokinin stimulus. |
| GO:0009736 | 1 | Cytokinin-activated signaling pathway | Biological Process | A series of molecular signals generated by the binding of a cytokinin to a receptor, and ending with regulation of a downstream cellular process, e.g. transcription. |
| GO:0009737 | 3 | Response to abscisic acid | Biological Process | Any process that results in a change in state or activity of a cell or an organism (in terms of movement, secretion, enzyme production, gene expression, etc.) as a result of an abscisic acid stimulus. |
| GO:0009739 | 1 | Response to gibberellin | Biological Process | Any process that results in a change in state or activity of a cell or an organism (in terms of movement, secretion, enzyme production, gene expression, etc.) as a result of a gibberellin stimulus. |
| GO:0009741 | 1 | Response to brassinosteroid | Biological Process | Any process that results in a change in state or activity of a cell or an organism (in terms of movement, secretion, enzyme production, gene expression, etc.) as a result of a brassinosteroid stimulus. |
| GO:0009744 | 1 | Response to sucrose | Biological Process | Any process that results in a change in state or activity of a cell or an organism (in terms of movement, secretion, enzyme production, gene expression, etc.) as a result of a sucrose stimulus. |
| GO:0009767 | 1 | Photosynthetic electron transport chain | Biological Process | A process, occurring as part of photosynthesis, in which light provides the energy for a series of electron carriers to operate together to transfer electrons and generate a transmembrane electrochemical gradient. |
| GO:0009772 | 4 | Photosynthetic electron transport in photosystem II | Biological Process | A photosynthetic electron transport chain in which electrons move from the primary electron acceptor (Quinone, Q) through a chain of electron transport molecules in the thylakoid membrane until they reach the ultimate electron acceptor of Photosystem II, which is plastocyanin (PC). The electron is then passed to the P700 chlorophyll a molecules of the reaction centre of photosystem I. |
| GO:0009773 | 1 | Photosynthetic electron transport in photosystem I | Biological Process | A photosynthetic electron transport chain in which electrons move from the primary electron acceptor (Quinone, X) through a chain of electron transport molecules in the thylakoid membrane until they reach ferredoxin which passes the electron to the ultimate electron acceptor; NADP. |
| GO:0009788 | 1 | Negative regulation of abscisic acid-activated signaling pathway | Biological Process | Any process that stops, prevents, or reduces the frequency, rate or extent of abscisic acid (ABA) signaling. |
| GO:0009790 | 1 | Embryo development | Biological Process | The process whose specific outcome is the progression of an embryo from its formation until the end of its embryonic life stage. The end of the embryonic stage is organism-specific. For example, for mammals, the process would begin with zygote formation and end with birth. For insects, the process would begin at zygote formation and end with larval hatching. For plant zygotic embryos, this would be from zygote formation to the end of seed dormancy. For plant vegetative embryos, this would be from the initial determination of the cell or group of cells to form an embryo until the point when the embryo becomes independent of the parent plant. |
| GO:0009791 | 1 | Post-embryonic development | Biological Process | The process whose specific outcome is the progression of the organism over time, from the completion of embryonic development to the mature structure. See embryonic development. |
| GO:0009793 | 3 | Embryo development ending in seed dormancy | Biological Process | The process whose specific outcome is the progression of the embryo over time, from zygote formation to the end of seed dormancy. An example of this process is found in Arabidopsis thaliana. |
| GO:0009812 | 1 | Flavonoid metabolic process | Biological Process | The chemical reactions and pathways involving flavonoids, a group of water-soluble phenolic derivatives containing a flavan skeleton including flavones, flavonols and flavanoids, and anthocyanins. |
| GO:0009813 | 2 | Flavonoid biosynthetic process | Biological Process | The chemical reactions and pathways resulting in the formation of flavonoids, a group of phenolic derivatives containing a flavan skeleton. |
| GO:0009825 | 2 | Multidimensional cell growth | Biological Process | The process in which a cell irreversibly increases in size in two or three [spatial] dimensions or along two or three axes. |
| GO:0009832 | 2 | Plant-type cell wall biogenesis | Biological Process | A cellular process that results in the biosynthesis of constituent macromolecules, assembly, and arrangement of constituent parts of a cellulose and pectin-containing cell wall. An example of this is found in Arabidopsis thaliana. |
| GO:0009834 | 1 | Plant-type secondary cell wall biogenesis | Biological Process | A cellular process that results in the biosynthesis of constituent macromolecules, assembly, and arrangement of constituent parts of inextensible cellulose- and pectin-containing cell walls that are formed between the plasma membrane and primary cell wall after cell expansion is complete. An example of this is found in Arabidopsis thaliana. |
| GO:0009835 | 1 | Fruit ripening | Biological Process | An aging process that has as participant a fruit. Ripening causes changes in one or more characteristics of a fruit (color, aroma, flavor, texture, hardness, cell wall structure) and may make it more attractive to animals and aid in seed dispersal. |
| GO:0009838 | 1 | Abscission | Biological Process | The controlled shedding of a body part. |
| GO:0009856 | 1 | Pollination | Biological Process | The cascade of biological processes occurring in plants beginning when the pollen lands on the female reproductive organs of a plant and continuing up to, but not including, fertilization, as defined by sperm-egg cell fusion. |
| GO:0009860 | 1 | Pollen tube growth | Biological Process | Growth of pollen via tip extension of the intine wall. |
| GO:0009867 | 1 | Jasmonic acid mediated signaling pathway | Biological Process | A series of molecular signals mediated by jasmonic acid. |
| GO:0009873 | 1 | Ethylene-activated signaling pathway Biological Process | | A series of molecular signals generated by the reception of ethylene (ethene, C2H4) by a receptor and ending with modulation of a cellular process, e.g. transcription. |
| GO:0009897 | 1 | External side of plasma membrane | Cellular Component | The leaflet of the plasma membrane that faces away from the cytoplasm and any proteins embedded or anchored in it or attached to its surface. |
| GO:0009909 | 1 | Regulation of flower development | Biological Process | Any process that modulates the frequency, rate or extent of flower development. |
| GO:0009910 | 1 | Negative regulation of flower development | Biological Process | Any process that stops, prevents, or reduces the frequency, rate or extent of flower development. |
| GO:0009911 | 1 | Positive regulation of flower development | Biological Process | Any process that activates or increases the frequency, rate or extent of flower development. |
| GO:0009930 | 1 | Longitudinal side of cell surface | Cellular Component | The side of the cell parallel to the zygotic axis. |
| GO:0009933 | 1 | Meristem structural organization | Biological Process | Organization of a region of tissue in a plant that is composed of one or more undifferentiated cells capable of undergoing mitosis and differentiation, thereby effecting growth and development of a plant by giving rise to more meristem or specialized tissue. |
| GO:0009941 | 5 | Chloroplast envelope | Cellular Component | The double lipid bilayer enclosing the chloroplast and separating its contents from the rest of the cytoplasm; includes the intermembrane space. |
| GO:0009960 | 1 | Endosperm development | Biological Process | The process whose specific outcome is the progression of the endosperm over time, from its formation to the mature structure. The endosperm is formed during fertilization and provides nutrients to the developing embryo. |
| GO:0009987 | 5 | Cellular process | Biological Process | Any process that is carried out at the cellular level, but not necessarily restricted to a single cell. For example, cell communication occurs among more than one cell, but occurs at the cellular level. |
| GO:0010020 | 1 | Chloroplast fission | Biological Process | The division of a chloroplast within a cell to form two or more separate chloroplast compartments. This division occurs independently of mitosis. |
| GO:0010029 | 1 | Regulation of seed germination | Biological Process | Any process that modulates the frequency, rate or extent of seed germination. |
| GO:0010091 | 1 | Trichome branching | Biological Process | Any process involved in the formation of branches in plant hair cells. An example of this process is found in Arabidopsis thaliana. |
| GO:0010114 | 2 | Response to red light | Biological Process | Any process that results in a change in state or activity of a cell or an organism (in terms of movement, secretion, enzyme production, gene expression, etc.) as a result of a red light stimulus. Red light is electromagnetic radiation of wavelength of 580-700nm. An example of this response is seen at the beginning of many plant species developmental stages. These include germination, and the point when cotyledon expansion is triggered. In certain species these processes take place in response to absorption of red light by the pigment molecule phytochrome, but the signal can be reversed by exposure to far red light. During the initial phase the phytochrome molecule is only present in the red light absorbing form, but on absorption of red light it changes to a far red light absorbing form, triggering progress through development. An immediate short period of exposure to far red light entirely returns the pigment to its initial state and prevents triggering of the developmental process. A thirty minute break between red and subsequent far red light exposure renders the red light effect irreversible, and development then occurs regardless of whether far red light exposure subsequently occurs. |
| GO:0010165 | 2 | Response to X-ray | Biological Process | Any process that results in a change in state or activity of a cell or an organism (in terms of movement, secretion, enzyme production, gene expression, etc.) as a result of X-ray radiation. An X-ray is a form of electromagnetic radiation with a wavelength in the range of 10 nanometers to 100 picometers (corresponding to frequencies in the range 30 PHz to 3 EHz). |
| GO:0010181 | 3 | FMN binding | Molecular Function | Interacting selectively and non-covalently with flavin mono nucleotide. Flavin mono nucleotide (FMN) is the coenzyme or the prosthetic group of various flavoprotein oxidoreductase enzymes. |
| GO:0010200 | 1 | Response to chitin | Biological Process | A process that results in a change in state or activity of a cell or an organism (in terms of movement, secretion, enzyme production, gene expression, etc.) as a result of a chitin stimulus. |
| GO:0010215 | 1 | Cellulose microfibril organization | Biological Process | A process that is carried out at the cellular level which results in the assembly, arrangement of constituent parts, or disassembly of a cellulose microfibril, any of the cellulose structures laid down in orthogonal layers in a plant cell wall. |
| GO:0010218 | 1 | Response to far red light | Biological Process | Any process that results in a change in state or activity of a cell or an organism (in terms of movement, secretion, enzyme production, gene expression, etc.) as a result of far red light stimulus. Far red light is electromagnetic radiation of wavelength 700-800nm. An example of this response is seen at the beginning of many plant species developmental stages. These include germination, and the point when cotyledon expansion is triggered. In certain species these processes take place in response to absorption of red light by the pigment molecule phytochrome, but the signal can be reversed by exposure to far red light. During the initial phase the phytochrome molecule is only present in the red light absorbing form, but on absorption of red light it changes to a far red light absorbing form, triggering progress through development. An immediate short period of exposure to far red light entirely returns the pigment to its initial state and prevents triggering of the developmental process. A thirty minute break between red and subsequent far red light exposure renders the red light effect irreversible, and development then occurs regardless of whether far red light exposure subsequently occurs. |
| GO:0010222 | 1 | Stem vascular tissue pattern formation | Biological Process | Vascular tissue pattern formation as it occurs in the stem of vascular plants. |
| GO:0010229 | 1 | Inflorescence development | Biological Process | The process whose specific outcome is the progression of an inflorescence over time, from its formation to the mature structure. |
| GO:0010306 | 1 | Rhamnogalacturonan II biosynthetic process | Biological Process | The chemical reactions and pathways resulting in the formation of rhamnogalacturonan II, a low molecular mass (5 - 10KDa) pectic polysaccharide, conserved in the primary walls of dicotyledenous and monocotyledenous plants and gymnosperms. |
| GO:0010319 | 1 | Stromule | Cellular Component | Thin filamentous structure extending from the surface of all plastid types examined so far, including chloroplast, proplastid, etioplast, leucoplast, amyloplast, and chromoplast. In general, stromules are more abundant in tissues containing non-green plastids, and in cells containing smaller plastids. The primary function of stromules is still unresolved, although the presence of stromules markedly increases the plastid surface area, potentially increasing transport to and from the cytosol. Other functions of stromules, such as transfer of macromolecules between plastids and starch granule formation in cereal endosperm, may be restricted to particular tissues and cell types. |
| GO:0010342 | 1 | Endosperm cellularization | Biological Process | The separation of the multi-nucleate endosperm into individual cells. In many plant species, the endosperm that nurtures the embryo in the seed initially develops as a syncytium. This syncytial phase ends with simultaneous partitioning of the multi-nucleate cytoplasm into individual cells, a process referred to as cellularization. |
| GO:0010345 | 2 | Suberin biosynthetic process | Biological Process | The chemical reactions and pathways resulting in the formation of suberin monomers and suberin polyesters. Suberin monomers are derived from fatty acids and trans-cinnamic acids. The monomers are then cross-linked with glycerols. |
| GO:0010359 | 1 | Regulation of anion channel activity | Biological Process | Any process that modulates the frequency, rate or extent of anion channel activity. |
| GO:0010588 | 1 | Cotyledon vascular tissue pattern formation | Biological Process | Vascular tissue pattern formation as it occurs in the cotyledon of vascular plants. |
| GO:0010686 | 1 | Tetracyclic triterpenoid biosynthetic process | Biological Process | The chemical reactions and pathways resulting in the formation of tetracyclic triterpenoid compounds, terpenoids with six isoprene units and 4 carbon rings. |
| GO:0010731 | 1 | Protein glutathionylation | Biological Process | The protein modification process in which a glutathione molecule is added to a protein amino acid through a disulfide linkage. |
| GO:0012505 | 5 | Endomembrane system | Cellular Component | A collection of membranous structures involved in transport within the cell. The main components of the endomembrane system are endoplasmic reticulum, Golgi bodies, vesicles, cell membrane and nuclear envelope. Members of the endomembrane system pass materials through each other or though the use of vesicles. |
| GO:0015031 | 2 | Protein transport | Biological Process | The directed movement of proteins into, out of or within a cell, or between cells, by means of some agent such as a transporter or pore. |
| GO:0015035 | 1 | Protein disulfide oxidoreductase activity | Molecular Function | Catalysis of the reaction: a protein with reduced sulfide groups = a protein with oxidized disulfide bonds. |
| GO:0015074 | 4 | DNA integration | Biological Process | The process in which a segment of DNA is incorporated into another, usually larger, DNA molecule such as a chromosome. |
| GO:0015078 | 2 | Hydrogen ion transmembrane transporter activity | Molecular Function | Enables the transfer of hydrogen ions from one side of a membrane to the other. |
| GO:0015079 | 3 | Potassium ion transmembrane transporter activity | Molecular Function | Enables the transfer of potassium ions (K+) from one side of a membrane to the other. |
| GO:0015095 | 1 | Magnesium ion transmembrane transporter activity | Molecular Function | Enables the transfer of magnesium (Mg) ions from one side of a membrane to the other. |
| GO:0015140 | 3 | Malate transmembrane transporter activity | Molecular Function | Enables the transfer of malate from one side of the membrane to the other. Malate is a chiral hydroxydicarboxylic acid, hydroxybutanedioic acid. The (+) enantiomer is an important intermediate in metabolism as a component of both the TCA cycle and the glyoxylate cycle. |
| GO:0015171 | 1 | Amino acid transmembrane transporter activity | Molecular Function | Enables the transfer of amino acids from one side of a membrane to the other. Amino acids are organic molecules that contain an amino group and a carboxyl group. |
| GO:0015232 | 3 | Heme transporter activity | Molecular Function | Enables the directed movement of heme, any compound of iron complexed in a porphyrin (tetrapyrrole) ring, into, out of or within a cell, or between cells. |
| GO:0015238 | 5 | Drug transmembrane transporter activity | Molecular Function | Enables the directed movement of a drug from one side of a membrane to the other. A drug is any naturally occurring or synthetic substance, other than a nutrient, that, when administered or applied to an organism, affects the structure or functioning of the organism; in particular, any such substance used in the diagnosis, prevention, or treatment of disease. |
| GO:0015245 | 1 | Fatty acid transporter activity | Molecular Function | Enables the directed movement of fatty acids into, out of or within a cell, or between cells. Fatty acids are aliphatic monocarboxylic acids liberated from naturally occurring fats and oils by hydrolysis. |
| GO:0015267 | 1 | Channel activity | Molecular Function | Enables the energy-independent facilitated diffusion, mediated by passage of a solute through a transmembrane aqueous pore or channel. Stereospecificity is not exhibited but this transport may be specific for a particular molecular species or class of molecules. |
| GO:0015297 | 5 | Antiporter activity | Molecular Function | Enables the active transport of a solute across a membrane by a mechanism whereby two or more species are transported in opposite directions in a tightly coupled process not directly linked to a form of energy other than chemiosmotic energy. The reaction is: solute A(out) + solute B(in) = solute A(in) + solute B(out). |
| GO:0015385 | 1 | Sodium:proton antiporter activity | Molecular Function | Catalysis of the transfer of a solute or solutes from one side of a membrane to the other according to the reaction: Na+(out) + H+(in) = Na+(in) + H+(out). |
| GO:0015693 | 1 | Magnesium ion transport | Biological Process | The directed movement of magnesium (Mg) ions into, out of or within a cell, or between cells, by means of some agent such as a transporter or pore. |
| GO:0015696 | 1 | Ammonium transport | Biological Process | The directed movement of ammonium into, out of or within a cell, or between cells, by means of some agent such as a transporter or pore. Ammonium is the cation NH4+ which is formed from N2 by root-nodule bacteria in leguminous plants and is an excretory product in ammonotelic animals. |
| GO:0015698 | 1 | Inorganic anion transport | Biological Process | The directed movement of inorganic anions into, out of or within a cell, or between cells, by means of some agent such as a transporter or pore. Inorganic anions are atoms or small molecules with a negative charge which do not contain carbon in covalent linkage. |
| GO:0015743 | 3 | Malate transport | Biological Process | The directed movement of malate into, out of or within a cell, or between cells, by means of some agent such as a transporter or pore. |
| GO:0015758 | 1 | Glucose transport | Biological Process | The directed movement of the hexose monosaccharide glucose into, out of or within a cell, or between cells, by means of some agent such as a transporter or pore. |
| GO:0015770 | 1 | Sucrose transport | Biological Process | The directed movement of sucrose into, out of or within a cell, or between cells by means of some agent such as a transporter or pore. Sucrose is the disaccharide fructofuranosyl-glucopyranoside. |
| GO:0015780 | 2 | Nucleotide-sugar transport | Biological Process | The directed movement of nucleotide-sugars into, out of or within a cell, or between cells, by means of some agent such as a transporter or pore. Nucleotide-sugars are any nucleotide in which the distal phosphoric residue of a nucleoside 5'-diphosphate is in glycosidic linkage with a monosaccharide or monosaccharide derivative. |
| GO:0015886 | 3 | Heme transport | Biological Process | The directed movement of heme, any compound of iron complexed in a porphyrin (tetrapyrrole) ring, into, out of or within a cell, or between cells, by means of some agent such as a transporter or pore. |
| GO:0015908 | 1 | Fatty acid transport | Biological Process | The directed movement of fatty acids into, out of or within a cell, or between cells, by means of some agent such as a transporter or pore. Fatty acids are aliphatic monocarboxylic acids liberated from naturally occurring fats and oils by hydrolysis. |
| GO:0015930 | 1 | Glutamate synthase activity | Molecular Function | Catalysis of the formation of L-glutamine and 2-oxoglutarate from L-glutamate, using NADH, NADPH or ferredoxin as hydrogen acceptors. |
| GO:0015934 | 2 | Large ribosomal subunit | Cellular Component | The larger of the two subunits of a ribosome. Two sites on the ribosomal large subunit are involved in translation, namely the aminoacyl site (A site) and peptidyl site (P site). |
| GO:0015935 | 11 | Small ribosomal subunit | Cellular Component | The smaller of the two subunits of a ribosome. |
| GO:0015977 | 3 | Carbon fixation | Biological Process | A metabolic process in which carbon (usually derived from carbon dioxide) is incorporated into organic compounds (usually carbohydrates). |
| GO:0015979 | 6 | Photosynthesis | Biological Process | The synthesis by organisms of organic chemical compounds, especially carbohydrates, from carbon dioxide (CO2) using energy obtained from light rather than from the oxidation of chemical compounds. |
| GO:0015986 | 7 | ATP synthesis coupled proton transport | Biological Process | The transport of protons across a membrane to generate an electrochemical gradient (proton-motive force) that powers ATP synthesis. |
| GO:0015991 | 7 | ATP hydrolysis coupled proton transport | Biological Process | The transport of protons against an electrochemical gradient, using energy from ATP hydrolysis. |
| GO:0015992 | 1 | Proton transport | Biological Process | The directed movement of protons (hydrogen ions) into, out of or within a cell, or between cells, by means of some agent such as a transporter or pore. |
| GO:0016020 | 67 | Membrane | Cellular Component | A lipid bilayer along with all the proteins and protein complexes embedded in it an attached to it. |
| GO:0016021 | 215 | Integral component of membrane | Cellular Component | The component of a membrane consisting of the gene products and protein complexes having at least some part of their peptide sequence embedded in the hydrophobic region of the membrane. |
| GO:0016043 | 1 | Cellular component organization | Biological Process | A process that results in the assembly, arrangement of constituent parts, or disassembly of a cellular component. |
| GO:0016045 | 1 | Detection of bacterium | Biological Process | The series of events in which a stimulus from a bacterium is received and converted into a molecular signal. |
| GO:0016049 | 1 | Cell growth | Biological Process | The process in which a cell irreversibly increases in size over time by accretion and biosynthetic production of matter similar to that already present. |
| GO:0016055 | 1 | Wnt signaling pathway | Biological Process | The series of molecular signals initiated by binding of a Wnt protein to a frizzled family receptor on the surface of the target cell and ending with a change in cell state. |
| GO:0016114 | 2 | Terpenoid biosynthetic process | Biological Process | The chemical reactions and pathways resulting in the formation of terpenoids, any member of a class of compounds characterized by an isoprenoid chemical structure. |
| GO:0016117 | 2 | Carotenoid biosynthetic process | Biological Process | The chemical reactions and pathways resulting in the formation of carotenoids, tetraterpenoid compounds in which two units of 4 isoprenoid residues joined head-to-tail are themselves joined tail-to-tail. |
| GO:0016165 | 1 | Linoleate 13S-lipoxygenase activity | Molecular Function | Catalysis of the reaction: linoleate + O2 = (9Z,11E)-(13S)-13-hydroperoxyoctadeca-9,11-dienoate. |
| GO:0016168 | 4 | Chlorophyll binding | Molecular Function | Interacting selectively and non-covalently with chlorophyll; any compound of magnesium complexed in a porphyrin (tetrapyrrole) ring and which functions as a photosynthetic pigment. |
| GO:0016174 | 2 | NAD(P)H oxidase activity | Molecular Function | Catalysis of the reaction: NAD(P)H + H+ + O2 = NAD(P)+ + hydrogen peroxide. |
| GO:0016192 | 1 | Vesicle-mediated transport | Biological Process | A cellular transport process in which transported substances are moved in membrane-bounded vesicles; transported substances are enclosed in the vesicle lumen or located in the vesicle membrane. The process begins with a step that directs a substance to the forming vesicle, and includes vesicle budding and coating. Vesicles are then targeted to, and fuse with, an acceptor membrane. |
| GO:0016226 | 1 | Iron-sulfur cluster assembly | Biological Process | The incorporation of iron and exogenous sulfur into a metallo-sulfur cluster. |
| GO:0016255 | 1 | Attachment of GPI anchor to protein | Biological Process | A transamidation reaction that results in the cleavage of the polypeptide chain and the concomitant transfer of the GPI anchor to the newly formed carboxy-terminal amino acid of the anchored protein. The cleaved C-terminal contains the C-terminal GPI signal sequence of the newly synthesized polypeptide chain. |
| GO:0016297 | 2 | Acyl-[acyl-carrier-protein] hydrolase activity | Molecular Function | Catalysis of the reaction: acyl-[acyl-carrier protein] + H2O = [acyl-carrier protein] + a fatty acid. |
| GO:0016298 | 2 | Lipase activity | Molecular Function | Catalysis of the hydrolysis of a lipid or phospholipid. |
| GO:0016301 | 10 | Kinase activity | Molecular Function | Catalysis of the transfer of a phosphate group, usually from ATP, to a substrate molecule. |
| GO:0016307 | 1 | Phosphatidylinositol phosphate kinase activity | Molecular Function | Catalysis of the reaction: ATP + a phosphatidylinositol phosphate = ADP + a phosphatidylinositol |
| GO:0016308 | 1 | 1-phosphatidylinositol-4-phosphate 5-kinase activity | Molecular Function | Catalysis of the reaction: 1-phosphatidyl-1D-myo-inositol 4-phosphate + ATP = 1-phosphatidyl-1D-myo-inositol 4,5-bisphosphate + ADP + 2 H(+). |
| GO:0016310 | 16 | Phosphorylation | Biological Process | The process of introducing a phosphate group into a molecule, usually with the formation of a phosphoric ester, a phosphoric anhydride or a phosphoric amide. |
| GO:0016311 | 1 | Dephosphorylation | Biological Process | The process of removing one or more phosphoric (ester or anhydride) residues from a molecule. |
| GO:0016314 | 1 | Phosphatidylinositol-3,4,5-trisphosphate 3-phosphatase activity | Molecular Function | Catalysis of the reaction: phosphatidylinositol-3,4,5-trisphosphate + H2O = phosphatidylinositol-4,5-bisphosphate + phosphate. |
| GO:0016413 | 2 | O-acetyltransferase activity | Molecular Function | Catalysis of the transfer of an acetyl group to an oxygen atom on the acceptor molecule. |
| GO:0016459 | 1 | Myosin complex | Cellular Component | A protein complex, formed of one or more myosin heavy chains plus associated light chains and other proteins, that functions as a molecular motor; uses the energy of ATP hydrolysis to move actin filaments or to move vesicles or other cargo on fixed actin filaments; has magnesium-ATPase activity and binds actin. Myosin classes are distinguished based on sequence features of the motor, or head, domain, but also have distinct tail regions that are believed to bind specific cargoes. |
| GO:0016491 | 22 | Oxidoreductase activity | Molecular Function | Catalysis of an oxidation-reduction (redox) reaction, a reversible chemical reaction in which the oxidation state of an atom or atoms within a molecule is altered. One substrate acts as a hydrogen or electron donor and becomes oxidized, while the other acts as hydrogen or electron acceptor and becomes reduced. |
| GO:0016567 | 8 | Protein ubiquitination | Biological Process | The process in which one or more ubiquitin groups are added to a protein. |
| GO:0016573 | 1 | Histone acetylation | Biological Process | The modification of a histone by the addition of an acetyl group. |
| GO:0016592 | 2 | Mediator complex | Cellular Component | A protein complex that interacts with the carboxy-terminal domain of the largest subunit of RNA polymerase II and plays an active role in transducing the signal from a transcription factor to the transcriptional machinery. The mediator complex is required for activation of transcription of most protein-coding genes, but can also act as a transcriptional corepressor. The Saccharomyces complex contains several identifiable subcomplexes: a head domain comprising Srb2, -4, and -5, Med6, -8, and -11, and Rox3 proteins; a middle domain comprising Med1, -4, and -7, Nut1 and -2, Cse2, Rgr1, Soh1, and Srb7 proteins; a tail consisting of Gal11p, Med2p, Pgd1p, and Sin4p; and a regulatory subcomplex comprising Ssn2, -3, and -8, and Srb8 proteins. Metazoan mediator complexes have similar modular structures and include homologs of yeast Srb and Med proteins. |
| GO:0016598 | 1 | Protein arginylation | Biological Process | The conjugation of arginine to the N-terminal aspartate or glutamate of a protein; required for the degradation of the protein via the ubiquitin pathway. |
| GO:0016602 | 2 | CCAAT-binding factor complex | Cellular Component | A heteromeric transcription factor complex that binds to the CCAAT-box upstream of promoters; functions as both an activator and a repressor, depending on its interacting cofactors. Typically trimeric consisting of NFYA, NFYB and NFYC subunits. In Saccharomyces, it activates the transcription of genes in response to growth in a nonfermentable carbon source and consists of four known subunits: HAP2, HAP3, HAP4 and HAP5. |
| GO:0016614 | 1 | Oxidoreductase activity, acting on CH-OH group of donors | Molecular Function | Catalysis of an oxidation-reduction (redox) reaction in which a CH-OH group act as a hydrogen or electron donor and reduces a hydrogen or electron acceptor. |
| GO:0016616 | 2 | Oxidoreductase activity, acting on the CH-OH group of donors, NAD or NADP as acceptor | Molecular Function | Catalysis of an oxidation-reduction (redox) reaction in which a CH-OH group acts as a hydrogen or electron donor and reduces NAD+ or NADP. |
| GO:0016627 | 1 | Oxidoreductase activity, acting on the CH-CH group of donors | Molecular Function | Catalysis of an oxidation-reduction (redox) reaction in which a CH-CH group acts as a hydrogen or electron donor and reduces a hydrogen or electron acceptor. |
| GO:0016635 | 1 | Oxidoreductase activity, acting on the CH-CH group of donors, quinone or related compound as acceptor | Molecular Function | Catalysis of an oxidation-reduction (redox) reaction in which a CH-CH group acts as a hydrogen or electron donor and reduces a quinone or related compound. |
| GO:0016639 | 1 | Oxidoreductase activity, acting on the CH-NH2 group of donors, NAD or NADP as acceptor | Molecular Function | Catalysis of an oxidation-reduction (redox) reaction in which a CH-NH2 group acts as a hydrogen or electron donor and reduces NAD+ or NADP. |
| GO:0016651 | 2 | Oxidoreductase activity, acting on NAD(P)H | Molecular Function | Catalysis of an oxidation-reduction (redox) reaction in which NADH or NADPH acts as a hydrogen or electron donor and reduces a hydrogen or electron acceptor. |
| GO:0016671 | 1 | Oxidoreductase activity, acting on a sulfur group of donors, disulfide as acceptor | Molecular Function | Catalysis of an oxidation-reduction (redox) reaction in which a sulfur-containing group acts as a hydrogen or electron donor and reduces disulfide. |
| GO:0016702 | 3 | Oxidoreductase activity, acting on single donors with incorporation of molecular oxygen, incorporation of two atoms of oxygen | Molecular Function | Catalysis of an oxidation-reduction (redox) reaction in which hydrogen or electrons are transferred from one donor, and two oxygen atoms is incorporated into a donor. |
| GO:0016705 | 15 | Oxidoreductase activity, acting on paired donors, with incorporation or reduction of molecular oxygen | Molecular Function | Catalysis of an oxidation-reduction (redox) reaction in which hydrogen or electrons are transferred from each of two donors, and molecular oxygen is reduced or incorporated into a donor. |
| GO:0016709 | 3 | Oxidoreductase activity, acting on paired donors, with incorporation or reduction of molecular oxygen, NAD(P)H as one donor, and incorporation of one atom of oxygen | Molecular Function | Catalysis of an oxidation-reduction (redox) reaction in which hydrogen or electrons are transferred from NADH or NADPH and one other donor, and one atom of oxygen is incorporated into one donor. |
| GO:0016740 | 17 | Transferase activity | Molecular Function | Catalysis of the transfer of a group, e.g. a methyl group, glycosyl group, acyl group, phosphorus-containing, or other groups, from one compound (generally regarded as the donor) to another compound (generally regarded as the acceptor). Transferase is the systematic name for any enzyme of EC class 2. |
| GO:0016746 | 5 | Transferase activity, transferring acyl groups | Molecular Function | Catalysis of the transfer of an acyl group from one compound (donor) to another (acceptor). |
| GO:0016747 | 7 | Transferase activity, transferring acyl groups other than amino-acyl groups | Molecular Function | Catalysis of the transfer of an acyl group, other than amino-acyl, from one compound (donor) to another (acceptor). |
| GO:0016757 | 2 | Transferase activity, transferring glycosyl groups | Molecular Function | Catalysis of the transfer of a glycosyl group from one compound (donor) to another (acceptor). |
| GO:0016758 | 2 | Transferase activity, transferring hexosyl groups | Molecular Function | Catalysis of the transfer of a hexosyl group from one compound (donor) to another (acceptor). |
| GO:0016760 | 6 | Cellulose synthase (UDP-forming) activity | Molecular Function | Catalysis of the reaction: UDP-glucose + ((1,4)-beta-D-glucosyl)(n) = UDP + ((1,4)-beta-D-glucosyl)(n+1). |
| GO:0016765 | 2 | Transferase activity, transferring alkyl or aryl (other than methyl) groups | Molecular Function | Catalysis of the transfer of an alkyl or aryl (but not methyl) group from one compound (donor) to another (acceptor). |
| GO:0016773 | 1 | Phosphotransferase activity, alcohol group as acceptor | Molecular Function | Catalysis of the transfer of a phosphorus-containing group from one compound (donor) to an alcohol group (acceptor). |
| GO:0016779 | 2 | Nucleotidyltransferase activity | Molecular Function | Catalysis of the transfer of a nucleotidyl group to a reactant. |
| GO:0016787 | 30 | Hydrolase activity | Molecular Function | Catalysis of the hydrolysis of various bonds, e.g. C-O, C-N, C-C, phosphoric anhydride bonds, etc. Hydrolase is the systematic name for any enzyme of EC class 3. |
| GO:0016788 | 2 | Hydrolase activity, acting on ester bonds | Molecular Function | Catalysis of the hydrolysis of any ester bond. |
| GO:0016791 | 1 | Phosphatase activity | Molecular Function | Catalysis of the hydrolysis of phosphoric monoesters, releasing inorganic phosphate. |
| GO:0016798 | 2 | Hydrolase activity, acting on glycosyl bonds | Molecular Function | Catalysis of the hydrolysis of any glycosyl bond. |
| GO:0016813 | 1 | Hydrolase activity, acting on carbon-nitrogen (but not peptide) bonds, in linear amidines | Molecular Function | Catalysis of the hydrolysis of any non-peptide carbon-nitrogen bond in a linear amidine, a compound of the form R-C(=NH)-NH2. |
| GO:0016818 | 1 | Hydrolase activity, acting on acid anhydrides, in phosphorus-containing anhydrides | Molecular Function | Catalysis of the hydrolysis of any acid anhydride which contains phosphorus. |
| GO:0016829 | 4 | Lyase activity | Molecular Function | Catalysis of the cleavage of C-C, C-O, C-N and other bonds by other means than by hydrolysis or oxidation, or conversely adding a group to a double bond. They differ from other enzymes in that two substrates are involved in one reaction direction, but only one in the other direction. When acting on the single substrate, a molecule is eliminated and this generates either a new double bond or a new ring. |
| GO:0016831 | 1 | Carboxy-lyase activity | Molecular Function | Catalysis of the nonhydrolytic addition or removal of a carboxyl group to or from a compound. |
| GO:0016832 | 1 | Aldehyde-lyase activity | Molecular Function | Catalysis of the cleavage of a C-C bond in a molecule containing a hydroxyl group and a carbonyl group to form two smaller molecules, each being an aldehyde or a ketone. |
| GO:0016853 | 2 | Isomerase activity | Molecular Function | Catalysis of the geometric or structural changes within one molecule. Isomerase is the systematic name for any enzyme of EC class 5. |
| GO:0016866 | 1 | Intramolecular transferase activity | Molecular Function | Catalysis of the transfer of a functional group from one position to another within a single molecule. |
| GO:0016871 | 1 | Cycloartenol synthase activity | Molecular Function | Catalysis of the reaction: (S)-2,3-epoxysqualene = cycloartenol. |
| GO:0016874 | 12 | Ligase activity | Molecular Function | Catalysis of the joining of two substances, or two groups within a single molecule, with the concomitant hydrolysis of the diphosphate bond in ATP or a similar triphosphate. |
| GO:0016887 | 6 | ATPase activity | Molecular Function | Catalysis of the reaction: ATP + H2O = ADP + phosphate + 2 H+. May or may not be coupled to another reaction. |
| GO:0016925 | 1 | Protein sumoylation | Biological Process | The process in which a SUMO protein (small ubiquitin-related modifier) is conjugated to a target protein via an isopeptide bond between the carboxyl terminus of SUMO with an epsilon-amino group of a lysine residue of the target protein. |
| GO:0016984 | 1 | Ribulose-bisphosphate carboxylase activity | Molecular Function | Catalysis of the reaction: D-ribulose 1,5-bisphosphate + CO2 + H2O = 2 3-phospho-D-glycerate. |
| GO:0017004 | 3 | Cytochrome complex assembly | Biological Process | The aggregation, arrangement and bonding together of a cytochrome complex. A cytochrome complex is a protein complex in which at least one of the proteins is a cytochrome, i.e. a heme-containing protein involved in catalysis of redox reactions. |
| GO:0017089 | 1 | Glycolipid transporter activity | Molecular Function | Enables the directed movement of glycolipids, compounds containing (usually) 1-4 linked monosaccharide residues joined by a glycosyl linkage to a lipid, into, out of or within a cell, or between cells. |
| GO:0017119 | 1 | Golgi transport complex | Cellular Component | A multisubunit tethering complex of the CATCHR family (complexes associated with tethering containing helical rods) that has a role in tethering vesicles to the Golgi prior to fusion. In yeast, this complex is called the Sec34/35 complex and is composed of eight subunits (Sec34p, Sec35p, Dor1p, Cod1p, Cod2p, Cod3p, Cod4p, and Cod5p). In mammals the subunits are named COG1-8. |
| GO:0017183 | 1 | Peptidyl-diphthamide biosynthetic process from peptidyl-histidine | Biological Process | The modification of peptidyl-histidine to 2'-(3-carboxamido-3-(trimethylammonio)propyl)-L-histidine, known as diphthamide, found in translation elongation factor EF-2. The process occurs in eukaryotes and archaea but not eubacteria. |
| GO:0017198 | 1 | N-terminal peptidyl-serine acetylation | Biological Process | The acetylation of the N-terminal serine of proteins to form the derivative N-acetyl-L-serine. |
| GO:0018002 | 1 | N-terminal peptidyl-glutamic acid acetylation | Biological Process | The acetylation of the N-terminal glutamic acid of proteins to form the derivate acetyl-glutamic acid. |
| GO:0018105 | 1 | Peptidyl-serine phosphorylation | Biological Process | The phosphorylation of peptidyl-serine to form peptidyl-O-phospho-L-serine. |
| GO:0018108 | 8 | Peptidyl-tyrosine phosphorylation | Biological Process | The phosphorylation of peptidyl-tyrosine to form peptidyl-O4'-phospho-L-tyrosine. |
| GO:0018171 | 2 | Peptidyl-cysteine oxidation | Biological Process | The oxidation of peptidyl-cysteine to peptidyl-L-cysteine sulfinic acid or peptidyl-L-cysteine sulfenic acid. |
| GO:0018298 | 3 | Protein-chromophore linkage | Biological Process | The covalent or noncovalent attachment of a chromophore to a protein. |
| GO:0019013 | 1 | Viral nucleocapsid | Cellular Component | The complete protein-nucleic acid complex that is the packaged form of the genome in a virus particle. |
| GO:0019172 | 1 | Glyoxalase III activity | Molecular Function | Catalysis of the reaction: methylglyoxal + H2O = D-lactate. |
| GO:0019243 | 1 | Methylglyoxal catabolic process to D-lactate via S-lactoyl-glutathione | Biological Process | The chemical reactions and pathways resulting in the breakdown of methylglyoxal, CH3-CO-CHO, into D-lactate via the intermediate S-lactoyl-glutathione. Glutathione is used in the first step of the pathway and then regenerated in the second step. |
| GO:0019249 | 1 | Lactate biosynthetic process | Biological Process | The chemical reactions and pathways resulting in the formation of lactate, the anion of lactic acid. |
| GO:0019252 | 2 | Starch biosynthetic process | Biological Process | The chemical reactions and pathways resulting in the formation of starch, the most important reserve polysaccharide in plants. |
| GO:0019279 | 1 | L-methionine biosynthetic process from L-homoserine via cystathionine | Biological Process | The chemical reactions and pathways resulting in the formation of L-methionine from other compounds, including L-homoserine, via the intermediate cystathionine. |
| GO:0019294 | 1 | Keto-3-deoxy-D-manno-octulosonic acid biosynthetic process | Biological Process | The chemical reactions and pathways resulting in the formation of keto-3-deoxy-D-manno-octulosonic acid, an acidic sugar present in lipopolysaccharides of the outer membranes of some Gram-negative bacteria. |
| GO:0019310 | 6 | Inositol catabolic process | Biological Process | The chemical reactions and pathways resulting in the breakdown of inositol, 1,2,3,4,5,6-cyclohexanehexol, a growth factor for animals and microorganisms. |
| GO:0019343 | 1 | Cysteine biosynthetic process via cystathionine | Biological Process | The chemical reactions and pathways resulting in the formation of cysteine, via the intermediate cystathionine. |
| GO:0019346 | 1 | Transsulfuration | Biological Process | The interconversion of homocysteine and cysteine via cystathionine. In contrast with enteric bacteria and mammals, Saccharomyces cerevisiae has two transsulfuration pathways employing two separate sets of enzymes. |
| GO:0019388 | 1 | Galactose catabolic process | Biological Process | The chemical reactions and pathways resulting in the breakdown of galactose, the aldohexose galacto-hexose. |
| GO:0019430 | 1 | Removal of superoxide radicals | Biological Process | Any process, acting at the cellular level, involved in removing superoxide radicals (O2-) from a cell or organism, e.g. by conversion to dioxygen (O2) and hydrogen peroxide (H2O2). |
| GO:0019499 | 1 | Cyanide metabolic process | Biological Process | The chemical reactions and pathways involving cyanide, NC-, the anion of hydrocyanic acid. Cyanide is a potent inhibitor of respiration, reacting with the ferric form of cytochrome aa3 and thus blocking the electron transport chain. |
| GO:0019684 | 3 | Photosynthesis, light reaction | Biological Process | The light reactions of photosynthesis, which take place in photosystems II and I. Light energy is harvested and used to power the transfer of electrons among a series of electron donors and acceptors. The final electron acceptor is NADP+, which is reduced to NADPH. NADPH generated from light reactions is used in sugar synthesis in dark reactions. Light reactions also generate a proton motive force across the thylakoid membrane, and the proton gradient is used to synthesize ATP. There are two chemical reactions involved in the light reactions: water oxidation in photosystem II, and NADP reduction in photosystem I. |
| GO:0019745 | 2 | Pentacyclic triterpenoid biosynthetic process | Biological Process | The chemical reactions and pathways resulting in the formation of pentacyclic triterpenoid compounds, terpenoids with six isoprene units and 5 carbon rings. |
| GO:0019748 | 1 | Secondary metabolic process | Biological Process | The chemical reactions and pathways resulting in many of the chemical changes of compounds that are not necessarily required for growth and maintenance of cells, and are often unique to a taxon. In multicellular organisms secondary metabolism is generally carried out in specific cell types, and may be useful for the organism as a whole. In unicellular organisms, secondary metabolism is often used for the production of antibiotics or for the utilization and acquisition of unusual nutrients. |
| GO:0019752 | 1 | Carboxylic acid metabolic process | Biological Process | The chemical reactions and pathways involving carboxylic acids, any organic acid containing one or more carboxyl (COOH) groups or anions (COO-). |
| GO:0019789 | 1 | SUMO transferase activity | Molecular Function | Catalysis of the transfer of SUMO from one protein to another via the reaction X-SUMO + Y --> Y-SUMO + X, where both X-SUMO and Y-SUMO are covalent linkages. |
| GO:0019843 | 17 | rRNA binding | Molecular Function | Interacting selectively and non-covalently with ribosomal RNA. |
| GO:0019888 | 2 | Protein phosphatase regulator activity | Molecular Function | Modulates the activity of a protein phosphatase, an enzyme which catalyzes of the removal of a phosphate group from a protein substrate molecule. |
| GO:0019898 | 1 | Extrinsic component of membrane | Cellular Component | The component of a membrane consisting of gene products and protein complexes that are loosely bound to one of its surfaces, but not integrated into the hydrophobic region. |
| GO:0019953 | 1 | Sexual reproduction | Biological Process | A reproduction process that creates a new organism by combining the genetic material of two organisms. It occurs both in eukaryotes and prokaryotes: in multicellular eukaryotic organisms, an individual is created anew; in prokaryotes, the initial cell has additional or transformed genetic material. In a process called genetic recombination, genetic material (DNA) originating from two different individuals (parents) join up so that homologous sequences are aligned with each other, and this is followed by exchange of genetic information. After the new recombinant chromosome is formed, it is passed on to progeny. |
| GO:0020037 | 22 | Heme binding | Molecular Function | Interacting selectively and non-covalently with heme, any compound of iron complexed in a porphyrin (tetrapyrrole) ring. |
| GO:0022618 | 1 | Ribonucleoprotein complex assembly | Biological Process | The aggregation, arrangement and bonding together of proteins and RNA molecules to form a ribonucleoprotein complex. |
| GO:0022625 | 4 | Cytosolic large ribosomal subunit | Cellular Component | The large subunit of a ribosome located in the cytosol. |
| GO:0022626 | 1 | Cytosolic ribosome | Cellular Component | A ribosome located in the cytosol. |
| GO:0022627 | 5 | Cytosolic small ribosomal subunit | Cellular Component | The small subunit of a ribosome located in the cytosol. |
| GO:0022857 | 5 | Transmembrane transporter activity | Molecular Function | Enables the transfer of a substance from one side of a membrane to the other. |
| GO:0022891 | 13 | Substrate-specific transmembrane transporter activity | Molecular Function | Enables the transfer of a specific substance or group of related substances from one side of a membrane to the other. |
| GO:0022900 | 2 | Electron transport chain | Biological Process | A process in which a series of electron carriers operate together to transfer electrons from donors to any of several different terminal electron acceptors to generate a transmembrane electrochemical gradient. |
| GO:0023014 | 1 | Signal transduction by protein phosphorylation | Biological Process | A process in which the transfer of one or more phosphate groups to a substrate transmits a signal to the phosphorylated substrate. |
| GO:0030001 | 2 | Metal ion transport | Biological Process | The directed movement of metal ions, any metal ion with an electric charge, into, out of or within a cell, or between cells, by means of some agent such as a transporter or pore. |
| GO:0030026 | 1 | Cellular manganese ion homeostasis | Biological Process | Any process involved in the maintenance of an internal steady state of manganese ions at the level of a cell. |
| GO:0030117 | 1 | Membrane coat | Cellular Component | Any of several different proteinaceous coats that can associate with membranes. Membrane coats include those formed by clathrin plus an adaptor complex, the COPI and COPII complexes, and possibly others. They are found associated with membranes on many vesicles as well as other membrane features such as pits and perhaps tubules. |
| GO:0030145 | 2 | Manganese ion binding | Molecular Function | Interacting selectively and non-covalently with manganese (Mn) ions. |
| GO:0030151 | 1 | Molybdenum ion binding | Molecular Function | Interacting selectively and non-covalently with molybdenum (Mo) ions. |
| GO:0030163 | 3 | Protein catabolic process | Biological Process | The chemical reactions and pathways resulting in the breakdown of a protein by the destruction of the native, active configuration, with or without the hydrolysis of peptide bonds. |
| GO:0030170 | 6 | Pyridoxal phosphate binding | Molecular Function | Interacting selectively and non-covalently with pyridoxal 5' phosphate, 3-hydroxy-5-(hydroxymethyl)-2-methyl4-pyridine carboxaldehyde 5' phosphate, the biologically active form of vitamin B6. |
| GO:0030176 | 3 | Integral component of endoplasmic reticulum membrane | Cellular Component | The component of the endoplasmic reticulum membrane consisting of the gene products and protein complexes having at least some part of their peptide sequence embedded in the hydrophobic region of the membrane. |
| GO:0030244 | 6 | Cellulose biosynthetic process | Biological Process | The chemical reactions and pathways resulting in the formation of cellulose, a linear beta1-4 glucan of molecular mass 50-400 kDa with the pyranose units in the -4C1 conformation. |
| GO:0030246 | 3 | Carbohydrate binding | Molecular Function | Interacting selectively and non-covalently with any carbohydrate, which includes monosaccharides, oligosaccharides and polysaccharides as well as substances derived from monosaccharides by reduction of the carbonyl group (alditols), by oxidation of one or more hydroxy groups to afford the corresponding aldehydes, ketones, or carboxylic acids, or by replacement of one or more hydroxy group(s) by a hydrogen atom. Cyclitols are generally not regarded as carbohydrates. |
| GO:0030247 | 2 | Polysaccharide binding | Molecular Function | Interacting selectively and non-covalently with any polysaccharide, a polymer of many (typically more than 10) monosaccharide residues linked glycosidically. |
| GO:0030490 | 2 | Maturation of SSU-rRNA | Biological Process | Any process involved in the maturation of a precursor Small SubUnit (SSU) ribosomal RNA (rRNA) molecule into a mature SSU-rRNA molecule. |
| GO:0030570 | 2 | Pectate lyase activity | Molecular Function | Catalysis of the reaction: a pectate = a pectate + a pectate oligosaccharide with 4-(4-deoxy-alpha-D-galact-4-enuronosyl)-D-galacturonate end. This reaction is the eliminative cleavage of pectate to give oligosaccharides with 4-deoxy-alpha-D-gluc-4-enuronosyl groups at their non-reducing ends. |
| GO:0030599 | 8 | Pectinesterase activity | Molecular Function | Catalysis of the reaction: pectin + n H2O = n methanol + pectate. |
| GO:0030626 | 1 | U12 snRNA binding | Molecular Function | Interacting selectively and non-covalently with the U12 small nuclear RNA (U12 snRNA). |
| GO:0030660 | 3 | Golgi-associated vesicle membrane | Cellular Component | The lipid bilayer surrounding a vesicle associated with the Golgi apparatus. |
| GO:0030663 | 1 | COPI-coated vesicle membrane | Cellular Component | The lipid bilayer surrounding a COPI-coated vesicle. |
| GO:0030686 | 1 | 90S preribosome | Cellular Component | A large ribonucleoprotein complex considered to be the earliest preribosomal complex. In S. cerevisiae, it has a size of 90S and consists of the 35S pre-rRNA, early-associating ribosomal proteins most of which are part of the small ribosomal subunit, the U3 snoRNA and associated proteins. |
| GO:0030785 | 1 | [ribulose-bisphosphate carboxylase]-lysine N-methyltransferase activity | Molecular Function | Catalysis of the reaction: S-adenosyl-L-methionine + [ribulose-1,5-bisphosphate carboxylase]-lysine = S-adenosyl-L-homocysteine + [ribulose-1,5-bisphosphate carboxylase]-N6-methyl-L-lysine. |
| GO:0030904 | 1 | Retromer complex | Cellular Component | A conserved hetero-pentameric membrane-associated complex involved in retrograde transport from endosomes to the Golgi apparatus. The budding yeast retromer comprises Vps35p, Vps29p, Vps26p, Vps5p, and Vps17p. The mammalian complex shows slight variation in composition compared to yeast, and comprises SNX1 or SNX2, SNX5 or SNX6, VPS26A or VPS26B, VPS29, and VPS35. |
| GO:0030942 | 1 | Endoplasmic reticulum signal peptide binding | Molecular Function | Interacting selectively and non-covalently with an endoplasmic reticulum signal peptide, a specific peptide sequence that acts as a signal to localize the protein within the endoplasmic reticulum. |
| GO:0030955 | 1 | Potassium ion binding | Molecular Function | Interacting selectively and non-covalently with potassium (K+) ions. |
| GO:0030956 | 1 | Glutamyl-tRNA(Gln) amidotransferase complex | Cellular Component | A protein complex that possesses glutamyl-tRNA(Gln) amidotransferase activity, and therefore creates Gln-tRNA by amidating Glu-tRNA; usually composed of 3 subunits: A, B, and C. Note that the C subunit may not be required in all organisms. |
| GO:0031087 | 1 | Deadenylation-independent decapping of nuclear-transcribed mRNA | Biological Process | Cleavage of the 5'-cap of a nuclear-transcribed mRNA that is independent of poly(A) tail shortening. |
| GO:0031122 | 4 | Cytoplasmic microtubule organization | Biological Process | A process that is carried out at the cellular level which results in the assembly, arrangement of constituent parts, or disassembly of structures formed of microtubules and associated proteins in the cytoplasm of a cell. |
| GO:0031167 | 1 | rRNA methylation | Biological Process | The posttranscriptional addition of methyl groups to specific residues in an rRNA molecule. |
| GO:0031177 | 1 | Phosphopantetheine binding | Molecular Function | Interacting selectively and non-covalently with phosphopantetheine, the vitamin pantetheine 4'-(dihydrogen phosphate). |
| GO:0031201 | 2 | SNARE complex | Cellular Component | A protein complex involved in membrane fusion; a stable ternary complex consisting of a four-helix bundle, usually formed from one R-SNARE and three Q-SNAREs with an ionic layer sandwiched between hydrophobic layers. One well-characterized example is the neuronal SNARE complex formed of synaptobrevin 2, syntaxin 1a, and SNAP-25. |
| GO:0031347 | 1 | Regulation of defense response | Biological Process | Any process that modulates the frequency, rate or extent of a defense response. |
| GO:0031349 | 1 | Positive regulation of defense response | Biological Process | Any process that activates or increases the frequency, rate or extent of a defense response. |
| GO:0031369 | 1 | Translation initiation factor binding | Molecular Function | Interacting selectively and non-covalently with a translation initiation factor, any polypeptide factor involved in the initiation of ribosome-mediated translation. |
| GO:0031408 | 1 | Oxylipin biosynthetic process | Biological Process | The chemical reactions and pathways resulting in the formation of any oxylipin, any of a group of biologically active compounds formed by oxidative metabolism of polyunsaturated fatty acids. |
| GO:0031415 | 1 | NatA complex | Cellular Component | A conserved complex that catalyzes the transfer of an acetyl group to an N-terminal Ser, Ala, Gly, or Thr residue of a protein acceptor molecule. In Saccharomyces the complex includes Nat1p and Ard1p, and may contain additional proteins. |
| GO:0031418 | 1 | L-ascorbic acid binding | Molecular Function | Interacting selectively and non-covalently with L-ascorbic acid, (2R)-2-[(1S)-1,2-dihydroxyethyl]-4-hydroxy-5-oxo-2,5-dihydrofuran-3-olate; L-ascorbic acid is vitamin C and has co-factor and anti-oxidant activities in many species. |
| GO:0031461 | 2 | Cullin-RING ubiquitin ligase complex | Cellular Component | Any ubiquitin ligase complex in which the catalytic core consists of a member of the cullin family and a RING domain protein; the core is associated with one or more additional proteins that confer substrate specificity. |
| GO:0031463 | 1 | Cul3-RING ubiquitin ligase complex | Cellular Component | A ubiquitin ligase complex in which a cullin from the Cul3 subfamily and a RING domain protein form the catalytic core; substrate specificity is conferred by a BTB-domain-containing protein. |
| GO:0031507 | 1 | Heterochromatin assembly | Biological Process | The assembly of chromatin into heterochromatin, a compact and highly condensed form that is often, but not always, transcriptionally silent. |
| GO:0031514 | 1 | Motile cilium | Cellular Component | A cilium which may have a variable arrangement of axonemal microtubules and also contains molecular motors. It may beat with a whip-like pattern that promotes cell motility or transport of fluids and other cells across a cell surface, such as on epithelial cells that line the lumenal ducts of various tissues; or they may display a distinct twirling motion that directs fluid flow asymmetrically across the cellular surface to affect asymmetric body plan organization. Motile cilia can be found in single as well as multiple copies per cell. |
| GO:0031624 | 3 | Ubiquitin conjugating enzyme binding | Molecular Function | Interacting selectively and non-covalently with a ubiquitin conjugating enzyme, any of the E2 proteins. |
| GO:0031625 | 5 | Ubiquitin protein ligase binding | Molecular Function | Interacting selectively and non-covalently with a ubiquitin protein ligase enzyme, any of the E3 proteins. |
| GO:0031668 | 1 | Cellular response to extracellular stimulus | Biological Process | Any process that results in a change in state or activity of a cell (in terms of movement, secretion, enzyme production, gene expression, etc.) as a result of an extracellular stimulus. |
| GO:0032040 | 1 | Small-subunit processome | Cellular Component | A large ribonucleoprotein complex that is an early preribosomal complex. In S. cerevisiae, it has a size of 80S and consists of the 35S pre-rRNA, early-associating ribosomal proteins most of which are part of the small ribosomal subunit, the U3 snoRNA and associated proteins. |
| GO:0032182 | 2 | Ubiquitin-like protein binding | Molecular Function | Interacting selectively and non-covalently with a small conjugating protein such as ubiquitin or a ubiquitin-like protein. |
| GO:0032259 | 10 | Methylation | Biological Process | The process in which a methyl group is covalently attached to a molecule. |
| GO:0032264 | 2 | IMP salvage | Biological Process | Any process which produces inosine monophosphate from derivatives of it, without de novo synthesis. |
| GO:0032436 | 1 | Positive regulation of proteasomal ubiquitin-dependent protein catabolic process | Biological Process | Any process that activates or increases the frequency, rate or extent of the breakdown of a protein or peptide by hydrolysis of its peptide bonds, initiated by the covalent attachment of ubiquitin, and mediated by the proteasome. |
| GO:0032440 | 3 | 2-alkenal reductase [NAD(P)] activity | Molecular Function | Catalysis of the reaction: n-alkanal + NAD(P)+ = alk-2-enal + NAD(P)H + H+. |
| GO:0032508 | 2 | DNA duplex unwinding | Biological Process | The process in which interchain hydrogen bonds between two strands of DNA are broken or 'melted', generating a region of unpaired single strands. |
| GO:0032543 | 1 | Mitochondrial translation | Biological Process | The chemical reactions and pathways resulting in the formation of a protein in a mitochondrion. This is a ribosome-mediated process in which the information in messenger RNA (mRNA) is used to specify the sequence of amino acids in the protein; the mitochondrion has its own ribosomes and transfer RNAs, and uses a genetic code that differs from the nuclear code. |
| GO:0032549 | 2 | Ribonucleoside binding | Molecular Function | Interacting selectively and non-covalently with a ribonucleoside, a compound consisting of a purine or pyrimidine nitrogenous base linked to ribose. |
| GO:0032550 | 1 | Purine ribonucleoside binding | Molecular Function | Interacting selectively and non-covalently with a purine ribonucleoside, a compound consisting of a purine base linked to ribose. |
| GO:0032559 | 1 | Adenyl ribonucleotide binding | Molecular Function | Interacting selectively and non-covalently with an adenyl ribonucleotide, any compound consisting of adenosine esterified with (ortho)phosphate or an oligophosphate at any hydroxyl group on the ribose moiety. |
| GO:0032587 | 1 | Ruffle membrane | Cellular Component | The portion of the plasma membrane surrounding a ruffle. |
| GO:0032807 | 2 | DNA ligase IV complex | Cellular Component | A eukaryotically conserved protein complex that contains DNA ligase IV and is involved in DNA repair by non-homologous end joining; in addition to the ligase, the complex also contains XRCC4 or a homolog, e.g. Saccharomyces Lif1p. |
| GO:0032968 | 1 | Positive regulation of transcription elongation from RNA polymerase II promoter | Biological Process | Any process that activates or increases the frequency, rate or extent of transcription elongation, the extension of an RNA molecule after transcription initiation and promoter clearance by the addition of ribonucleotides, catalyzed by RNA polymerase II. |
| GO:0033177 | 1 | Proton-transporting two-sector ATPase complex, proton-transporting domain | Cellular Component | A protein complex that forms part of a proton-transporting two-sector ATPase complex and carries out proton transport across a membrane. The proton-transporting domain (F0, V0, or A0) includes integral and peripheral membrane proteins. |
| GO:0033609 | 1 | Oxalate metabolic process | Biological Process | The chemical reactions and pathways involving oxalate, the organic acid ethanedioate. |
| GO:0033619 | 3 | Membrane protein proteolysis | Biological Process | The proteolytic cleavage of a transmembrane protein leading to the release of its intracellular or ecto-domains. |
| GO:0033897 | 1 | Ribonuclease T2 activity | Molecular Function | Catalysis of the two-stage endonucleolytic cleavage to nucleoside 3'-phosphates and 3'-phosphooligonucleotides with 2',3'-cyclic phosphate intermediates. |
| GO:0034219 | 1 | Carbohydrate transmembrane transport | Biological Process | The process in which a carbohydrate is transported from one side of a membrane to the other by means of some agent such as a transporter or pore. |
| GO:0034399 | 2 | Nuclear periphery | Cellular Component | The portion of the nuclear lumen proximal to the inner nuclear membrane. |
| GO:0034457 | 1 | Mpp10 complex | Cellular Component | A protein complex that forms a subcomplex of the 90S preribosome. In S. cerevisiae, it is composed of Mpp10p, Imp3p and Imp4p. |
| GO:0034599 | 1 | Cellular response to oxidative stress | Biological Process | Any process that results in a change in state or activity of a cell (in terms of movement, secretion, enzyme production, gene expression, etc.) as a result of oxidative stress, a state often resulting from exposure to high levels of reactive oxygen species, e.g. superoxide anions, hydrogen peroxide (H2O2), and hydroxyl radicals. |
| GO:0035091 | 1 | Phosphatidylinositol binding | Molecular Function | Interacting selectively and non-covalently with any inositol-containing glycerophospholipid, i.e. phosphatidylinositol (PtdIns) and its phosphorylated derivatives. |
| GO:0035194 | 1 | Posttranscriptional gene silencing by RNA | Biological Process | Any process of posttranscriptional gene inactivation (silencing) mediated by small RNA molecules that may trigger mRNA degradation or negatively regulate translation. |
| GO:0035235 | 2 | Ionotropic glutamate receptor signaling pathway | Biological Process | A series of molecular signals initiated by glutamate binding to a glutamate receptor on the surface of the target cell, followed by the movement of ions through a channel in the receptor complex. Ends with regulation of a downstream cellular process, e.g. transcription. |
| GO:0035251 | 1 | UDP-glucosyltransferase activity | Molecular Function | Catalysis of the transfer of a glucosyl group from UDP-glucose to an acceptor molecule. |
| GO:0035335 | 3 | Peptidyl-tyrosine dephosphorylation | Biological Process | The removal of phosphoric residues from peptidyl-O-phospho-tyrosine to form peptidyl-tyrosine. |
| GO:0035336 | 2 | Long-chain fatty-acyl-CoA metabolic process | Biological Process | The chemical reactions and pathways involving long-chain fatty-acyl-CoAs, any derivative of coenzyme A in which the sulfhydryl group is in a thioester linkage with a long-chain fatty-acyl group. Long-chain fatty-acyl-CoAs have chain lengths of C13 or more. |
| GO:0035428 | 1 | Hexose transmembrane transport | Biological Process | The directed movement of hexose across a membrane by means of some agent such as a transporter or pore. Hexoses are any aldoses with a chain of six carbon atoms in the molecule. |
| GO:0035556 | 1 | Intracellular signal transduction | Biological Process | The process in which a signal is passed on to downstream components within the cell, which become activated themselves to further propagate the signal and finally trigger a change in the function or state of the cell. |
| GO:0035725 | 2 | Sodium ion transmembrane transport | Biological Process | A process in which a sodium ion is transported from one side of a membrane to the other by means of some agent such as a transporter or pore. |
| GO:0035999 | 2 | Tetrahydrofolate interconversion | Biological Process | The chemical reactions and pathways by which one-carbon (C1) units are transferred between tetrahydrofolate molecules, to synthesise other tetrahydrofolate molecules. |
| GO:0036092 | 2 | Phosphatidylinositol-3-phosphate biosynthetic process | Biological Process | The chemical reactions and pathways resulting in the formation of phosphatidylinositol-3-phosphate, a phosphatidylinositol monophosphate carrying the phosphate group at the 3-position. |
| GO:0042147 | 2 | Retrograde transport, endosome to Golgi | Biological Process | The directed movement of membrane-bounded vesicles from endosomes back to the trans-Golgi network where they are recycled for further rounds of transport. |
| GO:0042221 | 1 | Response to chemical | Biological Process | Any process that results in a change in state or activity of a cell or an organism (in terms of movement, secretion, enzyme production, gene expression, etc.) as a result of a chemical stimulus. |
| GO:0042281 | 1 | Dolichyl pyrophosphate Man9GlcNAc2 alpha-1,3-glucosyltransferase activity | Molecular Function | Catalysis of the addition of the first glucose residue to the lipid-linked oligosaccharide precursor for N-linked glycosylation; the transfer of glucose from dolichyl phosphate glucose (Dol-P-Glc) on to the lipid-linked oligosaccharide Man(9)GlcNAc(2)-PP-Dol. |
| GO:0042299 | 1 | Lupeol synthase activity | Molecular Function | Catalysis of the reaction: (S)-2,3-epoxysqualene = lupeol. This reaction is the cyclization of (S)-2,3-epoxysqualene (2,3-oxidosqualene) to lupeol. |
| GO:0042301 | 1 | Phosphate ion binding | Molecular Function | Interacting selectively and non-covalently with phosphate. |
| GO:0042335 | 2 | Cuticle development | Biological Process | The chemical reactions and pathways resulting in the formation of a cuticle, the outer layer of some animals and plants, which acts to prevent water loss. |
| GO:0042391 | 1 | Regulation of membrane potential | Biological Process | Any process that modulates the establishment or extent of a membrane potential, the electric potential existing across any membrane arising from charges in the membrane itself and from the charges present in the media on either side of the membrane. |
| GO:0042500 | 3 | Aspartic endopeptidase activity, intramembrane cleaving | Molecular Function | Catalysis of the hydrolysis of nonterminal peptide bonds in a polypeptide chain, occurring within a membrane. |
| GO:0042538 | 1 | Hyperosmotic salinity response | Biological Process | Any process that results in a change in state or activity of a cell or an organism (in terms of movement, secretion, enzyme production, gene expression, etc.) as a result of detection of, or exposure to, an increase in the concentration of salt (particularly but not exclusively sodium and chloride ions) in the environment. |
| GO:0042545 | 8 | Cell wall modification | Biological Process | The series of events leading to chemical and structural alterations of an existing cell wall that can result in loosening, increased extensibility or disassembly. |
| GO:0042578 | 2 | Phosphoric ester hydrolase activity | Molecular Function | Catalysis of the reaction: RPO-R' + H2O = RPOOH + R'H. This reaction is the hydrolysis of any phosphoric ester bond, any ester formed from orthophosphoric acid, O=P(OH)3. |
| GO:0042626 | 4 | ATPase activity, coupled to transmembrane movement of substances | Molecular Function | Catalysis of the reaction: ATP + H2O = ADP + phosphate, to directly drive the active transport of a substance across a membrane. |
| GO:0042719 | 1 | Mitochondrial intermembrane space protein transporter complex | Cellular Component | Soluble complex of the mitochondrial intermembrane space composed of various combinations of small Tim proteins; acts as a protein transporter to guide proteins to the Tim22 complex for insertion into the mitochondrial inner membrane. |
| GO:0042721 | 1 | Mitochondrial inner membrane protein insertion complex | Cellular Component | A multi-subunit complex embedded in the mitochondrial inner membrane that mediates insertion of carrier proteins into the inner membrane. |
| GO:0042732 | 1 | D-xylose metabolic process | Biological Process | The chemical reactions and pathways involving D-xylose, a naturally occurring plant polysaccharide. |
| GO:0042742 | 3 | Defense response to bacterium | Biological Process | Reactions triggered in response to the presence of a bacterium that act to protect the cell or organism. |
| GO:0042744 | 2 | Hydrogen peroxide catabolic process | Biological Process | The chemical reactions and pathways resulting in the breakdown of hydrogen peroxide (H2O2). |
| GO:0042765 | 1 | GPI-anchor transamidase complex | Cellular Component | An enzyme complex which in humans and yeast consists of at least five proteins; for example, the complex contains GAA1, GPI8, PIG-S, PIG-U, and PIG-T in human, and Gaa1p, Gab1p, Gpi8p, Gpi16p, and Gpi17p in yeast. Catalyzes the posttranslational attachment of the carboxyl-terminus of a precursor protein to a GPI-anchor. |
| GO:0042773 | 8 | ATP synthesis coupled electron transport | Biological Process | The transfer of electrons through a series of electron donors and acceptors, generating energy that is ultimately used for synthesis of ATP. |
| GO:0042787 | 7 | Protein ubiquitination involved in ubiquitin-dependent protein catabolic process | Biological Process | The process in which a ubiquitin group, or multiple groups, are covalently attached to the target protein, thereby initiating the degradation of that protein. |
| GO:0042803 | 5 | Protein homodimerization activity | Molecular Function | Interacting selectively and non-covalently with an identical protein to form a homodimer. |
| GO:0043085 | 1 | Positive regulation of catalytic activity | Biological Process | Any process that activates or increases the activity of an enzyme. |
| GO:0043130 | 1 | Ubiquitin binding | Molecular Function | Interacting selectively and non-covalently with ubiquitin, a protein that when covalently bound to other cellular proteins marks them for proteolytic degradation. |
| GO:0043161 | 4 | Proteasome-mediated ubiquitin-dependent protein catabolic process | Biological Process | The chemical reactions and pathways resulting in the breakdown of a protein or peptide by hydrolysis of its peptide bonds, initiated by the covalent attachment of ubiquitin, and mediated by the proteasome. |
| GO:0043167 | 3 | Ion binding | Molecular Function | Interacting selectively and non-covalently with ions, charged atoms or groups of atoms. |
| GO:0043168 | 1 | Anion binding | Molecular Function | Interacting selectively and non-covalently with anions, charged atoms or groups of atoms with a net negative charge. |
| GO:0043169 | 1 | Cation binding | Molecular Function | Interacting selectively and non-covalently with cations, charged atoms or groups of atoms with a net positive charge. |
| GO:0043170 | 3 | Macromolecule metabolic process | Biological Process | The chemical reactions and pathways involving macromolecules, any molecule of high relative molecular mass, the structure of which essentially comprises the multiple repetition of units derived, actually or conceptually, from molecules of low relative molecular mass. |
| GO:0043190 | 1 | ATP-binding cassette (ABC) transporter complex | Cellular Component | A complex for the transport of metabolites into and out of the cell, typically comprised of four domains; two membrane-associated domains and two ATP-binding domains at the intracellular face of the membrane, that form a central pore through the plasma membrane. Each of the four core domains may be encoded as a separate polypeptide or the domains can be fused in any one of a number of ways into multidomain polypeptides. In Bacteria and Archaebacteria, ABC transporters also include substrate binding proteins to bind substrate external to the cytoplasm and deliver it to the transporter. |
| GO:0043231 | 8 | Intracellular membrane-bounded organelle | Cellular Component | Organized structure of distinctive morphology and function, bounded by a single or double lipid bilayer membrane and occurring within the cell. Includes the nucleus, mitochondria, plastids, vacuoles, and vesicles. Excludes the plasma membrane. |
| GO:0043531 | 11 | ADP binding | Molecular Function | Interacting selectively and non-covalently with ADP, adenosine 5'-diphosphate. |
| GO:0043547 | 2 | Positive regulation of GTPase activity | Biological Process | Any process that activates or increases the activity of a GTPase. |
| GO:0043550 | 1 | Regulation of lipid kinase activity | Biological Process | Any process that modulates the frequency, rate or extent of lipid kinase activity, the catalysis of the transfer of a phosphate group, usually from ATP, to a simple or complex lipid. |
| GO:0043562 | 1 | Cellular response to nitrogen levels | Biological Process | Any process that results in a change in state or activity of a cell (in terms of movement, secretion, enzyme production, gene expression, etc.) as a result of a stimulus reflecting the presence, absence, or concentration of inorganic nitrogen. |
| GO:0043565 | 6 | Sequence-specific DNA binding | Molecular Function | Interacting selectively and non-covalently with DNA of a specific nucleotide composition, e.g. GC-rich DNA binding, or with a specific sequence motif or type of DNA e.g. promotor binding or rDNA binding. |
| GO:0043622 | 1 | Cortical microtubule organization | Biological Process | A process that is carried out at the cellular level which results in the assembly, arrangement of constituent parts, or disassembly of structures formed of microtubules and associated proteins in the cell cortex, i.e. just beneath the plasma membrane of a cell. |
| GO:0044212 | 1 | Transcription regulatory region DNA binding | Molecular Function | Interacting selectively and non-covalently with a DNA region that regulates the transcription of a region of DNA, which may be a gene, cistron, or operon. Binding may occur as a sequence specific interaction or as an interaction observed only once a factor has been recruited to the DNA by other factors. |
| GO:0044237 | 3 | Cellular metabolic process | Biological Process | The chemical reactions and pathways by which individual cells transform chemical substances. |
| GO:0044238 | 3 | Primary metabolic process | Biological Process | The chemical reactions and pathways involving those compounds which are formed as a part of the normal anabolic and catabolic processes. These processes take place in most, if not all, cells of the organism. |
| GO:0044260 | 1 | Cellular macromolecule metabolic process | Biological Process | The chemical reactions and pathways involving macromolecules, any molecule of high relative molecular mass, the structure of which essentially comprises the multiple repetition of units derived, actually or conceptually, from molecules of low relative molecular mass, as carried out by individual cells. |
| GO:0044267 | 1 | Cellular protein metabolic process | Biological Process | The chemical reactions and pathways involving a specific protein, rather than of proteins in general, occurring at the level of an individual cell. Includes cellular protein modification. |
| GO:0044281 | 3 | Small molecule metabolic process | Biological Process | The chemical reactions and pathways involving small molecules, any low molecular weight, monomeric, non-encoded molecule. |
| GO:0044424 | 7 | Intracellular part | Biological Process | The process in which a ubiquitin group, or multiple groups, are covalently attached to the target protein, thereby initiating the degradation of that protein. |
| GO:0044434 | 3 | Chloroplast part | Cellular Component | Any constituent part of a chloroplast, a chlorophyll-containing plastid with thylakoids organized into grana and frets, or stroma thylakoids, and embedded in a stroma. |
| GO:0044444 | 3 | Cytoplasmic part | Cellular Component | Any constituent part of the cytoplasm, all of the contents of a cell excluding the plasma membrane and nucleus, but including other subcellular structures. |
| GO:0044446 | 2 | Intracellular organelle part | Cellular Component | A constituent part of an intracellular organelle, an organized structure of distinctive morphology and function, occurring within the cell. Includes constituent parts of the nucleus, mitochondria, plastids, vacuoles, vesicles, ribosomes and the cytoskeleton but excludes the plasma membrane. |
| GO:0044550 | 3 | Secondary metabolite biosynthetic process | Biological Process | The chemical reactions and pathways resulting in the formation of secondary metabolites, the compounds that are not necessarily required for growth and maintenance of cells, and are often unique to a taxon. |
| GO:0044699 | 2 | Single-organism process | Biological Process | A biological process that involves only one organism. |
| GO:0044702 | 1 | Single organism reproductive process | Biological Process | A biological process that directly contributes to the process of producing new individuals, involving a single organism. |
| GO:0044707 | 1 | Single-multicellular organism process | Biological Process | A biological process occurring within a single, multicellular organism. |
| GO:0044710 | 2 | Single-organism metabolic process | Biological Process | A metabolic process - chemical reactions and pathways, including anabolism and catabolism, by which living organisms transform chemical substances - which involves a single organism. |
| GO:0044763 | 5 | Single-organism cellular process | Biological Process | Any process that is carried out at the cellular level, occurring within a single organism. |
| GO:0044767 | 1 | Single-organism developmental process | Biological Process | A biological process whose specific outcome is the progression of an integrated living unit: an anatomical structure (which may be a subcellular structure, cell, tissue, or organ), or organism over time from an initial condition to a later condition, involving only one organism. |
| GO:0045039 | 1 | Protein import into mitochondrial inner membrane | Biological Process | The process comprising the import of proteins into the mitochondrion from outside the organelle and their insertion into the mitochondrial inner membrane. The translocase of the outer membrane complex mediates the passage of these proteins across the outer membrane, after which they are guided by either of two inner membrane translocase complexes into their final destination in the inner membrane. |
| GO:0045116 | 2 | Protein neddylation | Biological Process | Covalent attachment of the ubiquitin-like protein NEDD8 (RUB1) to another protein. |
| GO:0045156 | 4 | Electron transporter, transferring electrons within the cyclic electron transport pathway of photosynthesis activity | Molecular Function | Enables the directed movement of electrons within the cyclic electron transport pathway of photosynthesis. |
| GO:0045174 | 1 | Glutathione dehydrogenase (ascorbate) activity | Molecular Function | Catalysis of the reaction: dehydroascorbate + 2 glutathione = L-ascorbate + glutathione disulfide. |
| GO:0045261 | 6 | Proton-transporting ATP synthase complex, catalytic core F(1) | Cellular Component | The sector of a hydrogen-transporting ATP synthase complex in which the catalytic activity resides; it comprises the catalytic core and central stalk, and is peripherally associated with a membrane, such as the plasma membrane or the mitochondrial inner membrane, when the entire ATP synthase is assembled. |
| GO:0045263 | 1 | Proton-transporting ATP synthase complex, coupling factor F(o) | Cellular Component | All non-F1 subunits of a hydrogen-transporting ATP synthase, including integral and peripheral membrane proteins. |
| GO:0045330 | 3 | Aspartyl esterase activity | Molecular Function | Catalysis of the hydrolysis of an ester bond by a mechanism involving a catalytically active aspartic acid residue. |
| GO:0045454 | 4 | Cell redox homeostasis | Biological Process | Any process that maintains the redox environment of a cell or compartment within a cell. |
| GO:0045490 | 5 | Pectin catabolic process | Biological Process | The chemical reactions and pathways resulting in the breakdown of pectin, a polymer containing a backbone of alpha-1,4-linked D-galacturonic acid residues. |
| GO:0045551 | 2 | Cinnamyl-alcohol dehydrogenase activity | Molecular Function | Catalysis of the reaction: cinnamyl alcohol + NADP+ = cinnamaldehyde + NADPH + H+. |
| GO:0045552 | 1 | Dihydrokaempferol 4-reductase activity | Molecular Function | Catalysis of the reaction: cis-3,4-leucopelargonidin + NADP+ = (+)-dihydrokaempferol + NADPH + H+. |
| GO:0045735 | 2 | Nutrient reservoir activity | Molecular Function | Functions in the storage of nutritious substrates. |
| GO:0045892 | 4 | Negative regulation of transcription, DNA-templated | Biological Process | Any process that stops, prevents, or reduces the frequency, rate or extent of cellular DNA-templated transcription. |
| GO:0045893 | 1 | Positive regulation of transcription, DNA-templated | Biological Process | Any process that activates or increases the frequency, rate or extent of cellular DNA-templated transcription. |
| GO:0045903 | 5 | Positive regulation of translational fidelity | Biological Process | Any process that increases the ability of the translational apparatus to interpret the genetic code. |
| GO:0045944 | 2 | Positive regulation of transcription from RNA polymerase II promoter | Biological Process | Any process that activates or increases the frequency, rate or extent of transcription from an RNA polymerase II promoter. |
| GO:0046081 | 1 | dUTP catabolic process | Biological Process | The chemical reactions and pathways resulting in the breakdown of dUTP, deoxyuridine (5'-)triphosphate. |
| GO:0046488 | 1 | Phosphatidylinositol metabolic process | Biological Process | The chemical reactions and pathways involving phosphatidylinositol, any glycophospholipid in which a sn-glycerol 3-phosphate residue is esterified to the 1-hydroxyl group of 1D-myo-inositol. |
| GO:0046564 | 1 | Oxalate decarboxylase activity | Molecular Function | Catalysis of the reaction: H(+) + oxalate = CO(2) + formate. |
| GO:0046604 | 1 | Positive regulation of mitotic centrosome separation | Biological Process | Any process that activates or increases the frequency, rate or extent of centrosome separation. |
| GO:0046658 | 7 | Anchored component of plasma membrane | Cellular Component | The component of the plasma membrane consisting of the gene products that are tethered to the membrane only by a covalently attached anchor, such as a lipid group, that is embedded in the membrane. Gene products with peptide sequences that are embedded in the membrane are excluded from this grouping. |
| GO:0046777 | 1 | Protein autophosphorylation | Biological Process | The phosphorylation by a protein of one or more of its own amino acid residues (cis-autophosphorylation), or residues on an identical protein (trans-autophosphorylation). |
| GO:0046835 | 2 | Carbohydrate phosphorylation | Biological Process | The process of introducing a phosphate group into a carbohydrate, any organic compound based on the general formula Cx(H2O)y. |
| GO:0046836 | 1 | Glycolipid transport | Biological Process | The directed movement of glycolipids, compounds containing (usually) 1-4 linked monosaccharide residues joined by a glycosyl linkage to a lipid, into, out of or within a cell, or between cells, by means of some agent such as a transporter or pore. |
| GO:0046854 | 1 | Phosphatidylinositol phosphorylation | Biological Process | The process of introducing one or more phosphate groups into a phosphatidylinositol, any glycerophosphoinositol having one phosphatidyl group esterified to one of the hydroxy groups of inositol. |
| GO:0046856 | 1 | Phosphatidylinositol dephosphorylation | Biological Process | The process of removing one or more phosphate groups from a phosphatidylinositol. |
| GO:0046872 | 46 | Metal ion binding | Molecular Function | Interacting selectively and non-covalently with any metal ion. |
| GO:0046873 | 1 | Metal ion transmembrane transporter activity | Molecular Function | Enables the transfer of metal ions from one side of a membrane to the other. |
| GO:0046910 | 1 | Pectinesterase inhibitor activity | Molecular Function | Stops, prevents or reduces the activity of any pectinesterase enzyme. |
| GO:0046914 | 2 | Transition metal ion binding | Molecular Function | Interacting selectively and non-covalently with a transition metal ions; a transition metal is an element whose atom has an incomplete d-subshell of extranuclear electrons, or which gives rise to a cation or cations with an incomplete d-subshell. Transition metals often have more than one valency state. Biologically relevant transition metals include vanadium, manganese, iron, copper, cobalt, nickel, molybdenum and silver. |
| GO:0046916 | 2 | Cellular transition metal ion homeostasis | Biological Process | Any process involved in the maintenance of an internal steady state of transition metal ions at the level of a cell. A transition metal is an element whose atom has an incomplete d-subshell of extranuclear electrons, or which gives rise to a cation or cations with an incomplete d-subshell. Transition metals often have more than one valency state. Biologically relevant transition metals include vanadium, manganese, iron, copper, cobalt, nickel, molybdenum and silver. |
| GO:0046933 | 6 | Proton-transporting ATP synthase activity, rotational mechanism | Molecular Function | Catalysis of the transfer of protons from one side of a membrane to the other according to the reaction: ADP + H2O + phosphate + H+(in) = ATP + H+(out), by a rotational mechanism. |
| GO:0046961 | 6 | Proton-transporting ATPase activity, rotational mechanism | Molecular Function | Catalysis of the transfer of protons from one side of a membrane to the other according to the reaction: ATP + H2O + H+(in) = ADP + phosphate + H+(out), by a rotational mechanism. |
| GO:0046982 | 3 | Protein heterodimerization activity | Molecular Function | Interacting selectively and non-covalently with a nonidentical protein to form a heterodimer. |
| GO:0046983 | 39 | Protein dimerization activity | Molecular Function | The formation of a protein dimer, a macromolecular structure consists of two noncovalently associated identical or nonidentical subunits. |
| GO:0047184 | 2 | 1-acylglycerophosphocholine O-acyltransferase activity | Molecular Function | Catalysis of the reaction: 1-acyl-sn-glycero-3-phosphocholine + acyl-CoA = phosphatidylcholine + CoA. |
| GO:0047372 | 2 | Acylglycerol lipase activity | Molecular Function | Catalysis of the reaction: H2O + acylglycerol = a fatty acid + glycerol. |
| GO:0047792 | 1 | Cyanohydrin beta-glucosyltransferase activity | Molecular Function | Catalysis of the reaction: UDP-glucose + (S)-4-hydroxymandelonitrile = UDP + (S)-4-hydroxy-mandelonitrile beta-D-glucoside. |
| GO:0047800 | 2 | Cysteamine dioxygenase activity | Molecular Function | Catalysis of the reaction: cysteamine + O(2) = H(+) + hypotaurine. |
| GO:0048027 | 1 | mRNA 5'-UTR binding | Molecular Function | Interacting selectively and non-covalently with the 5' untranslated region of an mRNA molecule. |
| GO:0048038 | 8 | Quinone binding | Molecular Function | Interacting selectively and non-covalently with a quinone, any member of a class of diketones derivable from aromatic compounds by conversion of two CH groups into CO groups with any necessary rearrangement of double bonds. |
| GO:0048046 | 6 | Apoplast | Cellular Component | The cell membranes and intracellular regions in a plant are connected through plasmodesmata, and plants may be described as having two major compartments: the living symplast and the non-living apoplast. The apoplast is external to the plasma membrane and includes cell walls, intercellular spaces and the lumen of dead structures such as xylem vessels. Water and solutes pass freely through it. |
| GO:0048278 | 2 | Vesicle docking | Biological Process | The initial attachment of a transport vesicle membrane to the target membrane, mediated by proteins protruding from the membrane of the vesicle and the target membrane. Docking requires only that the two membranes come close enough for these proteins to interact and adhere. |
| GO:0048364 | 1 | Root development | Biological Process | The process whose specific outcome is the progression of the root over time, from its formation to the mature structure. The root is the water- and mineral-absorbing part of a plant which is usually underground, does not bear leaves, tends to grow downwards and is typically derived from the radicle of the embryo. |
| GO:0048366 | 3 | Leaf development | Biological Process | The process whose specific outcome is the progression of the leaf over time, from its formation to the mature structure. |
| GO:0048367 | 1 | Shoot system development | Biological Process | The process whose specific outcome is the progression of the shoot system over time, from its formation to the mature structure. |
| GO:0048522 | 1 | Positive regulation of cellular process | Biological Process | Any process that activates or increases the frequency, rate or extent of a cellular process, any of those that are carried out at the cellular level, but are not necessarily restricted to a single cell. For example, cell communication occurs among more than one cell, but occurs at the cellular level. |
| GO:0048544 | 5 | Recognition of pollen | Biological Process | The process, involving the sharing and interaction of the single locus incompatibility haplotypes, involved in the recognition or rejection of the self pollen by cells in the stigma. This process ensures out-breeding in certain plant species. |
| GO:0048573 | 1 | Photoperiodism, flowering | Biological Process | A change from the vegetative to the reproductive phase as a result of detection of, or exposure to, a period of light or dark of a given length. The length of the period of light or dark required to initiate the change is set relative to a particular duration known as the 'critical day length'. The critical day length varies between species. |
| GO:0048608 | 1 | Reproductive structure development | Biological Process | The reproductive developmental process whose specific outcome is the progression of structures that will be used in the process of creating new individuals from one or more parents, from their formation to the mature structures. |
| GO:0048856 | 1 | Anatomical structure development | Biological Process | The biological process whose specific outcome is the progression of an anatomical structure from an initial condition to its mature state. This process begins with the formation of the structure and ends with the mature structure, whatever form that may be including its natural destruction. An anatomical structure is any biological entity that occupies space and is distinguished from its surroundings. Anatomical structures can be macroscopic such as a carpel, or microscopic such as an acrosome. |
| GO:0050017 | 1 | L-3-cyanoalanine synthase activity | Molecular Function | Catalysis of the reaction: L-cysteine + HCN = sulfide + L-3-cyanoalanine. |
| GO:0050113 | 6 | Inositol oxygenase activity | Molecular Function | Catalysis of the reaction: myo-inositol + O(2) = D-glucuronate + H(2)O + H(+). |
| GO:0050136 | 1 | NADH dehydrogenase (quinone) activity | Molecular Function | Catalysis of the reaction: NADH + H+ + a quinone = NAD+ + a quinol. |
| GO:0050174 | 1 | Phenylalanine decarboxylase activity | Molecular Function | Catalysis of the reaction: L-phenylalanine = phenylethylamine + CO2. |
| GO:0050242 | 2 | Pyruvate, phosphate dikinase activity | Molecular Function | Catalysis of the reaction: ATP + phosphate + pyruvate = AMP + diphosphate + 2 H(+) + phosphoenolpyruvate. |
| GO:0050350 | 1 | Prihydroxystilbene synthase activity | Molecular Function | Catalysis of the reaction: 3 malonyl-CoA + 4-coumaroyl-CoA = 4 CoA + 3,4',5-trihydroxy-stilbene + 4 CO2. |
| GO:0050373 | 2 | UDP-arabinose 4-epimerase activity | Molecular Function | Catalysis of the reaction: UDP-L-arabinose = UDP-alpha-D-xylose. |
| GO:0050508 | 1 | Glucuronosyl-N-acetylglucosaminyl-proteoglycan 4-alpha-N-acetylglucosaminyltransferase activity | Molecular Function | Catalysis of the reaction: beta-D-glucuronosyl-(1,4)-N-acetyl-alpha-D-glucosaminyl-proteoglycan + UDP-N-acetyl-D-glucosamine = N-acetyl-alpha-D-glucosaminyl-(1,4)-beta-D-glucuronosyl-(1,4)-N-acetyl-alpha-D-glucosaminyl-proteoglycan + UDP. |
| GO:0050567 | 1 | Glutaminyl-tRNA synthase (glutamine-hydrolyzing) activity | Molecular Function | Catalysis of the reaction: L-glutamine + glutamyl-tRNA(Gln) + ATP = L-glutamate + glutaminyl-tRNA(Gln) + phosphate + ADP. |
| GO:0050660 | 4 | Flavin adenine dinucleotide binding | Molecular Function | Interacting selectively and non-covalently with FAD, flavin-adenine dinucleotide, the coenzyme or the prosthetic group of various flavoprotein oxidoreductase enzymes, in either the oxidized form, FAD, or the reduced form, FADH2. |
| GO:0050661 | 4 | NADP binding | Molecular Function | Interacting selectively and non-covalently with nicotinamide-adenine dinucleotide phosphate, a coenzyme involved in many redox and biosynthetic reactions; binding may be to either the oxidized form, NADP+, or the reduced form, NADPH. |
| GO:0050662 | 3 | Coenzyme binding | Molecular Function | Interacting selectively and non-covalently with a coenzyme, any of various nonprotein organic cofactors that are required, in addition to an enzyme and a substrate, for an enzymatic reaction to proceed. |
| GO:0050664 | 1 | Oxidoreductase activity, acting on NAD(P)H, oxygen as acceptor | Molecular Function | Catalysis of an oxidation-reduction (redox) reaction in which NADH or NADPH acts as a hydrogen or electron donor and reduces an oxygen molecule. |
| GO:0050789 | 2 | Regulation of biological process | Biological Process | Any process that modulates the frequency, rate or extent of a biological process. Biological processes are regulated by many means; examples include the control of gene expression, protein modification or interaction with a protein or substrate molecule. |
| GO:0050794 | 1 | Regulation of cellular process | Biological Process | Any process that modulates the frequency, rate or extent of a cellular process, any of those that are carried out at the cellular level, but are not necessarily restricted to a single cell. For example, cell communication occurs among more than one cell, but occurs at the cellular level. |
| GO:0050821 | 1 | Protein stabilization | Biological Process | Any process involved in maintaining the structure and integrity of a protein and preventing it from degradation or aggregation. |
| GO:0050832 | 2 | Defense response to fungus | Biological Process | Reactions triggered in response to the presence of a fungus that act to protect the cell or organism. |
| GO:0050896 | 3 | Response to stimulus | Biological Process | Any process that results in a change in state or activity of a cell or an organism (in terms of movement, secretion, enzyme production, gene expression, etc.) as a result of a stimulus. The process begins with detection of the stimulus and ends with a change in state or activity or the cell or organism. |
| GO:0050897 | 1 | Cobalt ion binding | Molecular Function | Interacting selectively and non-covalently with a cobalt (Co) ion. |
| GO:0051013 | 1 | Microtubule severing | Biological Process | The process in which a microtubule is broken down into smaller segments. |
| GO:0051026 | 2 | Chiasma assembly | Biological Process | The cell cycle process in which a connection between chromatids assembles, indicating where an exchange of homologous segments has taken place by the crossing-over of non-sister chromatids. |
| GO:0051082 | 1 | Unfolded protein binding | Molecular Function | Interacting selectively and non-covalently with an unfolded protein. |
| GO:0051090 | 1 | Regulation of sequence-specific DNA binding transcription factor activity | Biological Process | Any process that modulates the frequency, rate or extent of the activity of a transcription factor, any factor involved in the initiation or regulation of transcription. |
| GO:0051103 | 2 | DNA ligation involved in DNA repair | Biological Process | The re-formation of a broken phosphodiester bond in the DNA backbone, carried out by DNA ligase, that contributes to DNA repair. |
| GO:0051119 | 2 | Sugar transmembrane transporter activity | Molecular Function | Catalysis of the transfer of a sugar from one side of the membrane to the other. A sugar is any member of a class of sweet, water-soluble, crystallizable carbohydrates, which are the monosaccharides and smaller oligosaccharides. |
| GO:0051213 | 1 | Dioxygenase activity | Molecular Function | Catalysis of an oxidation-reduction (redox) reaction in which both atoms of oxygen from one molecule of O2 are incorporated into the (reduced) product(s) of the reaction. The two atoms of oxygen may be distributed between two different products. |
| GO:0051276 | 1 | Chromosome organization | Biological Process | A process that is carried out at the cellular level that results in the assembly, arrangement of constituent parts, or disassembly of chromosomes, structures composed of a very long molecule of DNA and associated proteins that carries hereditary information. This term covers covalent modifications at the molecular level as well as spatial relationships among the major components of a chromosome. |
| GO:0051287 | 5 | NAD binding | Molecular Function | Interacting selectively and non-covalently with nicotinamide adenine dinucleotide, a coenzyme involved in many redox and biosynthetic reactions; binding may be to either the oxidized form, NAD+, or the reduced form, NADH. |
| GO:0051301 | 1 | Cell division | Biological Process | The process resulting in division and partitioning of components of a cell to form more cells; may or may not be accompanied by the physical separation of a cell into distinct, individually membrane-bounded daughter cells. |
| GO:0051443 | 2 | Positive regulation of ubiquitin-protein transferase activity | Biological Process | Any process that activates, maintains or increases the rate of ubiquitin transferase activity. |
| GO:0051453 | 2 | Regulation of intracellular pH | Biological Process | Any process that modulates the internal pH of a cell, measured by the concentration of the hydrogen ion. |
| GO:0051537 | 1 | 2 iron, 2 sulfur cluster binding | Molecular Function | Interacting selectively and non-covalently with a 2 iron, 2 sulfur (2Fe-2S) cluster; this cluster consists of two iron atoms, with two inorganic sulfur atoms found between the irons and acting as bridging ligands. |
| GO:0051539 | 5 | 4 iron, 4 sulfur cluster binding | Molecular Function | Interacting selectively and non-covalently with a 4 iron, 4 sulfur (4Fe-4S) cluster; this cluster consists of four iron atoms, with the inorganic sulfur atoms found between the irons and acting as bridging ligands. |
| GO:0051603 | 5 | Proteolysis involved in cellular protein catabolic process | Biological Process | The hydrolysis of a peptide bond or bonds within a protein as part of the chemical reactions and pathways resulting in the breakdown of a protein by individual cells. |
| GO:0051716 | 1 | Cellular response to stimulus | Biological Process | Any process that results in a change in state or activity of a cell (in terms of movement, secretion, enzyme production, gene expression, etc.) as a result of a stimulus. The process begins with detection of the stimulus by a cell and ends with a change in state or activity or the cell. |
| GO:0051726 | 1 | Regulation of cell cycle | Biological Process | Any process that modulates the rate or extent of progression through the cell cycle. |
| GO:0051731 | 1 | Polynucleotide 5'-hydroxyl-kinase activity | Molecular Function | Catalysis of the reaction: NTP + 5'-dephosphopolynucleotide = NDP + 5'-phosphopolynucleotide. The polynucleotide may be DNA or RNA. |
| GO:0051861 | 1 | Glycolipid binding | Molecular Function | Interacting selectively and non-covalently with a glycolipid, any compound containing one or more monosaccharide residues bound by a glycosidic linkage to a hydrophobic group such as an acylglycerol, a sphingoid, a ceramide (N-acylsphingoid) or a prenyl phosphate. |
| GO:0052544 | 1 | Defense response by callose deposition in cell wall | Biological Process | Any process in which callose is transported to, and/or maintained in, the cell wall during the defense response. Callose is a linear 1,3-beta-d-glucan formed from UDP-glucose and is found in certain plant cell walls. |
| GO:0052696 | 2 | Flavonoid glucuronidation | Biological Process | The modification of a flavonoid by the conjugation of glucuronic acid. The resultant flavonoid glucuronosides are often much more water-soluble than the precursor. |
| GO:0052727 | 2 | Capsanthin synthase activity | Molecular Function | Catalysis of the reaction: antheraxanthin = capsanthin. |
| GO:0052728 | 2 | Capsorubin synthase activity | Molecular Function | Catalysis of the reaction: violaxanthin = capsorubin. |
| GO:0052747 | 2 | Sinapyl alcohol dehydrogenase activity | Molecular Function | Catalysis of the reaction: sinapaldehyde + NADPH + H+ = sinapyl-alcohol + NADP+. |
| GO:0052886 | 1 | 9,9'-dicis-carotene:quinone oxidoreductase activity | Molecular Function | Catalysis of the reaction: 9,9'-di-cis-zeta-carotene + a quinone = 7,9,9'-tri-cis-neurosporene + a quinol. |
| GO:0052887 | 1 | 7,9,9'-tricis-neurosporene:quinone oxidoreductase activity | Molecular Function | Catalysis of the reaction: 7,9,9'-tri-cis-neurosporene + a quinone = 7,9,7',9'-tetra-cis-lycopene + a quinol. |
| GO:0052889 | 1 | 9,9'-di-cis-zeta-carotene desaturation to 7,9,7',9'-tetra-cis-lycopene | Biological Process | The series of reactions in which 9,9'-di-cis-zeta-carotene is desaturated to 7,9,9'-tri-cis-neurosporene, and then 7,9,7',9'-tetra-cis-lycopene. The overall reaction for this process is: 9,9'-di-cis-zeta-carotene + 2 quinone = 2 quinol + 7,9,7',9'-tetra-cis-lycopene. |
| GO:0055074 | 1 | Calcium ion homeostasis | Biological Process | Any process involved in the maintenance of an internal steady state of calcium ions within an organism or cell. |
| GO:0055075 | 1 | Potassium ion homeostasis | Biological Process | Any process involved in the maintenance of an internal steady state of potassium ions within an organism or cell. |
| GO:0055085 | 23 | Transmembrane transport | Biological Process | The process in which a solute is transported across a lipid bilayer, from one side of a membrane to the other |
| GO:0055114 | 97 | Oxidation-reduction process | Biological Process | A metabolic process that results in the removal or addition of one or more electrons to or from a substance, with or without the concomitant removal or addition of a proton or protons. |
| GO:0061077 | 2 | Chaperone-mediated protein folding | Biological Process | The process of inhibiting aggregation and assisting in the covalent and noncovalent assembly of single chain polypeptides or multisubunit complexes into the correct tertiary structure that is dependent on interaction with a chaperone. |
| GO:0061630 | 7 | Ubiquitin protein ligase activity | Molecular Function | Catalysis of the transfer of ubiquitin to a substrate protein via the reaction X-ubiquitin + S -> X + S-ubiquitin, where X is either an E2 or E3 enzyme, the X-ubiquitin linkage is a thioester bond, and the S-ubiquitin linkage is an amide bond: an isopeptide bond between the C-terminal glycine of ubiquitin and the epsilon-amino group of lysine residues in the substrate or, in the linear extension of ubiquitin chains, a peptide bond the between the C-terminal glycine and N-terminal methionine of ubiquitin residues. |
| GO:0070181 | 1 | Small ribosomal subunit rRNA binding | Molecular Function | Interacting selectively and non-covalently with the small ribosomal subunit RNA (SSU rRNA), a constituent of the small ribosomal subunit. In S. cerevisiae, this is the 18S rRNA. |
| GO:0070449 | 1 | Elongin complex | Cellular Component | A transcription elongation factor complex that suppresses RNA polymerase II pausing, and may act by promoting proper alignment of the 3'-end of nascent transcripts with the polymerase catalytic site. Consists of a transcriptionally active Elongin A subunit (abut 100 kDa)and two smaller Elongin B (about 18 kDa) and Elongin C (about 15 kDa)subunits. |
| GO:0070469 | 4 | Respiratory chain | Cellular Component | The protein complexes that form the electron transport system (the respiratory chain), associated with a cell membrane, usually the plasma membrane (in prokaryotes) or the inner mitochondrial membrane (on eukaryotes). The respiratory chain complexes transfer electrons from an electron donor to an electron acceptor and are associated with a proton pump to create a transmembrane electrochemical gradient. |
| GO:0070481 | 2 | Nuclear-transcribed mRNA catabolic process, non-stop decay | Biological Process | The chemical reactions and pathways resulting in the breakdown of the transcript body of a nuclear-transcribed mRNA that is lacking a stop codon. |
| GO:0070483 | 2 | Detection of hypoxia | Biological Process | The series of events in which a stimulus indicating lowered oxygen tension is received by a cell and converted into a molecular signal. Hypoxia, defined as a decline in O2 levels below normoxic levels of 20.8 - 20.95%, results in metabolic adaptation at both the cellular and organismal level. |
| GO:0070681 | 1 | Glutaminyl-tRNAGln biosynthesis via transamidation | Biological Process | A tRNA aminoacylation process in which glutaminyl-tRNAGln is formed by a tRNA-dependent two-step pathway. In the first step a non-discriminating glutamyl-tRNAGlx synthetase generates the misacylated L-glutamyl-tRNAGln species, and in the second step it is amidated to the correctly charged L-glutaminyl-tRNAGln by a glutamyl-tRNAGln amidotransferase. |
| GO:0070772 | 1 | PAS complex | Cellular Component | A class III phosphatidylinositol 3-kinase complex that contains a phosphatidylinositol-3-phosphate 5-kinase subunit (Fab1p in yeast; PIKfyve in mammals), a kinase activator, and a phosphatase, and may also contain additional proteins; it is involved in regulating the synthesis and turnover of phosphatidylinositol 3,5-bisphosphate. In mammals the complex is composed of PIKFYVE, FIG4 and VAC14. In yeast it is composed of Atg18p, Fig4p, Fab1p, Vac14p and Vac7p. |
| GO:0070918 | 1 | Production of small RNA involved in gene silencing by RNA | Biological Process | The process in which a double-stranded RNA precursor is processed into short (20-30 nt) fragments. RNA cleavage is catalyzed by a Dicer endonuclease. |
| GO:0070966 | 2 | Nuclear-transcribed mRNA catabolic process, no-go decay | Biological Process | The chemical reactions and pathways resulting in the breakdown of the transcript body of a nuclear-transcribed mRNA with stalls in translation elongation. |
| GO:0070993 | 1 | Translation preinitiation complex | Cellular Component | A ribonucleoprotein complex that contains the small ribosomal subunit, a translation initiation ternary complex (i.e. an initiator tRNA, GTP, and an IF2 or eIF2 complex), and an mRNA. |
| GO:0071025 | 2 | RNA surveillance | Biological Process | The set of processes involved in identifying and degrading defective or aberrant RNAs. |
| GO:0071281 | 1 | Cellular response to iron ion | Biological Process | Any process that results in a change in state or activity of a cell (in terms of movement, secretion, enzyme production, gene expression, etc.) as a result of an iron ion stimulus. |
| GO:0071421 | 1 | Manganese ion transmembrane transport | Biological Process | A process in which a manganese ion is transported from one side of a membrane to the other by means of some agent such as a transporter or pore. |
| GO:0071458 | 3 | Integral component of cytoplasmic side of endoplasmic reticulum membrane | Cellular Component | The component of the endoplasmic reticulum membrane consisting of the gene products that penetrate only the cytoplasmic side of the membrane. |
| GO:0071554 | 2 | Cell wall organization or biogenesis | Biological Process | A process that results in the biosynthesis of constituent macromolecules, assembly, arrangement of constituent parts, or disassembly of a cell wall. |
| GO:0071555 | 5 | Cell wall organization | Biological Process | A process that results in the assembly, arrangement of constituent parts, or disassembly of the cell wall, the rigid or semi-rigid envelope lying outside the cell membrane of plant, fungal and most prokaryotic cells, maintaining their shape and protecting them from osmotic lysis. |
| GO:0071556 | 3 | Integral component of lumenal side of endoplasmic reticulum membrane | Cellular Component | The component of the endoplasmic reticulum membrane consisting of the gene products that penetrate only the lumenal side of the membrane. |
| GO:0071704 | 4 | Organic substance metabolic process | Biological Process | The chemical reactions and pathways involving an organic substance, any molecular entity containing carbon. |
| GO:0071805 | 5 | Potassium ion transmembrane transport | Biological Process | A process in which a potassium ion is transported from one side of a membrane to the other. |
| GO:0071897 | 4 | DNA biosynthetic process | Biological Process | The cellular DNA metabolic process resulting in the formation of DNA, deoxyribonucleic acid, one of the two main types of nucleic acid, consisting of a long unbranched macromolecule formed from one or two strands of linked deoxyribonucleotides, the 3'-phosphate group of each constituent deoxyribonucleotide being joined in 3',5'-phosphodiester linkage to the 5'-hydroxyl group of the deoxyribose moiety of the next one. |
| GO:0071949 | 2 | FAD binding | Molecular Function | Interacting selectively and non-covalently with the oxidized form, FAD, of flavin-adenine dinucleotide, the coenzyme or the prosthetic group of various flavoprotein oxidoreductase enzymes. |
| GO:0072321 | 1 | Chaperone-mediated protein transport | Biological Process | The directed movement of proteins into, out of or within a cell, or between cells, mediated by chaperone molecules that bind to the transported proteins. |
| GO:0080008 | 2 | Cul4-RING E3 ubiquitin ligase complex | Cellular Component | A ubiquitin ligase complex in which a cullin from the Cul4 family and a RING domain protein form the catalytic core; substrate specificity is conferred by an adaptor protein. |
| GO:0080019 | 2 | Fatty-acyl-CoA reductase (alcohol-forming) activity | Molecular Function | Catalysis of the reaction: a very long chain fatty acyl-CoA + NADPH + H+ = a very long chain primary alcohol + NADP+ + CoA. |
| GO:0080041 | 1 | ADP-ribose pyrophosphohydrolase activity | Molecular Function | Catalysis of the reaction: ADP-ribose + H2O = AMP + ribose-1-phosphate. |
| GO:0080042 | 1 | ADP-glucose pyrophosphohydrolase activity | Molecular Function | Catalysis of the reaction: ADP-glucose + H2O = AMP + glucose-1-phosphate. |
| GO:0080043 | 2 | Quercetin 3-O-glucosyltransferase activity | Molecular Function | Catalysis of the transfer of a glucosyl group from UDP-glucose to the 3-hydroxy group of a quercetin molecule. |
| GO:0080044 | 2 | Quercetin 7-O-glucosyltransferase activity | Molecular Function | Catalysis of the transfer of a glucosyl group from UDP-glucose to the 7-hydroxy group of a quercetin molecule. |
| GO:0080051 | 1 | Cutin transport | Biological Process | The directed movement of cutin into, out of or within a cell, or between cells, by means of some agent such as a transporter or pore. Cutin, which consists of C16-18 fatty acids, is the major component of the cuticle that covers the plant surface. |
| GO:0090304 | 1 | Nucleic acid metabolic process | Biological Process | Any cellular metabolic process involving nucleic acids. |
| GO:0090305 | 5 | Nucleic acid phosphodiester bond hydrolysis | Biological Process | The nucleic acid metabolic process in which the phosphodiester bonds between nucleotides are cleaved by hydrolysis. |
| GO:0090575 | 1 | RNA polymerase II transcription factor complex | Cellular Component | A transcription factor complex that acts at promoters of genes transcribed by RNA polymerase II. |
| GO:0097157 | 1 | Pre-mRNA intronic binding | Molecular Function | Interacting selectively and non-covalently with an intronic sequence of a pre-messenger RNA (pre-mRNA). |
| GO:0097159 | 1 | Organic cyclic compound binding | Molecular Function | Interacting selectively and non-covalently with an organic cyclic compound, any molecular entity that contains carbon arranged in a cyclic molecular structure. |
| GO:0097428 | 1 | Protein maturation by iron-sulfur cluster transfer | Biological Process | The transfer of an assembled iron-sulfur cluster from a scaffold protein to an acceptor protein that contributes to the attainment of the full functional capacity of a protein. |
| GO:0097502 | 2 | Mannosylation | Biological Process | The covalent attachment of a mannose residue to a substrate molecule. |
| GO:0097602 | 2 | Cullin family protein binding | Molecular Function | Interacting selectively and non-covalently with any member of the cullin family, hydrophobic proteins that act as scaffolds for ubiquitin ligases (E3). |
| GO:0098656 | 1 | Anion transmembrane transport | Biological Process | A process in which an anion is transported from one side of a membrane to the other by means of some agent such as a transporter or pore. |
| GO:0098869 | 6 | Cellular oxidant detoxification | Biological Process | Any process carried out at the cellular level that reduces or removes the toxicity superoxide radicals or hydrogen peroxide. |
| GO:0102483 | 1 | Scopolin beta-glucosidase activity | Molecular Function | Catalysis of the reaction: H2O + scopolin <=> beta-D-glucose + scopoletin |
| GO:1900027 | 1 | Regulation of ruffle assembly | Biological Process | Any process that modulates the frequency, rate or extent of ruffle assembly. |
| GO:1901002 | 1 | Positive regulation of response to salt stress | Biological Process | Any process that activates or increases the frequency, rate or extent of response to salt stress. |
| GO:1901363 | 1 | Heterocyclic compound binding | Molecular Function | Interacting selectively and non-covalently with heterocyclic compound. |
| GO:1901564 | 1 | Organonitrogen compound metabolic process | Biological Process | The chemical reactions and pathways involving organonitrogen compound. |
| GO:1902600 | 5 | Hydrogen ion transmembrane transport | Biological Process | The directed movement of hydrogen ion (proton) across a membrane. |
| GO:1903830 | 1 | Magnesium ion transmembrane transport | Biological Process | The directed movement of magnesium ion across a membrane. |
| GO:1905392 | 1 | Plant organ morphogenesis | Biological Process | The developmental process by which a plant organ is generated and organized. |
| GO:1990189 | 1 | Peptide-serine-N-acetyltransferase activity | Molecular Function | Catalysis of the reaction: acetyl-CoA + N-terminal L-serine in peptide = CoA + N-acetyl-L-serine-peptide. |
| GO:1990190 | 1 | Peptide-glutamate-N-acetyltransferase activity | Molecular Function | Catalysis of the reaction: acetyl-CoA + N-terminal L-glutamate in peptide = CoA + N-acetyl-L-glutamate-peptide. |
| GO:2000023 | 1 | Regulation of lateral root development | Biological Process | Any process that modulates the frequency, rate or extent of lateral root development. |
| GO:2000031 | 1 | Regulation of salicylic acid mediated signaling pathway | Biological Process | Any process that modulates the frequency, rate or extent of salicylic acid mediated signaling pathway. |
| GO:2001295 | 2 | Malonyl-CoA biosynthetic process | Biological Process | The chemical reactions and pathways resulting in the formation of malonyl-CoA, the S-malonyl derivative of coenzyme A. |
